# Supplementary material for: Mechanistic analysis and kinetic profiling of Soai’s asymmetric autocatalysis for pyridyl and pyrimidyl substrates
Source: Nat Commun. 2025 Aug 7;16:7303. doi: 10.1038/s41467-025-62591-3 (PMC12332059; doi:10.1038/s41467-025-62591-3)
Supplement: Supplementary file 1 — Supplementary Information [file 41467_2025_62591_MOESM1_ESM.pdf]

Supplementary Information

Mechanistic Analysis and Kinetic Profiling of Soai's Asymmetric Autocatalysis for Pyridyl and Pyrimidyl Substrates

Patrick Möhler,[1] Gloria Betzenbichler,[1] Laura Huber,[1] Alexander F. Siegle,[1] Oliver Trapp[1,2,*]

Affiliations:

[1]Department of Chemistry, Ludwig-Maximilians-University Munich, Munich, Germany.

[2]Max Planck Institute for Astronomy, Heidelberg, Germany.

*Corresponding author: Email: oliver.trapp@cup.uni-muenchen.de

Content

1 Materials and Methods 4
1.1 General 4
1.2 NMR Spectroscopy 4
1.3 Mass Spectrometry 4
1.4 Preparative HPLC 4
1.5 HPLC Analysis 5
2 Synthetic Procedures 6
2.1 6-((Trimethylsilyl)ethynyl)nicotinaldehyde **(TMSPyr-CHO)** 6
2.2 2-Methyl-(6-((trimethylsilyl)ethynyl)pyridine-3-yl)propanol (**TMSPyr-OH**) 6
2.3 5-Bromo-2-((trimethylsilyl)ethynyl)pyrimidine (**TMSPym-Br**) 7
2.4 2-(Ethynyltrimethyllsilane)-pyrimidine-5-carbaldehyde (**TMSPym-CHO**) 8
2.5 2-Methyl-((2-trimethylsilylalkynyl)-5-pyrimidinyl)propanol **(TMSPym-OH)** 8
2.6 Ethinyl Adamantane 9
2.7 6-((Adamantan-1-yl)ethynyl)nicotinaldehyde (**AdPyr-CHO**) 10
2.8 1-(6-((Adamantan-1-yl)ethynyl)pyridin-3-yl)-2-methylpropan-1-ol (**AdPyr-OH**) 10
2.9 5-Bromo-2-((adamantyl)ethynyl)pyrimidine (**AdPym-Br**) 11
2.10 2-(Ethynyl-adamantyl)-pyrimidine-5-carbaldehyde (**AdPym-CHO**) 12
2.11 1-(2-((Adamantan-1-yl)ethynyl)pyrimidin-5-yl)-2-methylpropan-1-ol (**AdPym-OH**) 12
3 *In situ* Reaction – High-Resolution MS measurements 14
3.1 General procedure for Orbitrap *In situ* High-resolution MS measurements 14
3.2 Time resolved tracking of substrate aldehyde, product alcohol and zinc hemiacetal 14
3.3 Identification of Reaction Intermediates 16
3.3.1 Intermediates of the TMSPyr autocatalytic system 16
3.3.2 Intermediates of the AdPyr autocatalytic system 26
3.3.3 Intermediates of the TMSPym autocatalytic system 30
3.3.4 Intermediates of the AdPym autocatalytic system 36
4 Kinetic Investigations 39
4.1 General procedure for flow injection analysis (FIA) HPLC measurements 39
4.2 TMSPyr-CHO/TMSPyr-OH System 40
4.2.1 Calibration plots for quantitative analysis of kinetic measurements 40
4.2.2 Variation of the TMSPyr-CHO concentration 41
4.2.3 Variation of the TMSPyr-OH concentration 46
4.2.4 Determination of the Reaction Orders 50

4.3 AdPyr-CHO/AdPyr-OH System 51
4.3.1 Calibration plots for quantitative analysis of kinetic measurements 51
4.3.2 Variation of the AdPyr-CHO concentration 52
4.3.3 Variation of the AdPyr-OH concentration 57
4.3.4 Determination of the Reaction Orders 61
4.4 AdPym-CHO/AdPym-OH System 62
4.4.1 Calibration plots for quantitative analysis of kinetic measurements 62
4.4.2 Variation of the AdPym-CHO concentration 63
4.4.3 Variation of the AdPym-OH concentration 68
4.4.4 Determination of the Reaction Orders 71
4.5 TMSPym-CHO/TMSPym-OH System 72
4.5.1 Calibration plots for quantitative analysis of kinetic measurements 72
4.5.2 Variation of the TMSPym-CHO concentration 73
4.5.3 Variation of the TMSPym-OH concentration 77
4.5.4 Determination of the Reaction Orders 80
5 Dynamic HPLC measurements of hemiacetal formation 81
5.1 Enantioselective dynamic HPLC measurements 81
5.2 Evaluation of the dynamic HPLC profiles 81
5.3 DHPLC measurements of Soai aldehyde TMSPyr-CHO 82
5.4 DHPLC measurements of Soai aldehyde TMSPym-CHO 83
5.5 DHPLC measurements of Soai aldehyde AdPyr-CHO 84
5.6 DHPLC measurements of Soai aldehyde AdPym-CHO 85
6 Kinetic Analysis and Simulation of Reaction Profiles 86
6.1 Reaction Rates of the Side Reaction 86
6.2 Mechanistic Model and Algorithm for the Kinetic Analysis of the Soai Reaction 90
6.3 Summary of the Reaction Rates Determined from the Experimental Data 96
6.4 Simulation of concentration-time profiles 97
6.4.1 TMSPyr-CHO/TMSPyr-OH System 97
6.4.2 AdPyr-CHO/AdPyr-OH System 111
6.4.3 TMSPym-CHO/TMSPym-OH System 125
6.4.4 AdPym-CHO/AdPym-OH System 137
7 NMR Spectra 149
8 References 159

1 Materials and Methods

1.1 General

All reactions involving the use of moisture and/or oxygen sensitive substances were carried out in glassware, previously dried with a heat-gun under argon atmosphere, using standard *Schlenk* techniques. Syringes were used to transfer solvents or reagents and purged three times with argon prior to use. All reagents were obtained from *Sigma-Aldrich* and *abcr* and were used without further purification. The dry solvents toluene and THF were taken from the solvent purification system MB SPS-800 and stored under argon.

1.2 NMR Spectroscopy

NMR spectra were recorded on a *Bruker AVIII HD400* and a *Varian vnmrs* spectrometer. Chemical shifts are reported as δ-values in ppm referenced to the residual solvent peak.[1] For the characterization of the observed signal multiplicities the following abbreviations were used: m (multiplet), s (singlet), d (doublet), t (triplet), p (pentet), dd (doublet of doublet) and dt (doublet of triplet).

1.3 Mass Spectrometry

High-resolution mass spectrometry was performed using flow injection analysis (FIA/ESI) with a *Surveyor* MS pump at a flow rate of 100 µl/min with acetonitrile/water as the running agent. Each 1-10 µL sample solution was injected using an inline filter. EI spectra were recorded on *Finnigan MAT 95Q* or *Finnigan MAT 90* instruments for electron impact ionization (EI). *In situ* mass spectrometric studies were performed using a *Q Exactive Plus* Orbitrap mass spectrometer (*Thermo Scientific*).

1.4 Preparative HPLC

Preparative separations were performed on an *Agilent 1260 Infinity* HPLC. Achiral stationary phases were purchased from *Chiral Technologies*. The HPLC-grade solvents were obtained from *Sigma-Aldrich*.

The enantiomers of the racemic TMS pyridine alcohol **TMSPyr-OH** were separated using a Chiralpak® IG-3 column (250 mm, i.D. 20 mm, particle size: 5 µm) and *n*-hexane/*i*PrOH = 90/10 at a flow rate of 18.9 mL/min.

The enantiomers of the racemic TMS pyrimidine alcohol **TMSPym-OH** were separated using a Chiralpak® IB column (250 mm, i.D. 20 mm, particle size: 5 µm) and *n*-hexane/*i*PrOH = 95/5 at a flow rate of 18.9 mL/min.

The enantiomers of the racemic adamantyl pyridine alcohol **AdPyr-OH** were separated using a Chiralpak® IC column (250 mm, i.D. 20 mm, particle size: 5 µm) and *n*-hexane/*i*PrOH = 75/25 at a flow rate of 18.9 mL/min.

The enantiomers of the racemic adamantyl pyrimidine alcohol **AdPym-OH** were separated using a Chiralpak® IB column (250 mm, i.D. 20 mm, particle size: 5 µm) and *n*-hexane/*i*PrOH = 75/25 at a flow rate of 18.9 mL/min.

1.5 HPLC Analysis

HPLC and HPLC-MS measurements were performed on an *Agilent 1200 Infinity* HPLC device equipped with a photodiode array detector (DAD) and a 6120 quadrupole mass spectrometer (APCI). The kinetic measurements of the *Soai* reaction were performed using the Flow Injection Analysis (FIA) method.

2 Synthetic Procedures

2.1 6-((Trimethylsilyl)ethynyl)nicotinaldehyde **(TMSPyr-CHO)**

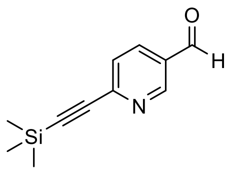

This synthesis is a modification of a reported procedure.[2] 6-Bromo-pyridine-3-carboxaldehyde (1.86 g, 10.0 mmol, 1.00 equiv.), $Pd(PPh_3)_4$ (231 mg, 0.50 mmol, 5 mol%) and CuI (95.2 mg, 531 µmol, 5 mol%) were dissolved in degassed THF (25 mL). The mixture was cooled in an ice bath and *N*,*N*-diisopropylethylamine (6.80 mL, 40.0 mmol, 4.00 equiv.) was added. After the mixture was stirred at 0 °C for 5 min, trimethylsilylacetylene (1.52 mg, 11.0 mmol, 1.10 equiv.) was added dropwise. The ice bath was removed after 1 h and the mixture was stirred at room temperature for 6 h. Afterwards, the reaction mixture was filtered through Celite. The solvents were removed under reduced pressure. Purification of the crude material was achieved by sublimation (0.015 mbar, 65-70 °C) to obtain **TMSPyr-CHO** as a white crystalline solid (1.36 g, 6.70 mmol, 77%).

^{1}H-NMR ($CDCl_3$, 400 MHz): δ [ppm] = 10.10 (d, J = 0.5 Hz, 1H), 9.02 (dd, J = 2.2, 0.9 Hz, 1H), 8.12 (dd, J = 8.1, 2.1 Hz, 1H), 7.60 (dt, J = 8.1, 0.8 Hz, 1H), 0.29 (s, 9H).

^{13}C-NMR ($CDCl_3$, 400 MHz): δ [ppm] = 189.3, 152.2, 147.9, 135.5, 130.9, 127.6, 103.6, 100.1, 0.5.

HR-MS (EI): m/z calc. for $C_{11}H_{13}NOSi$: 203.0766; found: 188.0524.

2.2 2-Methyl-(6-((trimethylsilyl)ethynyl)pyridine-3-yl)propanol (**TMSPyr-OH**)

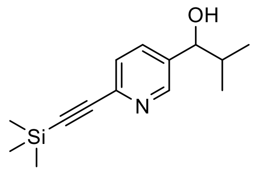

This synthesis is a modification of a reported procedure.[3] **TMSPyr-CHO** (1.08 g, 5.31 mmol, 1.00 equiv.) was dissolved in dry THF (50 mL). The solution was cooled to 0 °C and *i*PrMgCl solution (1.88 M in diethyl ether, 3.39 mL, 6.37 mmol, 1.20 equiv.) was added dropwise. The reaction mixture was stirred at room temperature for 2 h and quenched by the addition of saturated NH_4Cl solution (20 mL). The mixture was diluted with water (50 mL) and stirred for a further 10 min. The aqueous and organic layers were separated and the aqueous layer was extracted with ethyl acetate (3x 50 mL). The combined organic layers were dried over Na_2SO_4, filtrated and the solvents were removed under reduced pressure. The crude product was purified *via* column chromatography (pentane/acetone, 90/10) to yield the product **TMSPyr-OH** (0.72 g, 2.91 mmol, 55%) as an orange oil which solidified over time.

^{1}H-NMR ($CDCl_3$, 400 MHz): δ [ppm] = 8.48 (dt, J = 2.2, 0.7 Hz, 1H), 7.66 (ddd, J= 8.0, 2.2, 0.6 Hz, 1H), 7.45 (dd, J = 8.1, 0.8 Hz, 1H), 4.48 (d, J = 6.3 Hz, 1H), 1.95 (m, 1H) 0.95 (d, J = 6.7 Hz, 3H), 0.83 (d, J = 6.8 Hz, 3H), 0.27 (s, 9H).

^{13}C-NMR ($CDCl_3$, 151 MHz): δ [ppm] = 148.1, 141.5, 138.6, 134.6, 126.9, 103.1, 95.5, 77.0, 35.3, 18.6, 17.6, -0.3.

HR-MS (EI): m/z calc. for $C_{14}H_{21}NOSi$: 247.1392, found: 248.1463 $[M+H]^+$.

2.3 5-Bromo-2-((trimethylsilyl)ethynyl)pyrimidine (**TMSPym-Br**)

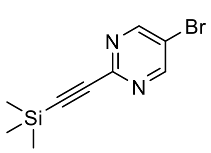

This synthesis is a modification of a reported procedure.[4] Diisopropylamine (7.73 mL, 54.8 mmol, 4.00 equiv.), CuI (156 mg, 0.82 mmol, 6 mol%), $Pd(PPh_3)_4$ (475 mg, 0.41 mmol, 3 mol%) and 5-bromo-2-iodo-pyrimidine **6** (3.90 g, 13.7 mmol, 1.00 equiv.) were dissolved in degassed THF (60 mL). The mixture was cooled to 0 °C before dropwise adding trimethylsilylacetylene (2.10 mL, 15.1 mmol, 1.10 equiv.). After 30 min the ice bath was removed and the reaction mixture was stirred at room temperature for 15 h. The reaction mixture was diluted with diethyl ether (100 mL), filtered through Celite and rewashed with diethyl ether (100 mL). After removal of the solvents under reduced pressure, the crude product was purified *via* flash column chromatography (cyclohexane/ethyl acetate, 33/1) to give compound **TMSPym-Br** as a white solid (2.94 g, 11.5 mmol, 84%).

^{1}H-NMR ($CDCl_3$, 400 MHz): δ [ppm] = 8.75 (s, 2H), 0.29 (s, 9H).

^{13}C-NMR ($CDCl_3$, 151 MHz): δ [ppm] = 157.9, 150.2, 119.5, 101.3, 96.2, -0.5.

HR-MS (EI): m/z calc. for $C_9H_{11}BrN_2Si$: 253.9875, found: 253.9869.

2.4 2-(Ethynyltrimethyllsilane)-pyrimidine-5-carbaldehyde (**TMSPym-CHO**)

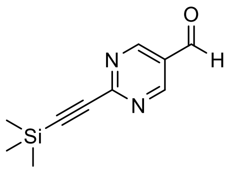

This synthesis is a modification of a reported procedure.[5] **TMSPym-Br** (1.02 g, 4.00 mmol, 1.00 equiv.) was dissolved in dry THF (40 mL). Ethyl formate (0.48 mL, 6.00 mmol, 1.50 equiv.) was added at −78 °C. After 5 min, *n*BuLi (1.90 M in hexane, 3.16 mL, 6.00 mmol, 1.50 equiv.) was added dropwise. The mixture was stirred for 5 min and subsequently quenched by the addition of conc. acetic acid (0.35 mL). The mixture was warmed to room temperature and saturated Na_2CO_3 solution (20 mL) was added. The aqueous and organic layers were separated and the aqueous layer was extracted with CH_2Cl_2 (3x 25 mL). The solvents were removed under reduced pressure and the obtained crude material was purified *via* column chromatography (cyclohexane/ethyl acetate, 10/1). **TMSPym-CHO** was obtained as a beige solid (363 mg, 1.76 mmol, 45%).

^{1}H-NMR ($CDCl_3$, 400 MHz): δ [ppm] = 10.14 (s, 1H), 9.14 (s, 2H), 0.32 (s, 9H).

^{13}C-NMR ($CDCl_3$, 151 MHz): δ [ppm] = 188.3, 158.4, 155.4, 126.7, 102.0, 100.0, -0.5.

HR-MS (EI): m/z calc. for $C_{10}H_{12}ON_2Si$: 204.0719, found: 204.0718.

2.5 2-Methyl-((2-trimethylsilylalkynyl)-5-pyrimidinyl)propanol **(TMSPym-OH)**

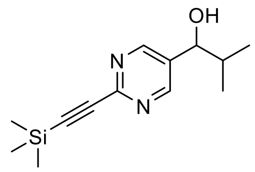

This synthesis is a modification of a reported procedure.[4] **TMSPym-Br** (1.02 g, 4.00 mmol, 1.00 equiv.) was dissolved in dry THF (30 mL). The mixture was cooled to −97 °C (MeOH/N_2) and *n*BuLi (1.5 M in hexane, 2.93 mL, 4.40 mmol, 1.10 equiv.) was added dropwise. After the addition, the mixture was stirred at −97 °C for 10 min before dropwise adding isobutyraldehyde (0.44 mL, 4.80 mmol, 1.20 equiv.) and stirring at −97 °C for a further 30 min. The reaction was quenched by adding HCl (2 M in Et_2O, 2.00 mL, 4.00 mmol, 1.00 equiv.). Afterwards, saturated Na_2CO_3 solution (5 mL) was added. The aqueous layer was separated from the organic layer and extracted with ethyl acetate (3x 25 mL). The combined organic layers were dried over Na_2SO_4, filtrated and the solvents were removed under reduced pressure. Purification of the crude product was performed by column chromatography using a CH_2Cl_2/ethyl acetate solvent gradient (40/1 - 30/1 - 20/1 - 10/1). The product **TMSPym-OH** was obtained as yellow oil which solidified over time (0.23 g, 0.91 mmol, 23%).

^{1}H-NMR (CDCl$_3$, 400 MHz): δ [ppm] = 8.66 (s, 2H), 4.54 (d, J = 5.9 Hz, 1H), 2.03 – 1.94 (m, 1H), 0.95 (d, J = 6.7 Hz, 3H), 0.89 (d, J = 6.8 Hz, 3H), 0.29 (s, 9H).

^{13}C-NMR (CDCl$_3$, 151 MHz): δ [ppm] = 155.6, 134.8, 75.3, 35.2, 18.4, 17.2, -0.4.

HR-MS (EI): m/z calc. for $C_{13}H_{20}N_2OSi$: 248.1345, found: 248.1339.

2.6 Ethinyl Adamantane

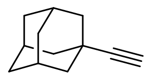

This synthesis is a modification of a reported procedure.[6] A LDA solution (1.00 M in THF, 15.0mL, 15.0 mmol, 1.00 equiv.) was cooled to −78 °C and a solution of adamantyl methyl ketone (2.67 g, 15.0 mmol, 1.00 equiv.) in dry THF (7 mL) was added dropwise. After stirring for 1 h, chloro diethyl phosphate (2.16 mL, 15.0 mmol, 1.00 equiv.) was added dropwise and the reaction mixture was allowed to warm to room temperature. After stirring for 3 h, this mixture was added dropwise to a second LDA solution (1.00 M in THF, 30˙0 mL, 30.0 mmol, 2.00 equiv.) at −78 °C. The solution was stirred at room temperature for 15 h. The reaction was quenched by the addition of water (50 mL). After separating the organic and aqueous layers, the aqueous layer was extracted with pentane (3x 50 mL). The combined organic extracts were washed with ice-cold aqueous HCl (1M, 2x 70 mL) and saturated $NaHCO_3$ solution (100 mL). After drying over Na_2SO_4 and filtration through Celite, the solvents were removed under reduced pressure. Purification was achieved by flash column chromatography (cyclohexane/ethyl acetate, 10/1). Ethinyl adamantane was obtained as white solid (1.97 g, 12.3 mmol, 82%).

^{1}H-NMR (CDCl$_3$, 400 MHz): δ [ppm] = 2.10 (s, 1H), 1.96 (t, J = 3.2 Hz, 3H), 1.89 (d, J = 2.9 Hz, 6H), 1.69 (t, J = 3.2 Hz, 6H).

^{13}C-NMR (CDCl$_3$, 151 MHz): δ [ppm] = 93.2, 66.7, 42.8, 36.4, 29.5, 28.0.

HR-MS (EI): m/z calc. for $C_{12}H_{16}$: 160.1252, found: 160.1245.

2.7 6-((Adamantan-1-yl)ethynyl)nicotinaldehyde (**AdPyr-CHO**)

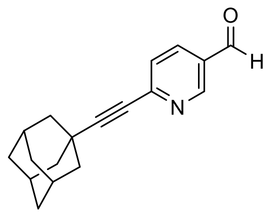

This synthesis is a modification of a reported procedure.[2] Ethinyl adamantane (1.06 g, 6.60 mmol, 1.10 equiv.) was dissolved in degassed THF (5 mL). In a separate *Schlenk* flask, 6-bromonicotinaldehyde (1.12 g, 6.00 mmol, 1.00 equiv.), CuI (57.1 mg, 300 µmol, 5 mol%) and $Pd(PPh_3)_4$ (13 mg, 120 µmol, 0.02 equiv.) were dissolved in dry THF (50 mL). Diisopropylamine (3.20 mL, 24.0 mmol, 4.00 equiv.) was added and the mixture was degassed by freeze-pump thaw (liquid N2, 3 times). The mixture was cooled to 0 °C, and the ethinyl adamantane solution was added. After stirring at room temperature for 24 h, the reaction mixture was filtered through Celite. After removal of the solvents under reduced pressure, the crude material was purified *via* column chromatography (cyclohexane/ethyl acetate, 10/1) to yield compound **AdPyr-CHO** as a white solid (1.32 g, 4.98 mmol, 83%).

^{1}H-NMR ($CDCl_3$, 400 MHz): δ [ppm] = 10.08 (s, 1H), 8.99 (d, J = 2.2 Hz, 1H), 8.10 (dd, J = 8.1, 2.1 Hz, 1H), 7.52 (d, J = 8.1 Hz, 1H), 2.01 (d, J = 1.3 Hz, 10H), 1.73 (d, J = 2.4 Hz, 6H).

^{13}C-NMR ($CDCl_3$, 151 MHz): δ [ppm] = 197.4, 189.8, 151.9, 135.8, 129.5, 127.3, 77.3, 77.0, 76.7, 42.1, 36.2, 30.3, 27.7.

HR-MS (EI): m/z calc. for $C_{18}H_{19}NO$: 265.1467, found: 265.1462.

2.8 1-(6-((Adamantan-1-yl)ethynyl)pyridin-3-yl)-2-methylpropan-1-ol (**AdPyr-OH**)

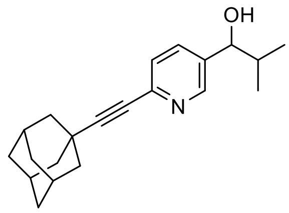

This synthesis is a modification of a reported procedure.[3] **AdPyr-CHO** (796 mg, 3.00 mmol, 1.00 equiv.) was dissolved in dry THF (30 mL). The solution was cooled to 0 °C before dropwise adding *i*PrMgCl solution (2.00 M in diethyl ether, 1.80mL, 3.60 mmol, 1.20 equiv.). The mixture was stirred at room temperature for 2 h before quenching the reaction with saturated NH_4Cl solution (15 mL). The mixture was diluted with water (30 mL) and stirred for a further 10 min. The aqueous and organic layers were separated and the aqueous layer was extracted with ethyl acetate (3x 30 mL). The solvents were removed under reduced pressure. The obtained crude material was purified by column chromatography (cyclohexane/ethyl acetate, 2/1) to yield compound **AdPyr/OH** as orange solid (433 mg, 1.39 mmol, 46%).

^{1}H-NMR ($CDCl_3$, 600 MHz): δ [ppm] = 8.45 (d, J = 2.2 Hz, 1H), 7.63 (dd, J = 8.1, 2.2 Hz, 1H), 7.37 (d, J = 8.0 Hz, 1H), 4.46 (d, J =6.4 Hz, 1H), 2.04 (s, 6H), 1.99 (s, 9H), 1.97 – 1.92 (m, 1H), 0.95 (d, J =6.7 Hz, 3H), 0.82 (d, J =6.8 Hz, 3H).

^{13}C-NMR ($CDCl_3$, 151 MHz): δ [ppm] = 147.9, 142.5, 137.9, 134.7, 126.7, 99.2, 78.8, 77.1, 42.4, 36.4, 35.3, 30.1, 27.9, 18.7, 17.9.

HR-MS (ESI): m/z calc. for $C_{21}H_{27}NO$: 309.2093, found: 309.2086.

2.9 5-Bromo-2-((adamantyl)ethynyl)pyrimidine (**AdPym-Br**)

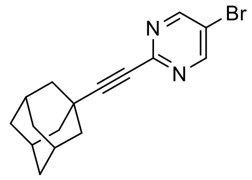

This synthesis is a modification of a reported procedure.[4] 5-Bromo-2-iodo-pyrimidine (1.20 g, 4.23 mmol, 1.00 equiv.), CuI (32.2 mg, 0.17 mmol, 4 mol%) and $Pd(PPh_3)_4$ (97.7 mg, 84.5 µmol, 2 mol%) were dissolved in degassed THF (18 mL). To this mixture, diisopropylamine (2.39 mL, 16.9 mmol, 4.00 equiv.) and ethinyl adamantane (745 mg, 4.65 mmol, 1.10 equiv.) were added at 0 °C. Afterwards, the reaction mixture was stirred at room temperature for 48 h, filtered through Celite and the solvents were removed under reduced pressure. Purification of the crude product was achieved *via* column chromatography (cyclohexane/ ethyl acetate, 50/1) to yield the product **AdPym-Br** as a white solid (1.32 g, 4.16 mmol, 90%).

^{1}H-NMR ($CDCl_3$, 600 MHz): δ [ppm] = 8.72 (s, 2H), 2.01 (s, 9H), 1.72 (s, 6H).

^{13}C-NMR ($CDCl_3$, 151 MHz): δ [ppm] = 157.9, 151.4, 118.6, 99.3, 78.3, 42.0, 36.3, 30.2, 27.8.

HR-MS (EI): m/z calc. for $C_{16}H_{17}N_2Br$: 316.0575, found: 316.0563.

2.10 2-(Ethynyl-adamantyl)-pyrimidine-5-carbaldehyde (**AdPym-CHO**)

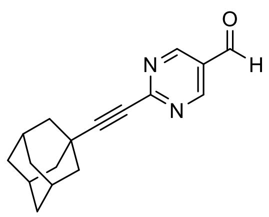

This synthesis is a modification of a reported procedure.[5] **AdPym-Br** (700 mg, 2.21 mmol, 1.00 equiv.) was dissolved in dry THF (20 mL). The solution was cooled to −78 °C and ethyl formate (0.27 mL, 3.31 mmol, 1.5 equiv.) was added and the mixture was stirred for 3 min. Afterwards, *n*BuLi (2.5 M in hexane, 1.32 mL, 3.31 mmol, 1.50 equiv.) was added over 10 min and the mixture was stirred at −78 °C for further 15 min. The reaction was quenched by the dropwise addition of acetic acid (17.0 M, 0.19 mL, 3.31 mmol, 1.50 equiv.) and saturated Na_2CO_3 solution (20 mL) was added. The organic and aqueous layers were separated and the aqueous layer was extracted with CH_2Cl_2 (3x 20 mL). The organic phase was dried over Na_2SO_4, filtered off and the solvents were removed under reduced pressure. Purification of the crude product was performed *via* column chromatography (cyclohexane/ethyl acetate, 20/1). Product **AdPym-CHO** was obtained as a white solid (295 mg, 1.11 mmol, 51%).

^{1}H-NMR ($CDCl_3$, 600 MHz): δ [ppm] = 10.12 (s, 1H), 9.10 (s, 2H), 2.07 – 2.00 (s, 9H), 1.74 (d, J = 2.9 Hz, 6H).

^{13}C-NMR ($CDCl_3$, 151 MHz): δ [ppm] = 188.6, 158.6, 156.8, 126.5, 103.1, 79.7, 42.1, 36.4, 30.6, 27.9, 27.2.

HR-MS (EI): m/z calc. for $C_{17}H_{18}N_2O$: 266.1419, found: 266.1415.

2.11 1-(2-((Adamantan-1-yl)ethynyl)pyrimidin-5-yl)-2-methylpropan-1-ol (**AdPym-OH**)

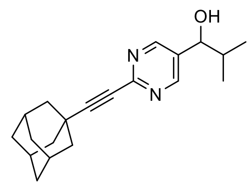

This synthesis is a modification of a reported procedure.[3] **AdPym-Br** (1.01 g, 3.18 mmol, 1.00 equiv.) was dissolved in dry THF (30 mL) and the solution was cooled to −97 °C (MeOH/ liquid N2). To this mixture, *n*BuLi (2.3 M in hexane, 1.66 mL, 3.82 mmol, 1.20 equiv.) was added dropwise. After stirring the mixture at −97 °C for 10 min, isobutyraldehyde (0.35 mL, 3.82 mmol, 1.20 equiv.) was added dropwise. The mixture was stirred for another 20 min before the reaction was quenched by adding HCl (2 M in Et_2O, 1.60 mL, 3.2 mmol, 1.00 equiv.). After adding saturated Na_2CO_3 solution (10 mL), the layers were separated. The aqueous layer was extracted with ethyl acetate (3x 20 mL). The combined organic layer was dried over Na_2SO_4, filtrated and the solvents were removed under reduced pressure. Purification

of the crude material was achieved by column chromatography (cyclohexane/ ethyl acetate, 8/1). Compound **AdPym-OH** was obtained as a white solid (182 mg, 0.59 mmol, 19%).

^{1}H-NMR ($CDCl_3$, 600 MHz): δ [ppm] = 8.62 (s, 2H), 4.51 (d, J = 6.0 Hz, 1H), 2.04 – 1.93 (m, 10H), 1.71 (t, J = 3.1 Hz, 6H), 0.94 (d, J = 6.7 Hz, 3H), 0.87 (d, J = 6.8 Hz, 3H).

^{13}C-NMR ($CDCl_3$, 151 MHz): δ [ppm] = 155.7, 152.0, 134.3, 98.3, 78.6, 75.3, 42.1, 36.3, 35.3, 30.1, 27.8, 18.5, 17.5.

HR-MS (ESI): m/z calc. for $C_{20}H_{27}N_2O$ $[M+H]^+$: 311.2118, found: 311.2123.

3 *In situ* Reaction – High-Resolution MS measurements

3.1 General procedure for Orbitrap *In situ* High-resolution MS measurements

The measurements were carried out according to a previously decribed procedure.[7] An HPLC vial was filled with a toluene stock solution of the *Soai* aldehyde and enantiopure alcohol under argon atmosphere. Immediately after the addition of the diisopropylzinc solution the reaction mixture (total volume 1 mL) was vortexed and injected *via* a syringe pump (10 µL/min) into Orbitrap mass spectrometer. A valve switched in fixed time intervals (30 s or 1 min) to change between reaction mixture and anhydrous toluene (200 µL/min). APCI was used for ionization under mild conditions with N_2 at an ion source temperature of 150 °C. The **TMSPyr**, **TMSPym** and **AdPym** systems were measured at concentrations of 30 mM aldehyde and 1.5 mM alcohol (*ee* >99%). The **AdPyr** system was measured at 40 mM aldehyde and 3 mM alcohol (*ee* >99%).

3.2 Time resolved tracking of substrate aldehyde, product alcohol and zinc hemiacetal

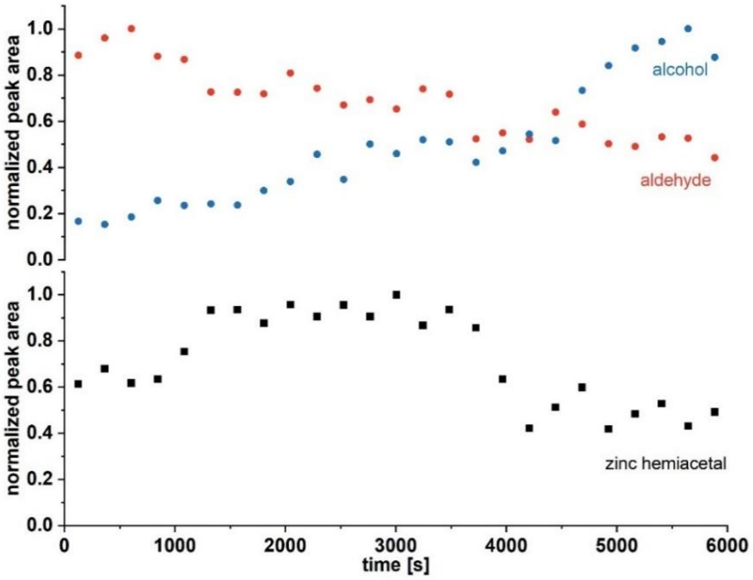

Supplementary Figure 1: Normalized peak areas of the aldehyde **AdPyr-CHO** and the alcohol **AdPyr-OH** (top) and the zinc hemiacetalate complex I_5 (bottom) plotted against time. Reaction conditions: 40.0 mM **TMSPym-CHO**, 1.5 mM **TMSPym-OH** (*ee* > 99%) and 40 mM *i*Pr_2Zn in toluene at r.t.

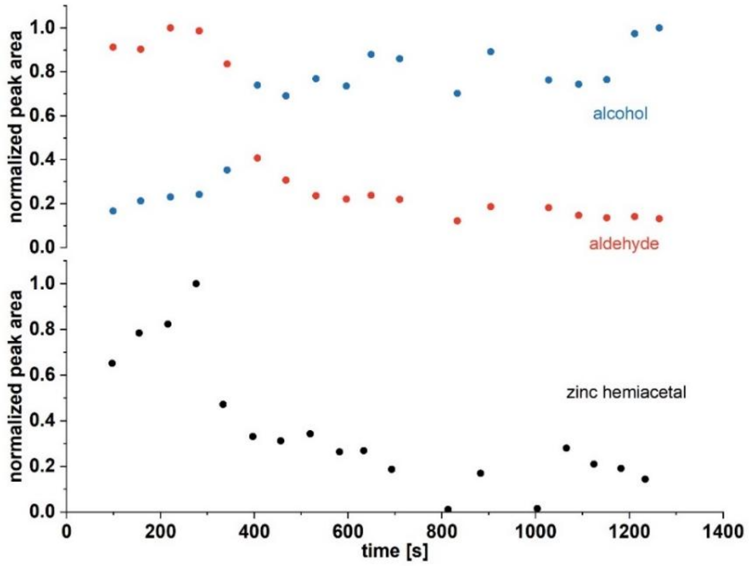

Supplementary Figure 2: Normalized peak areas of the aldehyde **TMSPym-CHO** and the alcohol **TMSPym-OH** (top) and the zinc hemiacetalate complex I_5 (bottom) plotted against time. Reaction conditions: 30.0 mM **TMSPym-CHO**, 1.5 mM **TMSPym-OH** (*ee* > 99%) and 40 mM *i*Pr_2Zn in toluene at r.t.

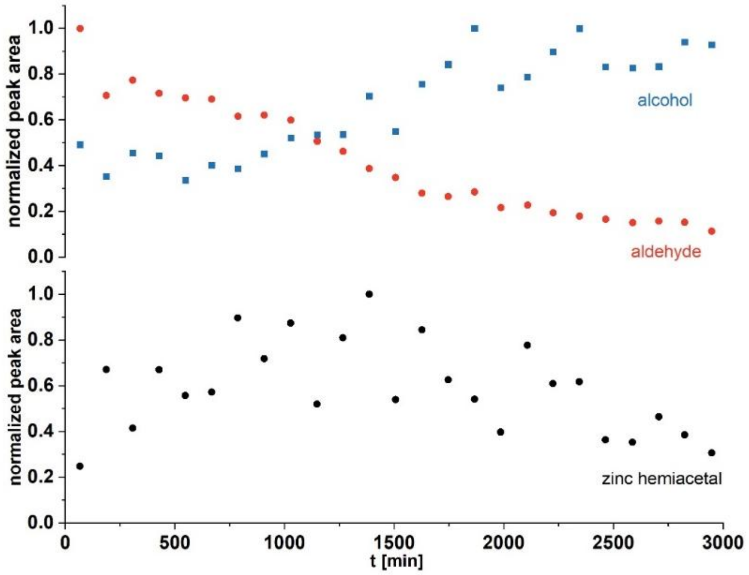

Supplementary Figure 3: Normalized peak areas of the aldehyde **AdPym-CHO** and the alcohol **AdPym-OH** (top) and the zinc hemiacetalate complex I_5 (bottom) plotted against time. Reaction conditions: 30.0 mM **AdPym-CHO**, 1.5 mM **AdPym-OH** (*ee* > 99%) and 40 mM *i*Pr_2Zn in toluene at r.t.

3.3 Identification of Reaction Intermediates

3.3.1 Intermediates of the TMSPyr autocatalytic system

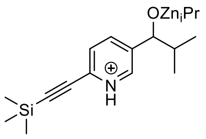

Chemical Formula: $C_{17}H_{28}NOSiZn^+$
Exact Mass: 354.1226

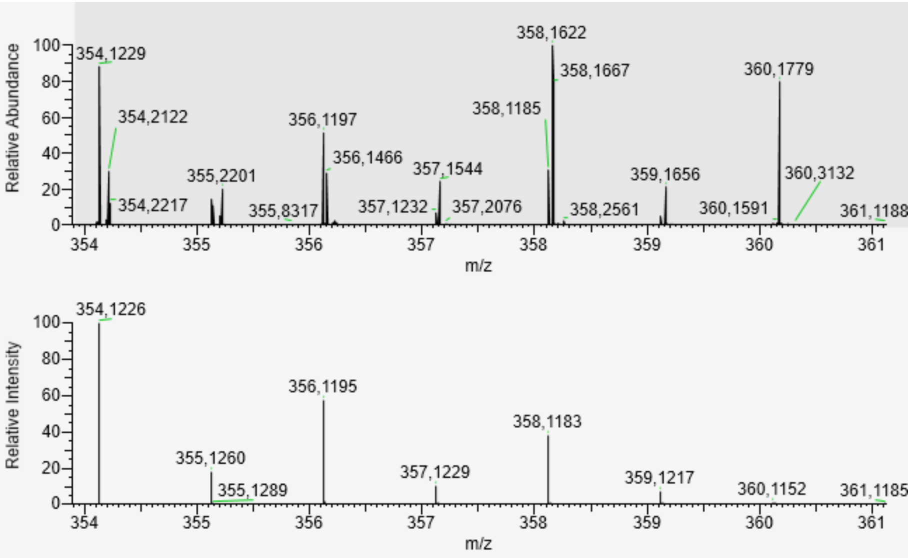

Supplementary Figure 4: Measured (top) and calculated (bottom) mass spectrum of intermediate **$I_{1,TMSPyr}$.**

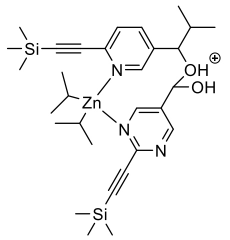

Chemical Formula: $C_{31}H_{49}N_2O_2Si_2Zn^+$
Exact Mass: 601,2619

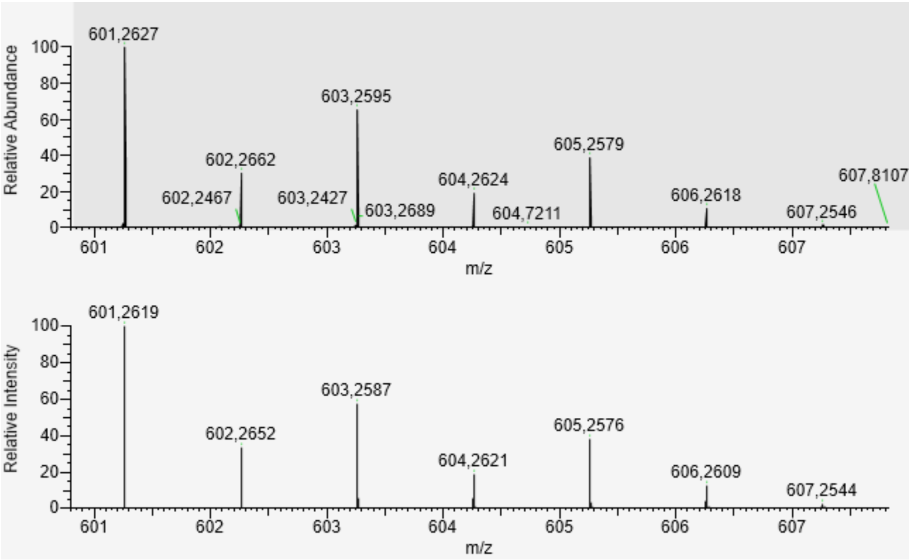

Supplementary Figure 5: Measured (top) and calculated (bottom) mass spectrum of an intermediate complex of $\mathbf{I}_{\mathbf{3,TMSPyr}}$ and diisopropyl zinc.

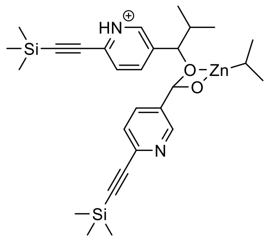

Chemical Formula: $C_{28}H_{41}N_2O_2Si_2Zn+$
Exact Mass: 557.1993

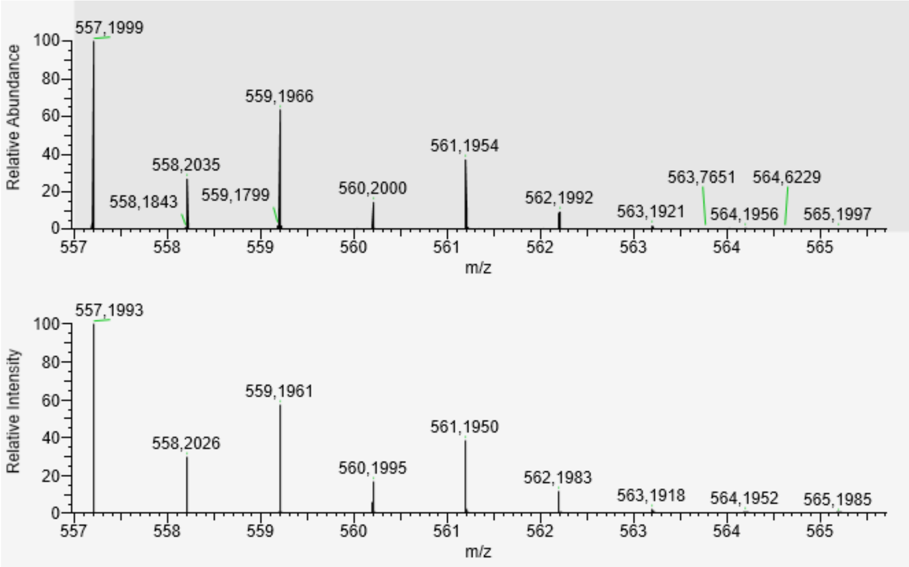

Supplementary Figure 6: Measured (top) and calculated (bottom) mass spectrum of intermediate **$I_{5,TMSPyr}$**.

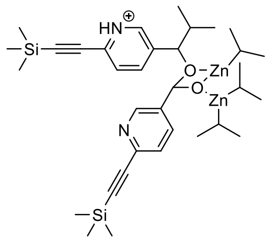

Chemical Formula: $C_{34}H_{55}N_2O_2Si_2Zn_2^+$
Exact Mass: 707,2380

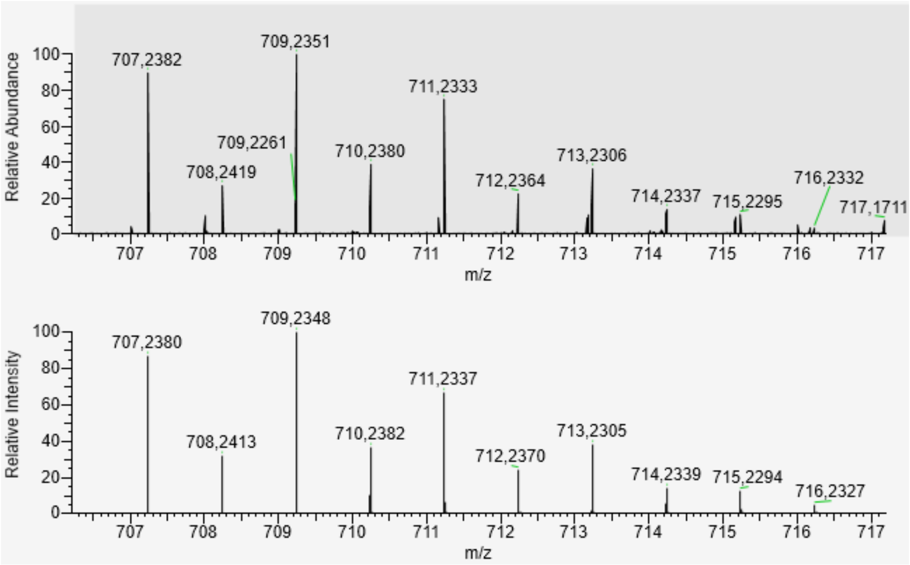

Supplementary Figure 7: Measured (top) and calculated (bottom) mass spectrum of intermediate **$I_{7,TMSPyr}$.**

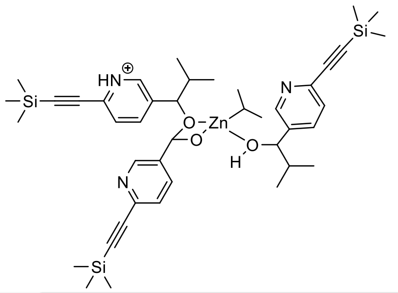

Chemical Formula: $C_{42}H_{62}N_3O_3Si_3Zn^+$
Exact Mass: 804,3385

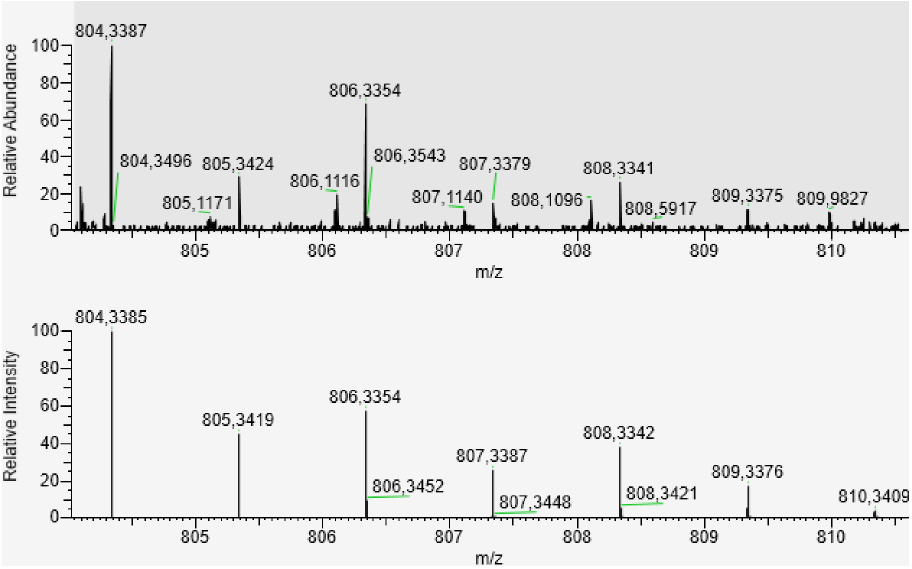

Supplementary Figure 8: Measured (top) and calculated (bottom) mass spectrum of intermediate **$I_{8,TMSPyr}$**.

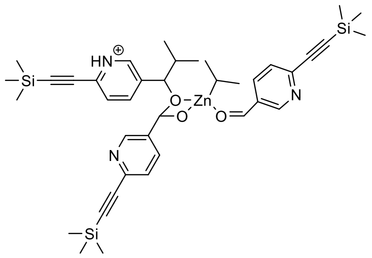

Chemical Formula: $C_{39}H_{54}N_3O_3Si_3Zn^+$
Exact Mass: 760.2759

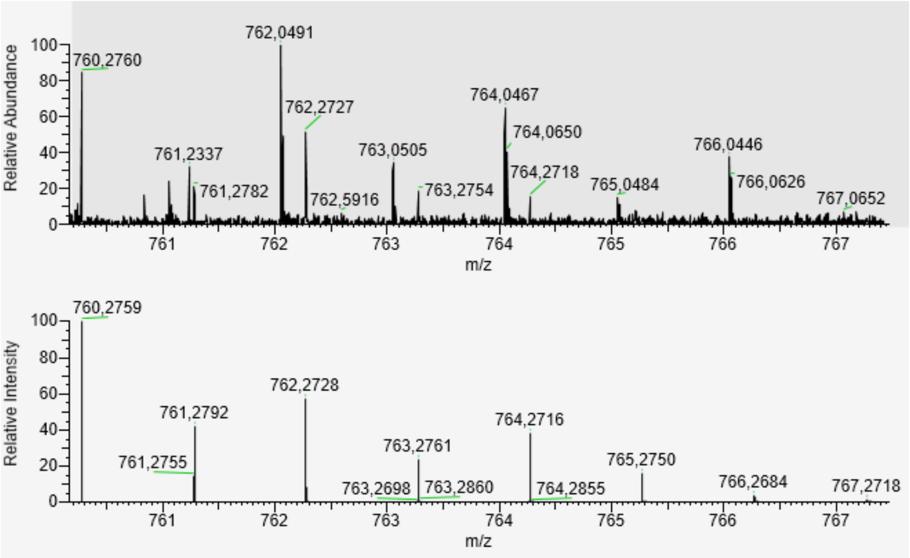

Supplementary Figure 9: Measured (top) and calculated (bottom) mass spectrum of intermediate $\mathbf{I_{6,TMSPyr}}$.

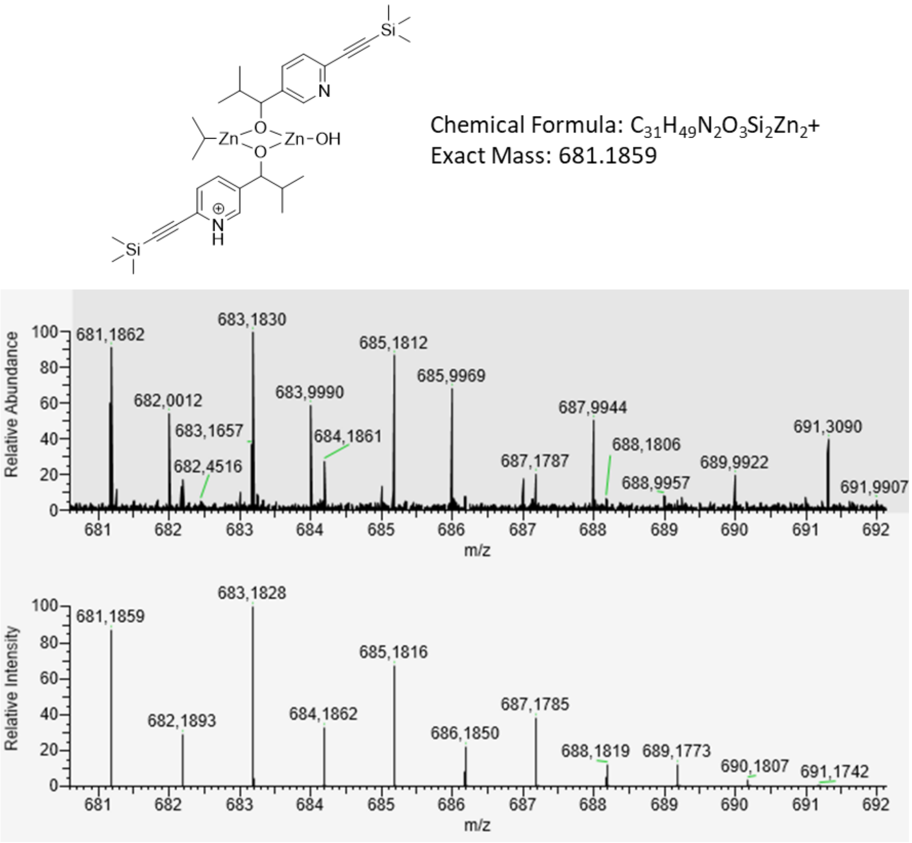

Supplementary Figure 10: Measured (top) and calculated (bottom) mass spectrum of intermediate **$I_{2,TMSPyr}$** (hydroxylated).

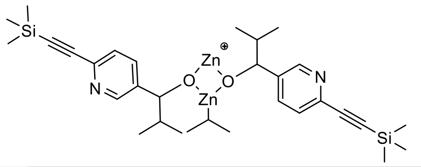

Chemical Formula: $C_{31}H_{47}N_2O_2Si_2Zn_2$+
Exact Mass: 663.1754

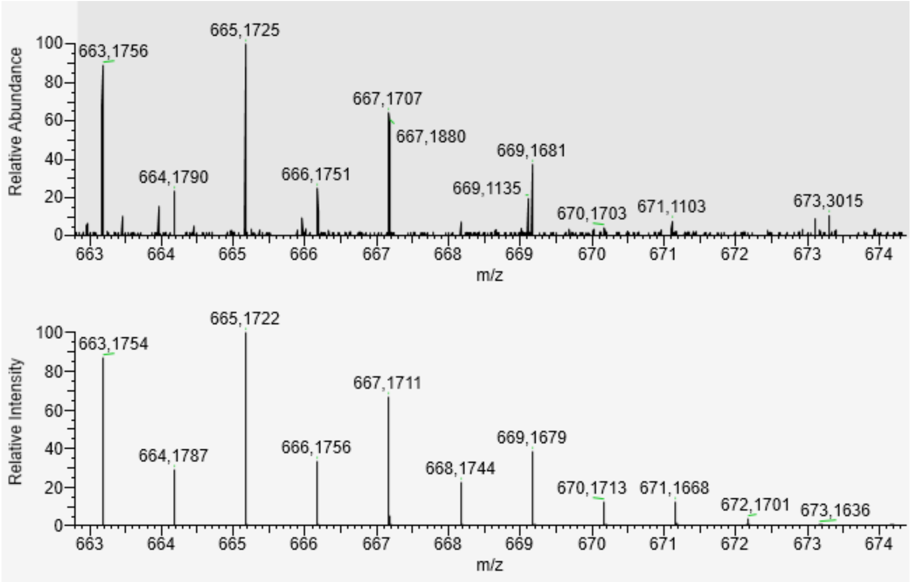

Supplementary Figure 11: Measured (top) and calculated (bottom) mass spectrum of intermediate $\mathbf{I_{2,TMSPyr}}$ (cleaved isopropyl group).

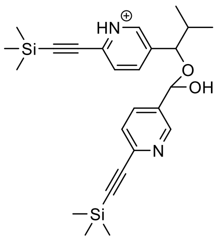

Chemical Formula: $C_{25}H_{35}N_2O_2Si_2^+$
Exact Mass: 451.2232

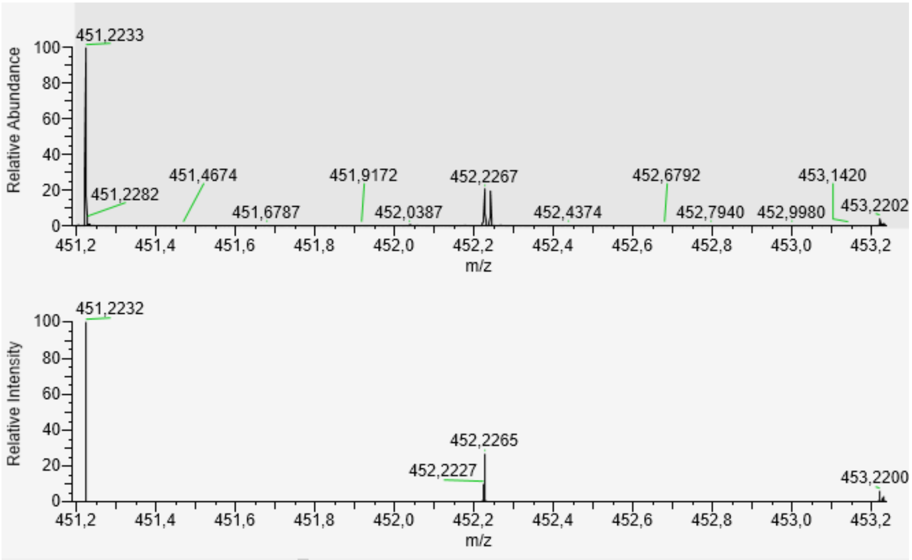

Supplementary Figure 12: Measured (top) and calculated (bottom) mass spectrum of intermediate **$I_{3,TMSPyr}$**.

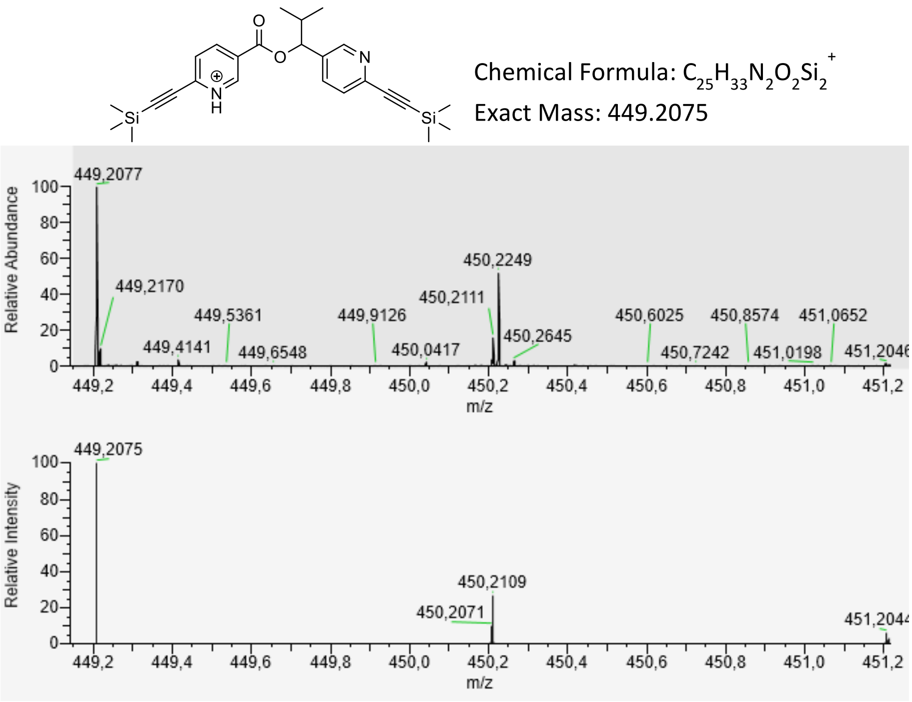

Supplementary Figure 13: Measured (top) and calculated (bottom) mass spectrum of intermediate **I$_{4,TMSPyr}$**.

3.3.2 Intermediates of the AdPyr autocatalytic system

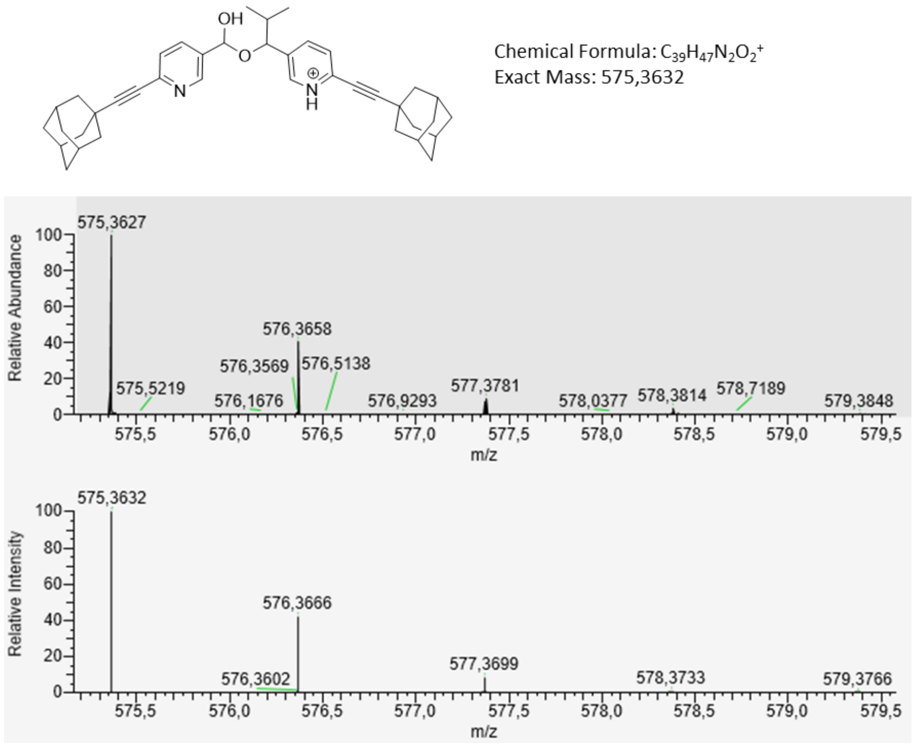

Supplementary Figure 14: Measured (top) and calculated (bottom) mass spectrum of intermediate $\mathbf{I}_{\mathbf{3,AdPyr}}$.

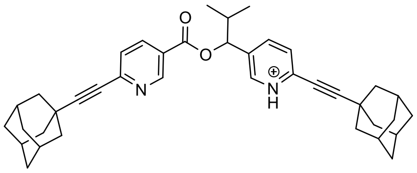

Chemical Formula: $C_{39}H_{45}N_2O_2^+$
Exact Mass: 573.3476

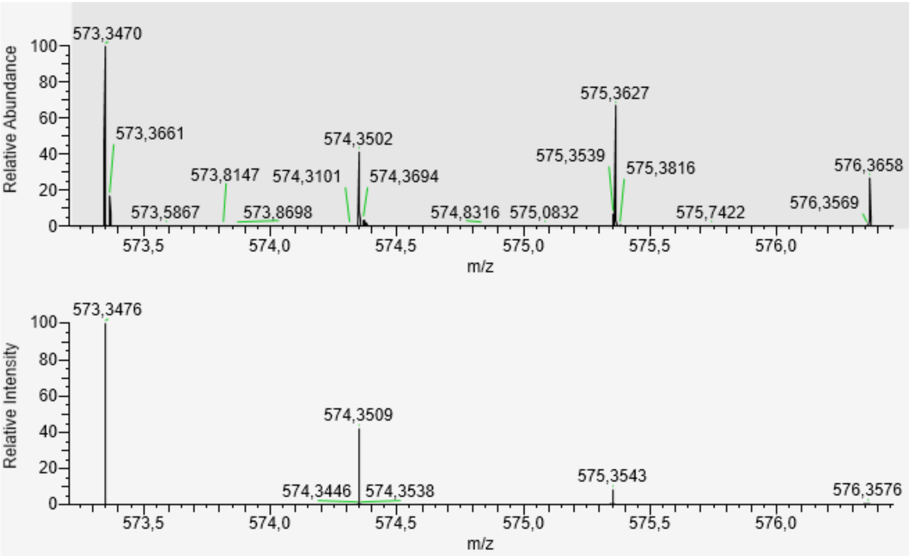

Supplementary Figure 15: Measured (top) and calculated (bottom) mass spectrum of intermediate **I4, AdPyr**.

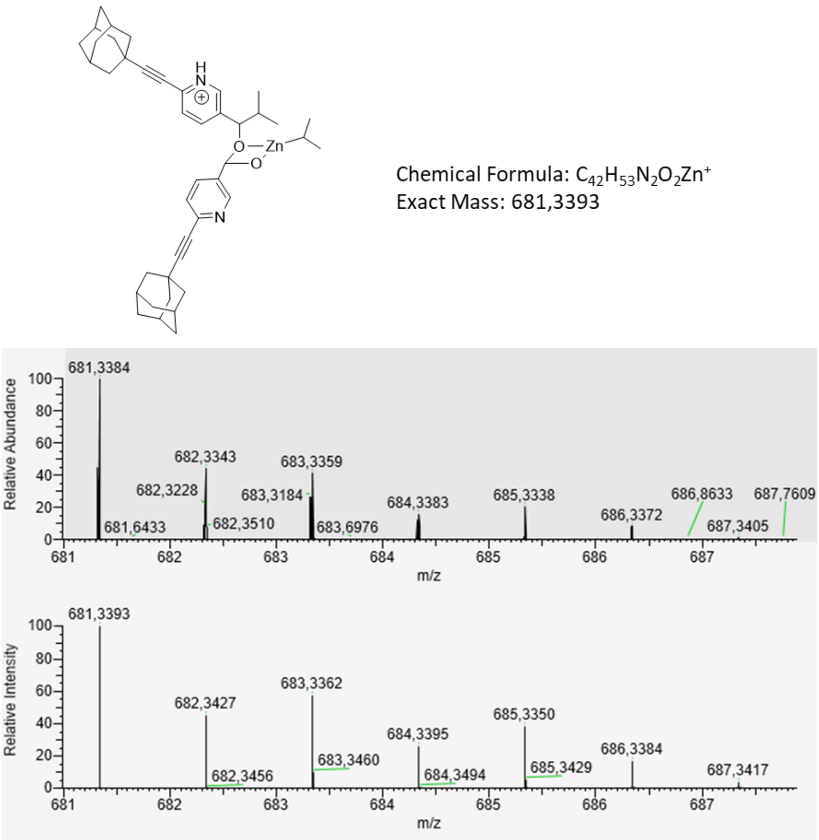

Supplementary Figure 16: Measured (top) and calculated (bottom) mass spectrum of intermediate $\mathbf{I_{5,AdPyr}}$.

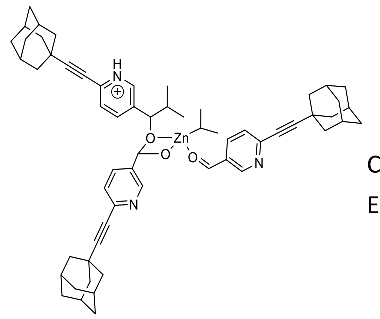

Chemical Formula: $C_{60}H_{72}N_3O_3Zn^+$
Exact Mass: 946.4860

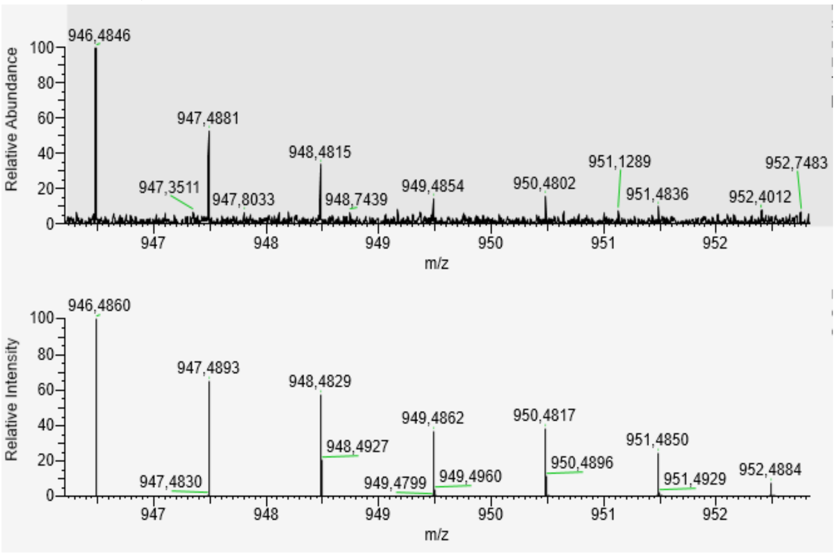

Supplementary Figure 17: Measured (top) and calculated (bottom) mass spectrum of intermediate $\mathbf{I_{6,AdPyr}}$.

3.3.3 Intermediates of the TMSPym autocatalytic system

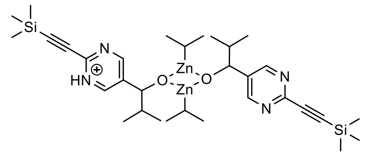

Chemical Formula: $C_{32}H_{53}N_4O_2Si_2Zn_2^+$
Exact Mass: 709.2284

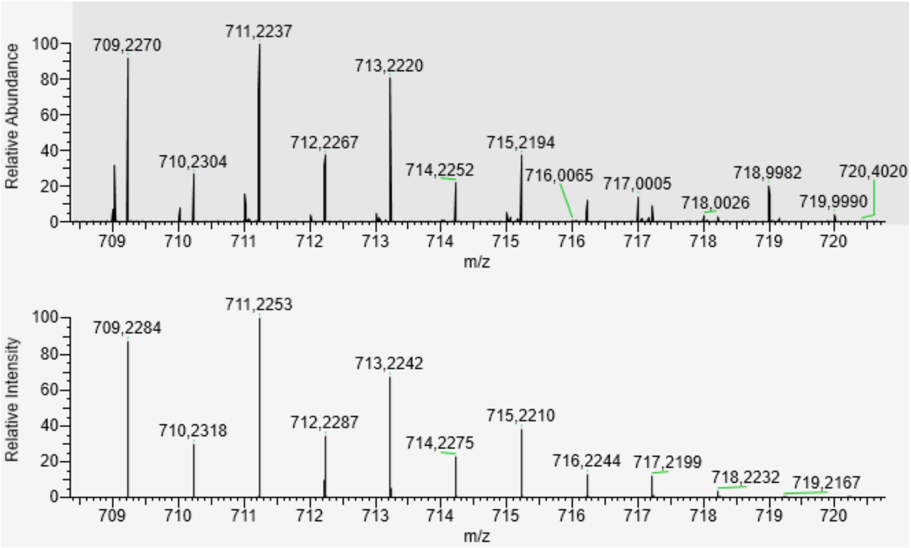

Supplementary Figure 18: Measured (top) and calculated (bottom) mass spectrum of intermediate $\mathbf{I_{2,TMSPym}}$.

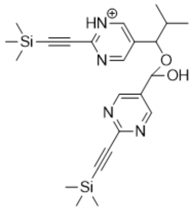

Chemical Formula: $C_{23}H_{33}N_4O_2Si_2^+$
Exact Mass: 453.2137

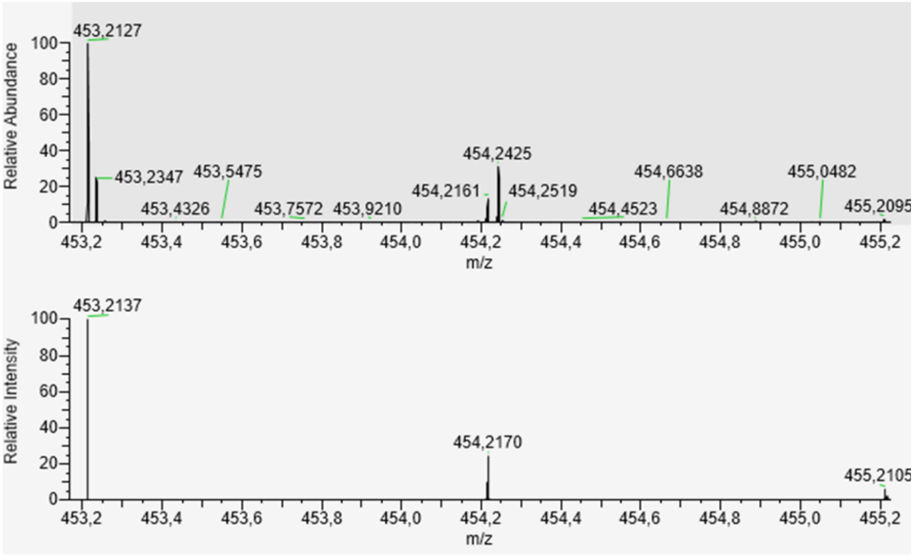

Supplementary Figure 19: Measured (top) and calculated (bottom) mass spectrum of intermediate **$I_{3,TMSPym}$**.

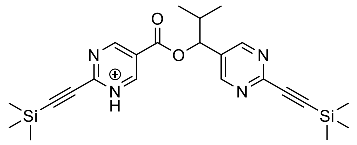

Chemical Formula: $C_{23}H_{31}N_4O_2Si_2^+$
Exact Mass: 451.1980

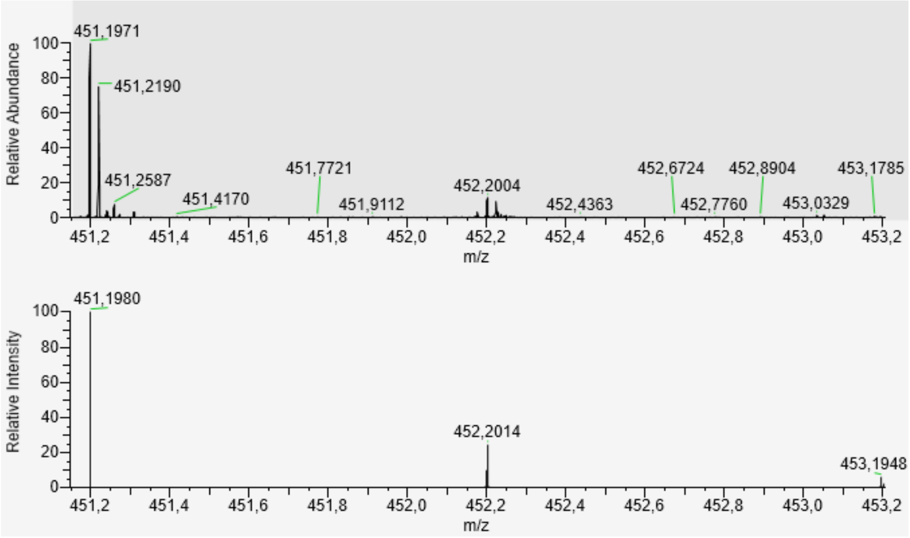

Supplementary Figure 20: Measured (top) and calculated (bottom) mass spectrum of intermediate **$I_{4,TMSPym}$**.

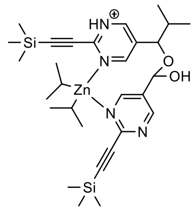

Chemical Formula: $C_{29}H_{47}N_4O_2Si_2Zn^+$
Exact Mass: 603.2524

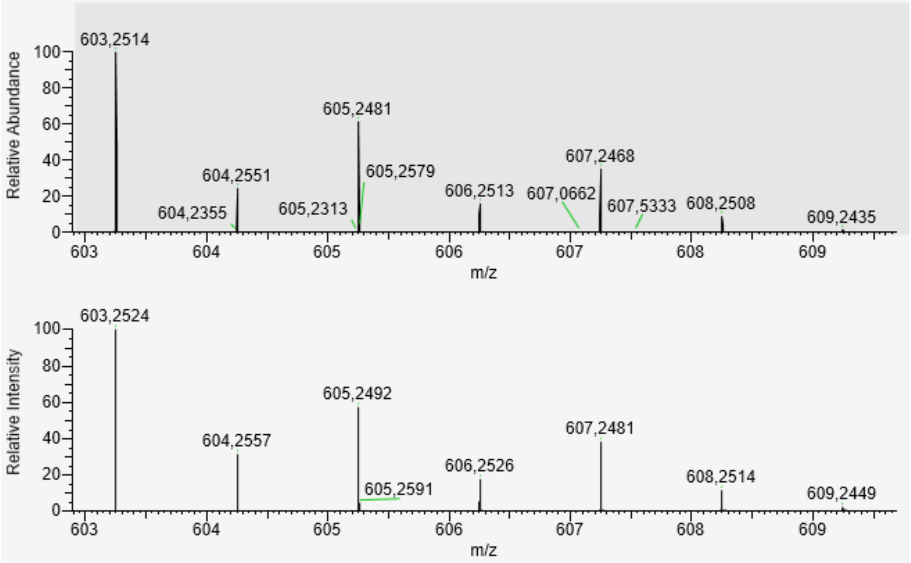

Supplementary Figure 21: Measured (top) and calculated (bottom) mass spectrum of intermediate complex of $\mathbf{I}_{\mathbf{3,TMSPym}}$ and diisopropyl zinc.

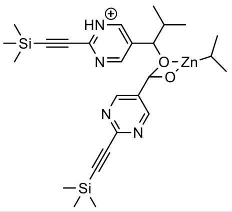

Chemical Formula: $C_{26}H_{39}N_4O_2Si_2Zn^+$
Exact Mass: 559.1898

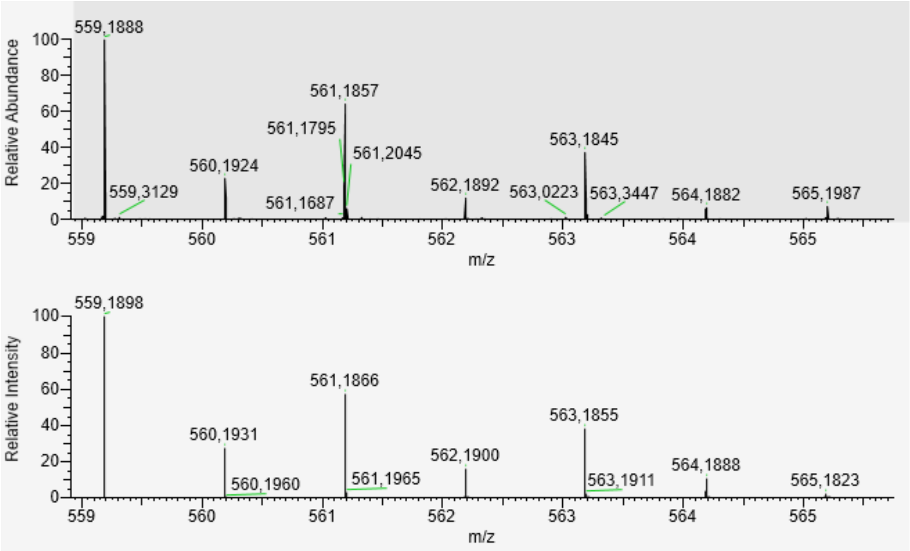

Supplementary Figure 22: Measured (top) and calculated (bottom) mass spectrum of intermediate $\mathbf{I_{5,TMSPym}}$.

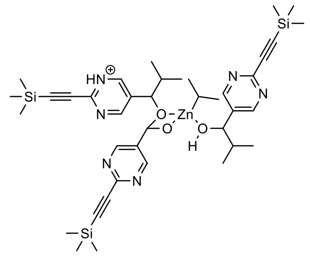

Chemical Formula: $C_{39}H_{59}N_6O_3Si_3Zn^+$
Exact Mass: 807.3242

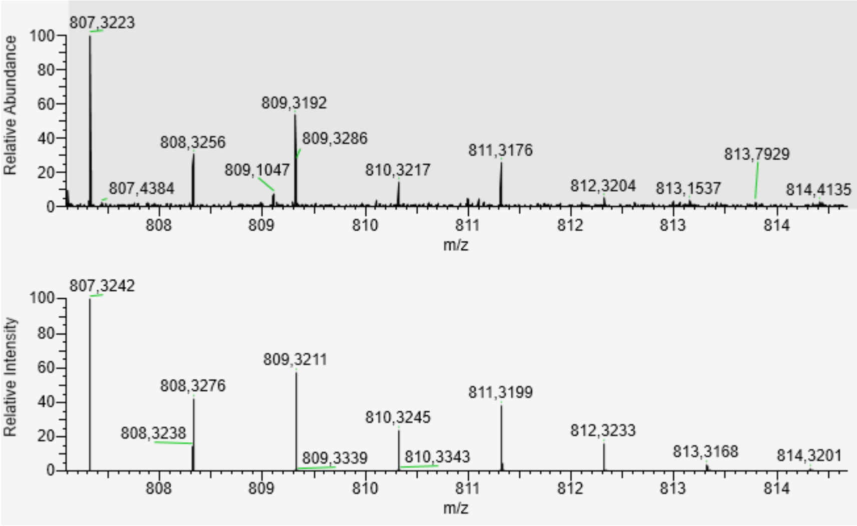

Supplementary Figure 23: Measured (top) and calculated (bottom) mass spectrum of intermediate **$I_{8,TMSPym}$**.

3.3.4 Intermediates of the AdPym autocatalytic system

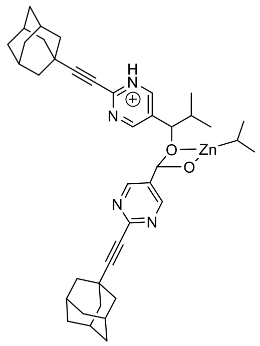

Chemical Formula: $C_{40}H_{51}N_4O_2Zn^+$
Exact Mass: 683.3298

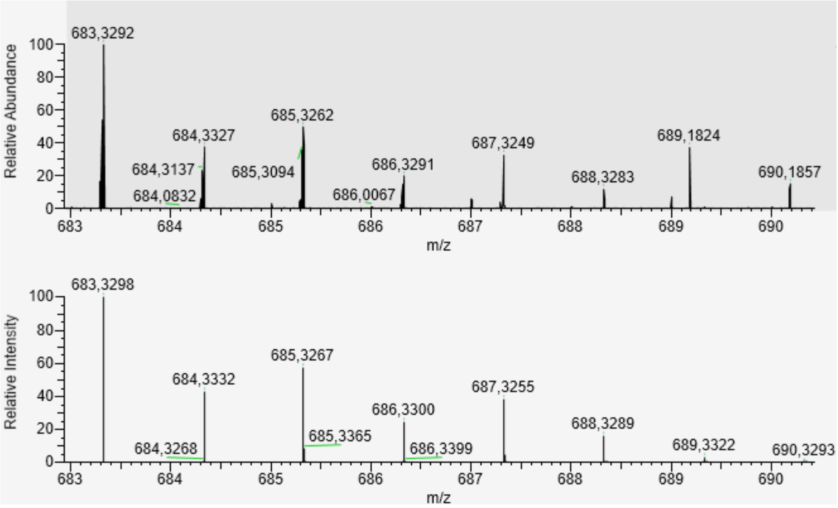

Supplementary Figure 24: Measured (top) and calculated (bottom) mass spectrum of intermediate $\mathbf{I_{5,AdPym}}$.

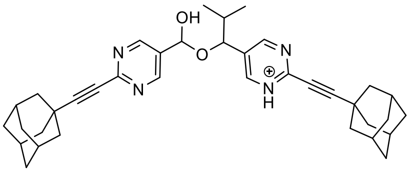

Chemical Formula: $C_{37}H_{45}N_4O_2^+$
Exact Mass: 577.3537

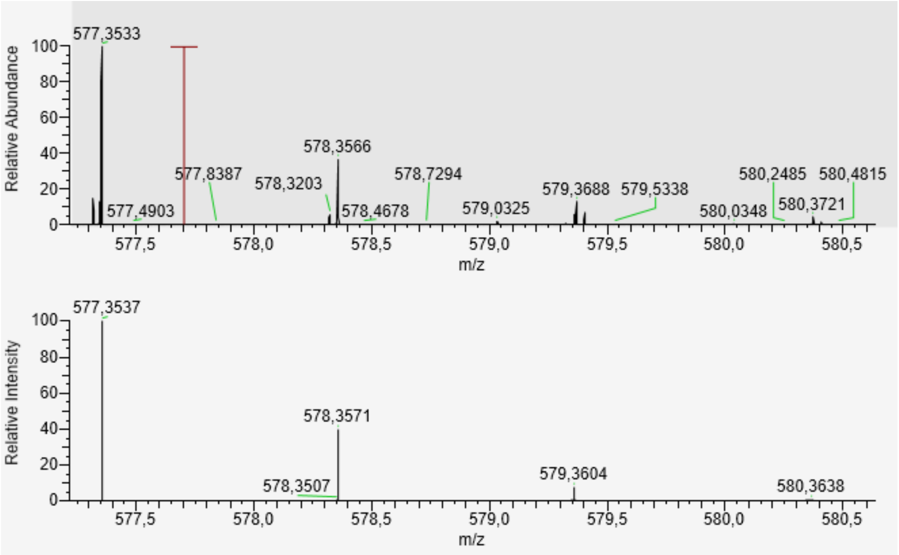

Supplementary Figure 25: Measured (top) and calculated (bottom) mass spectrum of intermediate $\mathbf{I_{5,AdPym}}$.

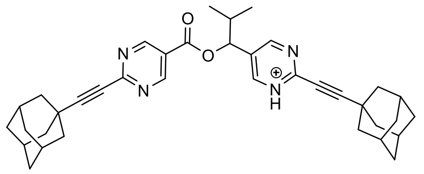

Chemical Formula: $C_{37}H_{43}N_4O_2^{+}$
Exact Mass: 575.3381

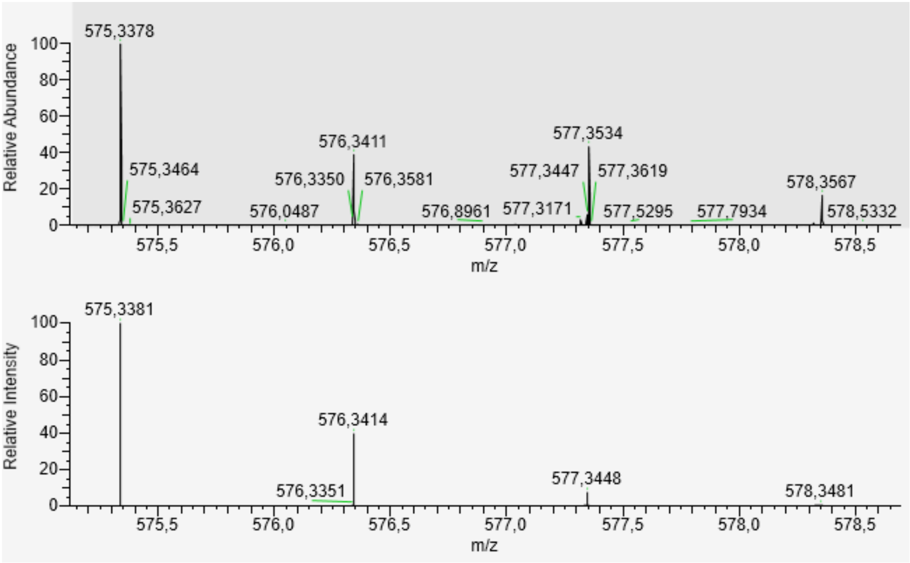

Supplementary Figure 26: Measured (top) and calculated (bottom) mass spectrum of intermediate **$I_{4,AdPym}$**.

4 Kinetic Investigations

4.1 General procedure for flow injection analysis (FIA) HPLC measurements

All kinetic measurements were carried out in new glass HPLC vials (Duran) which were dried in a 500 mL *Schlenk* flask prior to use and stored under Argon atmosphere. Stock solutions were prepared to mix them in a HPLC vial under argon atmosphere. All kinetic measurements were performed at room temperature. To ensure reproducibility, the measurements for one concentration were carried out three times and the respective mean value was used for further calculation.

An HPLC vial (1.5 mL) was filled with toluene stock solutions of aldehyde (0.86 mL) and alcohol (0.10 mL) under argon atmosphere. The vial was capped. Immediately after the addition of the diisopropylzinc stock solution (40 µL, 40 mM) the reaction mixture (total volume 1 mL) was vortexed and the FIA-HPLC measurement was started. A solvent mixture of *n*-hexane/THF was chosen as eluent to obtain separate the substrates and reaction products and to avoid hemiacetal formation of the aldehyde with isopropanol.

For the kinetic analysis of the pyridine system **TMSPyr**, a Chiralpak® ID column (250 mm, i.D. 4.6 mm, particle size: 5 µm) from *Chiral Technologies* was used as stationary phase. A *n*-hexane/THF mixture = 80/20 was used as eluent at a 1.2 ml/min flow rate. The injection of the reaction mixture into the column was performed in time intervals of 2.3 min.

For the kinetic analysis of the pyridine system **AdPyr**, a Chiralpak® ID column (250 mm, i.D. 4.6 mm, particle size: 5 µm) from *Chiral Technologies* was used as stationary phase. A *n*-hexane/THF mixture = 75/25 was used as eluent at a 1.0 ml/min flow rate. The injection of the reaction mixture into the column was performed in time intervals of 4.3 min.

For the kinetic analysis of the pyrimidine system **TMSPym**, a Chiralpak® IB column (250 mm, i.D. 4.6 mm, particle size: 5 µm) from *Chiral Technologies* was used as stationary phase. A *n*-hexane/THF mixture = 70/30 was used as eluent at a 1.2 ml/min flow rate. The injection of the reaction mixture into the column was performed in time intervals of 2.2 min.

For the kinetic analysis of the pyrimidine system **AdPym**, a Chiralpak® IC column (250 mm, i.D. 4.6 mm, particle size: 5 µm) from *Chiral Technologies* was used as stationary phase. A *n*-hexane/THF mixture = 75/25 was used as eluent at a 1.0 ml/min flow rate. The injection of the reaction mixture into the column was performed in time intervals of 3.6 min.

4.2 TMSPyr-CHO/TMSPyr-OH System

4.2.1 Calibration plots for quantitative analysis of kinetic measurements

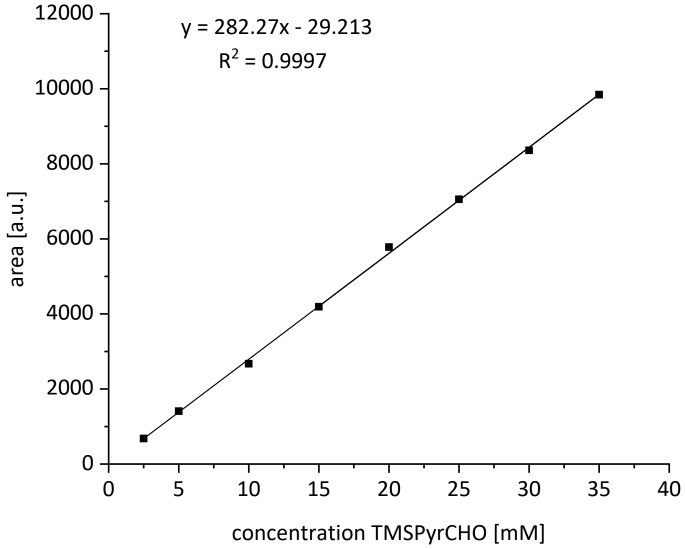

Supplementary Figure 27: Calibration plot on HPLC for **TMSPyr-CHO** on a Chiralpak ID® column (250 mm, i.D. 4.6 mm, particle size: 5 µm), *n*-hexane/THF = 80/20, 1.2 mL/min, λ = 250 nm, r.t.

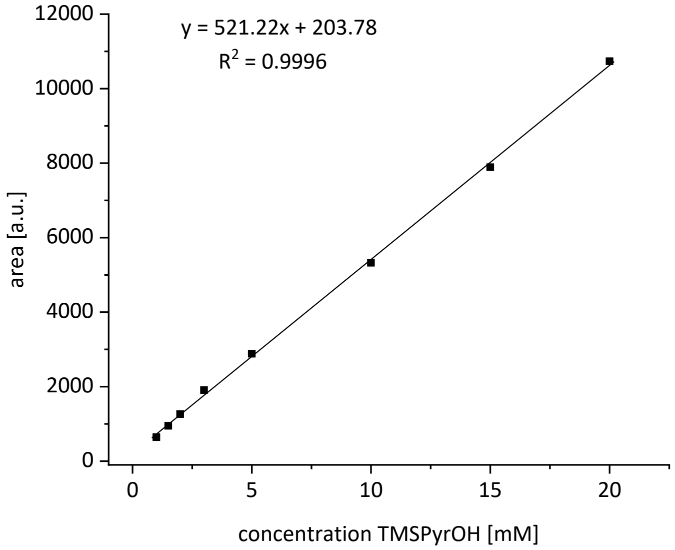

Supplementary Figure 28: Calibration plot on HPLC for **TMSPyr-OH** (*ee* > 99%) on a Chiralpak ID® column (250 mm, i.D. 4.6 mm, particle size: 5 µm), *n*-hexane/THF = 80/20, 1.2 mL/min, λ = 250 nm, r.t.

4.2.2 Variation of the TMSPyr-CHO concentration

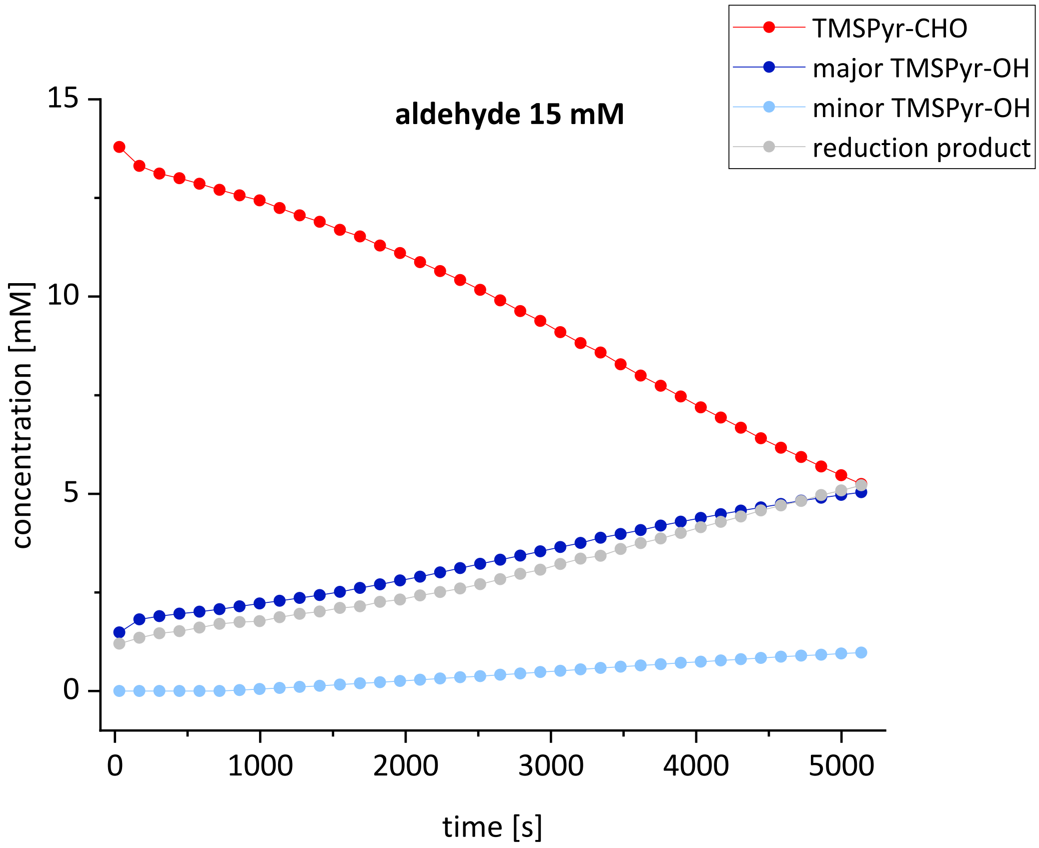

Supplementary Figure 29: Concentration-time profile of the Soai reaction in toluene (15 mM 6-((trimethylsilyl)ethynyl)nicotinaldehyde **TMSPyr-CHO**, 1.5 mM (1*R*)-2-methyl-(6-((trimethylsilyl)ethynyl)-pyridine-3-yl)propanol **TMSPyr-OH** (*ee* > 99.9%) and 40 mM *i*Pr_2Zn; r.t.

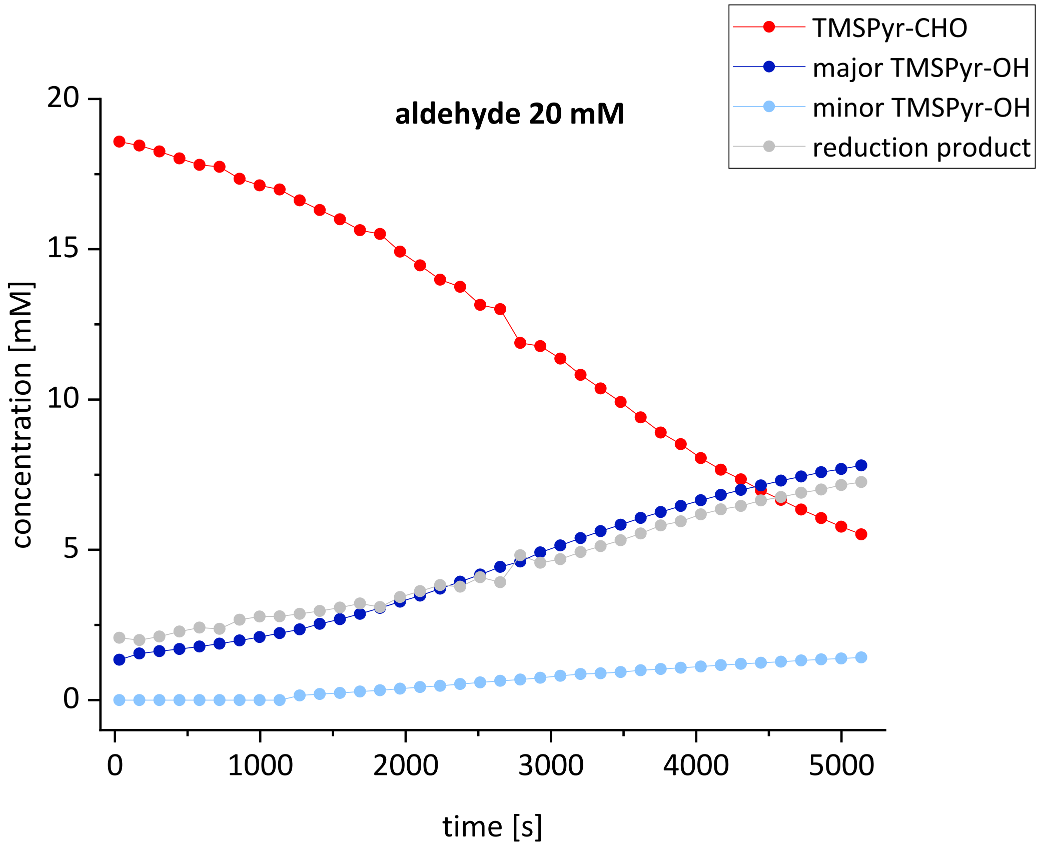

Supplementary Figure 30: Concentration-time profile of the Soai reaction in toluene (20 mM 6-((trimethylsilyl)ethynyl)nicotinaldehyde **TMSPyr-CHO**, 1.5 mM (1*R*)-2-methyl-(6-((trimethylsilyl)ethynyl)pyridine-3-yl)propanol **TMSPyr-OH** (*ee* > 99.9%) and 40 mM $i\text{Pr}_2\text{Zn}$; r.t.

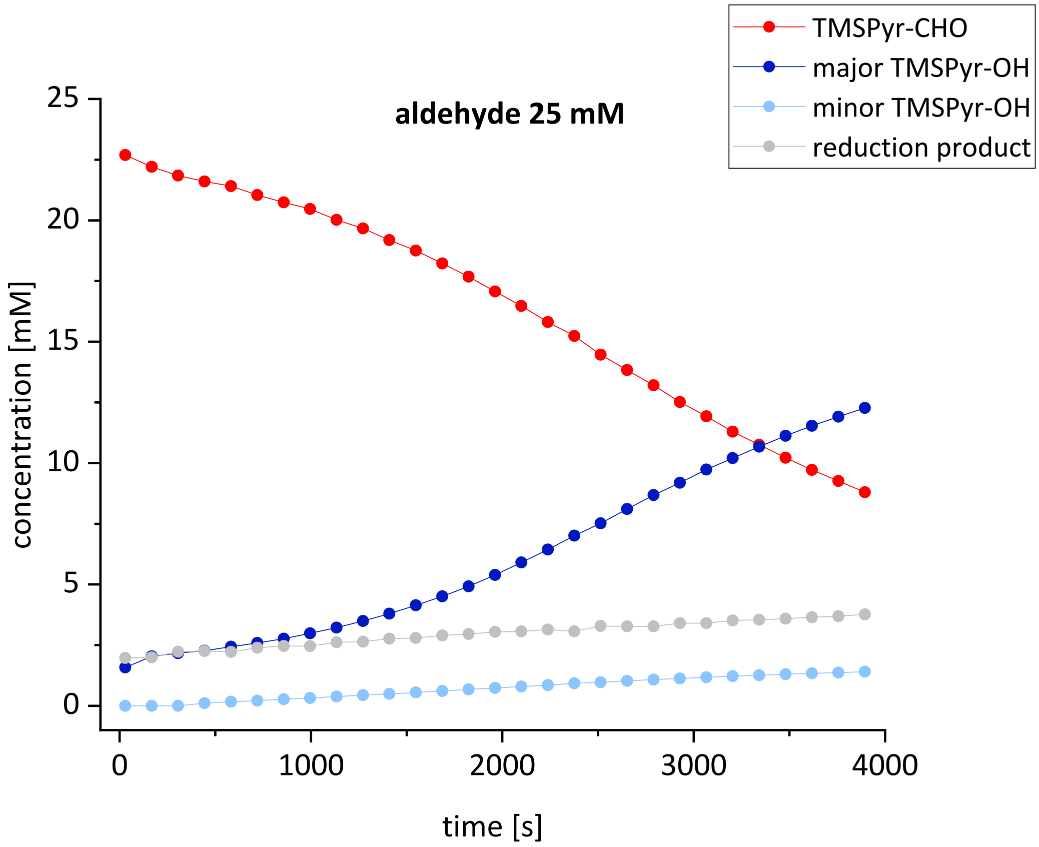

Supplementary Figure 31: Concentration-time profile of the Soai reaction in toluene (25 mM 6-((trimethylsilyl)ethynyl)nicotinaldehyde **TMSPyr-CHO**, 1.5 mM (1*R*)-2-methyl-(6-((trimethylsilyl)ethynyl)-pyridine-3-yl)propanol **TMSPyr-OH** (*ee* > 99.9%) and 40 mM *i*Pr_2Zn; r.t.

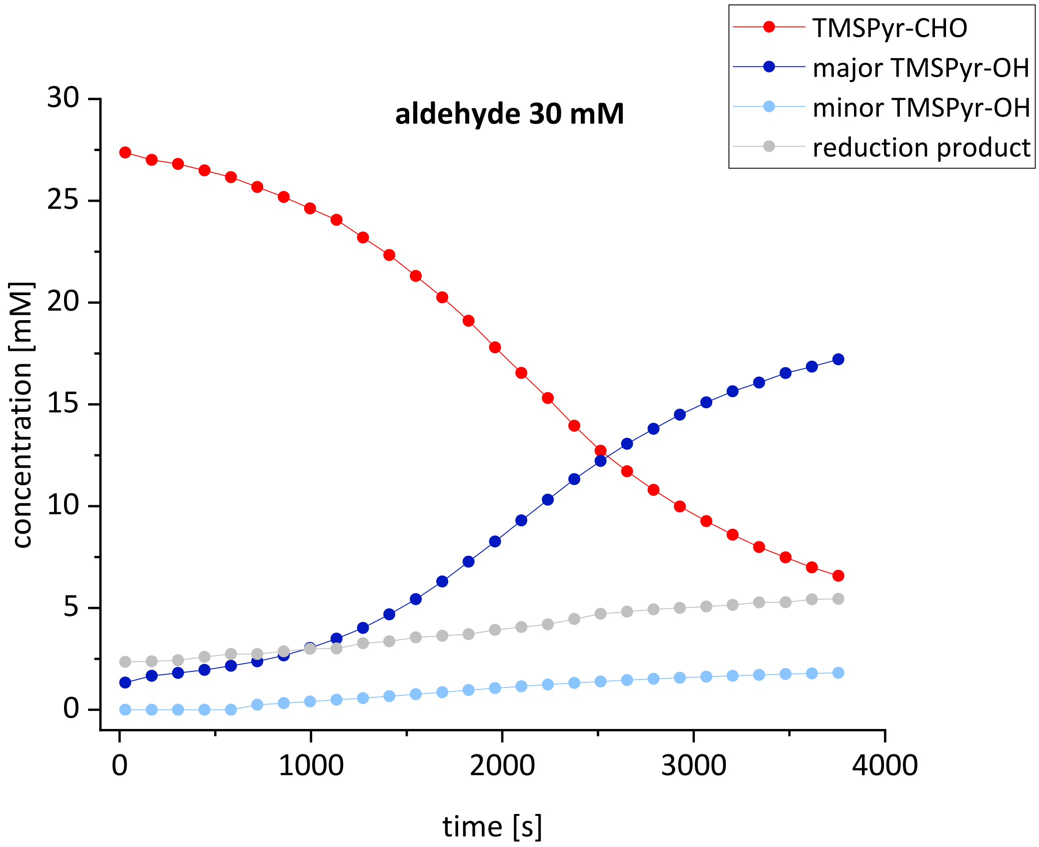

Supplementary Figure 32: Concentration-time profile of the Soai reaction in toluene (30 mM 6-((trimethylsilyl)ethynyl)nicotinaldehyde **TMSPyr-CHO**, 1.5 mM (1*R*)-2-methyl-(6-((trimethylsilyl)ethynyl)-pyridine-3-yl)propanol **TMSPyr-OH** (*ee* > 99.9%) and 40 mM *i*Pr_2Zn; r.t.

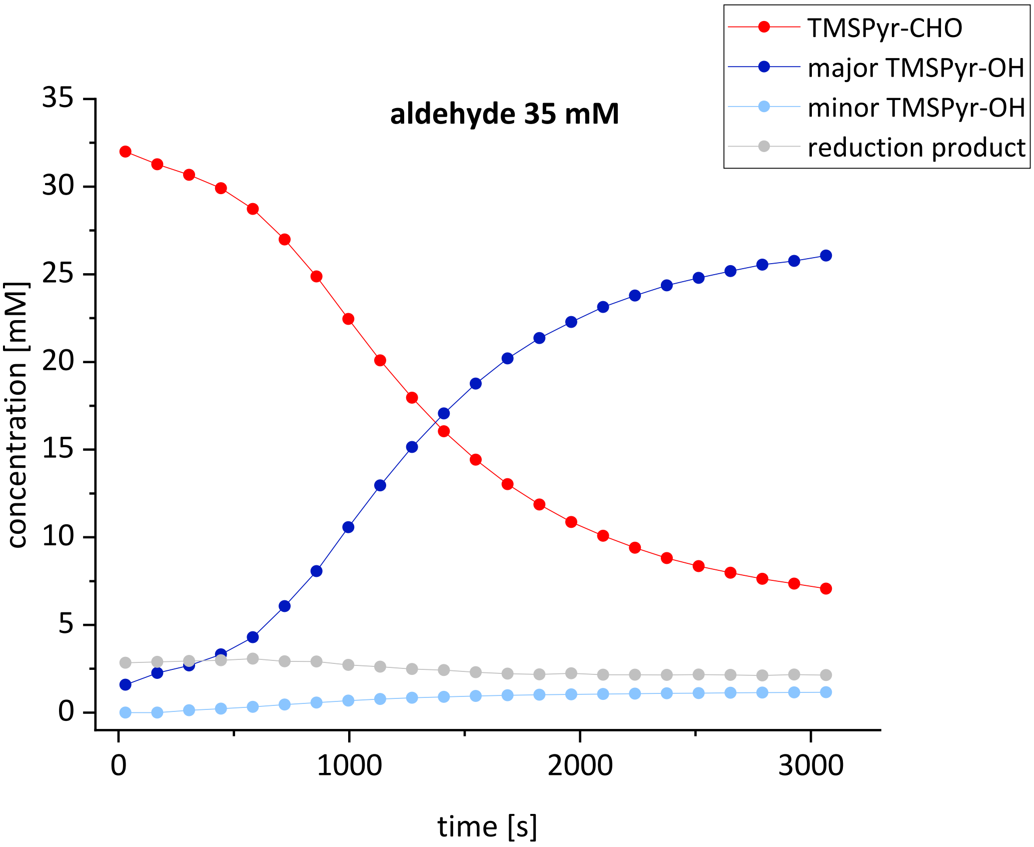

Supplementary Figure 33: Concentration-time profile of the Soai reaction in toluene (35 mM 6-((trimethylsilyl)-ethynyl)nicotinaldehyde **TMSPyr-CHO**, 1.5 mM (1*R*)-2-methyl-(6-((trimethylsilyl)ethynyl)-pyridine-3-yl)propanol **TMSPyr-OH** (*ee* > 99.9%) and 40 mM *i*Pr_2Zn; r.t.

4.2.3 Variation of the TMSPyr-OH concentration

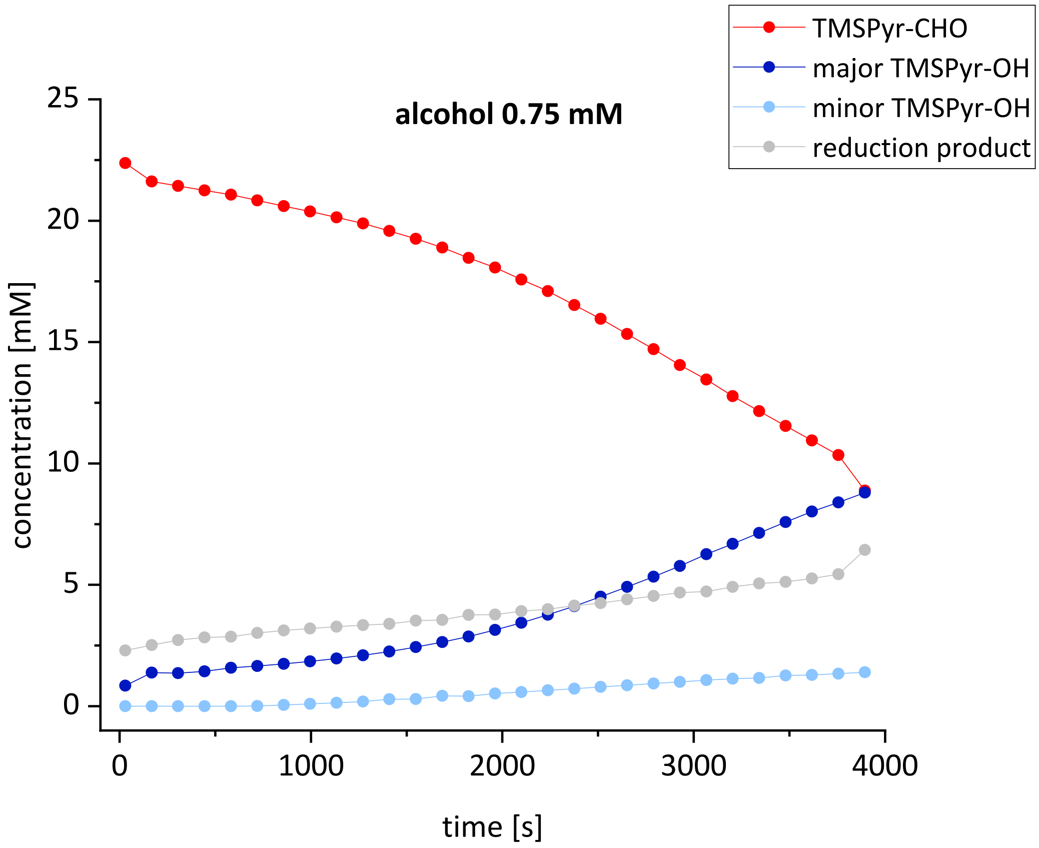

Supplementary Figure 34: Concentration-time profile of the Soai reaction in toluene (25 mM 6-((trimethylsilyl)-ethynyl)nicotinaldehyde **TMSPyr-CHO**, 0.75 mM (1*R*)-2-methyl-(6-((trimethylsilyl)ethynyl)pyridine-3-yl)propanol **TMSPyr-OH** (*ee* > 99.9%) and 40 mM *i*Pr_2Zn; r.t.

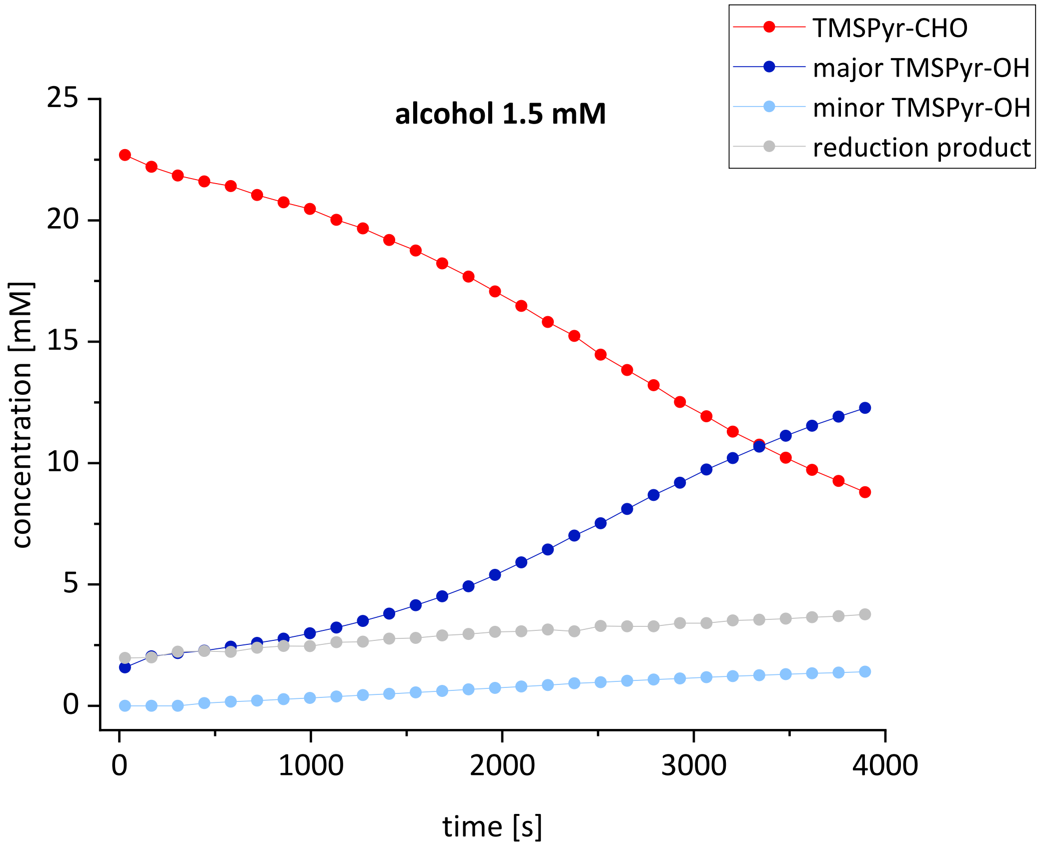

Supplementary Figure 35: Concentration-time profile of the Soai reaction in toluene (25 mM 6-((trimethylsilyl)ethynyl)nicotinaldehyde **TMSPyr-CHO**, 1.5 mM (1*R*)-2-methyl-(6-((trimethylsilyl)ethynyl)pyridine-3-yl)propanol **TMSPyr-OH** (*ee* > 99.9%) and 40 mM *i*Pr_2Zn; r.t.

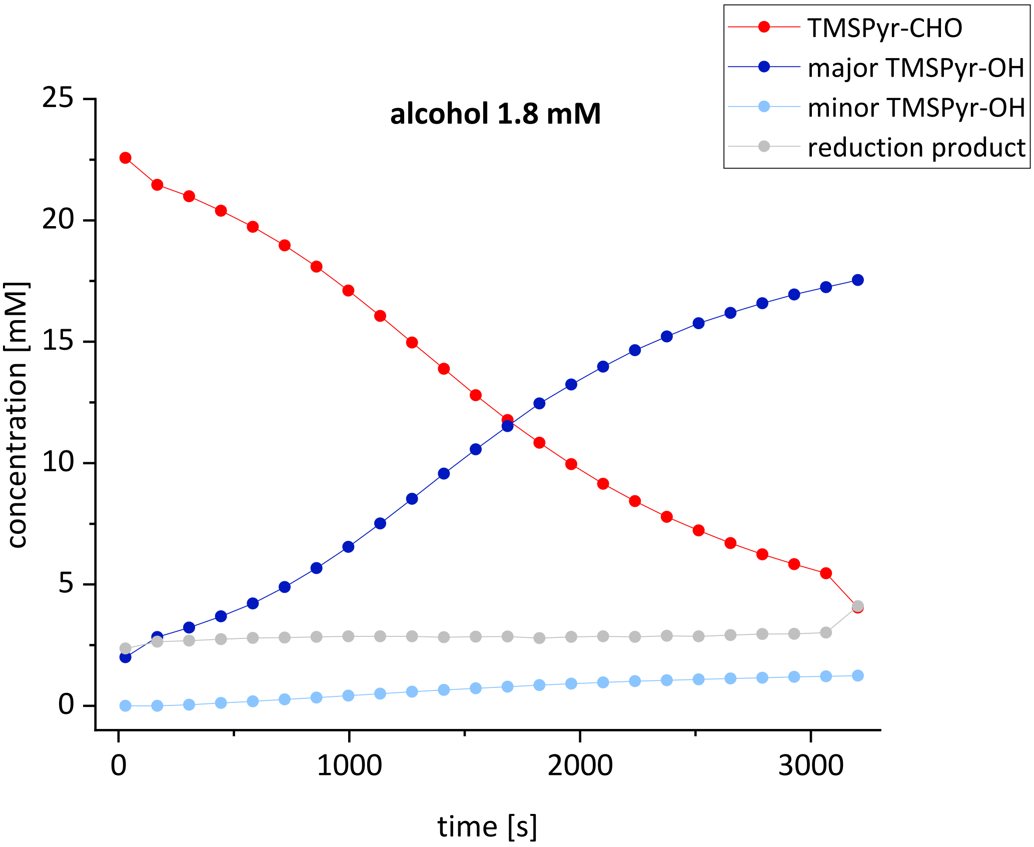

Supplementary Figure 36: Concentration-time profile of the Soai reaction in toluene (25 mM 6-((trimethylsilyl)ethynyl)nicotinaldehyde **TMSPyr-CHO**, 1.8 mM (1*R*)-2-methyl-(6-((trimethylsilyl)ethynyl)pyridine-3-yl)propanol **TMSPyr-OH** (*ee* > 99.9%) and 40 mM $i\text{Pr}_2\text{Zn}$; r.t.

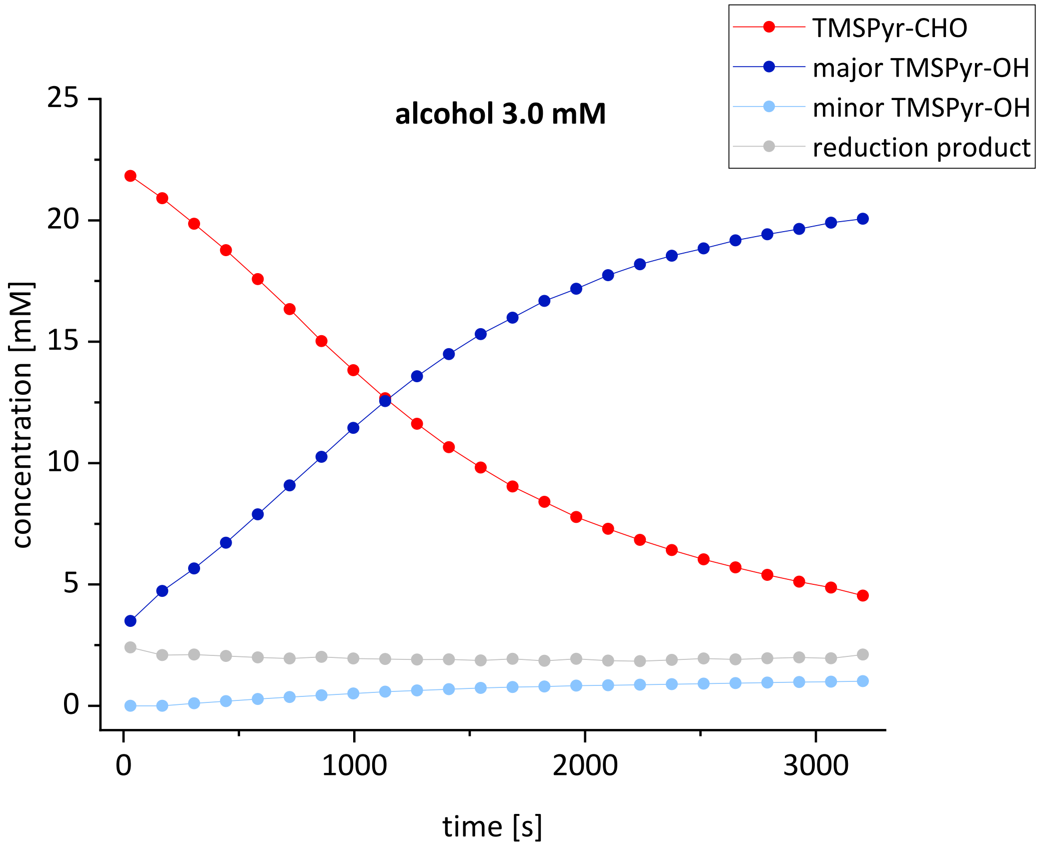

Supplementary Figure 37: Concentration-time profile of the Soai reaction in toluene (25 mM 6-((trimethylsilyl)-ethynyl)nicotinaldehyde **TMSPyr-CHO**, 3.0 mM (1*R*)-2-methyl-(6-((trimethylsilyl)ethynyl)pyridine-3-yl)propanol **TMSPyr-OH** (*ee* > 99.9%) and 40 mM iPr_2Zn; r.t.

4.2.4 Determination of the Reaction Orders

a. Reaction Order for aldehyde TMSPyr-CHO

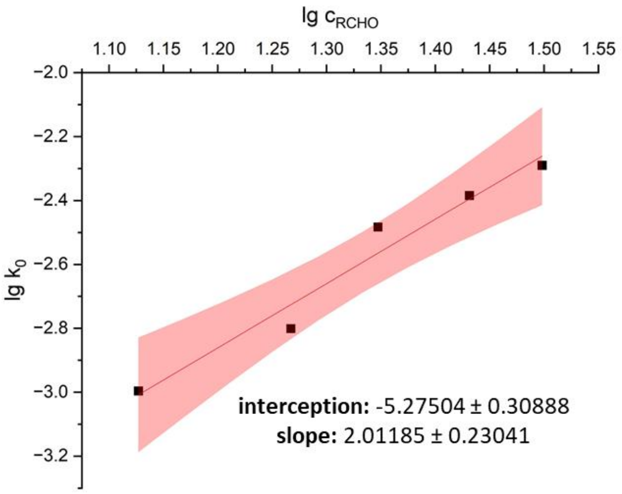

Supplementary Figure 38: Determination of the reaction order of **TMSPyr-CHO** by linear regression analysis of lg(k_0) vs. lg($c_{aldehyde}$).

b. Reaction Order for alcohol TMSPyr-OH

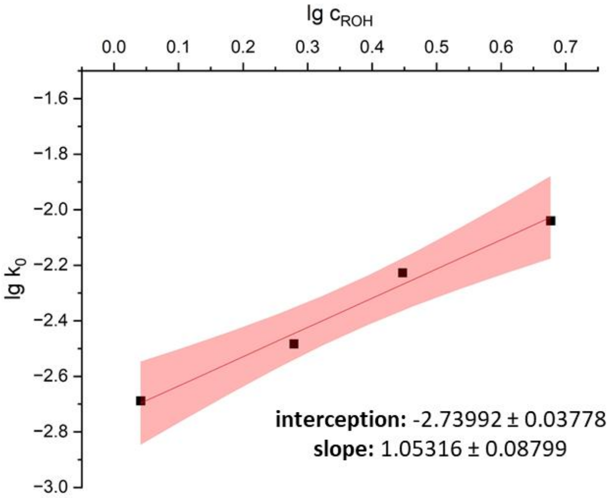

Supplementary Figure 39: Determination of the reaction order of **TMSPyr-OH** by linear regression analysis of lg(k_0) vs. lg($c_{alcohol}$).

4.3 AdPyr-CHO/AdPyr-OH System

4.3.1 Calibration plots for quantitative analysis of kinetic measurements

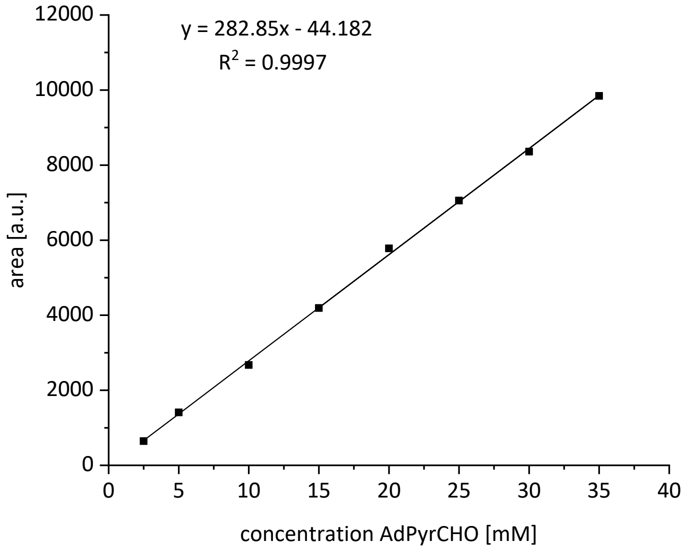

Supplementary Figure 40: Calibration plot on HPLC for **AdPyr-CHO** on a Chiralpak ID® column (250 mm, i.D. 4.6 mm, particle size: 5 µm), *n*-hexane/THF = 75/25, 1.0 mL/min, λ = 250 nm, r.t.

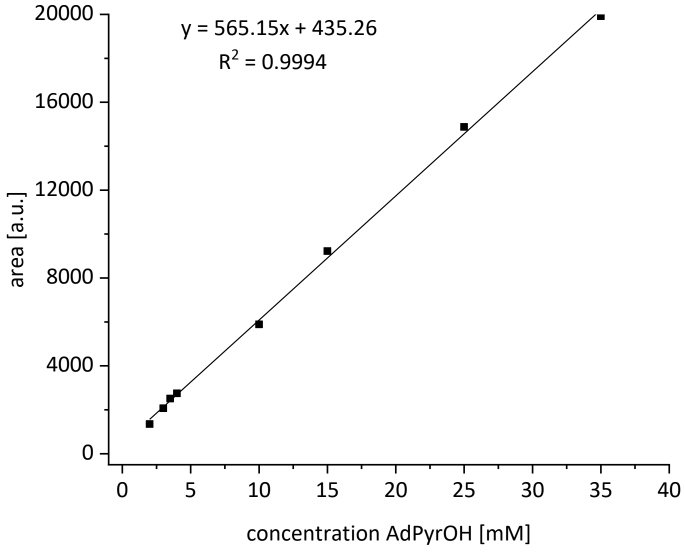

Supplementary Figure 41: Calibration plot on HPLC for **AdPyr-OH** on a Chiralpak ID® column (250 mm, i.D. 4.6 mm, particle size: 5 µm), *n*-hexane/THF = 75/25, 1.0 mL/min, λ = 250 nm, r.t.

4.3.2 Variation of the AdPyr-CHO concentration

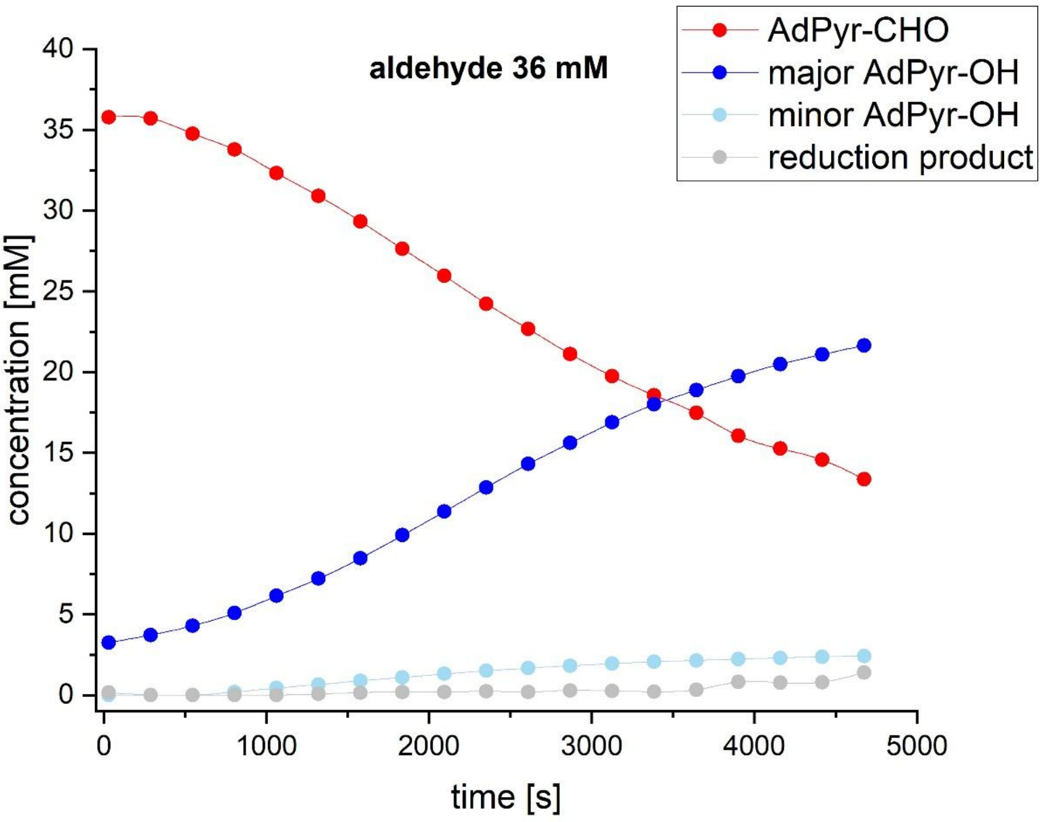

Supplementary Figure 42: Concentration-time profile of the Soai reaction in toluene (36 mM 6-((adamantan-1-yl)ethynyl)nicotinaldehyde **AdPyr-CHO**, 3.0 mM (1*R*)-1-(6-((adamantan-1-yl)ethynyl)pyridin-3-yl)-2-methylpropan-1-ol **AdPyr-OH** (*ee* > 99.9%) and 40 mM *i*Pr_2Zn; r.t.

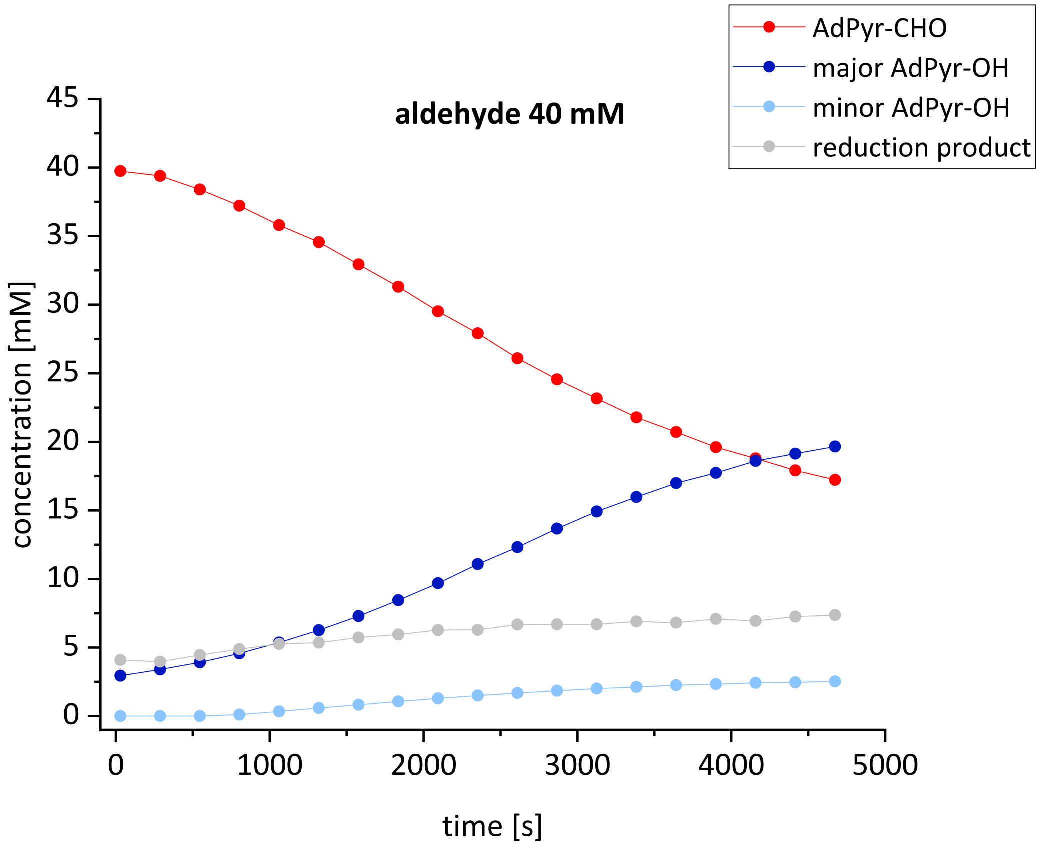

Supplementary Figure 43: Concentration-time profile of the Soai reaction in toluene (40 mM 6-((adamantan-1-yl)ethynyl)nicotinaldehyde **AdPyr-CHO**, 3.0 mM (1*R*)-1-(6-((adamantan-1-yl)ethynyl)pyridin-3-yl)-2-methylpropan-1-ol **AdPyr-OH** (*ee* > 99.9%) and 40 mM *i*Pr_2Zn; r.t.

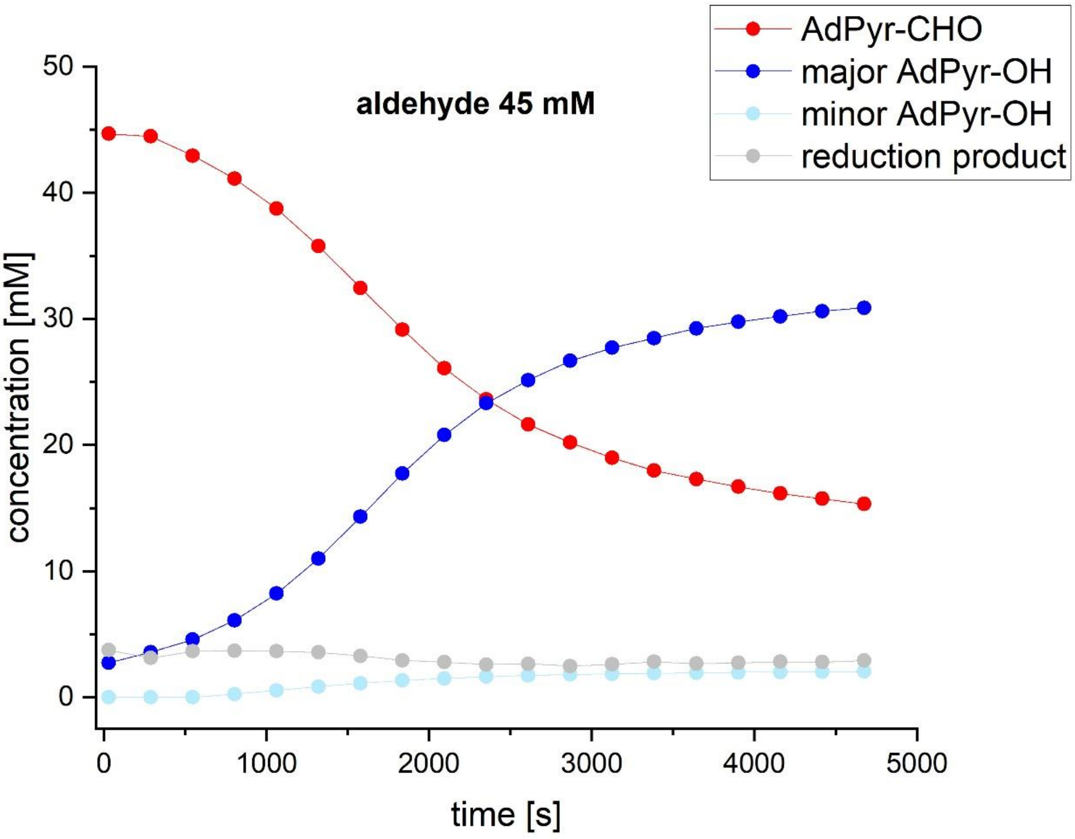

Supplementary Figure 44: Concentration-time profile of the Soai reaction in toluene (45 mM 6-((adamantan-1-yl)ethynyl)nicotinaldehyde **AdPyr-CHO**, 3.0 mM (1*R*)-1-(6-((adamantan-1-yl)ethynyl)pyridin-3-yl)-2-methylpropan-1-ol **AdPyr-OH** (*ee* > 99.9%) and 40 mM iPr_2Zn; r.t.

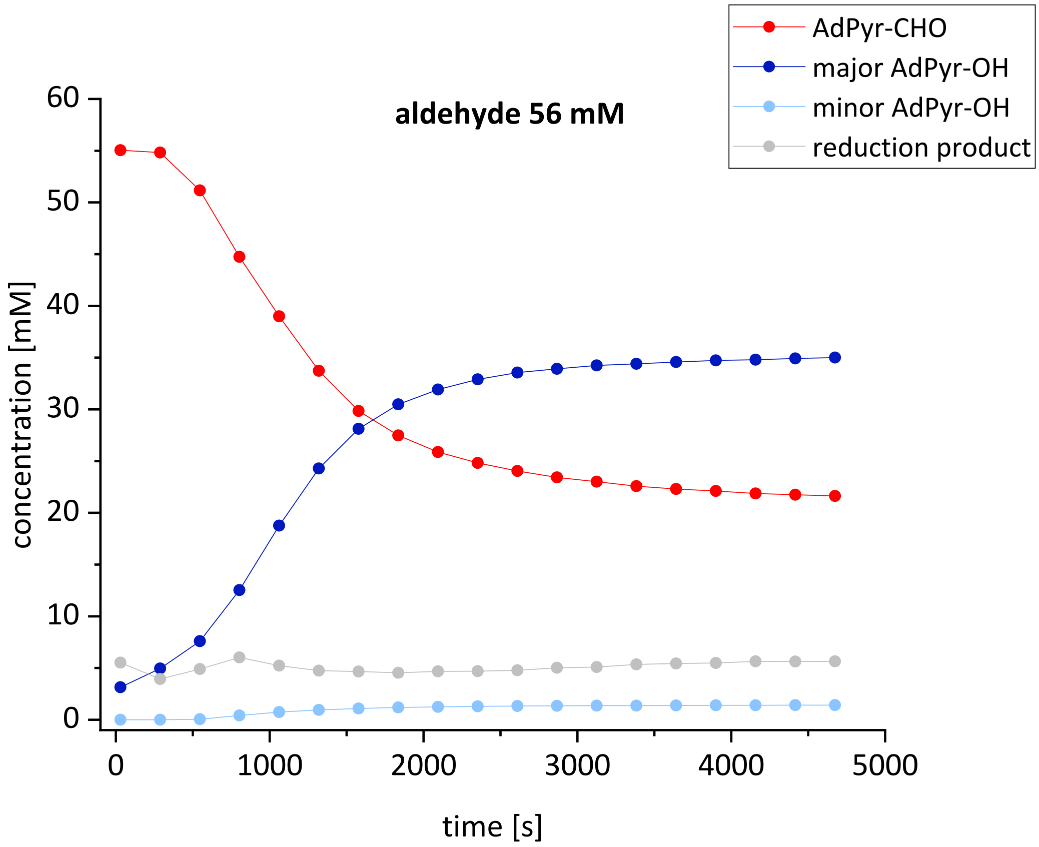

Supplementary Figure 45: Concentration-time profile of the Soai reaction in toluene (56 mM 6-((adamantan-1-yl)ethynyl)nicotinaldehyde **AdPyr-CHO**, 3.0 mM (1*R*)-1-(6-((adamantan-1-yl)ethynyl)pyridin-3-yl)-2-methylpropan-1-ol **AdPyr-OH** (*ee* > 99.9%) and 40 mM *i*Pr_2Zn; r.t.

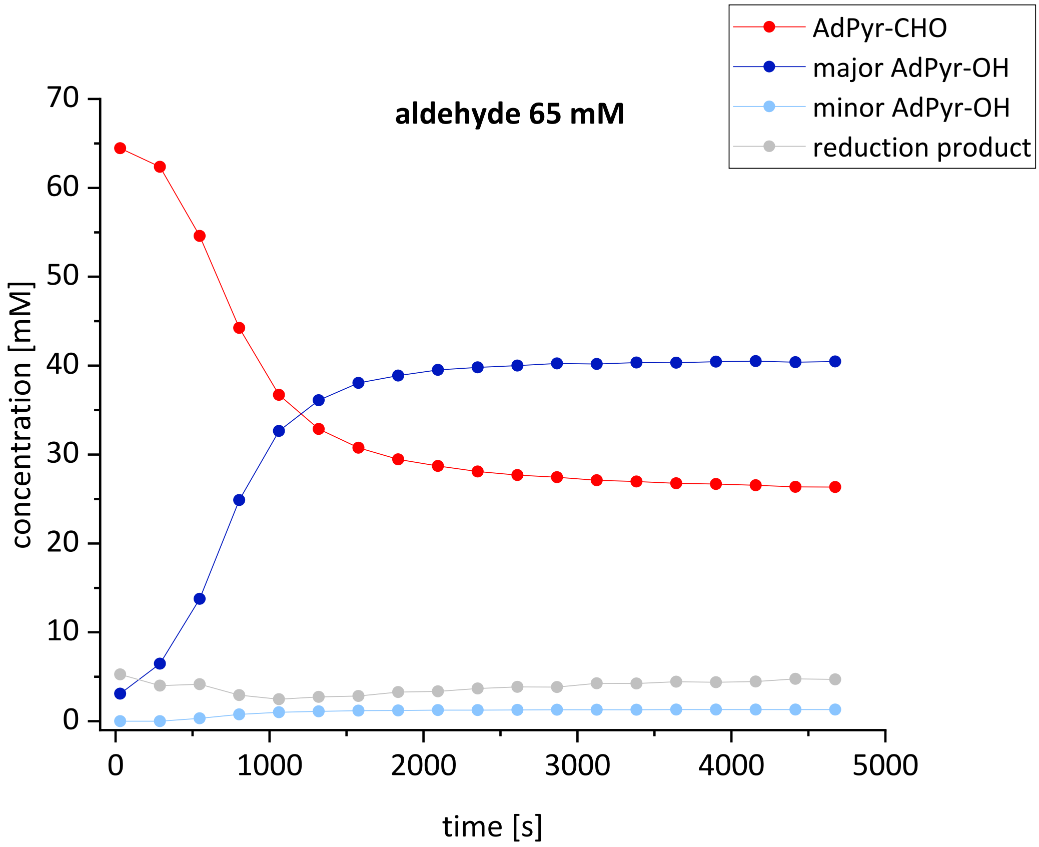

Supplementary Figure 46: Concentration-time profile of the Soai reaction in toluene (65 mM 6-((adamantan-1-yl)ethynyl)nicotinaldehyde **AdPyr-CHO**, 3.0 mM (1*R*)-1-(6-((adamantan-1-yl)ethynyl)pyridin-3-yl)-2-methylpropan-1-ol **AdPyr-OH** (*ee* > 99.9%) and 40 mM *i*Pr_2Zn; r.t.

4.3.3 Variation of the AdPyr-OH concentration

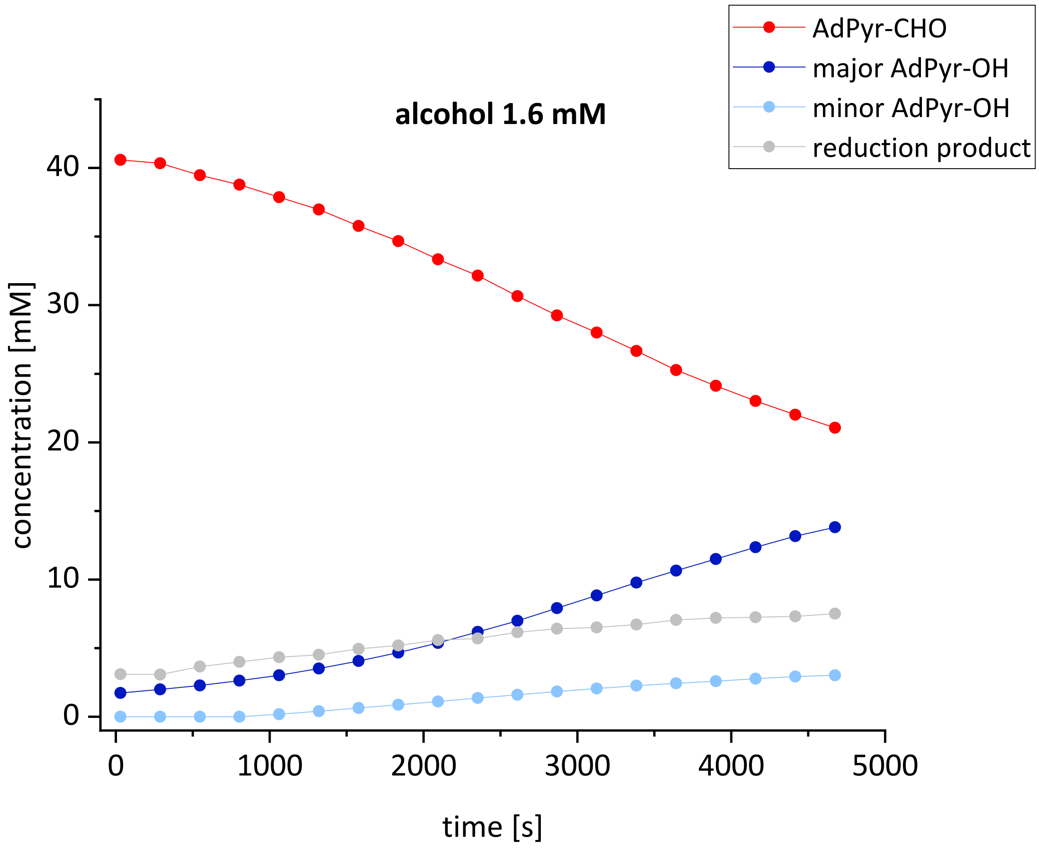

Supplementary Figure 47: Concentration-time profile of the Soai reaction in toluene (40 mM 6-((adamantan-1-yl)ethynyl)nicotinaldehyde **AdPyr-CHO**, 1.6 mM (1*R*)-1-(6-((adamantan-1-yl)ethynyl)pyridin-3-yl)-2-methylpropan-1-ol **AdPyr-OH** (*ee* > 99.9%) and 40 mM *i*Pr_2Zn; r.t.

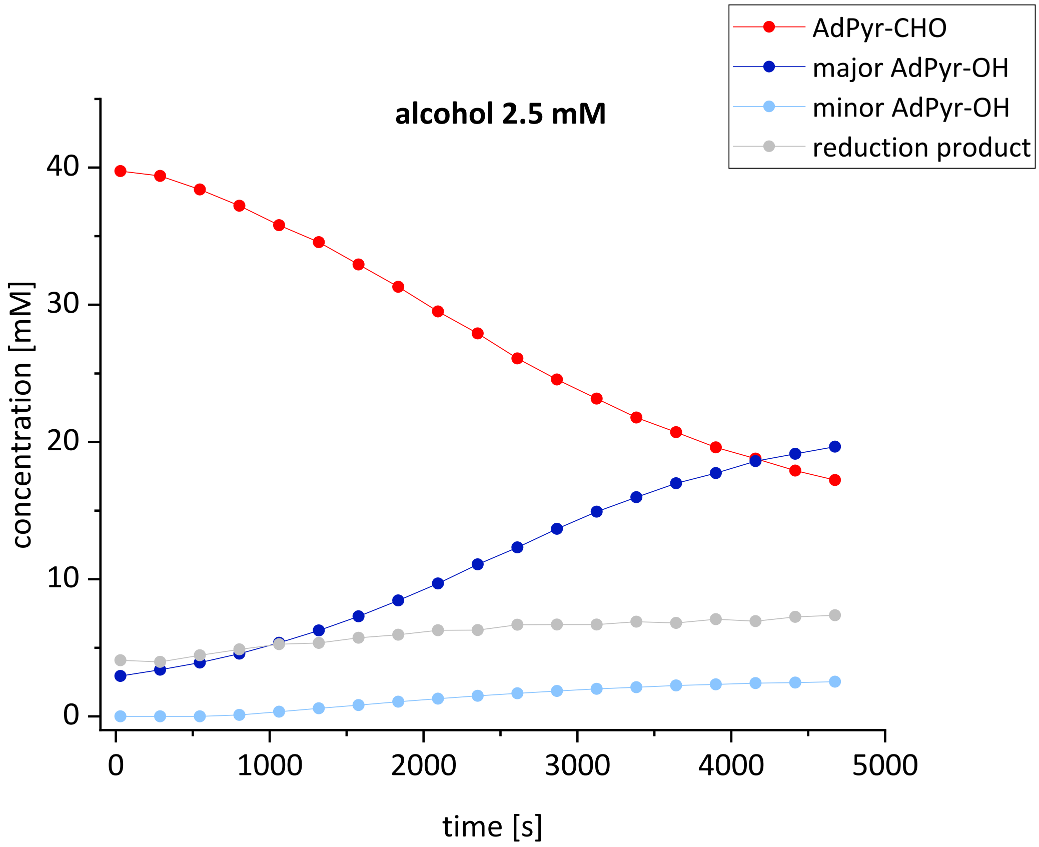

Supplementary Figure 48: Concentration-time profile of the Soai reaction in toluene (40 mM 6-((adamantan-1-yl)ethynyl)nicotinaldehyde **AdPyr-CHO**, 2.5 mM (1*R*)-1-(6-((adamantan-1-yl)ethynyl)pyridin-3-yl)-2-methylpropan-1-ol **AdPyr-OH** (*ee* > 99.9%) and 40 mM *i*Pr_2Zn; r.t.

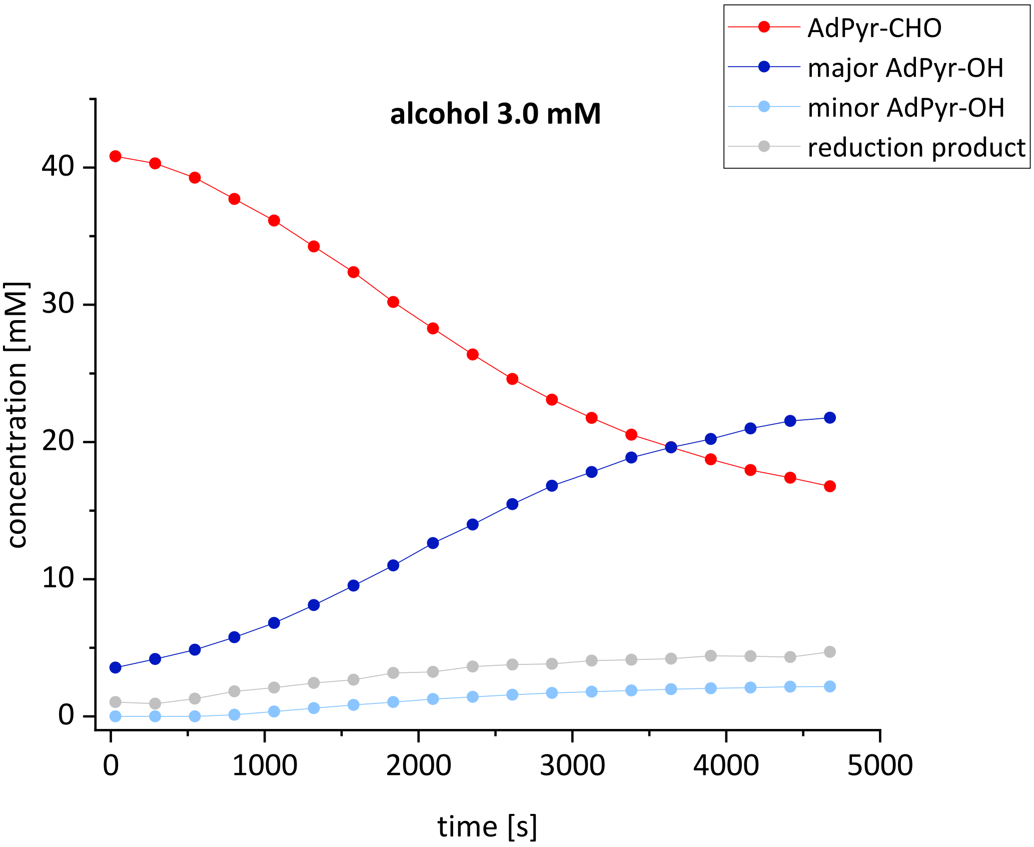

Supplementary Figure 49: Concentration-time profile of the Soai reaction in toluene (40 mM 6-((adamantan-1-yl)ethynyl)nicotinaldehyde **AdPyr-CHO**, 3.0 mM (1*R*)-1-(6-((adamantan-1-yl)ethynyl)pyridin-3-yl)-2-methylpropan-1-ol **AdPyr-OH** (*ee* > 99.9%) and 40 mM *i*Pr_2Zn; r.t.

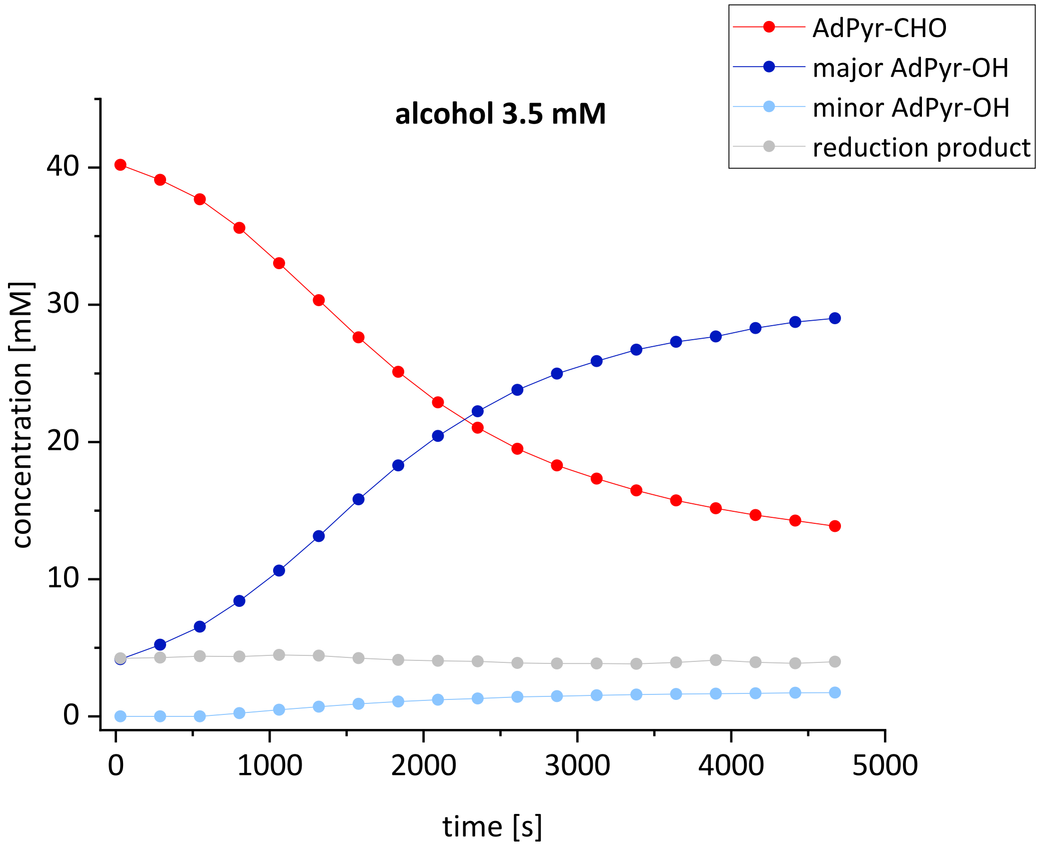

Supplementary Figure 50: Concentration-time profile of the Soai reaction in toluene (40 mM 6-((adamantan-1-yl)ethynyl)nicotinaldehyde **AdPyr-CHO**, 3.5 mM (1*R*)-1-(6-((adamantan-1-yl)ethynyl)pyridin-3-yl)-2-methylpropan-1-ol **AdPyr-OH** (*ee* > 99.9%) and 40 mM *i*Pr_2Zn; r.t.

4.3.4 Determination of the Reaction Orders

a. Reaction Order for aldehyde AdPyr-CHO

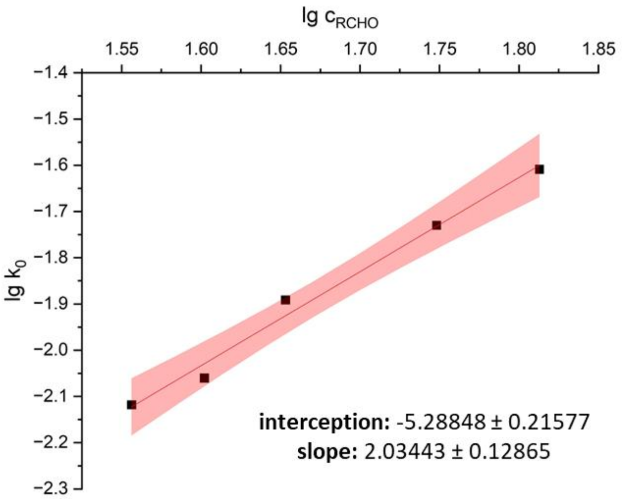

Supplementary Figure 51: Determination of the reaction order of **AdPyr-OH** by linear regression analysis of lg(k_0) vs. lg($c_{aldehyde}$).

b. Reaction Order for alcohol AdPyr-OH

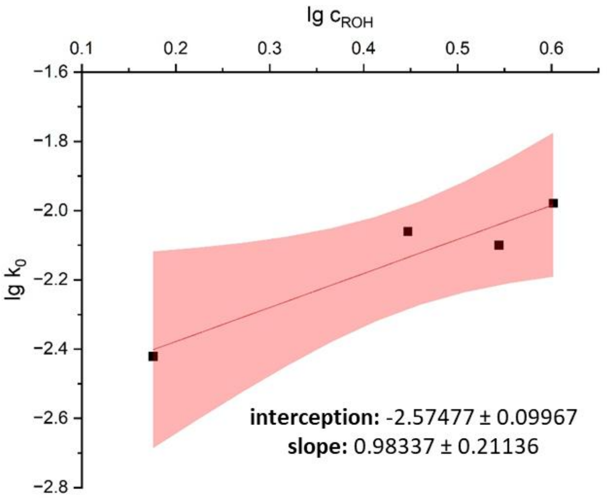

Supplementary Figure 52: Determination of the reaction order of **AdPyr-OH** by linear regression analysis of lg(k_0) *vs.* lg($c_{alcohol}$).

4.4 AdPym-CHO/AdPym-OH System

4.4.1 Calibration plots for quantitative analysis of kinetic measurements

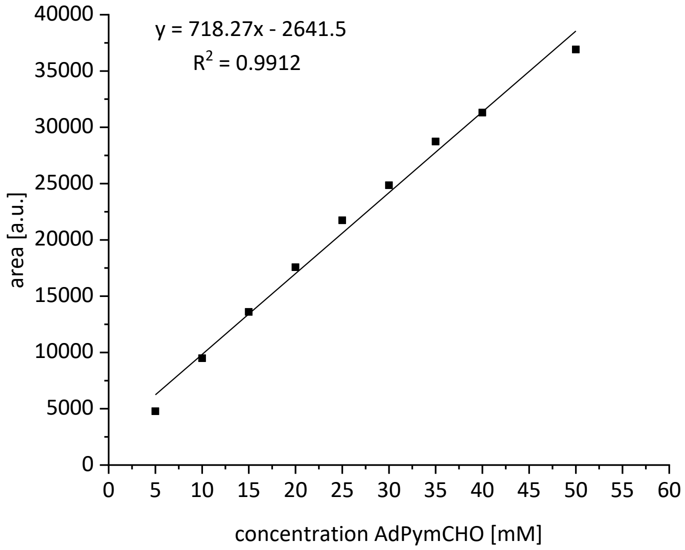

Supplementary Figure 53: Calibration plot on HPLC for **AdPym-CHO** on a Chiralpak IC® column (250 mm, i.D. 20 mm, particle size: 5 µm), *n*-hexane/THF = 75/25, 1.0 mL/min, λ = 280 nm, r.t.

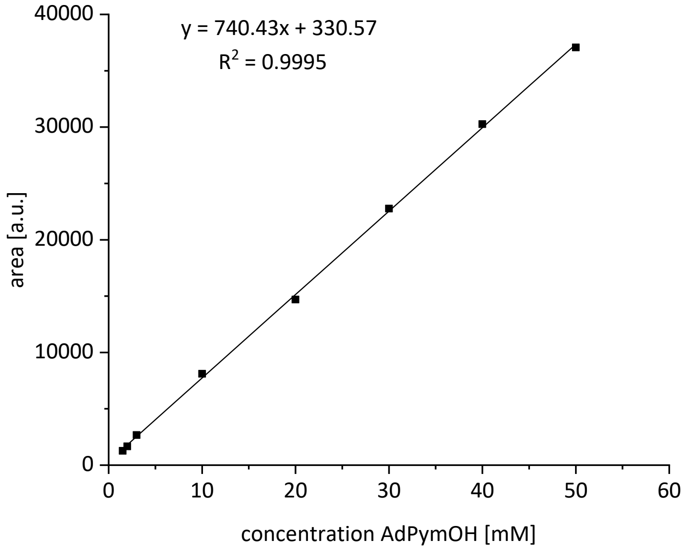

Supplementary Figure 54: Calibration plot on HPLC for **AdPym-OH** on a Chiralpak IC® column (250 mm, i.D. 20 mm, particle size: 5 µm), *n*-hexane/THF = 75/25, 1.0 mL/min, λ = 250 nm, r.t.

4.4.2 Variation of the AdPym-CHO concentration

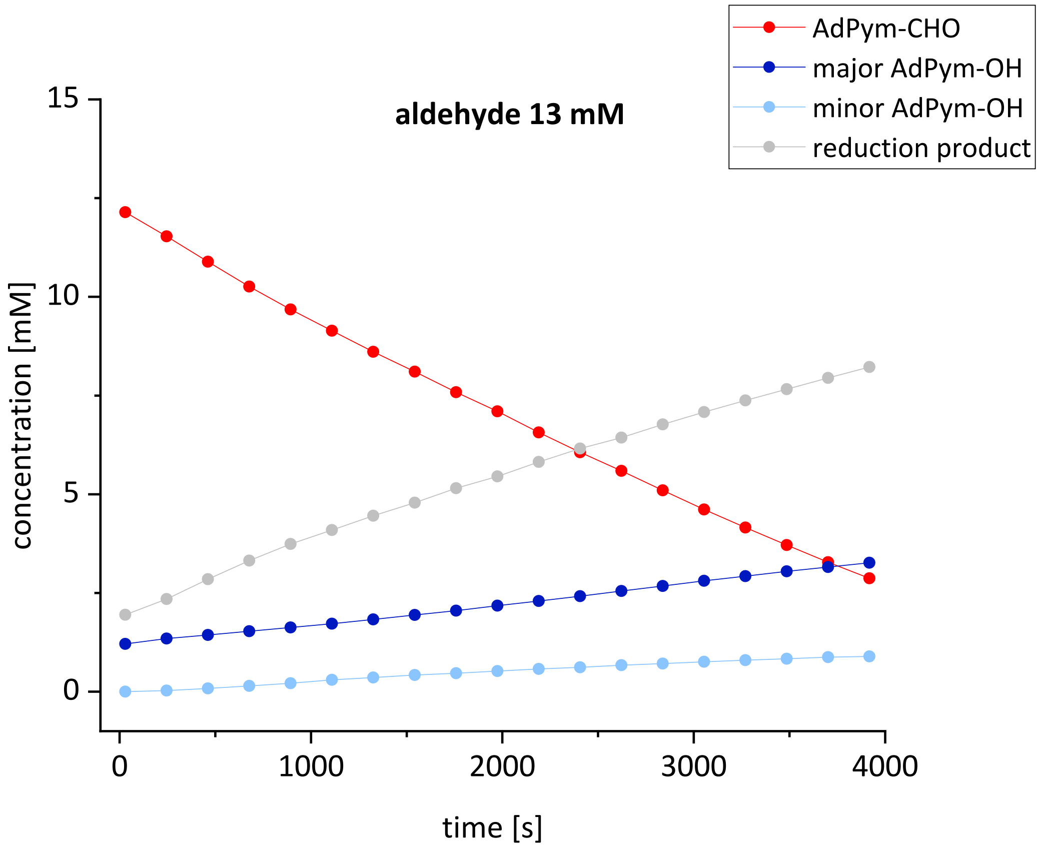

Supplementary Figure 55: Concentration-time profile of the Soai reaction in toluene (13 mM 2-((adamantan-1-yl)ethynyl)pyrimidine-5-carbaldehyde **AdPym-CHO,** 1.5 mM (1*R*)-1-(2-((adamantan-1-yl)ethynyl)pyrimidin-5-yl)-2-methylpropan-1-ol AdPym-OH (*ee* > 99.9%) and 40 mM *i*Pr_2Zn; r.t.

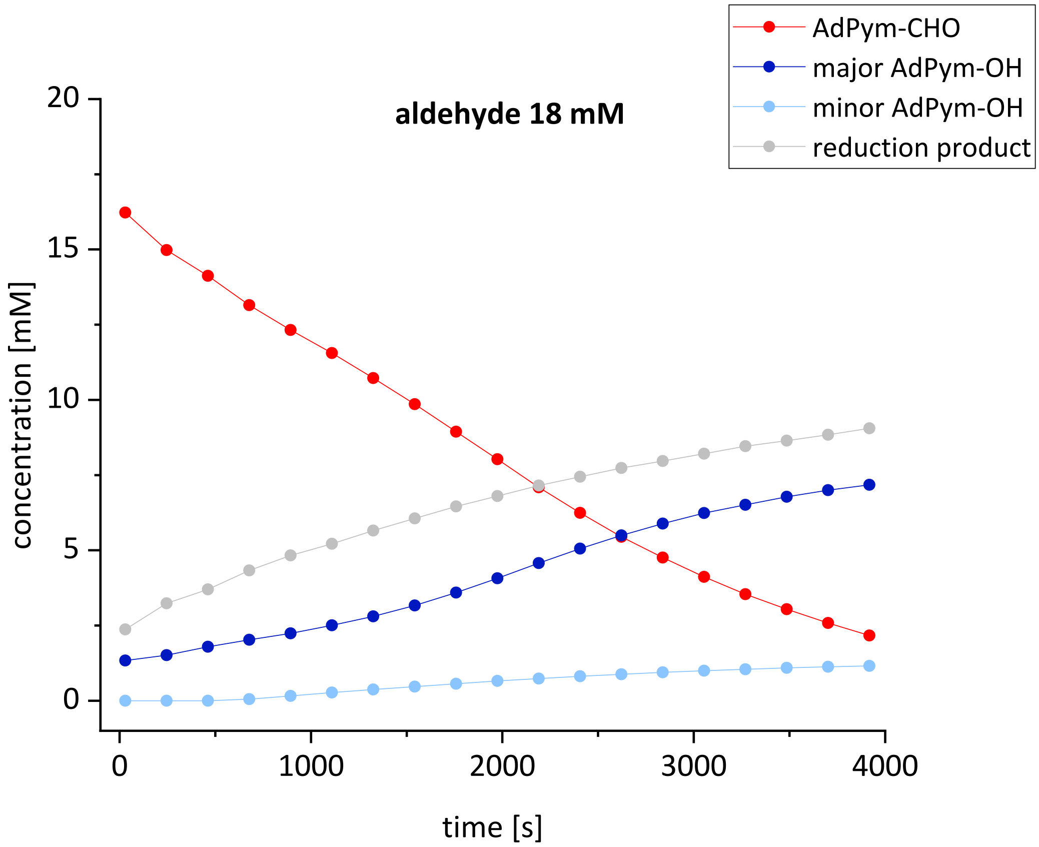

Supplementary Figure 56: Concentration-time profile of the *Soai* reaction in toluene (18 mM 2-((adamantan-1-yl)ethynyl)pyrimidine-5-carbaldehyde AdPym-CHO, 1.5 mM (1*R*)-1-(2-((adamantan-1-yl)ethynyl)pyrimidin-5-yl)-2-methylpropan-1-ol AdPym-OH (*ee* > 99.9%) and 40 mM *i*Pr_2Zn; r.t.

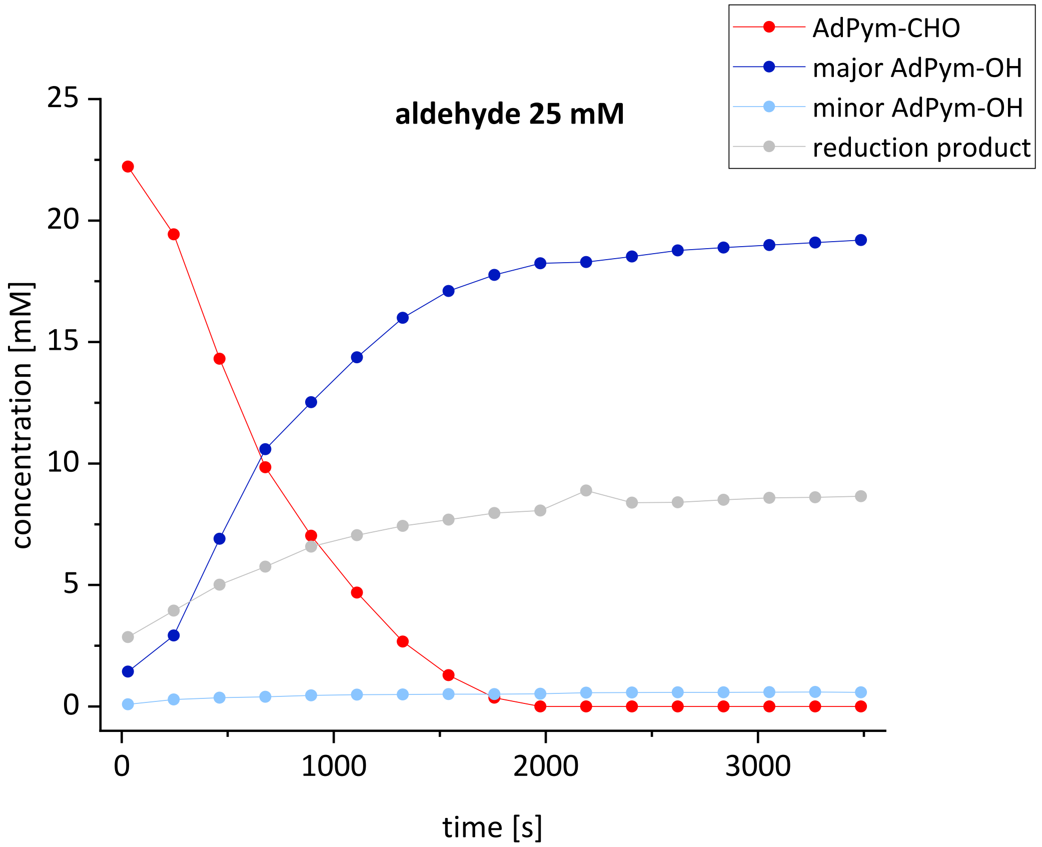

Supplementary Figure 57: Concentration-time profile of the Soai reaction in toluene (25 mM 2-((adamantan-1-yl)ethynyl)pyrimidine-5-carbaldehyde **AdPym-CHO**, 1.5 mM (1*R*)-1-(2-((adamantan-1-yl)ethynyl)pyrimidin-5-yl)-2-methylpropan-1-ol **AdPym-OH** (*ee* > 99.9%) and 40 mM $i\mathrm{Pr}_2\mathrm{Zn}$; r.t.

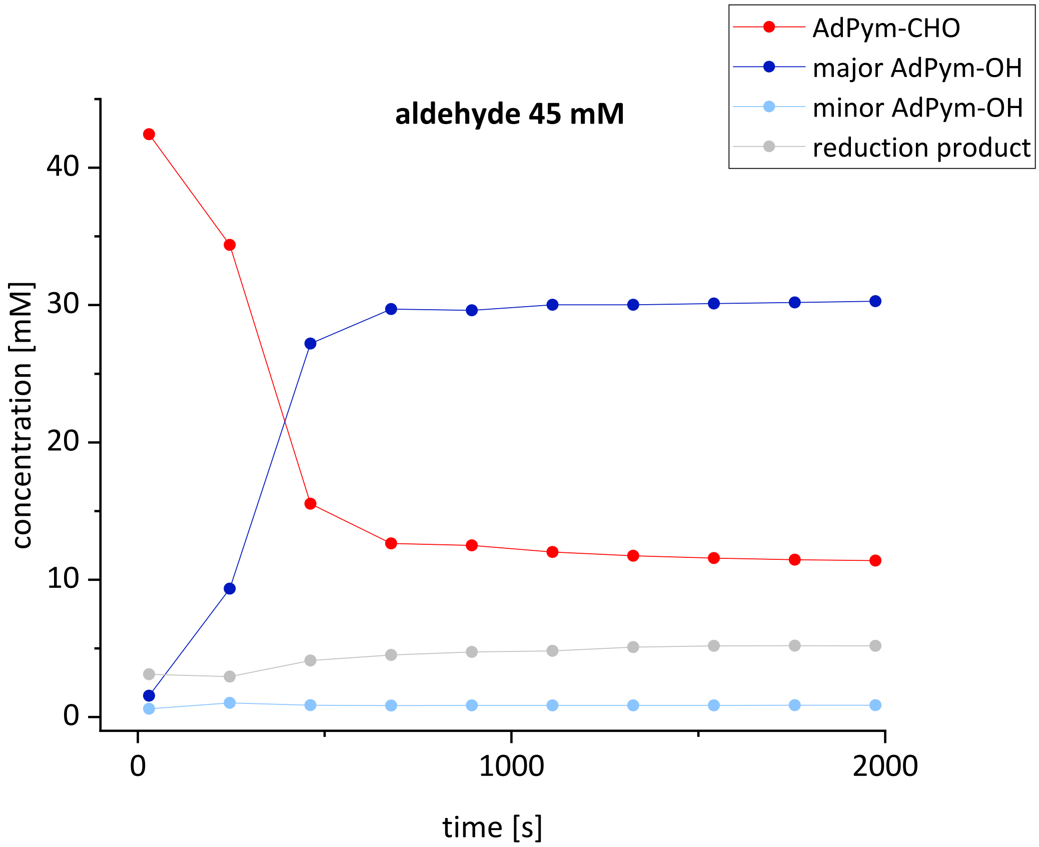

Supplementary Figure 58: Concentration-time profile of the *Soai* reaction in toluene (45 mM 2-((adamantan-1-yl)ethynyl)pyrimidine-5-carbaldehyde **AdPym-CHO**, 1.5 mM (1*R*)-1-(2-((adamantan-1-yl)ethynyl)pyrimidin-5-yl)-2-methylpropan-1-ol **AdPym-OH** (*ee* > 99.9%) and 40 mM *i*Pr_2Zn; r.t.

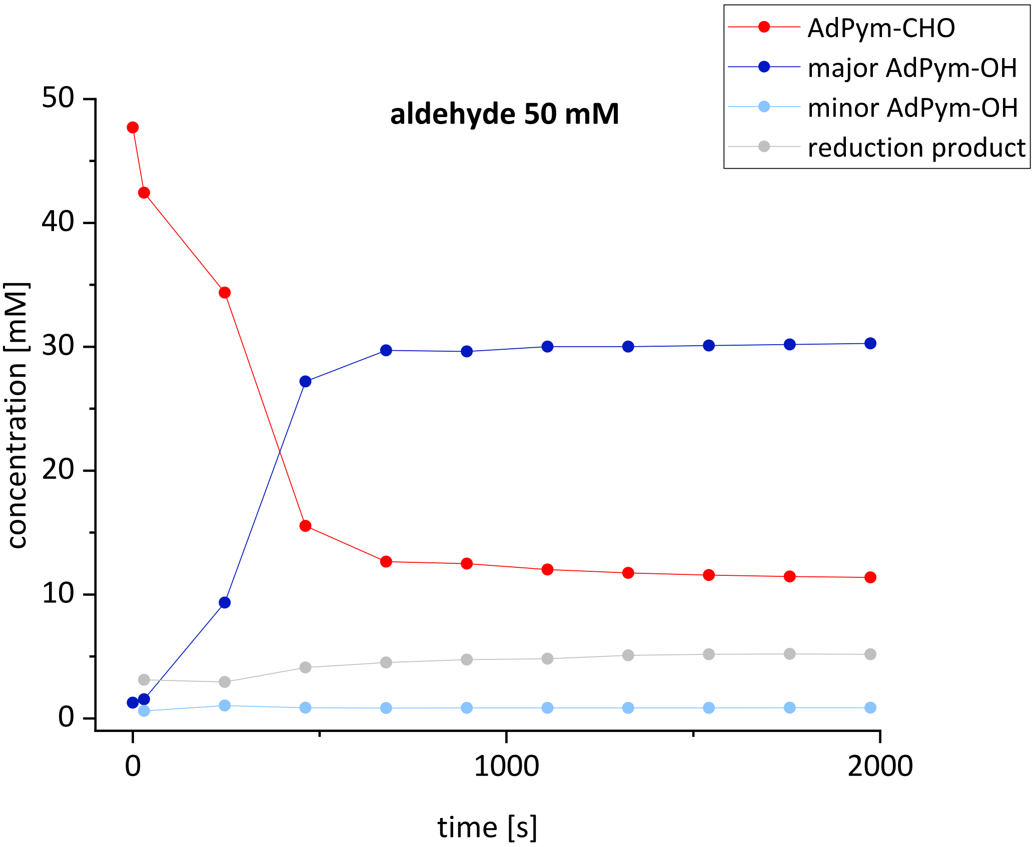

Supplementary Figure 59: Concentration-time profile of the Soai reaction in toluene (50 mM 2-((adamantan-1-yl)ethynyl)pyrimidine-5-carbaldehyde **AdPym-CHO**, 1.5 mM (1*R*)-1-(2-((adamantan-1-yl)ethynyl)pyrimidin-5-yl)-2-methylpropan-1-ol **AdPym-OH** (*ee* > 99.9%) and 40 mM *i*Pr_2Zn; r.t.

4.4.3 Variation of the AdPym-OH concentration

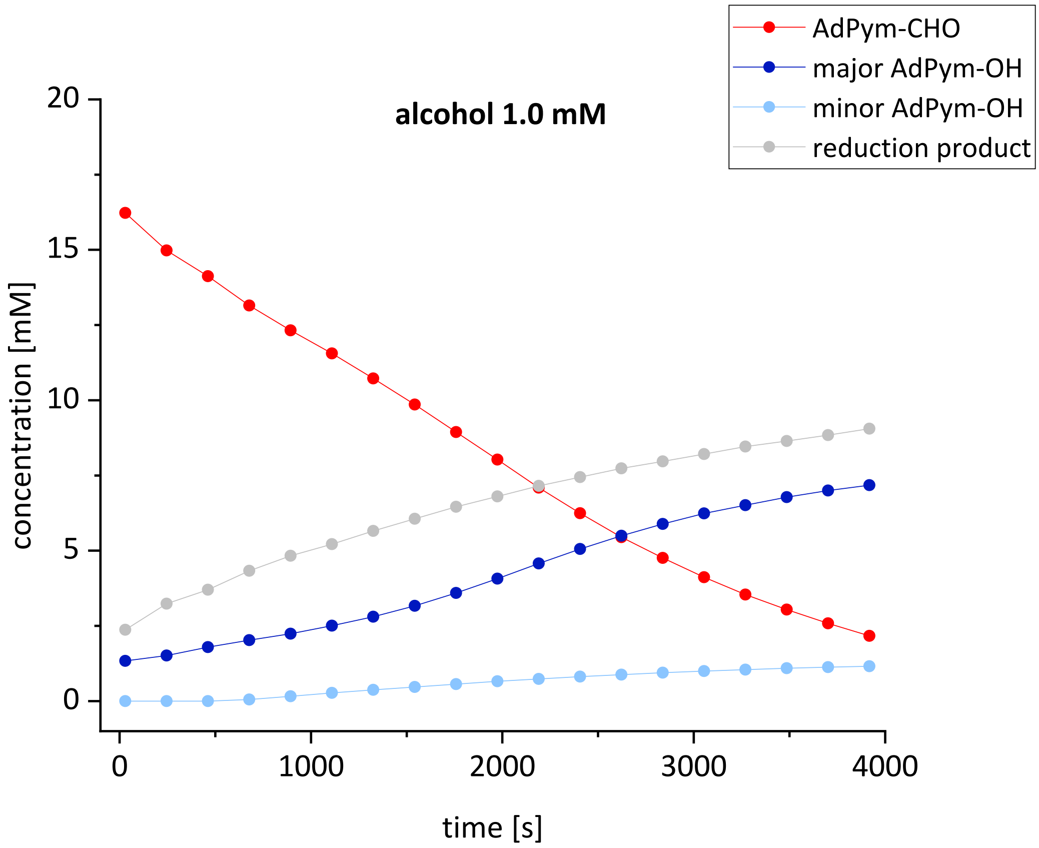

Supplementary Figure 60: Concentration-time profile of the Soai reaction in toluene (20 mM 2-((adamantan-1-yl)ethynyl)pyrimidine-5-carbaldehyde **AdPym-CHO**, 1.0 mM (1*R*)-1-(2-((adamantan-1-yl)ethynyl)pyrimidin-5-yl)-2-methylpropan-1-ol **AdPym-OH** (*ee* > 99.9%) and 40 mM *i*Pr_2Zn; r.t**.**

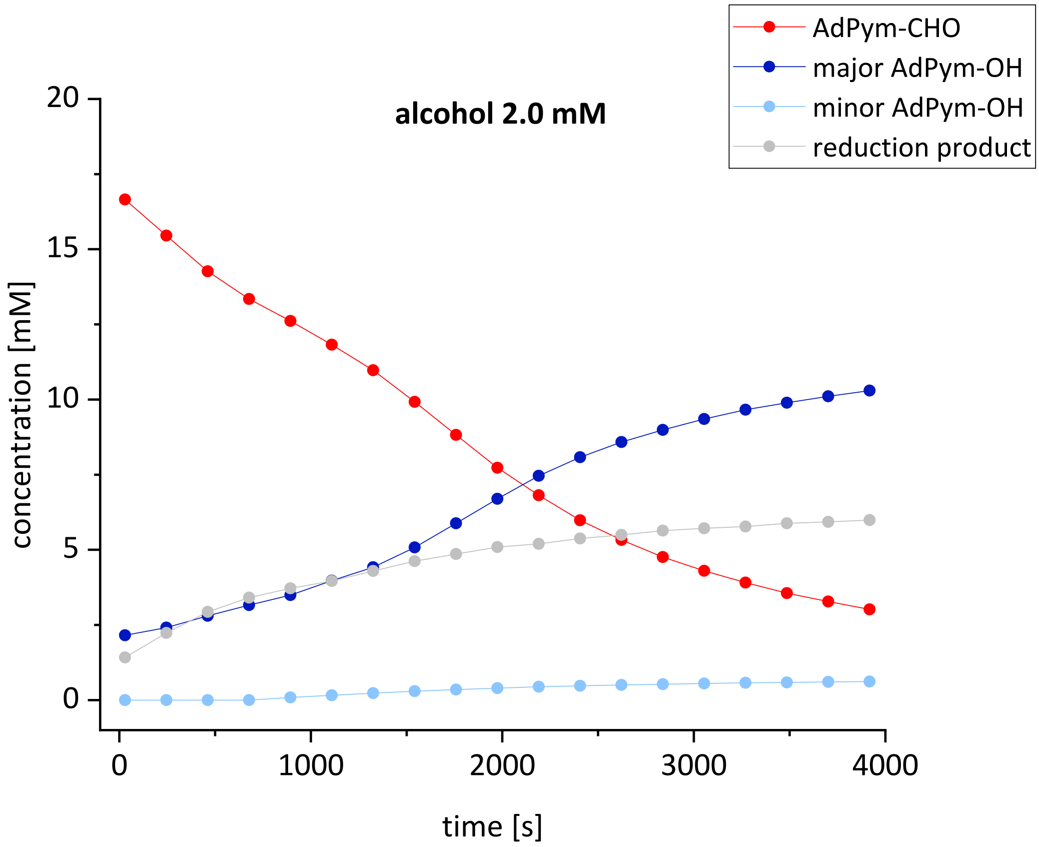

Supplementary Figure 61: Concentration-time profile of the Soai reaction in toluene (20 mM 2-((adamantan-1-yl)ethynyl)pyrimidine-5-carbaldehyde **AdPym-CHO**, 2.0 mM (1*R*)-1-(2-((adamantan-1-yl)ethynyl)pyrimidin-5-yl)-2-methylpropan-1-ol **AdPym-OH** (*ee* > 99.9%) and 40 mM $i\mathrm{Pr_2Zn}$; r.t.

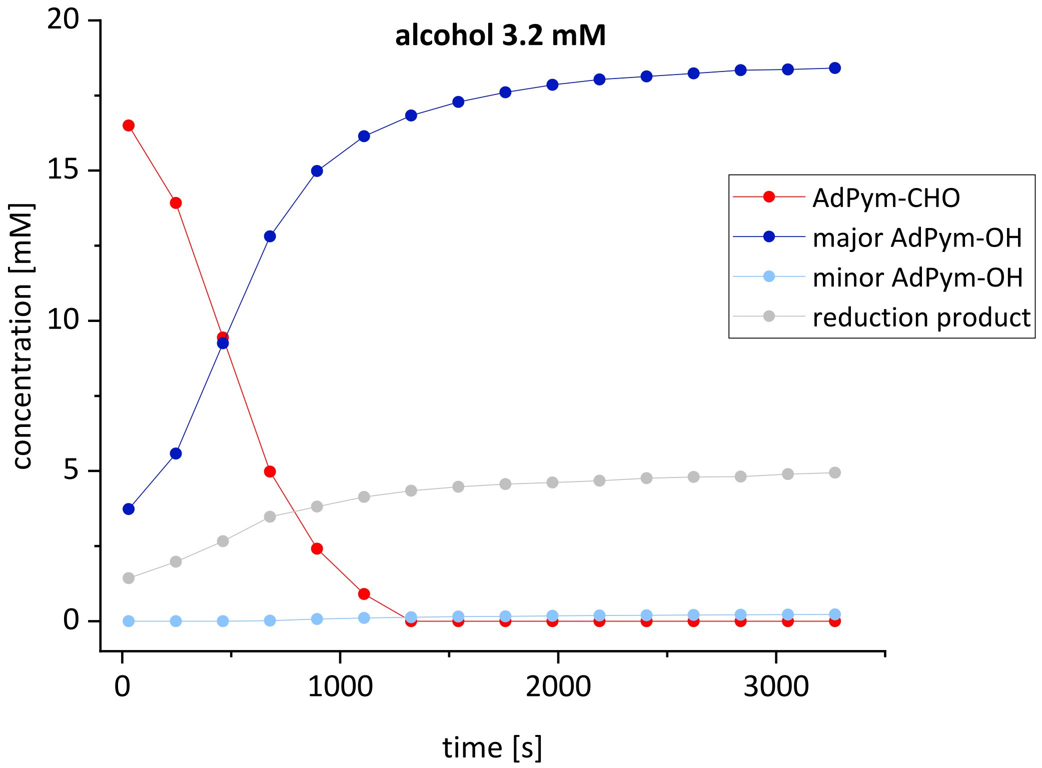

Supplementary Figure 62: Concentration-time profile of the Soai reaction in toluene (20 mM 2-((adamantan-1-yl)ethynyl)pyrimidine-5-carbaldehyde **AdPym-CHO**, 3.2 mM (1*R*)-1-(2-((adamantan-1-yl)ethynyl)pyrimidin-5-yl)-2-methylpropan-1-ol **AdPym-OH** (*ee* > 99.9%) and 40 mM $i\text{Pr}_2\text{Zn}$; r.t.

4.4.4 Determination of the Reaction Orders

a. Reaction Order for aldehyde AdPym-CHO

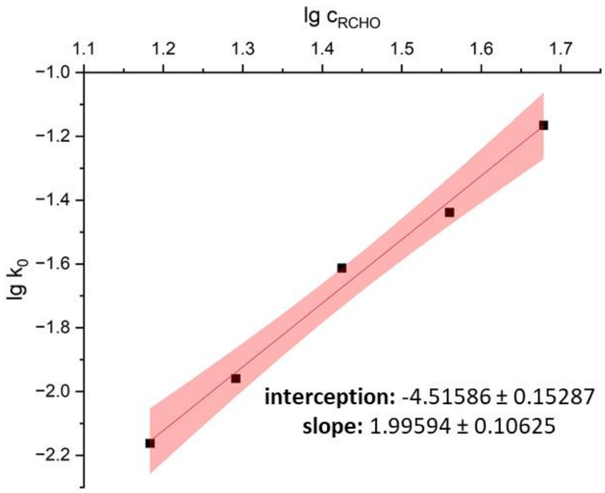

Supplementary Figure 63: Determination of the reaction order of **AdPym-OH** by linear regression analysisof lg(k_0) *vs.* lg($c_{aldehyde}$).

b. Reaction Order for alcohol AdPym-OH

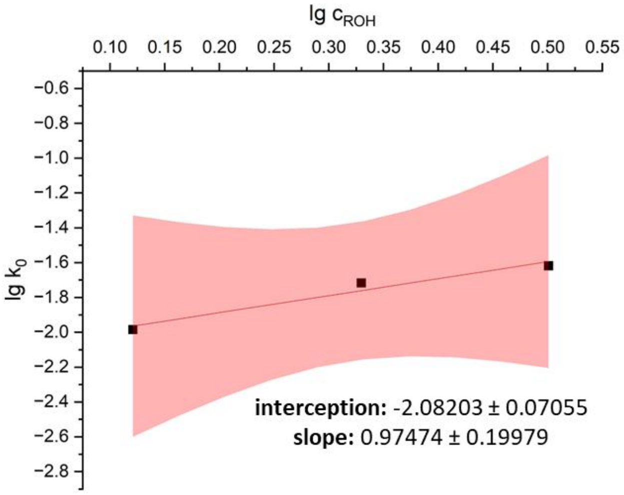

Supplementary Figure 64: Determination of the reaction order of **AdPym-OH** by linear regression analysis of lg(k_0) *vs.* lg($c_{alcohol}$).

4.5 TMSPym-CHO/TMSPym-OH System

4.5.1 Calibration plots for quantitative analysis of kinetic measurements

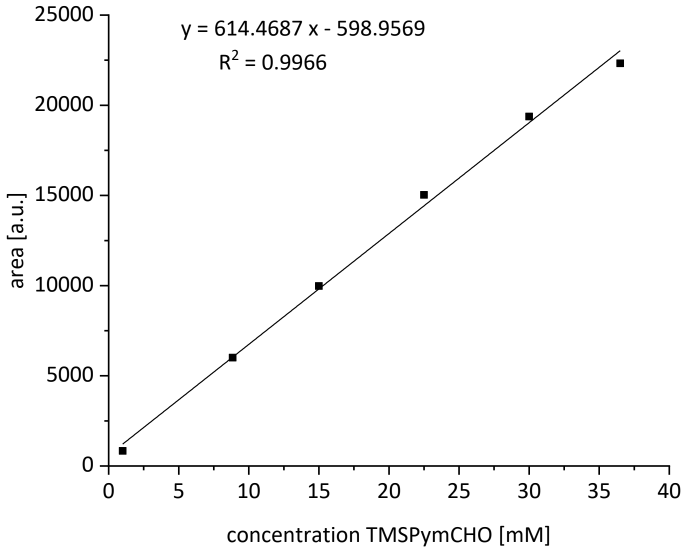

Supplementary Figure 65: Calibration plot on HPLC for **TMSPym-CHO** on a Chiralpak IB® column (250 mm, i.D. 4.6 mm, particle size: 5 µm), *n*-hexane/THF = 70/30, 1.2 mL, λ = 280 nm, r.t.

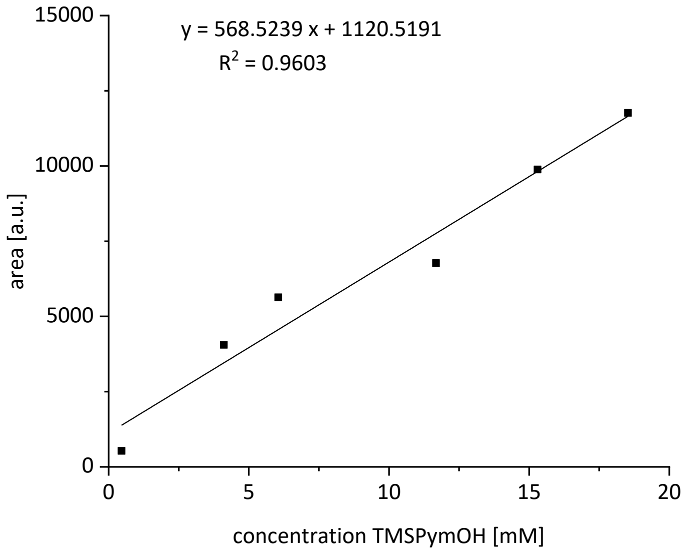

Supplementary Figure 66: Calibration plot on HPLC for **TMSPym-OH** on a Chiralpak IB® column (250 mm, i.D. 4.6 mm, particle size: 5 µm), *n*-hexane/THF = 70/30, 1.2 mL, λ = 250 nm, r.t.

4.5.2 Variation of the TMSPym-CHO concentration

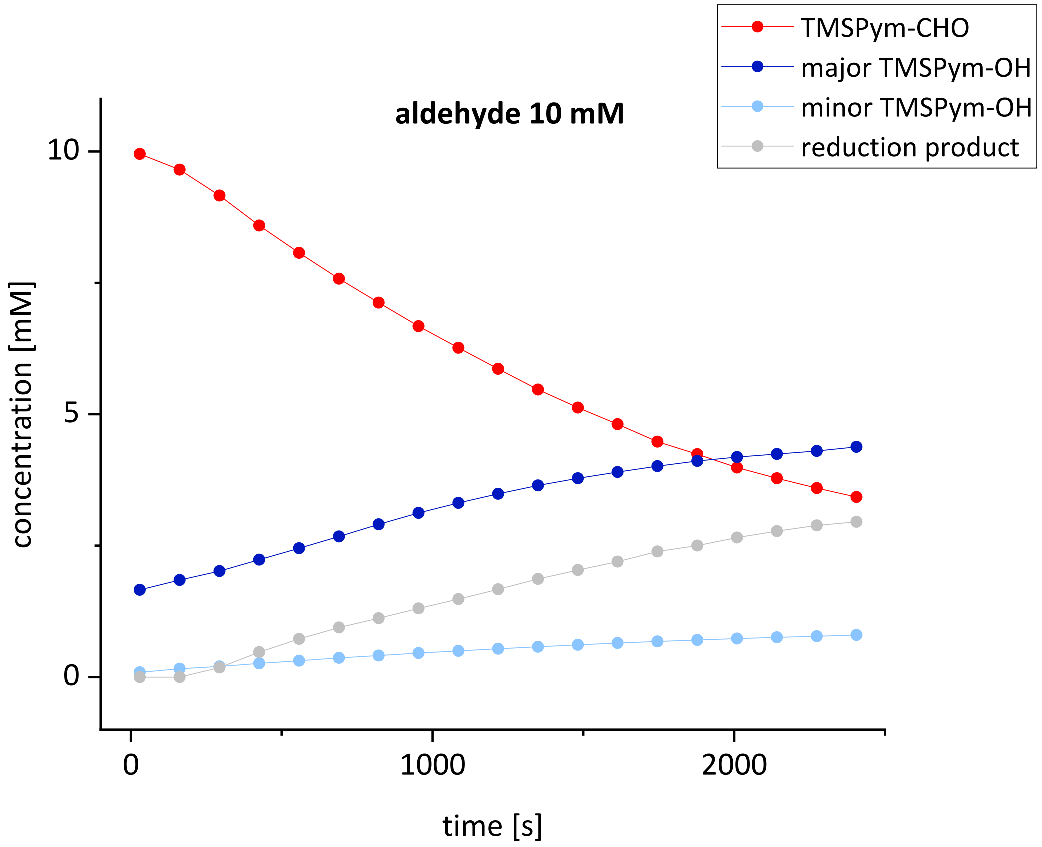

Supplementary Figure 67: Concentration-time profile of the Soai reaction in toluene (10 mM 2-((trimethylsilyl)ethynyl)pyrimidine-5-carbaldehyde **TMSPym-CHO**, 1.5 mM (1*R*)-2-methyl-1-(2-((trimethylsilyl)ethynyl)pyrimidin-5-yl)propan-1-ol **TMSPym-OH** (*ee* > 99.9%) and 40 mM *i*Pr_2Zn; r.t.

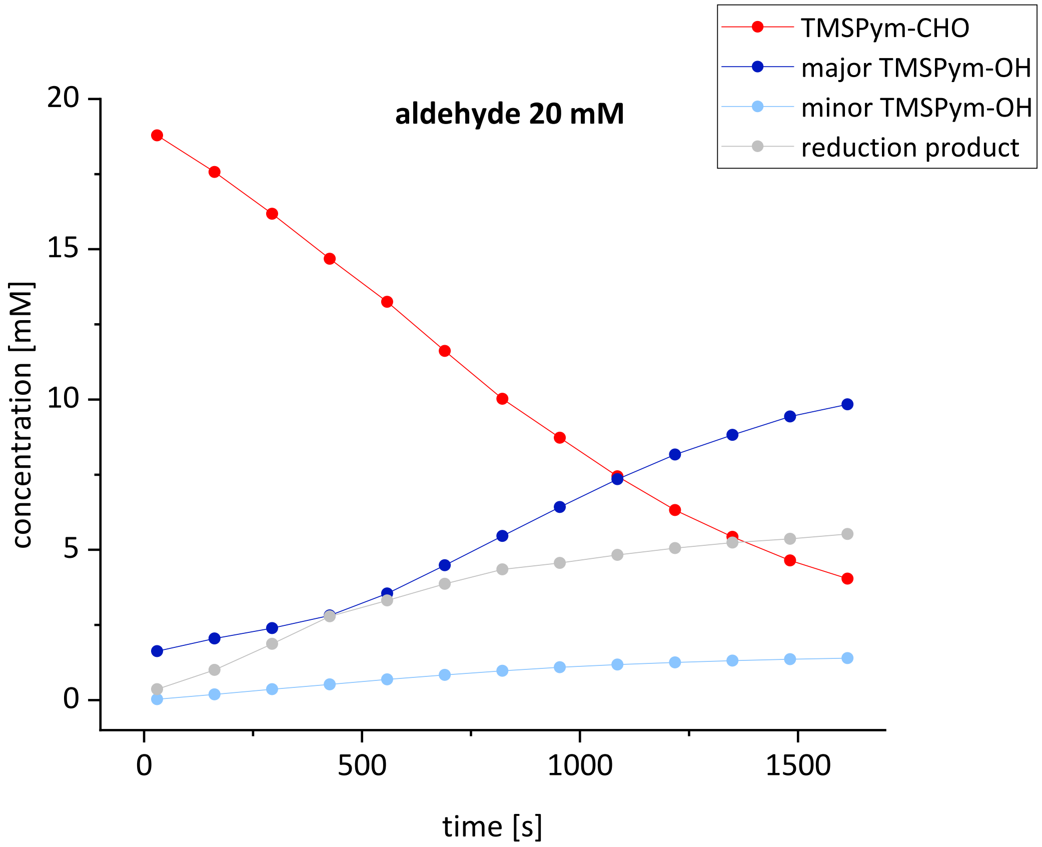

Supplementary Figure 68: Concentration-time profile of the Soai reaction in toluene (20 mM 2-((trimethylsilyl)ethynyl)pyrimidine-5-carbaldehyde **TMSPym-CHO**, 1.5 mM (1*R*)-2-methyl-1-(2-((trimethylsilyl)ethynyl)pyrimidin-5-yl)propan-1-ol **TMSPym-OH** (*ee* > 99.9%) and 40 mM *i*Pr_2Zn; r.t.

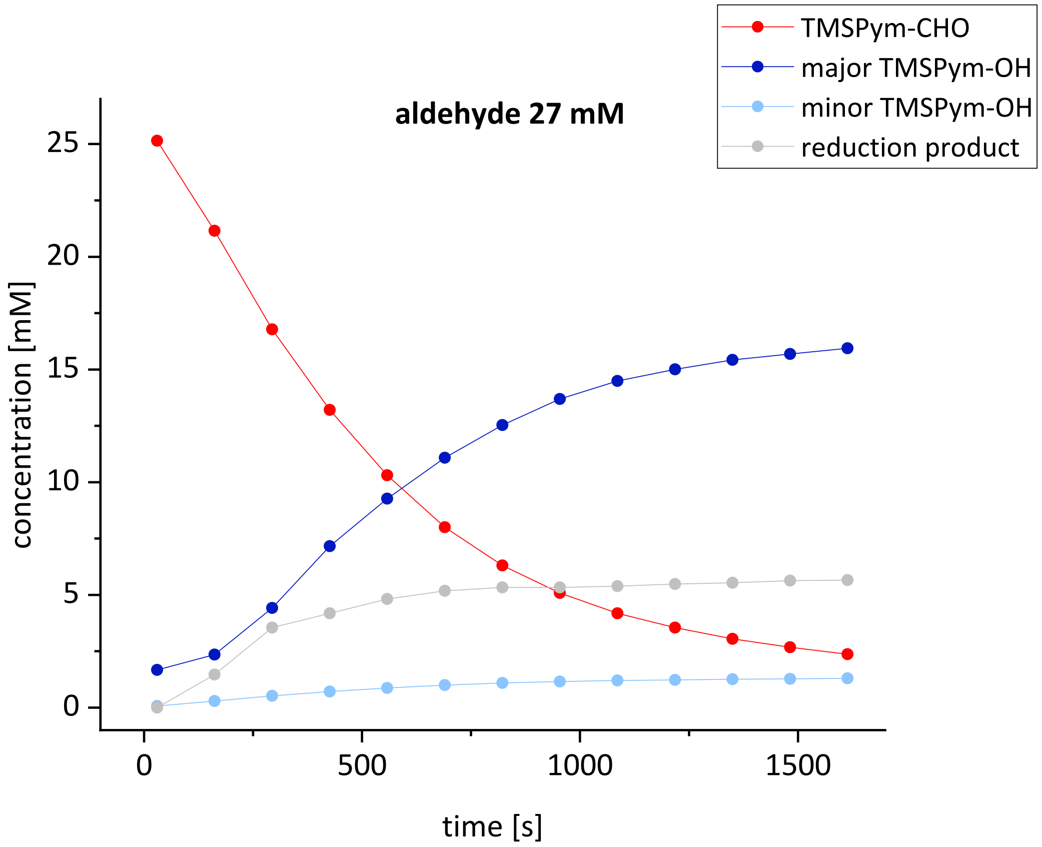

Supplementary Figure 69: Concentration-time profile of the Soai reaction in toluene (27 mM 2-((trimethylsilyl)ethynyl)pyrimidine-5-carbaldehyde **TMSPym-CHO**, 1.5 mM (1*R*)-2-methyl-1-(2-((trimethylsilyl)ethynyl)pyrimidin-5-yl)propan-1-ol **TMSPym-OH** (*ee* > 99.9%) and 40 mM *i*Pr_2Zn; r.t.

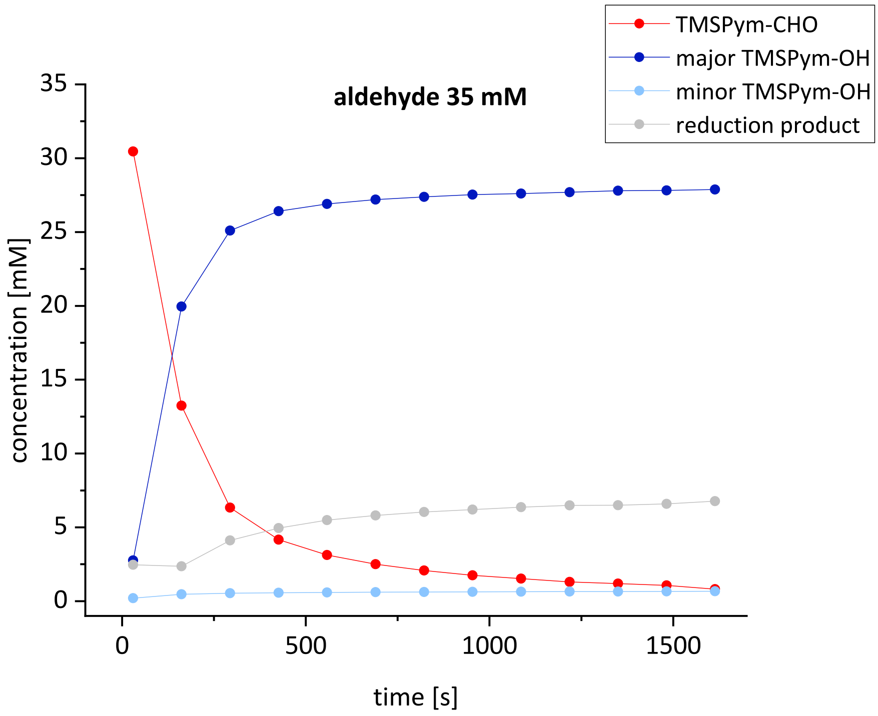

Supplementary Figure 70: Concentration-time profile of the Soai reaction in toluene (35 mM 2-((trimethylsilyl)ethynyl)pyrimidine-5-carbaldehyde **TMSPym-CHO**, 1.5 mM (1*R*)-2-methyl-1-(2-((trimethylsilyl)ethynyl)pyrimidin-5-yl)propan-1-ol **TMSPym-OH** (*ee* > 99.9%) and 40 mM *i*Pr_2Zn; r.t.

4.5.3 Variation of the TMSPym-OH concentration

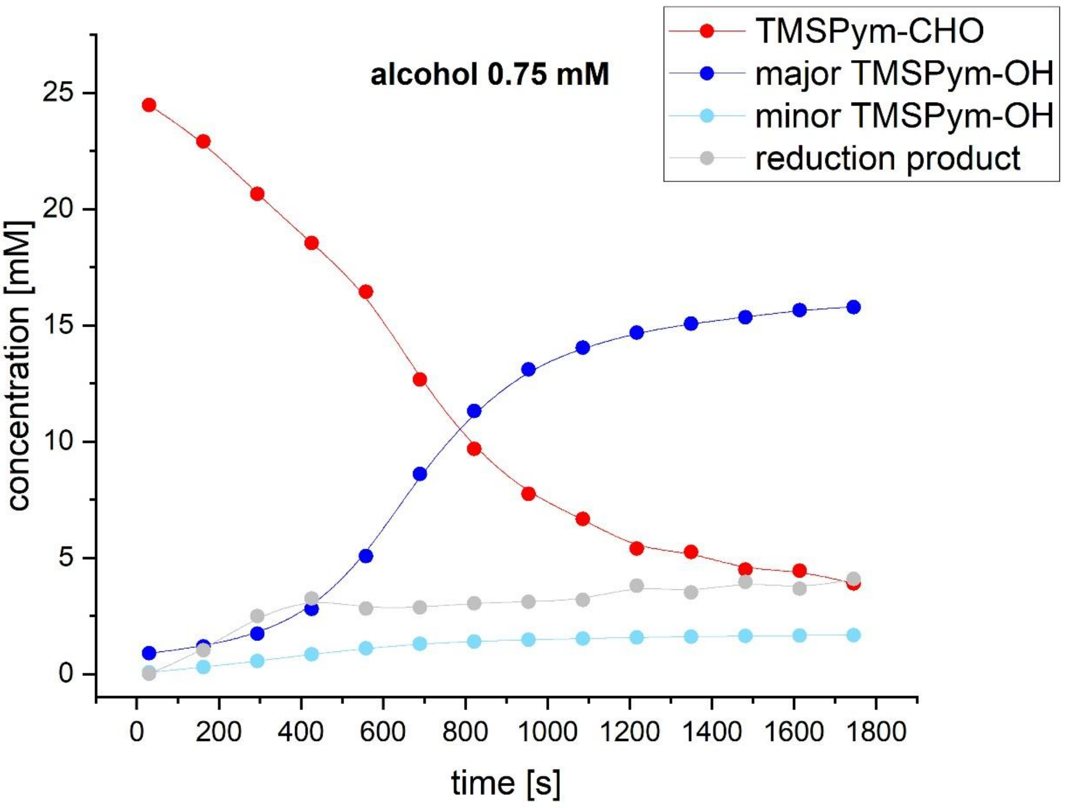

Supplementary Figure 71: Concentration-time profile of the Soai reaction in toluene (25 mM 2-((trimethylsilyl)ethynyl)pyrimidine-5-carbaldehyde **TMSPym-CHO**, 0.75 mM (1*R*)-2-methyl-1-(2-((trimethylsilyl)ethynyl)pyrimidin-5-yl)propan-1-ol **TMSPym-OH** (*ee* > 99.9%) and 40 mM *i*Pr_2Zn; r.t.

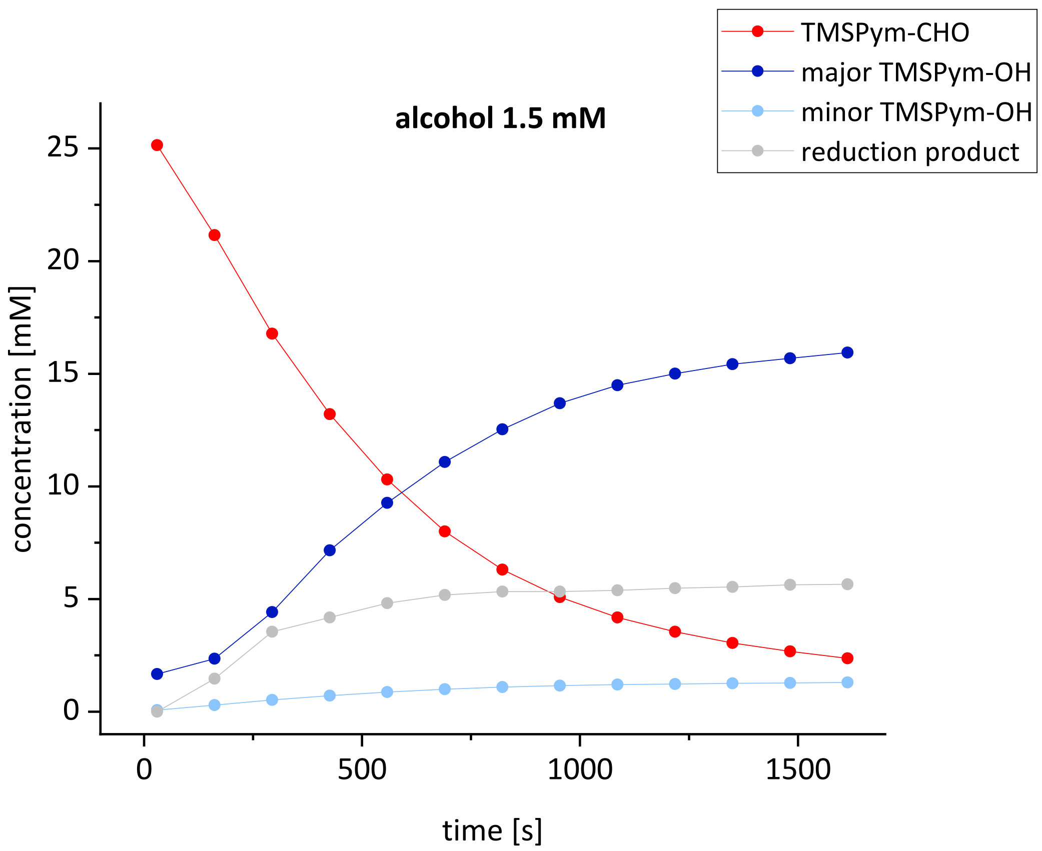

Supplementary Figure 72: Concentration-time profile of the Soai reaction in toluene (25 mM 2-((trimethylsilyl)ethynyl)pyrimidine-5-carbaldehyde **TMSPym-CHO**, 1.5 mM (1*R*)-2-methyl-1-(2-((trimethylsilyl)ethynyl)pyrimidin-5-yl)propan-1-ol **TMSPym-OH** (*ee* > 99.9%) and 40 mM *i*Pr_2Zn; r.t.

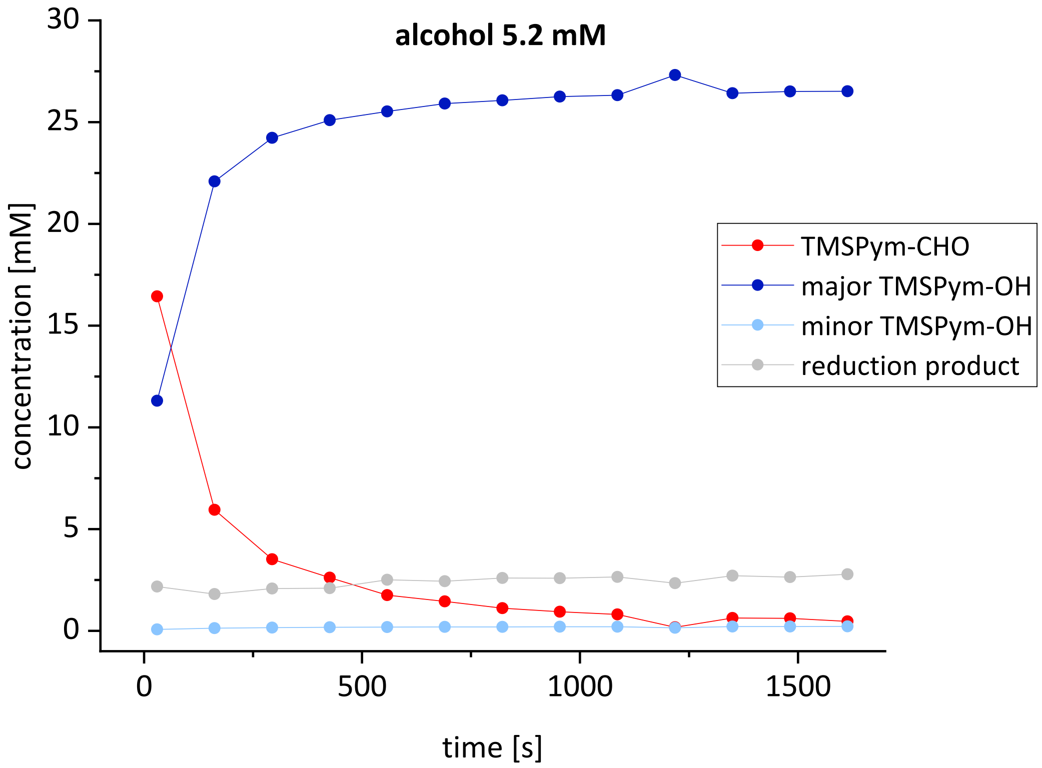

Supplementary Figure 73: Concentration-time profile of the Soai reaction in toluene (25 mM 2-((trimethylsilyl)ethynyl)pyrimidine-5-carbaldehyde **TMSPym-CHO**, 5.2 mM (1*R*)-2-methyl-1-(2-((trimethylsilyl)ethynyl)pyrimidin-5-yl)propan-1-ol **TMSPym-OH** (*ee* > 99.9%) and 40 mM *i*Pr_2Zn; r.t.

4.5.4 Determination of the Reaction Orders

a. Reaction Order for aldehyde TMSPym-CHO

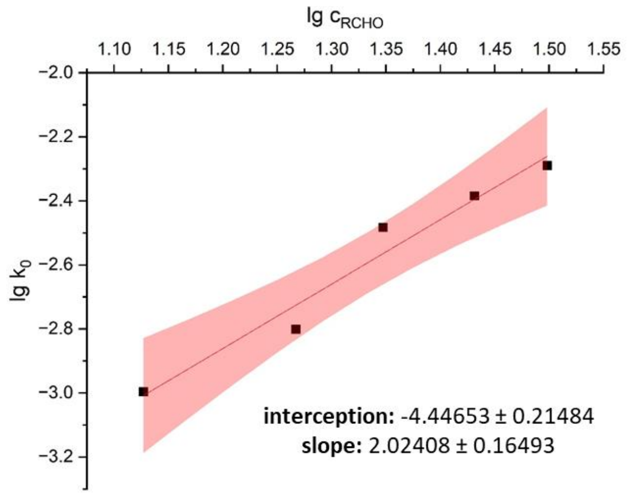

Supplementary Figure 74: Determination of the reaction order of **TMSPym-OH** by linear regression analysis of lg(k_0) *vs.* lg($c_{aldehyde}$).

b. Reaction Order for alcohol TMSPy,-OH

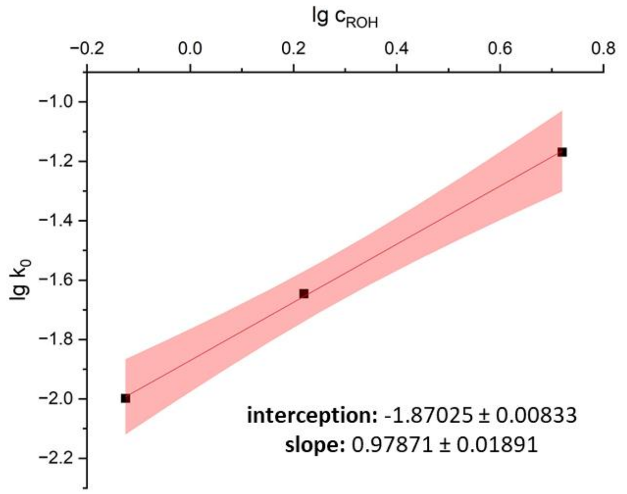

Supplementary Figure 75: Determination of the reaction order of **TMSPym-OH** by linear regression analysis of lg(k_0) *vs.* lg($c_{alcohol}$).

5 Dynamic HPLC measurements of hemiacetal formation

5.1 Enantioselective dynamic HPLC measurements

Dynamic HPLC (DHPLC) measurements were performed on an *Agilent* 1200 Infinity HPLC equipped with a binary pump, an autosampler (*Agilent* HiP+), a thermostated column oven and a photodiode array detector (DAD). All operations were controlled by the *Agilent Chemstation* software. The separations were performed on an immobilized stationary phase using *n*-hexane/*i*PrOH = 60/40 as mobile phase at a flow rate of 1.0 mL/min.

The on-column equilibrium between the aldehyde and the hemiacetal of the pyridine system **TMSPyr**, was monitored on a Chiralpak® IC column (250 mm, i.D. 4.6 mm, particle size: 5 µm) from *Chiral Technologies*. The equilibrium was monitored between 10 °C and 70 °C.

The on-column equilibrium between the aldehyde and the hemiacetal of the pyridine system **AdPyr**, was monitored on a Chiralpak® IA column (250 mm, i.D. 4.6 mm, particle size: 5 µm) from *Chiral Technologies*. The equilibrium was monitored between 10 °C and 70 °C.

The on-column equilibrium between the aldehyde and the hemiacetal of the pyridine system **TMSPym**, was monitored on a Chiralpak® IB column (250 mm, i.D. 4.6 mm, particle size: 5 µm) from *Chiral Technologies*. The equilibrium was monitored between 10 °C and 60 °C.

The on-column equilibrium between the aldehyde and the hemiacetal of the pyridine system **AdPym**, was monitored on a Chiralpak® IB column (250 mm, i.D. 4.6 mm, particle size: 5 µm) from *Chiral Technologies*. The equilibrium was monitored between 10 °C and 60 °C

5.2 Evaluation of the dynamic HPLC profiles

The evaluation of chromatographic profiles obtained from the DHPLC measurements was conducted according to a previously described method.[7] The rate constants *k* of hemiacetal formation were determined by analysis of the chromatographic peak profiles using the unified equation of dynamic chromatography implemented in the software *DCXplorer*.[8, 9]

5.3 DHPLC measurements of Soai aldehyde TMSPyr-CHO

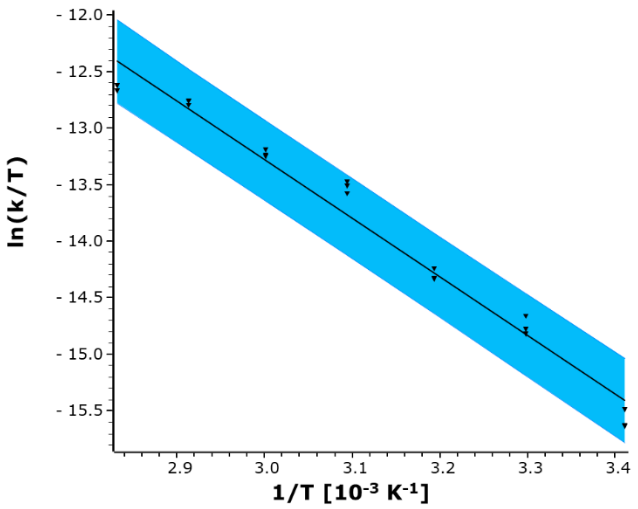

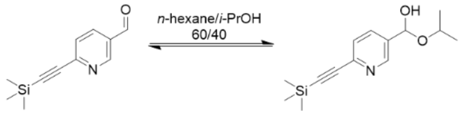

Linear Regression
y = -5.17334E+0003 x + 2.23883E+0000
r = 0.9877
sy = 0.1661

sigma(slope) = 187.6566
sigma(intercept) = 0.5841

ΔH = 43.01 ± 1.56 kJ/mol
ΔS = -173.2 ± 45.2 J/(K mol)
ΔG(298.15K) = 94.64 kJ/mol

Supplementary Figure 76: Eyring plot for the determination of the activation parameters ΔH, ΔS and ΔG of the hemiacetal formation with isopropanol obtained from the DHPLC experiment with the Soai aldehyde TMSPyr-CHO. The upper and lower curves represent the error bands of the linear regression with a level of confidence of 95%.

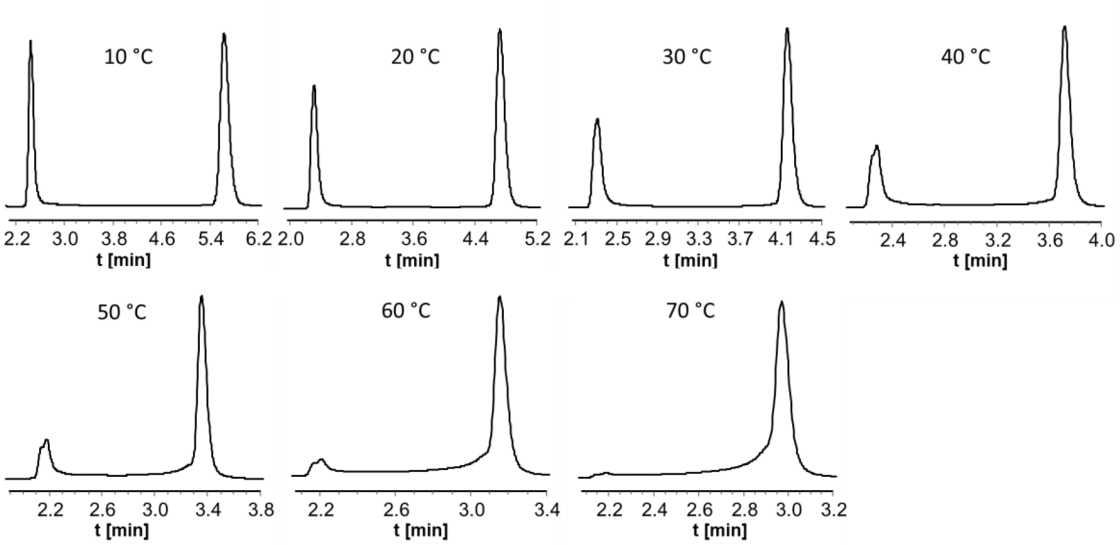

Supplementary Figure 77: Chromatographic profiles obtained from temperature dependent DHPLC measurements of the pyridine based aldehyde **TMSPyr-CHO.**

5.4 DHPLC measurements of Soai aldehyde TMSPym-CHO

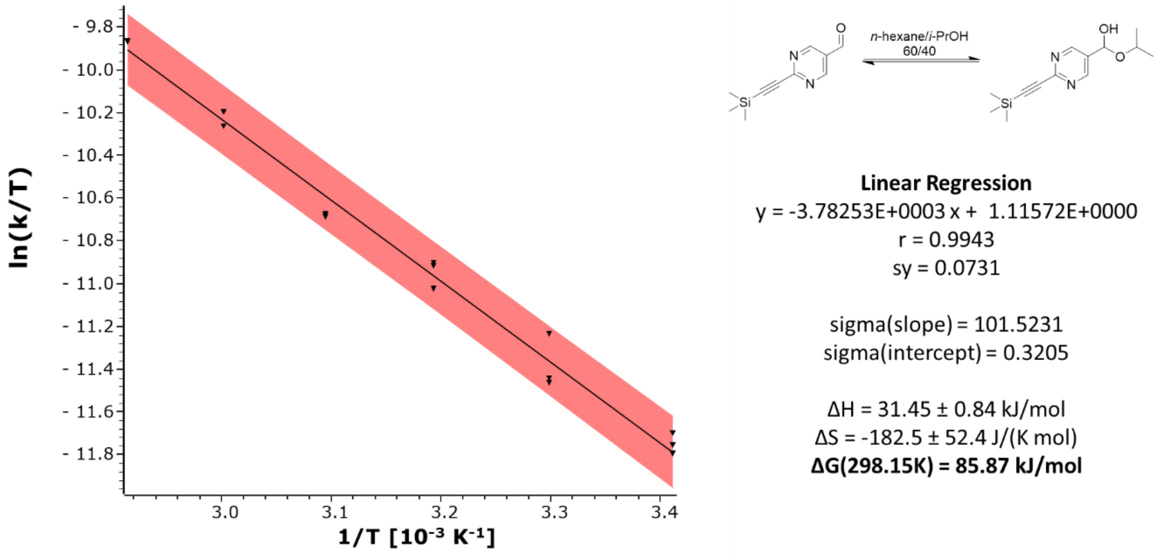

Supplementary Figure 78: Eyring plot for the determination of the activation parameters ΔH, ΔS and ΔG of the hemiacetal formation with isopropanol obtained from the DHPLC experiment with the Soai aldehyde **TMSPym-CHO**. The upper and lower curves represent the error bands of the linear regression with a level of confidence of 95%.

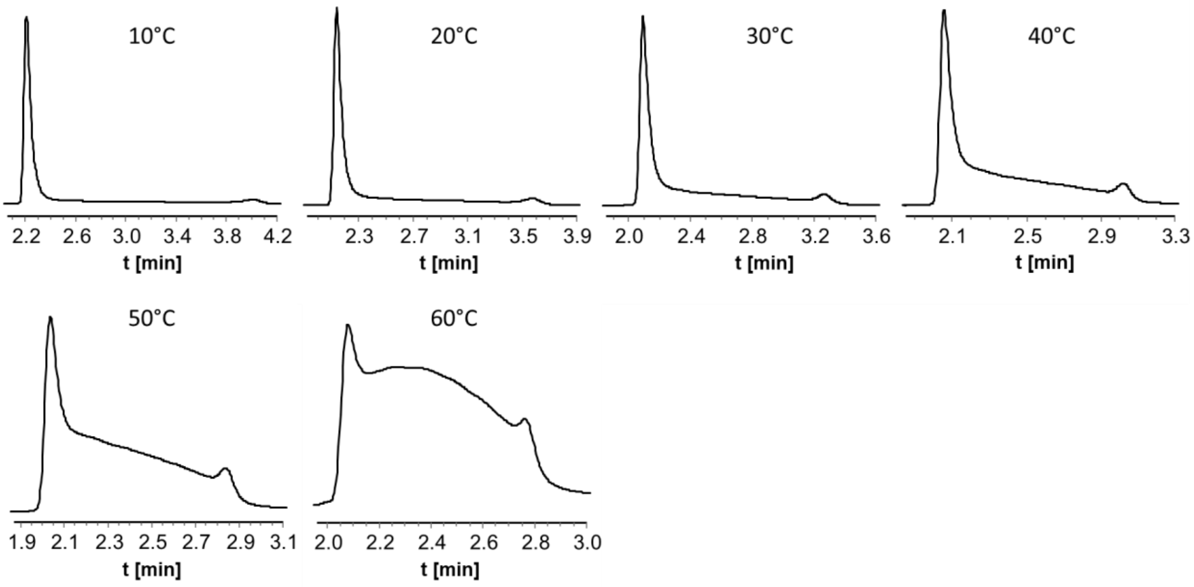

Supplementary Figure 79: Chromatographic profiles obtained from temperature dependent DHPLC measurements of the pyridine based aldehyde **TMSPym-CHO**.

5.5 DHPLC measurements of Soai aldehyde AdPyr-CHO

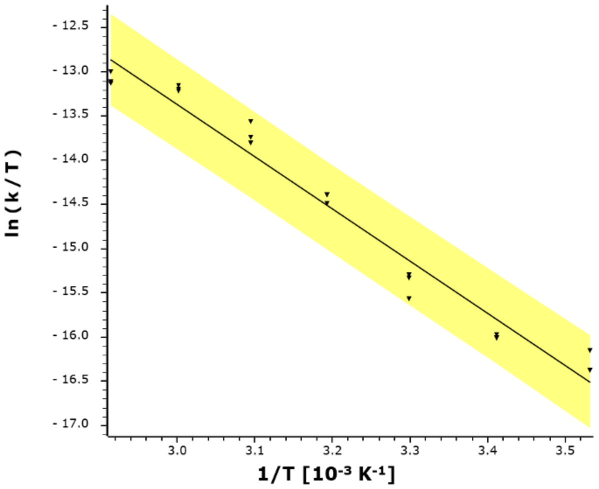

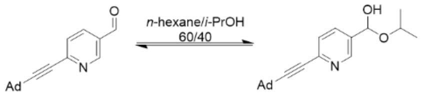

Linear Regression

y = -5.89151E+0003 x + 4.30560E+0000

r = 0.9839

sy = 0.2313

sigma(slope) = 245.3855

sigma(intercept) = 0.7884

ΔH = 48.98 ± 2.04 kJ/mol

ΔS = -156.0 ± 28.6 J/(K mol)

ΔG(298.15K) = 95.49 kJ/mol

Supplementary Figure 80: Eyring plot for the determination of the activation parameters ΔH, ΔS and ΔG of the hemiacetal formation with isopropanol obtained from the DHPLC experiment with the Soai aldehyde **AdPyr-CHO**. The upper and lower curves represent the error bands of the linear regression with a level of confidence of 95%.

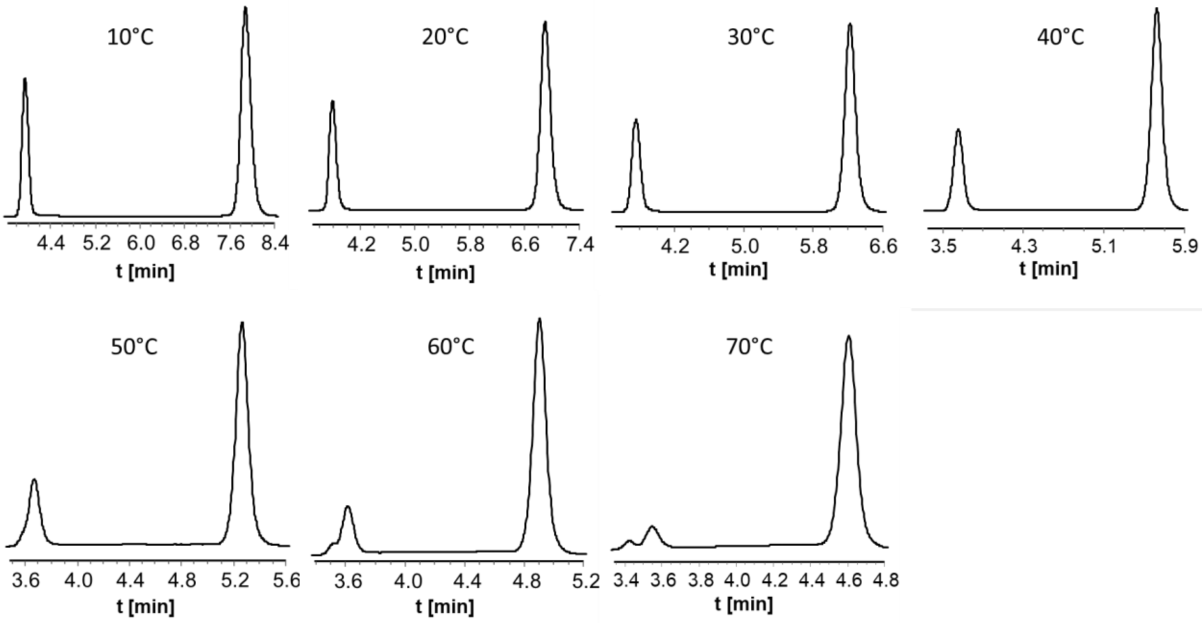

Supplementary Figure 81: Chromatographic profiles obtained from temperature dependent DHPLC measurements of the pyridine based aldehyde **AdPyr-CHO**.

5.6 DHPLC measurements of Soai aldehyde AdPym-CHO

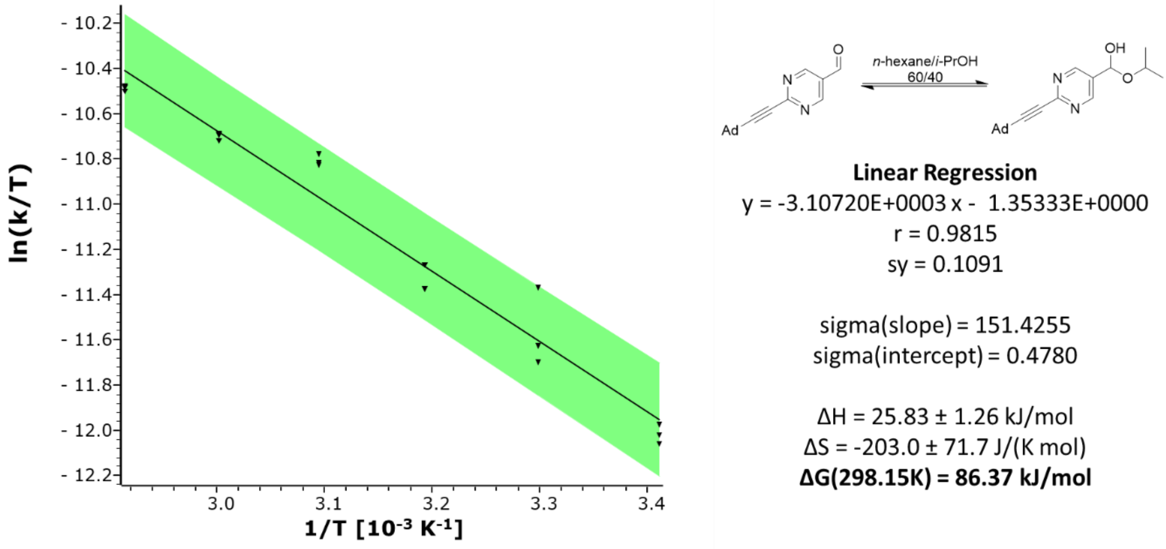

Supplementary Figure 82: Eyring plot for the determination of the activation parameters ΔH, ΔS and ΔG of the hemiacetal formation with isopropanol obtained from the DHPLC experiment with the Soai aldehyde **AdPym-CHO**. The upper and lower curves represent the error bands of the linear regression with a level of confidence of 95%.

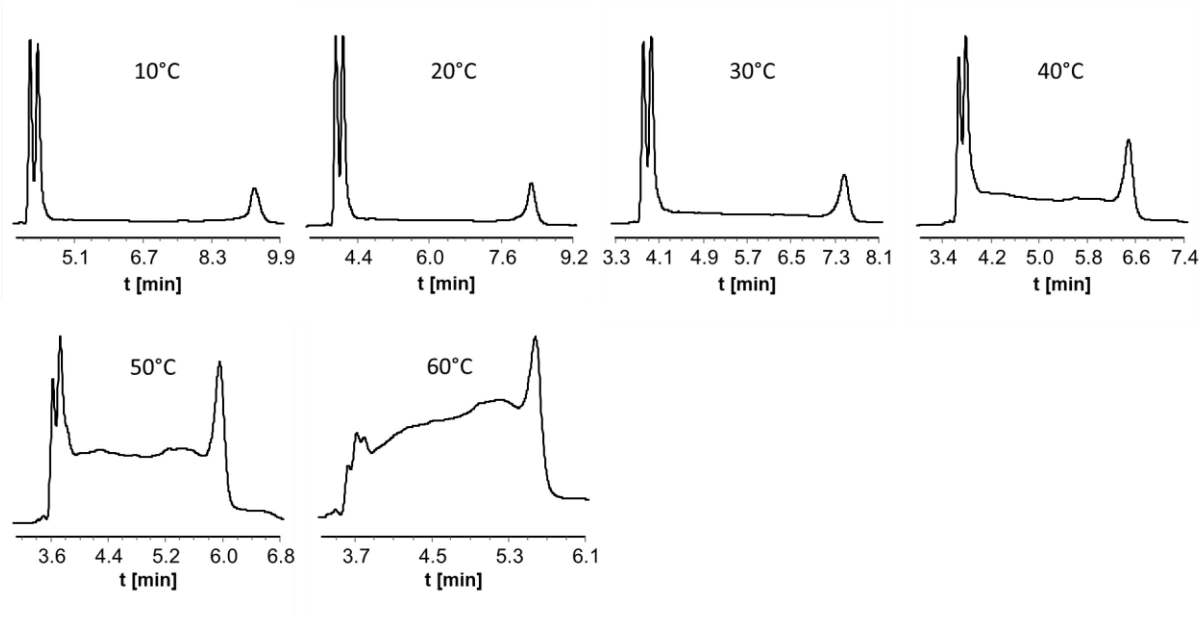

Supplementary Figure 83: Chromatographic profiles obtained from temperature dependent DHPLC measurements of the pyridine based aldehyde **AdPym-CHO**.

6 Kinetic Analysis and Simulation of Reaction Profiles

6.1 Reaction Rates of the Side Reaction

To precisely determine the reaction kinetics of the Soai reaction, it is first necessary to determine the reaction kinetics of the observed side reaction, in this case the reduction of the aldehyde to the achiral benzylic alcohol. Depending on the substrate, this side reaction has a considerable influence on the temporal concentration change of the aldehyde and thus also on the reaction order, as explained in this publication. We have first carried out an evaluation of the side reaction and the resulting reaction using their net reaction equations. It is known from the literature that the reaction order with regard to the aldehyde is between 1.6 and 1.9, without taking the side reaction into account. It must again be expressly pointed out that this side reaction does not occur to any great extent with all substrates, but only with those that lead to a relatively slow reaction.

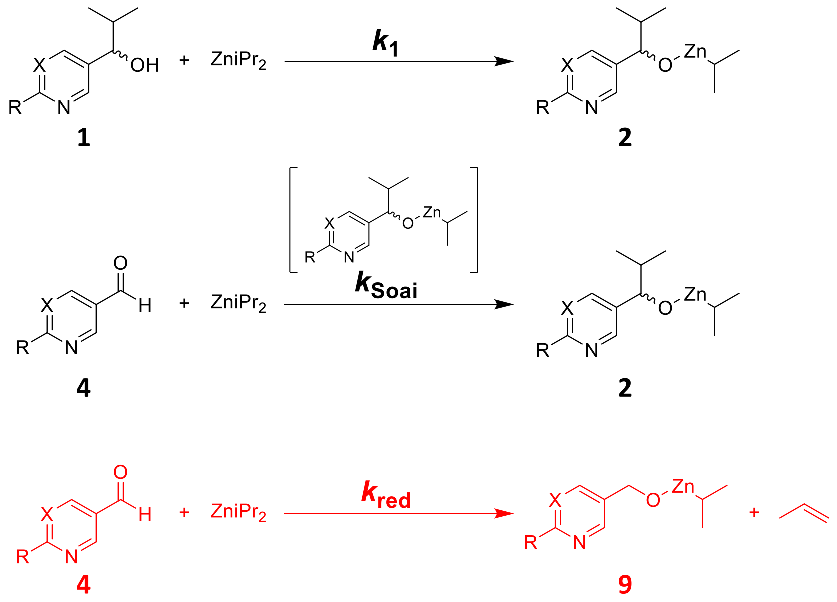

Supplementary Figure 84: Net reaction equations of the Soai reaction and the side reaction, here the reduction of the aldehyde to the corresponding alcohol (red). Pyridyl (X=CH) and pyrimidyl (X=N) derivatives.

The exact analysis of the reaction orders, as described above, has shown that these result in 2 for the aldehyde and 1 for the alcohol for the Soai reaction.

This can also be determined by a model-free kinetic analysis with a variable reaction order in the alsdehyde as follows.

$$\frac{d[1]}{dt} = -k_1[1][ZnR_2] \quad \textbf{(Eq. 5.1.1)}$$

$$\frac{d[2]}{dt} = k_1[1][ZnR_2] + k_{Soai}[4]^x[2] \quad \textbf{(Eq. 5.1.2)}$$

$$\frac{d[4]}{dt} = -k_{Soai}[4]^x[2] - k_{red}[4][Zn] \quad \textbf{(Eq. 5.1.3)}$$

$$\frac{d[9]}{dt} = k_{red}[4][Zn] \quad \textbf{(Eq. 5.1.4)}$$

$$\frac{d[ZnR_2]}{dt} = -k_{Soai}[4]^x[2] - k_{red}[4][Zn] - k_1[1][ZnR_2] \quad \textbf{(Eq. 5.1.5)}$$

This system of ordinary differential equations considers the formation of the alcoholate from the alcohol additive in the first step. In this system the selectivity is neglected, because experimentally only enantiomerically pure/ highly enriched alcohols were used.

Reaction rate k_1 is fixed to $1.5 \cdot 10^2 \pm 7$ $M^{-1}s^{-1}$ for a very rapid process. The equation for the alcoholate formation could be neglected, however for the mass balance of the reaction it should be included. Reaction rates k_{Soai} and k_{red} and the reaction order x were varied in the following ranges (Supplementary Table 1) with the given number of steps (logarithmic scaling for the reaction rates).

Supplementary Table 1: Parameter ranges for variables to determine the reaction kinetics and the reaction order (M = mol·L^{-1}).

Parameter	Low	High	Number of steps
k_1	150		1
k_{Soai}	$1 \cdot 10^{-1}$ $M^{-2}s^{-1}$	$1 \cdot 10^{3}$ $M^{-2}s^{-1}$	500
k_{red}	$1 \cdot 10^{-5}$ $M^{-1}s^{-1}$	1 $M^{-1}s^{-1}$	500
x	1.5	2.5	11

Kinetic reaction profiles were calculated with the given experimental starting parameters (concentrations) and the measured reaction time of one of the kinetic profiles. By this approach 2,750,000 reaction profiles are obtained and compared with the selected experimental profile. The parameter sets of the calculated profiles with a deviation less than 3σ of the experimental profile were selected. This process was then repeated with all other experimental profiles until the parameter range was applicable to all profiles with a deviation within 3σ. The new parameter range was then

split again into 500 x 500 sub-steps. The reaction order of 2 for the aldehyde was already obtained after the first iteration for all reactions investigated.

$$\frac{\mathrm{d}[1]}{\mathrm{d}t} = -k_1[1][\mathrm{ZnR_2}] \quad \textbf{(Eq. 5.1.6)}$$

$$\frac{\mathrm{d}[2]}{\mathrm{d}t} = k_1[1][\mathrm{ZnR_2}] + k_{Soai}[4]^2[2] \quad \textbf{(Eq. 5.1.7)}$$

$$\frac{\mathrm{d}[4]}{\mathrm{d}t} = -k_{Soai}[4]^2[2] - k_{\mathrm{red}}[4][\mathrm{ZnR_2}] \quad \textbf{(Eq. 5.1.8)}$$

$$\frac{\mathrm{d}[9]}{\mathrm{d}t} = k_{\mathrm{red}}[4][\mathrm{ZnR_2}] \quad \textbf{(Eq. 5.1.9)}$$

$$\frac{\mathrm{d}[\mathrm{ZnR_2}]}{\mathrm{d}t} = -k_{Soai}[4]^2[2] - k_{\mathrm{red}}[4][\mathrm{ZnR_2}] - k_1[1][\mathrm{ZnR_2}] \quad \textbf{(Eq. 5.1.10)}$$

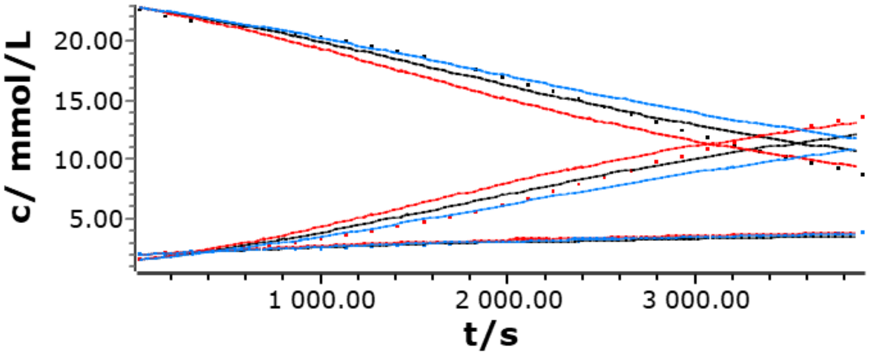

Supplementary Figure 85: Kinetic analysis of the Soai reaction (net reaction) and the side reaction for **TMSPyr-CHO**. First iteration to narrow down the parameter range for k_{Soai} and k_{red}, and determine the reaction order for the aldehyde.

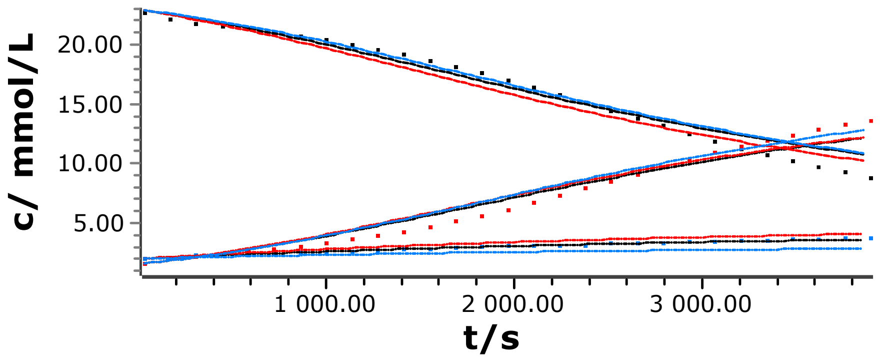

Supplementary Figure 86: Kinetic analysis of the Soai reaction (net reaction) and the side reaction for **TMSPyr-CHO**. Second iteration to refine the parameter range for k_{Soai} and k_{red}.

The reaction rates of the reducing side reaction k_{red} for all substrates obtained by this procedure are summarized in Supplementary Table 2.

Supplementary Table 2: Reaction rates k_{red} of the side reaction.

**Substrate**	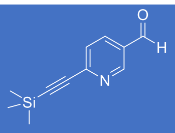	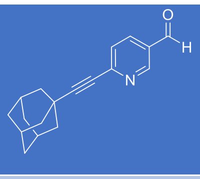	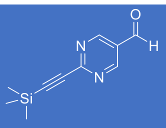	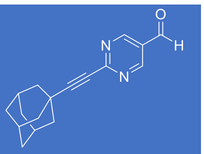
**Reaction rate k_{red}**	$1.21 \cdot 10^{-3}$ $M^{-2}s^{-1}$	$1.93 \cdot 10^{-3}$ $M^{-2}s^{-1}$	$9.60 \cdot 10^{-3}$ $M^{-2}s^{-1}$	$1.88 \cdot 10^{-2}$ $M^{-2}s^{-1}$

6.2 Mechanistic Model and Algorithm for the Kinetic Analysis of the Soai Reaction

Based on the experimentally identified intermediates and reaction traces obtained in situ high-resolution mass spectrometric (HRMS) reaction profiling the following mechanism is proposed.

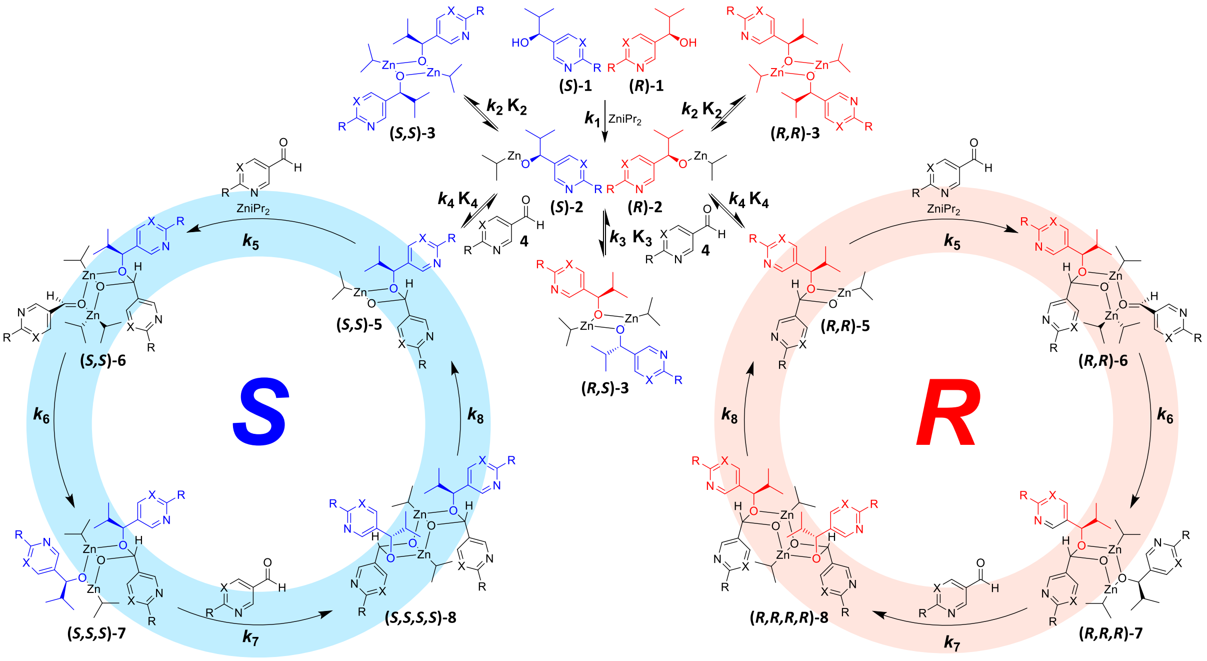

Supplementary Figure 87: Proposed reaction mechanism of the Soai reaction considering the *R*- and *S*-pathway of autocatalysis. Pyridyl (X=CH) and pyrimidyl (X=N) derivatives.

Based on this proposed mechanism the following system of ordinary differential equations (ODEs) was derived to describe the reaction kinetics of the Soai reaction. In addition, the side reaction (reduction) of the aldehyde is considered as an additional ODE and has been added to the corresponding differential equations for the conversion of the aldehyde and diisopropyl zinc. This system is numerically solved by a self-adaptive 4th order Runge-Kutta algorithm[10] programmed in Object Pascal (compiler Embarcadero Delphi XE 7). The program Soai 7 plus (plus: addition of the side reaction) has a graphical user interface to input single data sets, to graphically plot calculation results and an interface for the calculation of large data sets with definable ranges and steps of the reaction rate constants k_n and equilibrium constants K_n. Typically, datasets of 1,953,125 reaction profiles for given experimental parameters were generated and compared with the selected experimental profiles of (*R*)-1, (*S*)-1 and 4 to refine the kinetic parameters and thus the rate constants for the respective partial steps were determined. The parameter sets of the calculated profiles with a deviation less than 3σ of the experimental profile were selected and the experimental parameters adjusted to the next experimental conditions. This process was then repeated with all other experimental profiles until the parameter range was applicable to all profiles with a deviation within 3σ. For the refinement of the kinetic parameters, this process was repeated 3 times (sets A,

B, C, and D). In total around 20,000,000 reaction profiles were calculated for each experimental dataset. The remaining reaction profiles with a deviation within 3σ were statistically evaluated to obtain the mean value and the standard deviation of the reaction rates and equilibrium constants.

In the following the ODEs describing the Soai reaction network are summarized.

Differential equations to describe the time-dependent change of the concentration of the alcohol **1**:

$$\frac{\mathrm{d}[(R)-1]}{\mathrm{d}t} = -k_1[(R)-1][\mathrm{ZnR_2}]$$ **(Eq. 5.2.1)**

$$\frac{\mathrm{d}[(S)-1]}{\mathrm{d}t} = -k_1[(S)-1][\mathrm{ZnR_2}]$$ **(Eq. 5.2.2)**

Differential equation to describe the time-dependent change of the concentration of the dialkyl zinc reagent:

$$\frac{\mathrm{d}[\mathrm{ZnR_2}]}{\mathrm{d}t} = -k_1[(R)-1][\mathrm{ZnR_2}] - k_1[(S)-1][\mathrm{ZnR_2}] - k_5[(R,R)-5][\mathrm{ZnR_2}][4] - k_5[(S,S)-5][\mathrm{ZnR_2}][4] - k_{\mathrm{red}}[4][\mathrm{ZnR_2}]$$ **(Eq. 5.2.3)**

Differential equations to describe the time-dependent change of the concentration of the zinc alcoholates **2**:

$$\frac{\mathrm{d}[(R)-2]}{\mathrm{d}t} = k_1[(R)-1][\mathrm{ZnR_2}] - 2k_2[(R)-2]^2 + 2k_{-2}[(R,R)-3] - k_3[(R)-2][(S)-2] + k_{-3}[(R,S)-3] - k_4[4][(R)-2] + k_{-4}[(R,R)-5]$$ **(Eq. 5.2.4)**

$$\frac{\mathrm{d}[(S)-2]}{\mathrm{d}t} = k_1[(S)-1][\mathrm{ZnR_2}] - 2k_2[(S)-2]^2 + 2k_{-2}[(S,S)-3] - k_3[(R)-2][(S)-2] + k_{-3}[(R,S)-3] - k_4[4][(S)-2] + k_{-4}[(S,S)-5]$$ **(Eq. 5.2.5)**

Differential equations to describe the time-dependent change of the concentration of the homochiral and heterochiral dimeric zinc alcoholates **3**. The heterochiral dimeric zinc alcoholate can be dissolved (diss) or precipitated (solid):

$$\frac{\mathrm{d}[(R,R)-3]}{\mathrm{d}t} = k_2[(R)-2]^2 - k_{-2}[(R,R)-3]$$ **(Eq. 5.2.6)**

$$\frac{\mathrm{d}[(S,S)-3]}{\mathrm{d}t} = k_2[(S)-2]^2 - k_{-2}[(S,S)-3] \quad \textbf{(Eq. 5.2.7)}$$

$$\frac{\mathrm{d}[(R,S)-3_{diss}]}{\mathrm{d}t} = k_3[(R)-2][(S)-2] - k_{-3}[(R,S)-3_{diss}] - k_d[(R,S)-3_{diss}] + k_{-d}[(R,S)-3_{solid}] \quad \textbf{(Eq. 5.2.8)}$$

$$\frac{\mathrm{d}[(R,S)-3_{solid}]}{\mathrm{d}t} = k_d[(R,S)-3_{diss}] - k_{-d}[(R,S)-3_{solid}] \quad \textbf{(Eq. 5.2.9)}$$

Differential equation to describe the time-dependent change of the concentration of the aldehyde **4**:

$$\frac{\mathrm{d}[4]}{\mathrm{d}t} = -k_4[(R)-2][4] + k_{-4}[(R,R)-5] - k_4[(S)-2][4] + k_{-4}[(S,S)-5] - k_5[(R,R)-5][4][\mathrm{ZnR_2}] - k_5[(S,S)-5][4][\mathrm{ZnR_2}] - k_7[(R,R,R)-7][4] - k_7[(S,S,S)-7][4] \quad \textbf{(Eq. 5.2.10)}$$

Differential equation to describe the time-dependent change of the concentration of the hemiacetals **5**:

$$\frac{\mathrm{d}[(R,R)-5]}{\mathrm{d}t} = k_4[(R)-2][4] - k_{-4}[(R,R)-5] - k_5[(R,R)-5][4][\mathrm{ZnR_2}] + 2k_8[(R,R,R,R)-8] \quad \textbf{(Eq. 5.2.11)}$$

$$\frac{\mathrm{d}[(S,S)-5]}{\mathrm{d}t} = k_4[(S)-2][4] - k_{-4}[(S,S)-5] - k_5[(S,S)-5][4][\mathrm{ZnR_2}] + 2k_8[(S,S,S,S)-8] \quad \textbf{(Eq. 5.2.12)}$$

Differential equation to describe the time-dependent change of the concentration of the addition of aldehyde and dialkyl zinc to the hemiacetals **5**, forming enantiomers **6**:

$$\frac{\mathrm{d}[(R,R)-6]}{\mathrm{d}t} = k_5[(R,R)-5][4][\mathrm{ZnR_2}] - k_6[(R,R)-6] \quad \textbf{(Eq. 5.2.13)}$$

$$\frac{d[(S,S)-6]}{dt} = k_5[(S,S)-5][4][ZnR_2] - k_6[(S,S)-6] \quad \textbf{(Eq. 5.2.14)}$$

Differential equation to describe the time-dependent change of the insertion of the alkyl residue into the hemiacetal complex **6**, forming **7**:

$$\frac{d[(R,R,R)-7]}{dt} = k_6[(R,R)-6] - k_7[(R,R,R)-7][4] \quad \textbf{(Eq. 5.2.15)}$$

$$\frac{d[(S,S,S)-7]}{dt} = k_6[(S,S)-6] - k_7[(S,S,S)-7][4] \quad \textbf{(Eq. 5.2.16)}$$

Differential equation to describe the time-dependent formation of dimeric hemiacetal **8**:

$$\frac{d[(R,R,R,R)-8]}{dt} = k_7[(R,R,R)-7][4] - k_8[(R,R,R,R)-8] \quad \textbf{(Eq. 5.2.17)}$$

$$\frac{d[(S,S,S,S)-8]}{dt} = k_7[(S,S,S)-7][4] - k_8[(S,S,S,S)-8] \quad \textbf{(Eq. 5.2.18)}$$

Differential equation to describe the time-dependent formation of reduction side product **9**:

$$\frac{d[9]}{dt} = k_{red}[4][ZnR_2] \quad \textbf{(Eq. 5.2.19)}$$

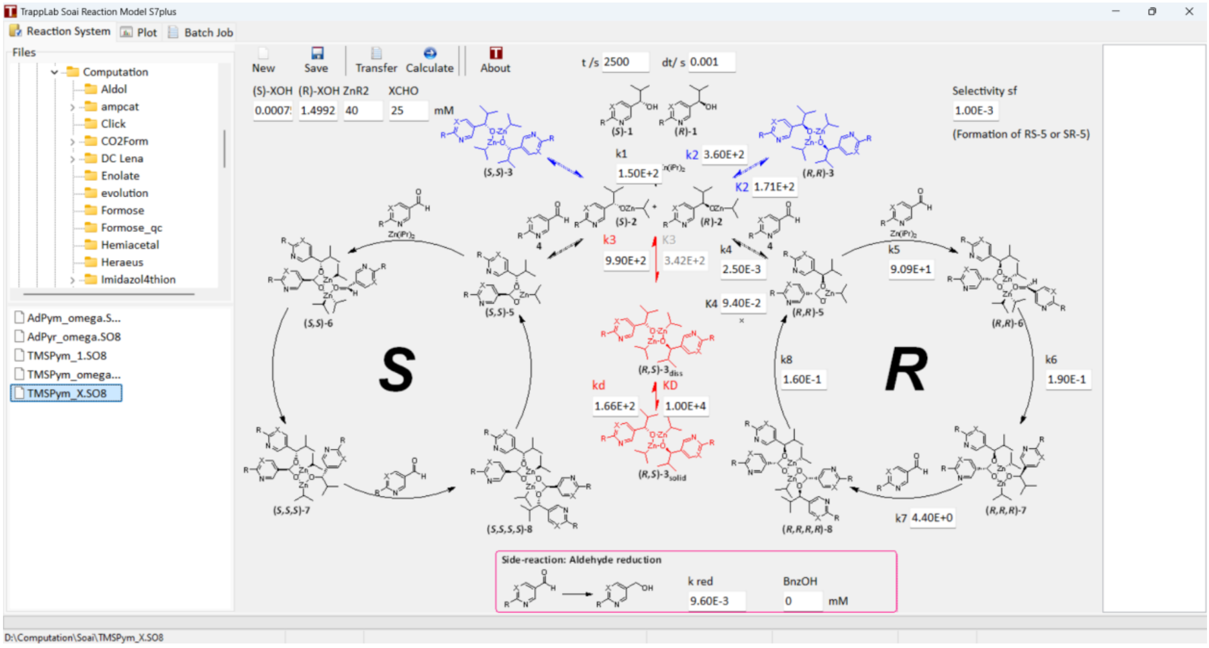

Supplementary Figure 88: User interface of the Soai 7 plus application for single reaction profile calculations.

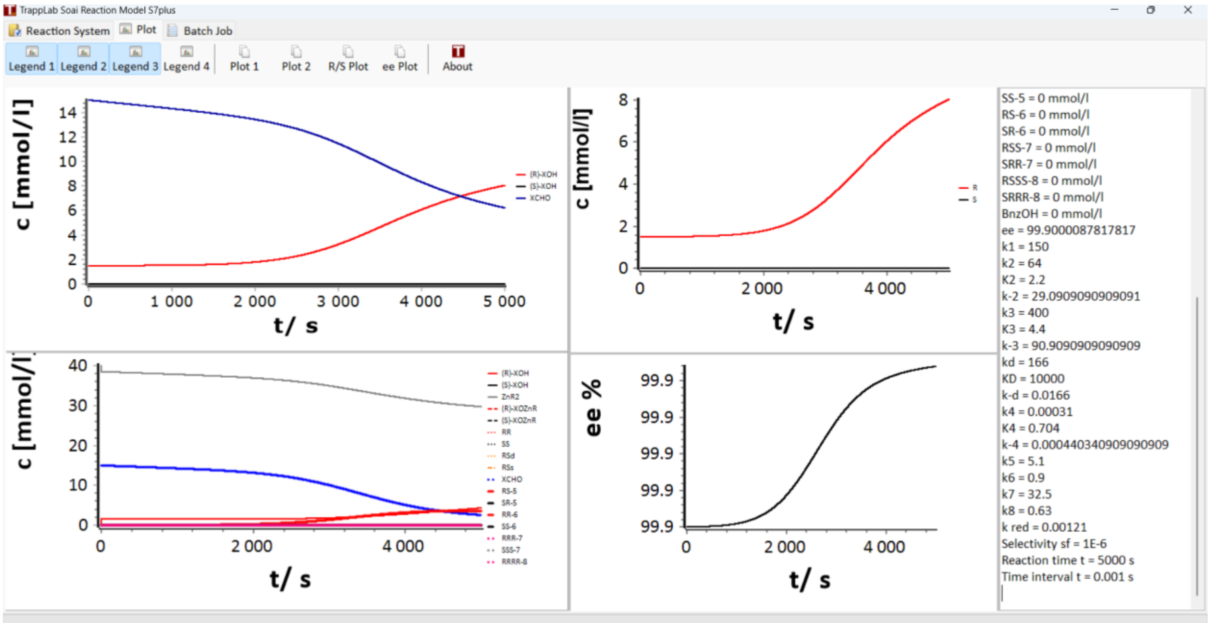

Supplementary Figure 89: Graphical user interface of the Soai 7 plus application for single reaction profile calculations.

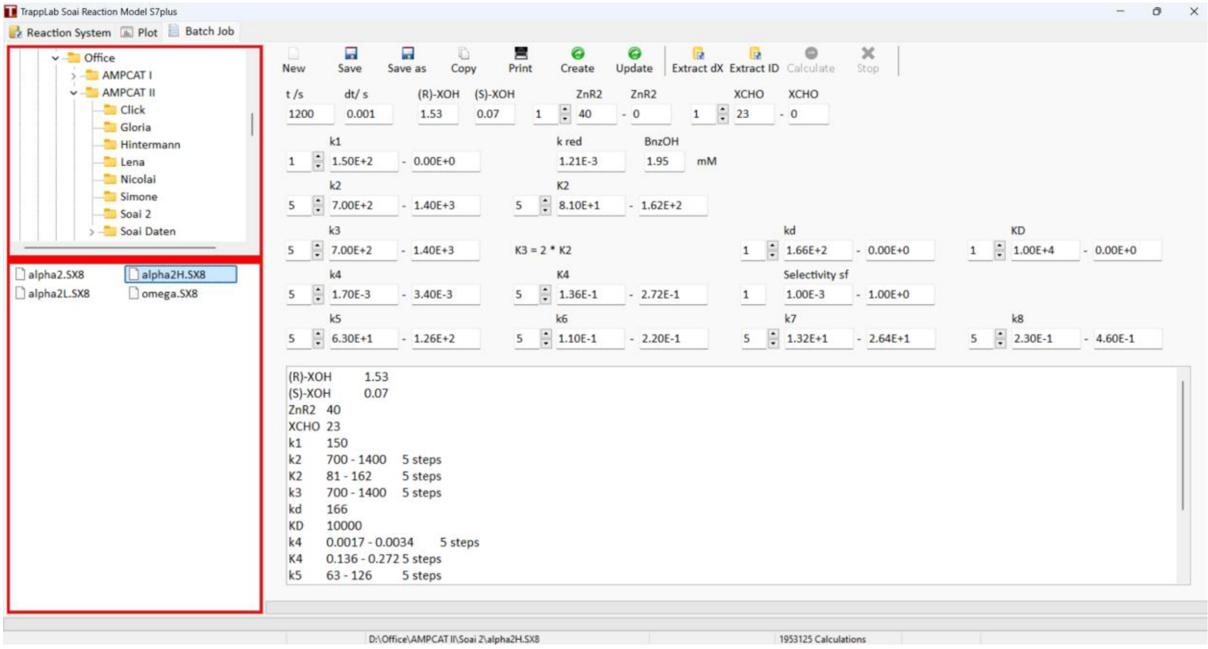

Supplementary Figure 90: User interface of the Soai 7 plus application to perform batch job calculations with systematic variation of reaction parameters.

Finally, a Microsoft Excel data sheet is generated based on this data. The calculation time for a data set of 1.9 mio. profiles is about 1 day on a HP Z4 G4 workstation (Xeon W5-2445, 96 GB RAM, 10 TB SSD).

6.3 Summary of the Reaction Rates Determined from the Experimental Data

Supplementary Table 3: Summarized kinetic data for the four aldehydes (**TMSPyr-CHO, Ad-Pyr-CHO**, **TMSPym-CHO**, **AdPym-CHO**) in comparison with *tert*-butylacetylene-1-yl)pyrimidyl-5-carbaldehyde (**tBuPym-CHO**).

		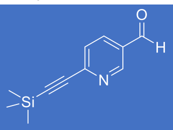	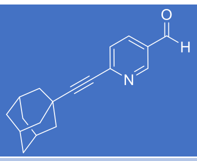	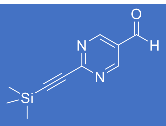	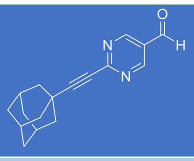	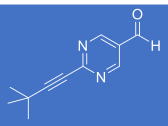
**1**	k_1	$1.5\cdot10^{2}$ $\pm$ 9 $M^{-1}s^{-1}$	$1.5\cdot10^{2}$ $\pm$ 11 $M^{-1}s^{-1}$	$1.5\cdot10^{2}$ $\pm$ 6 $M^{-1}s^{-1}$	$1.5\cdot10^{2}$ $\pm$ 10 $M^{-1}s^{-1}$	$1.5\cdot10^{2}$ $\pm$ 7 $M^{-1}s^{-1}$
**2**	k_2	$6.4\cdot10^{1}$ $\pm$ 11 $M^{-1}s^{-1}$	$4.9\cdot10^{1}$ $\pm$ 12 $M^{-1}s^{-1}$	$3.6\cdot10^{2}$ $\pm$ 6 $M^{-1}s^{-1}$	$2.5\cdot10^{2}$ $\pm$ 37 $M^{-1}s^{-1}$	$7.0\cdot10^{2}$ $\pm$ 32 $M^{-1}s^{-1}$
	k_{-2}	$2.8\cdot10^{1}$ $\pm$ 5 $M^{-1}s^{-1}$	$3.7\cdot10^{1}$ $\pm$ 1.7 s^{-1}	2.1 $\pm$ 0.1 s^{-1}	1.2 $\pm$ 0.3 s^{-1}	8.6 $\pm$ 0.8 s^{-1}
	K_2	2.2 $\pm$ 0.03 M^{-1}	1.35 $\pm$ 0.16 M^{-1}	171 $\pm$ 2 M^{-1}	208 $\pm$ 6 M^{-1}	81 $\pm$ 4 M^{-1}
**3**	k_3	$4.0\cdot10^{2}$ $\pm$ 8 $M^{-1}s^{-1}$	$2.6\cdot10^{2}$ $\pm$ 10 $M^{-1}s^{-1}$	$9.9\cdot10^{2}$ $\pm$ 64 $M^{-1}s^{-1}$	$5.8\cdot10^{2}$ $\pm$ 60 $M^{-1}s^{-1}$	$7.0\cdot10^{2}$ $\pm$ 32 $M^{-1}s^{-1}$
	k_{-3}	90.9 $\pm$ 1.7 s^{-1}	97.7 $\pm$ 0.5 s^{-1}	2.9 $\pm$ 0.2 s^{-1}	1.4 $\pm$ 0.1 s^{-1}	4.3 $\pm$ 0.4 s^{-1}
	K_3	4.4 $\pm$ 0.2 M^{-1}	2.7 $\pm$ 0.3 M^{-1}	342 $\pm$ 10 M^{-1}	416 $\pm$ 21 M^{-1}	162 $\pm$ 8 M^{-1}
**4**	k_4	$3.1\cdot10^{-4}$ $\pm$ $0.2\cdot10^{-4}$ $M^{-1}s^{-1}$	$3.5\cdot10^{-4}$ $\pm$ $0.5\cdot10^{-4}$ $M^{-1}s^{-1}$	$2.5\cdot10^{-3}$ $\pm$ $3.2\cdot10^{-5}$ $M^{-1}s^{-1}$	$1.4\cdot10^{-3}$ $\pm$ $5.9\cdot10^{-5}$ $M^{-1}s^{-1}$	$1.7\cdot10^{-3}$ $\pm$ $1.2\cdot10^{-4}$ $M^{-1}s^{-1}$
	k_{-4}	$4.4\cdot10^{-4}$ $\pm$ $0.3\cdot10^{-4}$ s^{-1}	$3.3\cdot10^{-4}$ $\pm$ $0.7\cdot10^{-4}$ s^{-1}	$2.6\cdot10^{-2}$ $\pm$ $3.5\cdot10^{-4}$ s^{-1}	$5.6\cdot10^{-3}$ $\pm$ $2.1\cdot10^{-4}$ s^{-1}	$1.3\cdot10^{-2}$ $\pm$ $1.0\cdot10^{-3}$ s^{-1}
	K_4	0.704 $\pm$ 0.002 M^{-1}	1.055 $\pm$ 0.006 M^{-1}	0.094 $\pm$ 0.001 M^{-1}	0.258 $\pm$ 0.004 M^{-1}	0.136 $\pm$ 0.001 M^{-1}
**5**	k_5	5.1 $\pm$ 0.1 $M^{-2}s^{-1}$	4.2 $\pm$ 0.2 $M^{-2}s^{-1}$	90.9 $\pm$ 1.0 $M^{-2}s^{-1}$	150.9 $\pm$ 0.9 $M^{-2}s^{-1}$	63.0 $\pm$ 5.0 $M^{-2}s^{-1}$
**6**	k_6	0.90 $\pm$ 0.02 s^{-1}	0.22 $\pm$ 0.01 s^{-1}	0.19 $\pm$ 0.02 s^{-1}	$6.0\cdot10^{-2}$ $\pm$ $0.6\cdot10^{-3}$ s^{-1}	0.11 $\pm$ 0.01 s^{-1}
**7**	k_7	32.5 $\pm$ 1.3 $M^{-1}s^{-1}$	127.8 $\pm$ 14.6 $M^{-1}s^{-1}$	4.4 $\pm$ 0.1 $M^{-1}s^{-1}$	9.4 $\pm$ 0.1 $M^{-1}s^{-1}$	13.2 $\pm$ 0.2 $M^{-1}s^{-1}$
**8**	k_8	$6.3\cdot10^{-1}$ $\pm$ $0.1\cdot10^{-1}$ s^{-1}	$1.8\cdot10^{-1}$ $\pm$ $0.4\cdot10^{-1}$ s^{-1}	$1.6\cdot10^{-1}$ $\pm$ $0.3\cdot10^{-1}$ s^{-1}	$5.6\cdot10^{-2}$ $\pm$ $0.6\cdot10^{-2}$ s^{-1}	$2.3\cdot10^{-1}$ $\pm$ $0.2\cdot10^{-1}$ s^{-1}
	k_{red}	$1.21\cdot10^{-3}$ $M^{-1}s^{-1}$	$1.93\cdot10^{-3}$ $M^{-1}s^{-1}$	$9.60\cdot10^{-3}$ $M^{-1}s^{-1}$	$1.88\cdot10^{-2}$ $M^{-1}s^{-1}$	

6.4 Simulation of concentration-time profiles

6.4.1 TMSPyr-CHO/TMSPyr-OH System

Supplementary Table 4: Simulation Parameters for the **TMSPyr-CHO/TMSPyr-OH** System.

	TMSPyrCHO [mmol/L]	(*R*)-TMSPyrOH [mmol/L]	(*S*)-TMSPyrOH [mmol/L]	ee [%]	er	$ZniPr_2$ [mmol/L]	t [s]
**1**	15	1.49925	0.00075	99.9	1999	40	5000
**2**	20	1.49925	0.00075	99.9	1999	40	5000
**3**	25	1.49925	0.00075	99.9	1999	40	5000
**4**	30	1.49925	0.00075	99.9	1999	40	5000
**5**	35	1.49925	0.00075	99.9	1999	40	5000
**6**	25	0.749625	0.000375	99.9	1999	40	5000
**7**	25	1.49925	0.00075	99.9	1999	40	5000
**8**	25	1.7991	0.0009	99.9	1999	40	5000
**9**	25	2.9985	0.0015	99.9	1999	40	5000
**10**	25	1.49925	0.00075	99.9	1999	25	5000
**11**	25	1.49925	0.00075	99.9	1999	50	5000
**12**	25	1.49925	0.00075	99.9	1999	75	5000
**13**	25	1.49925	0.00075	99.9	1999	100	5000

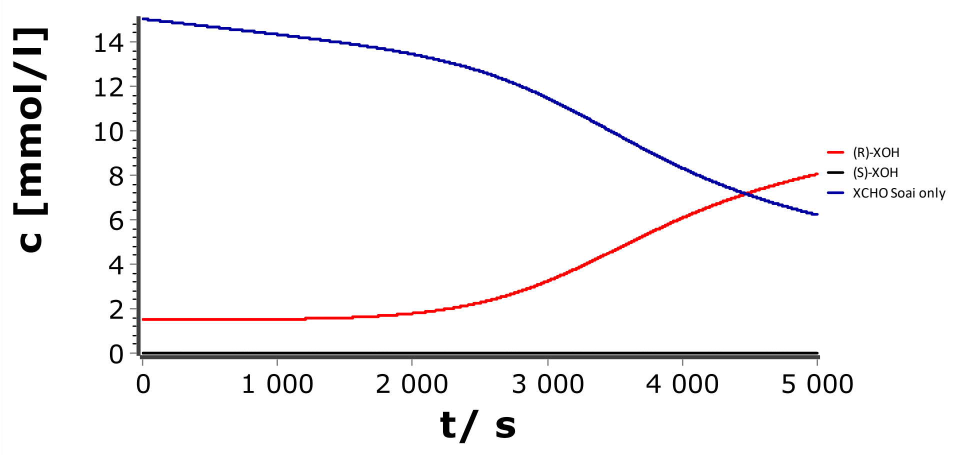

Supplementary Figure 91: Simulated concentration-time profile of the Soai reaction (15 mM 6-((Trimethylsilyl)ethynyl)nicotinaldehyde **TMSPyr-CHO**, 1.5 mM 2-Methyl-(6-((trimethylsilyl)ethynyl)-pyridine-3-yl)propanol **TMSPyr-OH** (*ee* > 99.9%) and 40 mM iPr_2Zn).

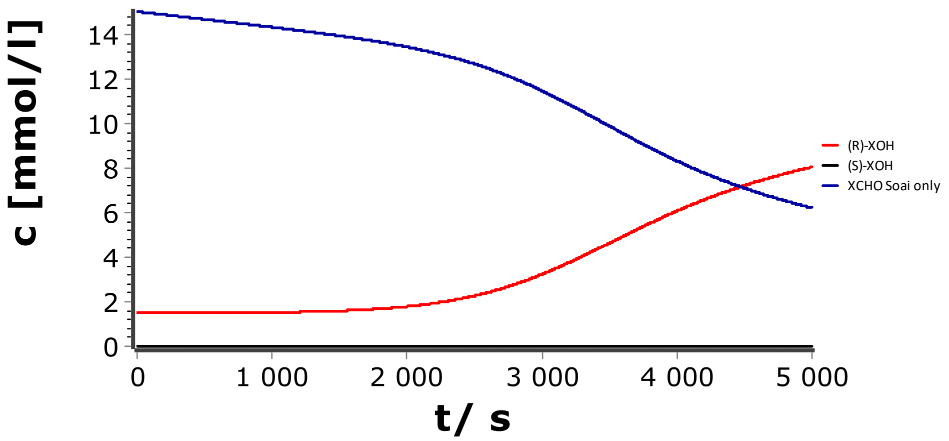

Supplementary Figure 92: Simulated concentration-time profile of the *Soai* reaction (20 mM 6-((Trimethylsilyl)ethynyl)nicotinaldehyde **TMSPyr-CHO**, 1.5 mM 2-Methyl-(6-((trimethylsilyl)ethynyl)-pyridine-3-yl)propanol **TMSPyr-OH** (*ee* > 99.9%) and 40 mM *i*Pr_2Zn).

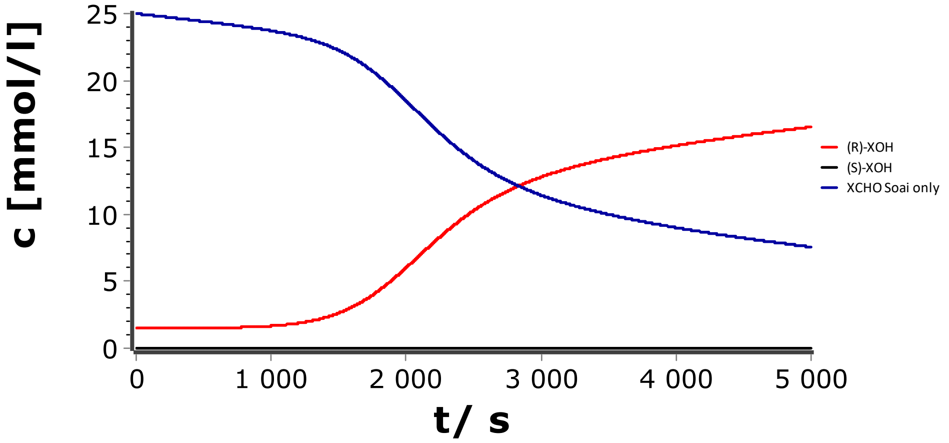

Supplementary Figure 93: Simulated concentration-time profile of the Soai reaction (25 mM 6-((Trimethylsilyl)ethynyl)nicotinaldehyde **TMSPyr-CHO**, 1.5 mM 2-Methyl-(6-((trimethylsilyl)ethynyl)-pyridine-3-yl)propanol **TMSPyr-OH** (*ee* > 99.9%) and 40 mM *i*Pr_2Zn).

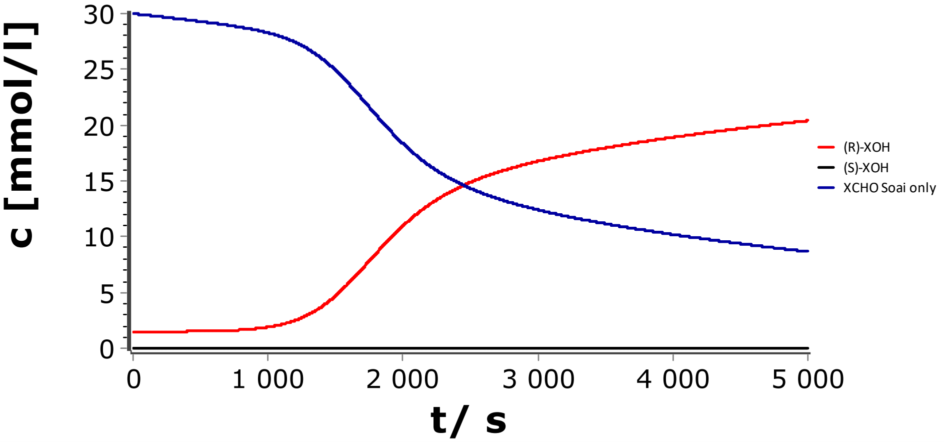

Supplementary Figure 94: Simulated concentration-time profile of the Soai reaction (30 mM 6-((Trimethylsilyl)ethynyl)nicotinaldehyde **TMSPyr-CHO**, 1.5 mM 2-Methyl-(6-((trimethylsilyl)ethynyl)-pyridine-3-yl)propanol **TMSPyr-OH** (*ee* > 99.9%) and 40 mM $i\text{Pr}_2\text{Zn}$).

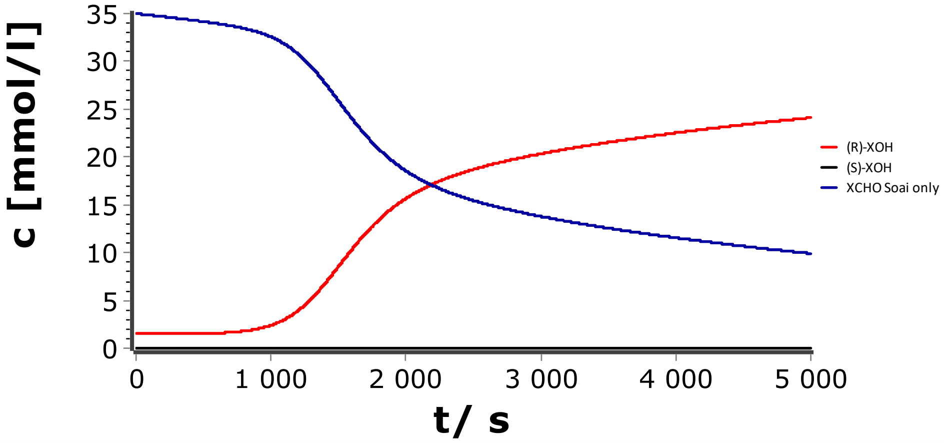

Supplementary Figure 95: Simulated concentration-time profile of the Soai reaction (35 mM 6-((Trimethylsilyl)ethynyl)nicotinaldehyde **TMSPyr-CHO**, 1.5 mM 2-Methyl-(6-((trimethylsilyl)ethynyl)-pyridine-3-yl)propanol **TMSPyr-OH** (*ee* > 99.9%) and 40 mM $i\text{Pr}_2\text{Zn}$).

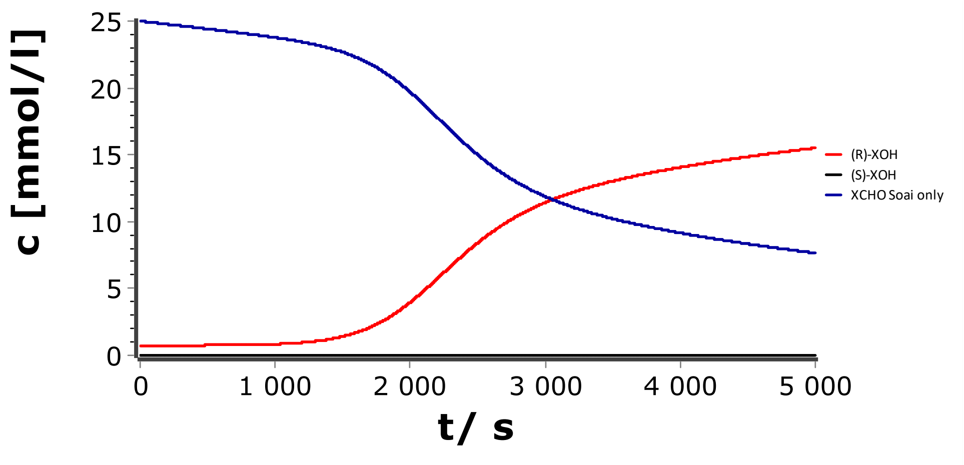

Supplementary Figure 96: Simulated concentration-time profile of the Soai reaction (25 mM 6-((Trimethylsilyl)ethynyl)nicotinaldehyde **TMSPyr-CHO**, 0.75 mM 2-Methyl-(6-((trimethylsilyl)ethynyl)-pyridine-3-yl)propanol **TMSPyr-OH** (*ee* > 99.9%) and 40 mM *i*Pr_2Zn).

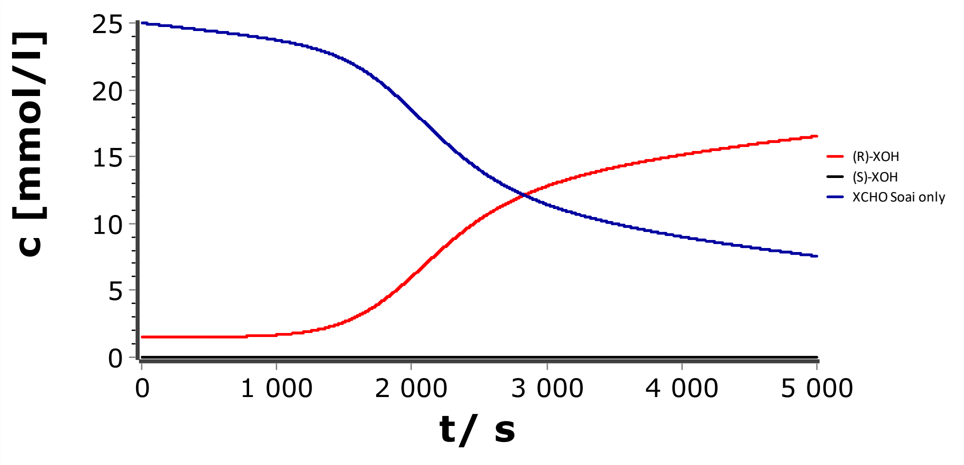

Supplementary Figure 97: Simulated concentration-time profile of the Soai reaction (25 mM 6-((Trimethylsilyl)ethynyl)nicotinaldehyde **TMSPyr-CHO**, 1.5 mM 2-Methyl-(6-((trimethylsilyl)ethynyl)-pyridine-3-yl)propanol **TMSPyr-OH** (*ee* > 99.9%) and 40 mM *i*Pr_2Zn).

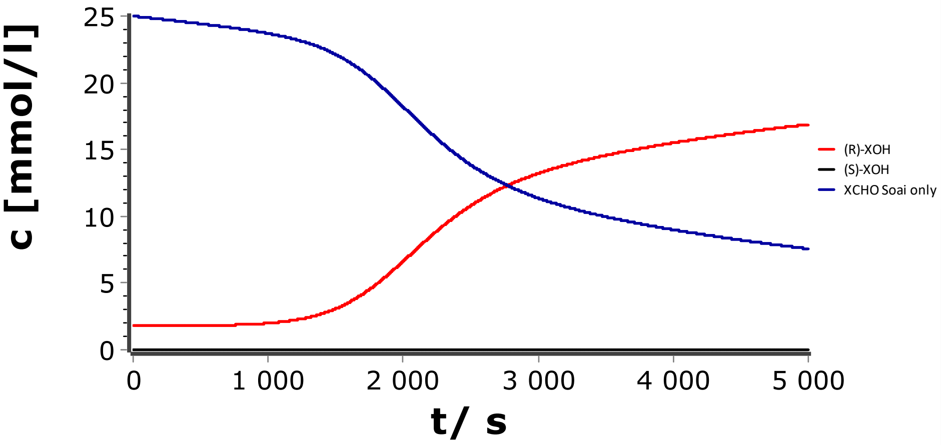

Supplementary Figure 98: Simulated concentration-time profile of the Soai reaction (25 mM 6-((Trimethylsilyl)ethynyl)nicotinaldehyde **TMSPyr-CHO**, 1.8 mM 2-Methyl-(6-((trimethylsilyl)ethynyl)-pyridine-3-yl)propanol **TMSPyr-OH** (*ee* > 99.9%) and 40 mM *i*Pr_2Zn).

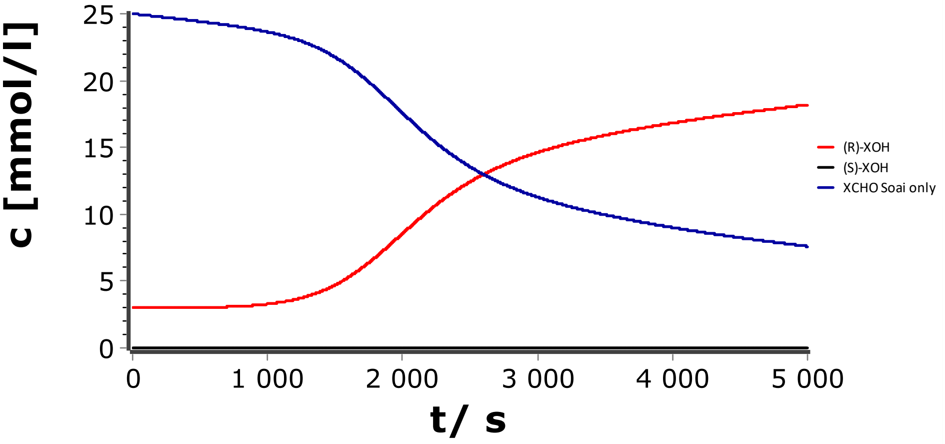

Supplementary Figure 99: Simulated concentration-time profile of the Soai reaction (25 mM 6-((Trimethylsilyl)ethynyl)nicotinaldehyde **TMSPyr-CHO**, 3.0 mM 2-Methyl-(6-((trimethylsilyl)ethynyl)-pyridine-3-yl)propanol **TMSPyr-OH** (*ee* > 99.9%) and 40 mM *i*Pr_2Zn).

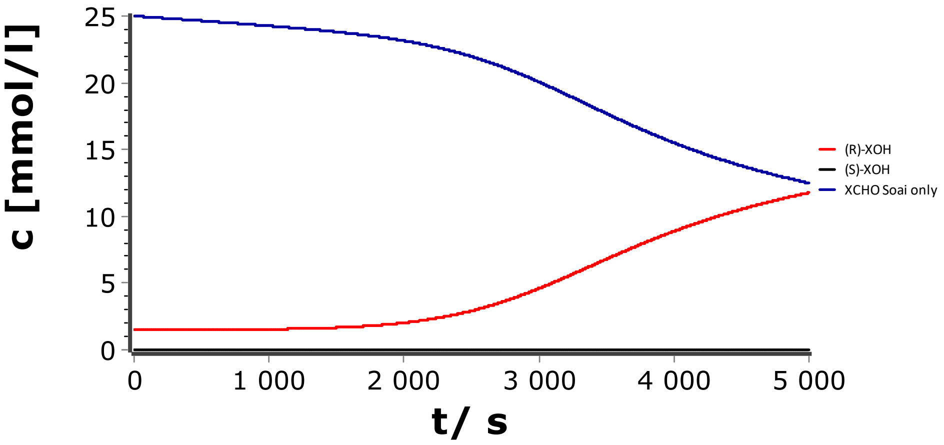

Supplementary Figure 100: Simulated concentration-time profile of the Soai reaction (25 mM 6-((Trimethylsilyl)ethynyl)nicotinaldehyde **TMSPyr-CHO**, 1.5 mM 2-Methyl-(6-((trimethylsilyl)ethynyl)-pyridine-3-yl)propanol **TMSPyr-OH** (*ee* > 99.9%) and 25 mM *i*Pr_2Zn).

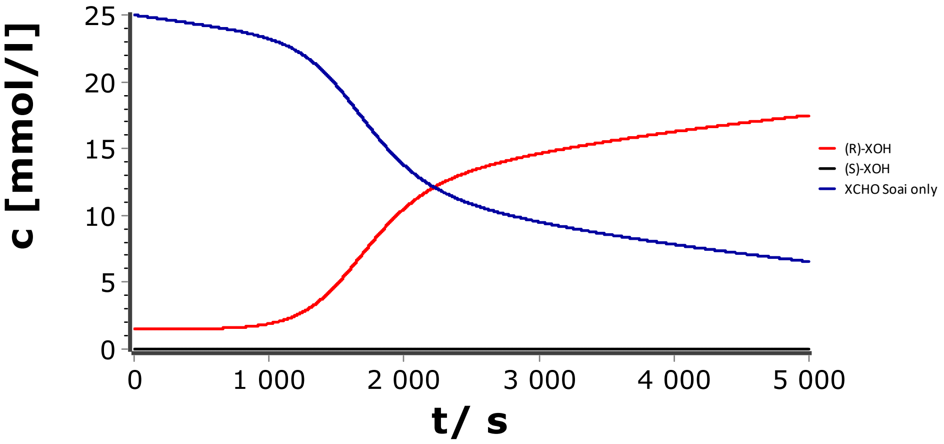

Supplementary Figure 101: Simulated concentration-time profile of the Soai reaction (25 mM 6-((Trimethylsilyl)ethynyl)nicotinaldehyde **TMSPyr-CHO**, 1.5 mM 2-Methyl-(6-((trimethylsilyl)ethynyl)-pyridine-3-yl)propanol **TMSPyr-OH** (*ee* > 99.9%) and 50 mM *i*Pr_2Zn).

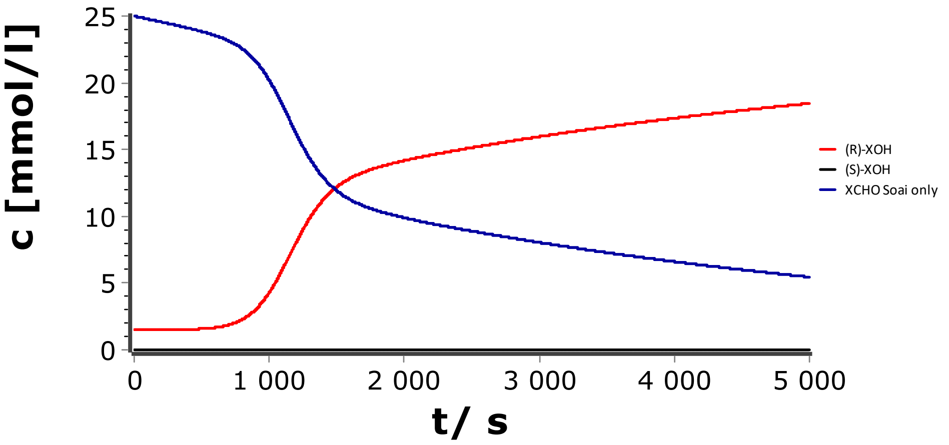

Supplementary Figure 102: Simulated concentration-time profile of the Soai reaction (25 mM 6-((Trimethylsilyl)ethynyl)nicotinaldehyde **TMSPyr-CHO**, 1.5 mM 2-Methyl-(6-((trimethylsilyl)ethynyl)-pyridine-3-yl)propanol **TMSPyr-OH** (*ee* > 99.9%) and 75 mM *i*Pr_2Zn).

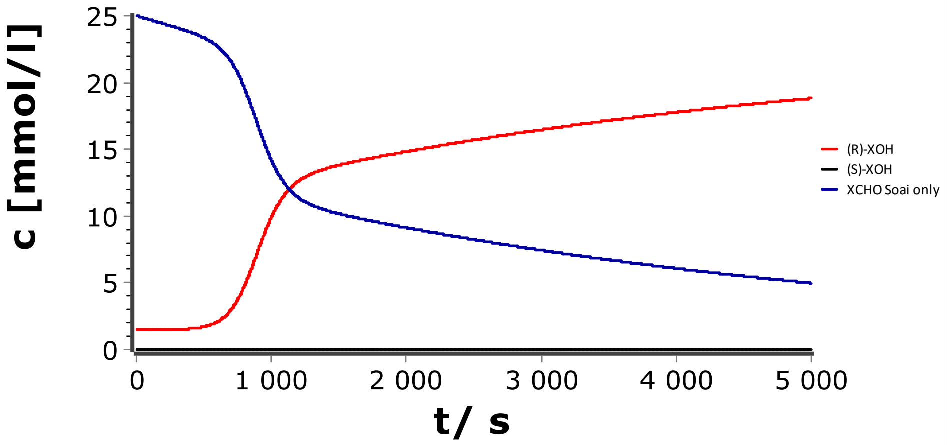

Supplementary Figure 103: Simulated concentration-time profile of the Soai reaction (25 mM 6-((Trimethylsilyl)ethynyl)nicotinaldehyde **TMSPyr-CHO**, 1.5 mM 2-Methyl-(6-((trimethylsilyl)ethynyl)-pyridine-3-yl)propanol **TMSPyr-OH** (*ee* > 99.9%) and 100 mM *i*Pr_2Zn).

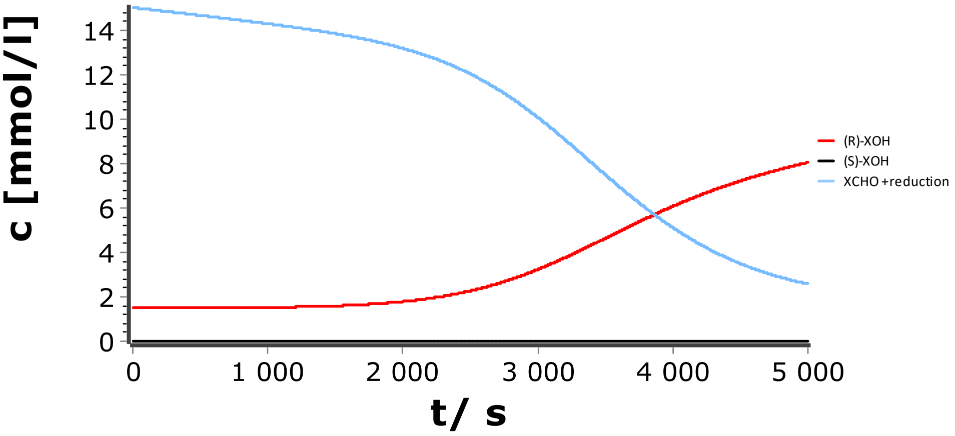

Supplementary Figure 104: Simulated concentration-time profile of the Soai reaction and the reduction side reaction (15 mM 6-((Trimethylsilyl)ethynyl)nicotinaldehyde **TMSPyr-CHO,** 1.5 mM 2-Methyl-(6-((trimethylsilyl)ethynyl)-pyridine-3-yl)propanol **TMSPyr-OH** (*ee* > 99.9%) and 40 mM *i*Pr_2Zn).

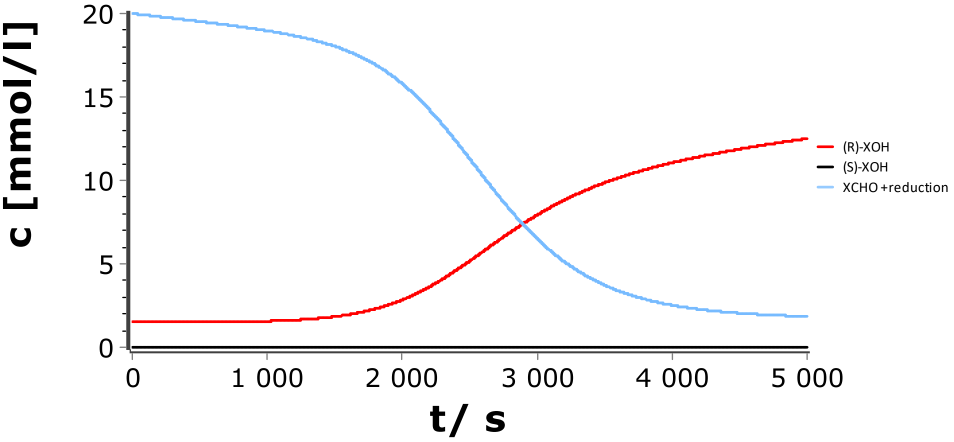

Supplementary Figure 105: Simulated concentration-time profile of the Soai reaction and the reduction side reaction (20 mM 6-((Trimethylsilyl)ethynyl)nicotinaldehyde **TMSPyr-CHO**, 1.5 mM 2-Methyl-(6-((trimethylsilyl)ethynyl)-pyridine-3-yl)propanol **TMSPyr-OH** (*ee* > 99.9%) and 40 mM *i*Pr_2Zn).

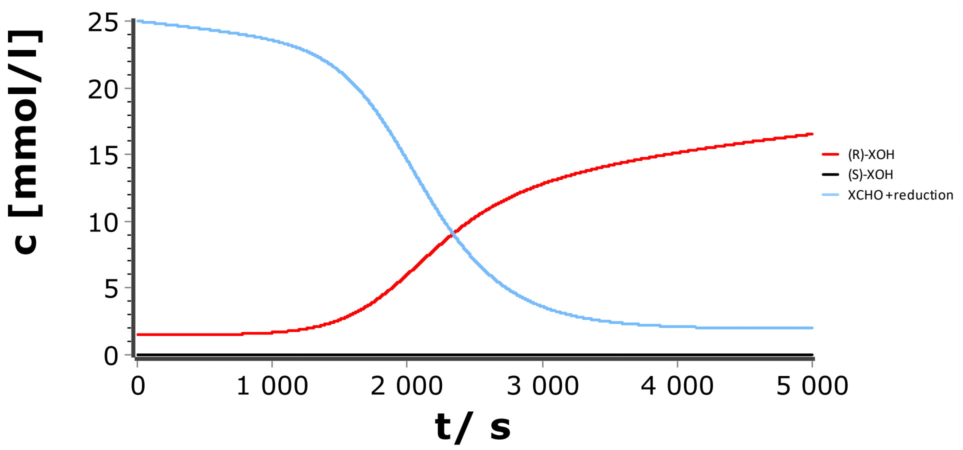

Supplementary Figure 106: Simulated concentration-time profile of the Soai reaction and the reduction side reaction (25 mM 6-((Trimethylsilyl)ethynyl)nicotinaldehyde **TMSPyr-CHO**, 1.5 mM 2-Methyl-(6-((trimethylsilyl)ethynyl)-pyridine-3-yl)propanol **TMSPyr-OH** (*ee* > 99.9%) and 40 mM *i*Pr_2Zn).

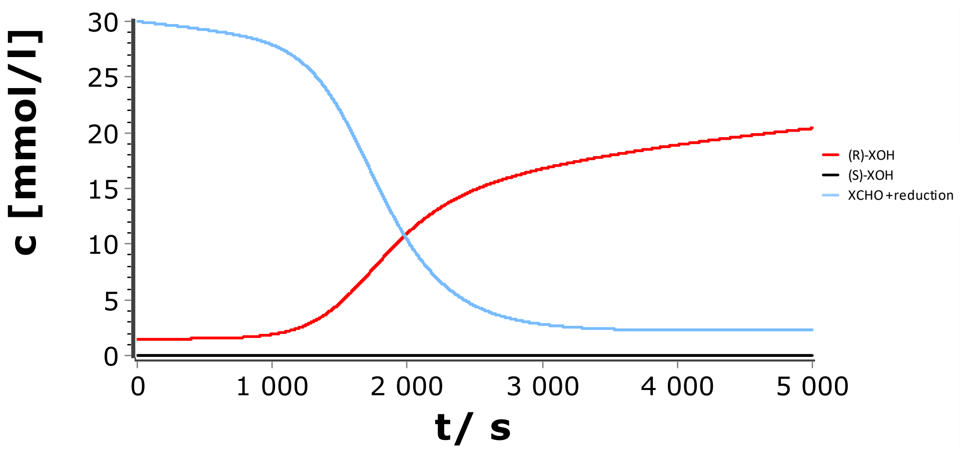

Supplementary Figure 107: Simulated concentration-time profile of the Soai reaction and the reduction side reaction (30 mM 6-((Trimethylsilyl)ethynyl)nicotinaldehyde **TMSPyr-CHO**, 1.5 mM 2-Methyl-(6-((trimethylsilyl)ethynyl)-pyridine-3-yl)propanol **TMSPyr-OH** (*ee* > 99.9%) and 40 mM *i*Pr_2Zn).

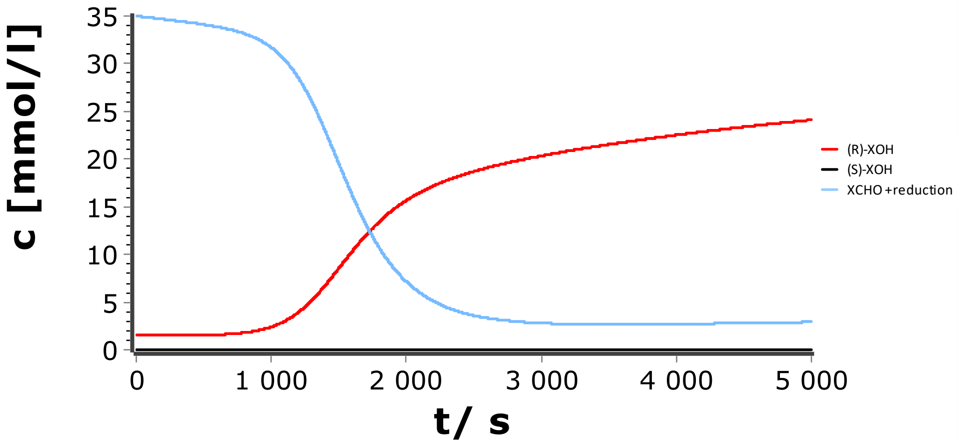

Supplementary Figure 108: Simulated concentration-time profile of the Soai reaction and the reduction side reaction (35 mM 6-((Trimethylsilyl)ethynyl)nicotinaldehyde **TMSPyr-CHO**, 1.5 mM 2-Methyl-(6-((trimethylsilyl)ethynyl)-pyridine-3-yl)propanol **TMSPyr-OH** (*ee* > 99.9%) and 40 mM *i*Pr_2Zn).

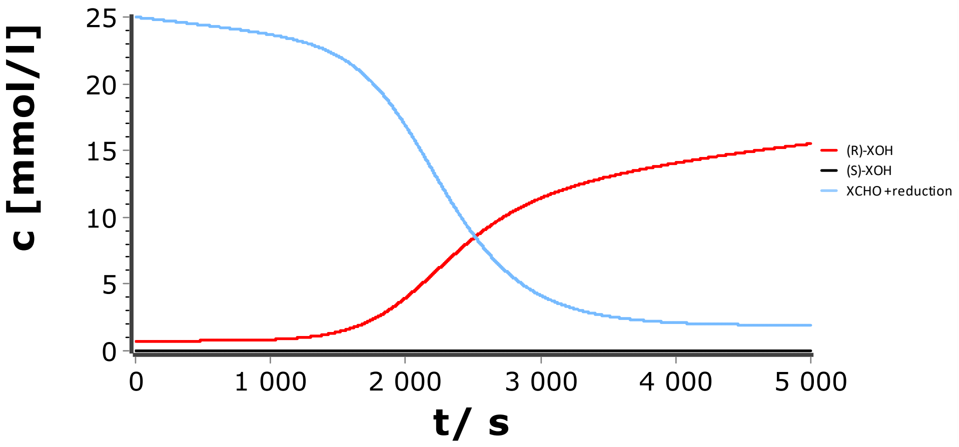

Supplementary Figure 109: Simulated concentration-time profile of the Soai reaction and the reduction side reaction (25 mM 6-((Trimethylsilyl)ethynyl)nicotinaldehyde **TMSPyr-CHO**, 0.75 mM 2-Methyl-(6-((trimethylsilyl)ethynyl)-pyridine-3-yl)propanol **TMSPyr-OH** (*ee* > 99.9%) and 40 mM *i*Pr_2Zn).

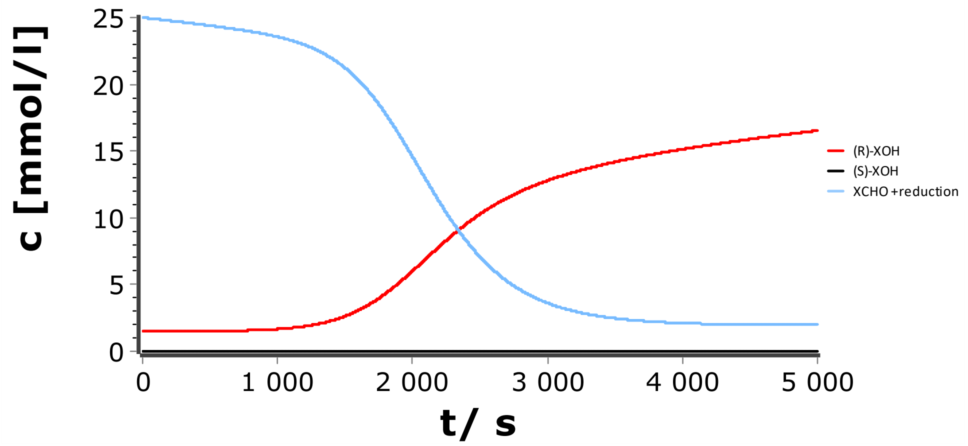

Supplementary Figure 110: Simulated concentration-time profile of the Soai reaction and the reduction side reaction (25 mM 6-((Trimethylsilyl)ethynyl)nicotinaldehyde **TMSPyr-CHO**, 1.5 mM 2-Methyl-(6-((trimethylsilyl)ethynyl)-pyridine-3-yl)propanol **TMSPyr-OH** (*ee* > 99.9%) and 40 mM *i*Pr_2Zn).

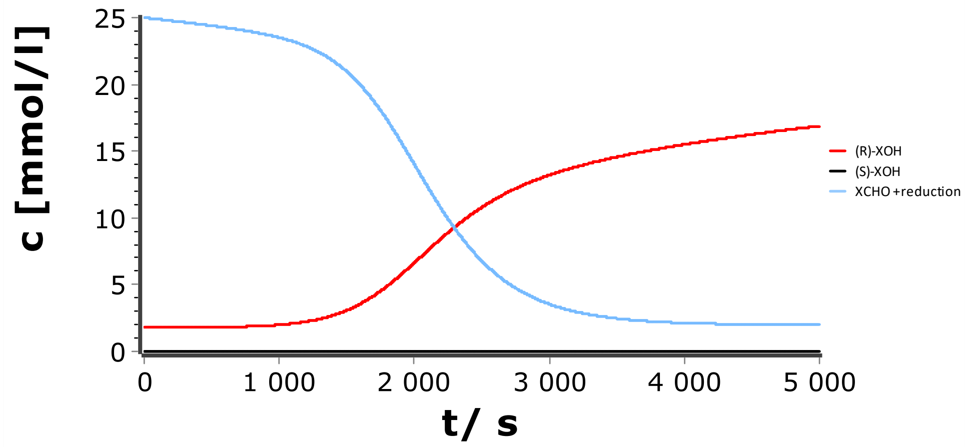

Supplementary Figure 111: Simulated concentration-time profile of the Soai reaction and the reduction side reaction (25 mM 6-((Trimethylsilyl)ethynyl)nicotinaldehyde **TMSPyr-CHO**, 1.8 mM 2-Methyl-(6-((trimethylsilyl)ethynyl)-pyridine-3-yl)propanol **TMSPyr-OH** (*ee* > 99.9%) and 40 mM *i*Pr_2Zn).

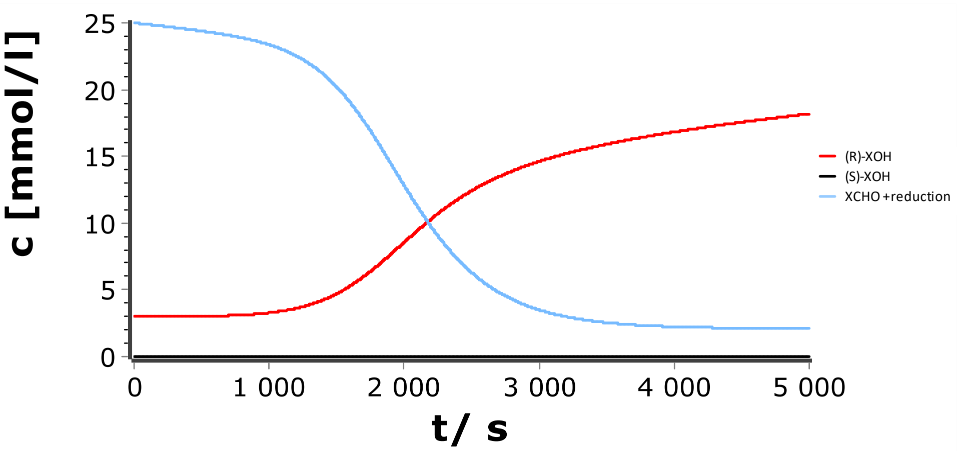

Supplementary Figure 112: Simulated concentration-time profile of the Soai reaction and the reduction side reaction (25 mM 6-((Trimethylsilyl)ethynyl)nicotinaldehyde **TMSPyr-CHO**, 3.0 mM 2-Methyl-(6-((trimethylsilyl)ethynyl)-pyridine-3-yl)propanol **TMSPyr-OH** (*ee* > 99.9%) and 40 mM *i*Pr_2Zn).

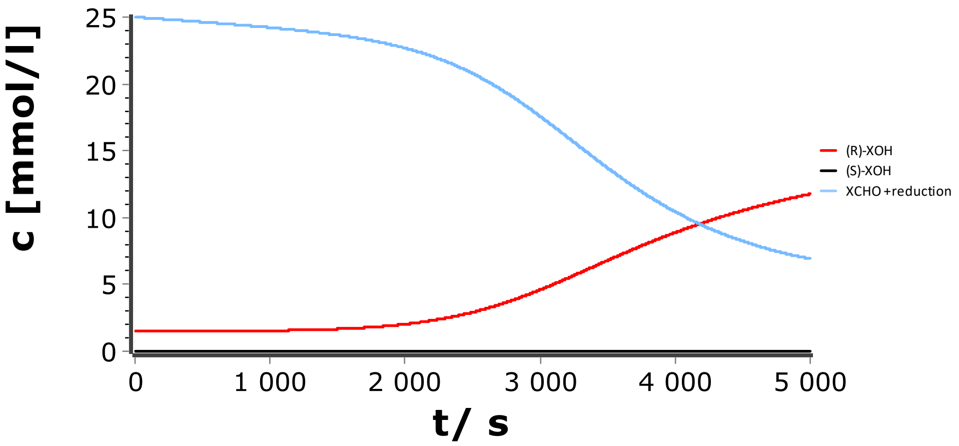

Supplementary Figure 113: Simulated concentration-time profile of the Soai reaction and the reduction side reaction (25 mM 6-((Trimethylsilyl)ethynyl)nicotinaldehyde **TMSPyr-CHO**, 1.5 mM 2-Methyl-(6-((trimethylsilyl)ethynyl)-pyridine-3-yl)propanol **TMSPyr-OH** (*ee* > 99.9%) and 25 mM *i*Pr_2Zn).

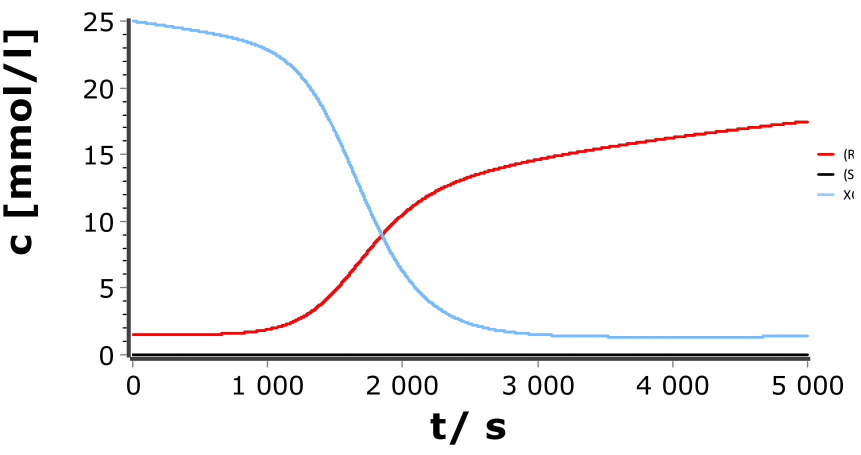

Supplementary Figure 114: Simulated concentration-time profile of the Soai reaction and the reduction side reaction (25 mM 6-((Trimethylsilyl)ethynyl)nicotinaldehyde **TMSPyr-CHO**, 1.5 mM 2-Methyl-(6-((trimethylsilyl)ethynyl)-pyridine-3-yl)propanol **TMSPyr-OH** (*ee* > 99.9%) and 50 mM *i*Pr_2Zn).

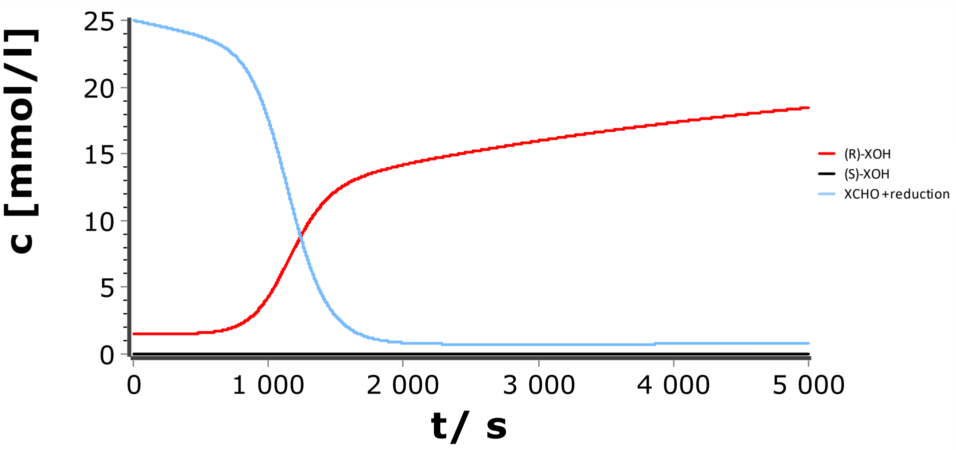

Supplementary Figure 115: Simulated concentration-time profile of the Soai reaction and the reduction side reaction (25 mM 6-((Trimethylsilyl)ethynyl)nicotinaldehyde **TMSPyr-CHO**, 1.5 mM 2-Methyl-(6-((trimethylsilyl)ethynyl)-pyridine-3-yl)propanol **TMSPyr-OH** (*ee* > 99.9%) and 75 mM *i*Pr_2Zn).

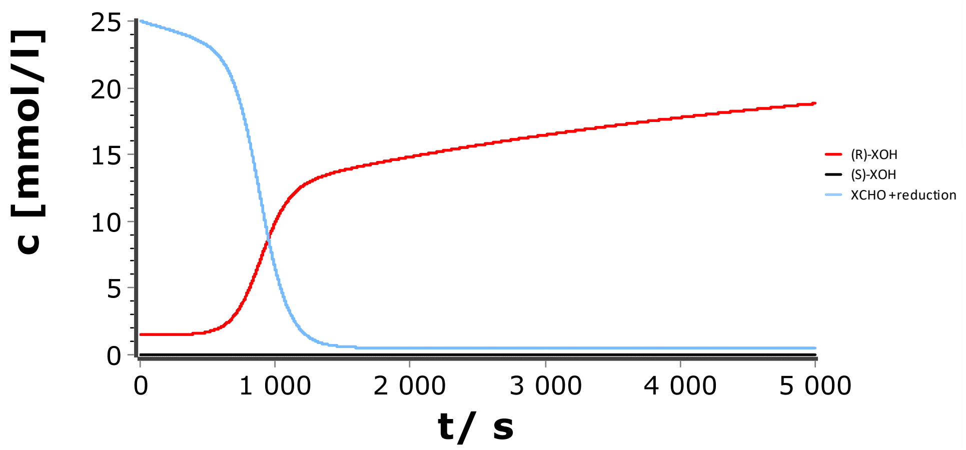

Supplementary Figure 116: Simulated concentration-time profile of the Soai reaction and the reduction side reaction (25 mM 6-((Trimethylsilyl)ethynyl)nicotinaldehyde **TMSPyr-CHO**, 1.5 mM 2-Methyl-(6-((trimethylsilyl)ethynyl)-pyridine-3-yl)propanol **TMSPyr-OH** (*ee* > 99.9%) and 100 mM $i\text{Pr}_2\text{Zn}$).

6.4.2 AdPyr-CHO/AdPyr-OH System

Supplementary Table 5: Simulation Parameters for the **AdPyr-CHO/AdPyr-OH** System**.**

	AdPyrCHO [mmol/L]	(*R*)-AdPyrOH [mmol/L]	(*S*)-AdPyrOH [mmol/L]	ee [%]	er	ZniPr$_2$ [mmol/L]	t [s]
**1**	36	2.9985	0.0015	99.9	1999	40	4800
**2**	40	2.9985	0.0015	99.9	1999	40	4800
**3**	45	2.9985	0.0015	99.9	1999	40	4800
**4**	56	2.9985	0.0015	99.9	1999	40	4800
**5**	65	2.9985	0.0015	99.9	1999	40	4800
**6**	40	1.5992	0.0008	99.9	1999	40	4800
**7**	40	2.49875	0.00125	99.9	1999	40	4800
**8**	40	2.9985	0.0015	99.9	1999	40	4800
**9**	40	3.49825	0.00175	99.9	1999	40	4800
**10**	40	2.9985	0.0015	99.9	1999	25	4800
**11**	40	2.9985	0.0015	99.9	1999	50	4800
**12**	40	2.9985	0.0015	99.9	1999	75	4800
**13**	40	2.9985	0.0015	99.9	1999	100	4800

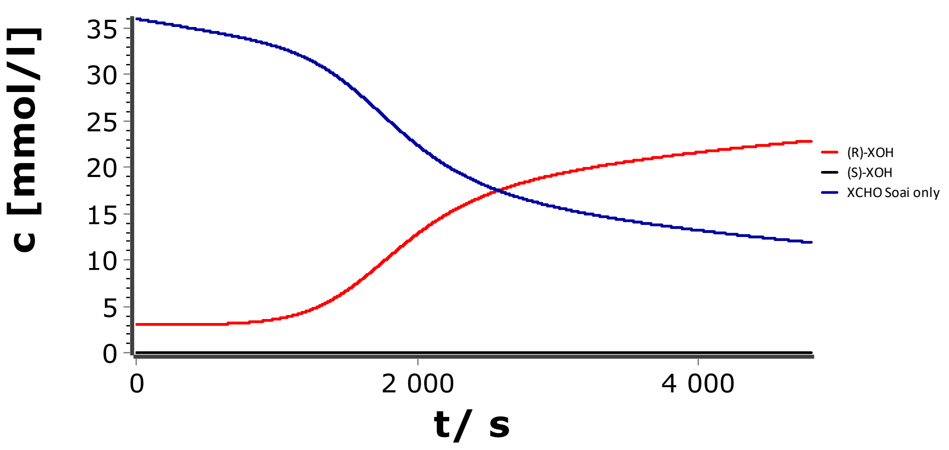

Supplementary Figure 117: Simulated concentration-time profile of the Soai reaction (36 mM 6-((adamantan-1-yl)ethynyl)nicotinaldehyde **AdPyr-CHO**, 3.0 mM (1R)-1-(6-((adamantan-1-yl)ethynyl)pyridin-3-yl)-2-methylpropan-1-ol **AdPyr-OH** (*ee* > 99.9%) and 40 mM *i*Pr$_2$Zn).

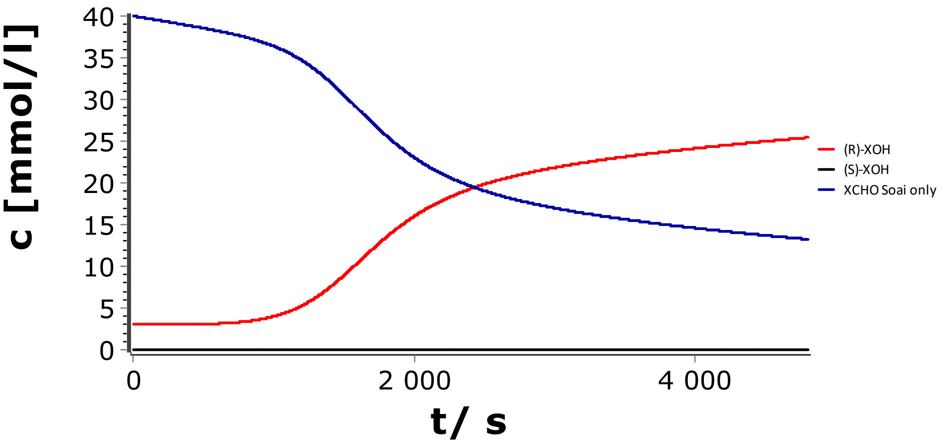

Supplementary Figure 118: Simulated concentration-time profile of the Soai reaction (40 mM 6-((adamantan-1-yl)ethynyl)nicotinaldehyde **AdPyr-CHO**, 3.0 mM (1R)-1-(6-((adamantan-1-yl)ethynyl)pyridin-3-yl)-2-methylpropan-1-ol **AdPyr-OH** (*ee* > 99.9%) and 40 mM *i*Pr_2Zn).

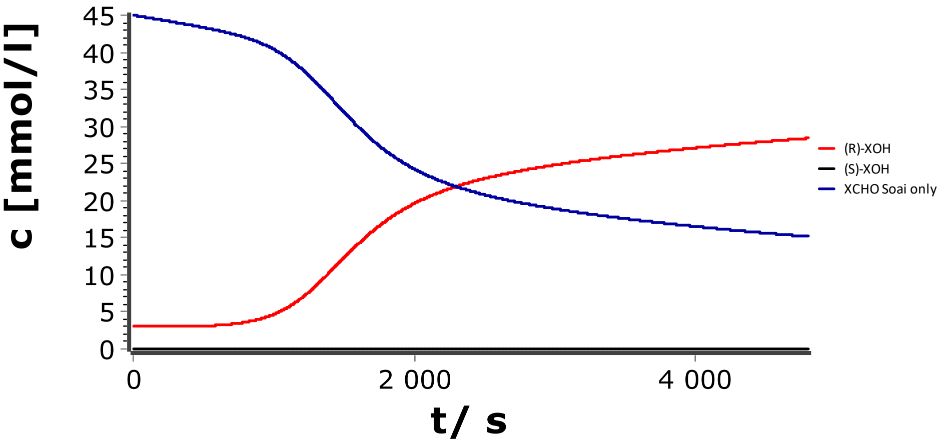

Supplementary Figure 119: Simulated concentration-time profile of the Soai reaction (45 mM 6-((adamantan-1-yl)ethynyl)nicotinaldehyde **AdPyr-CHO**, 3.0 mM (1R)-1-(6-((adamantan-1-yl)ethynyl)pyridin-3-yl)-2-methylpropan-1-ol **AdPyr-OH** (*ee* > 99.9%) and 40 mM *i*Pr_2Zn).

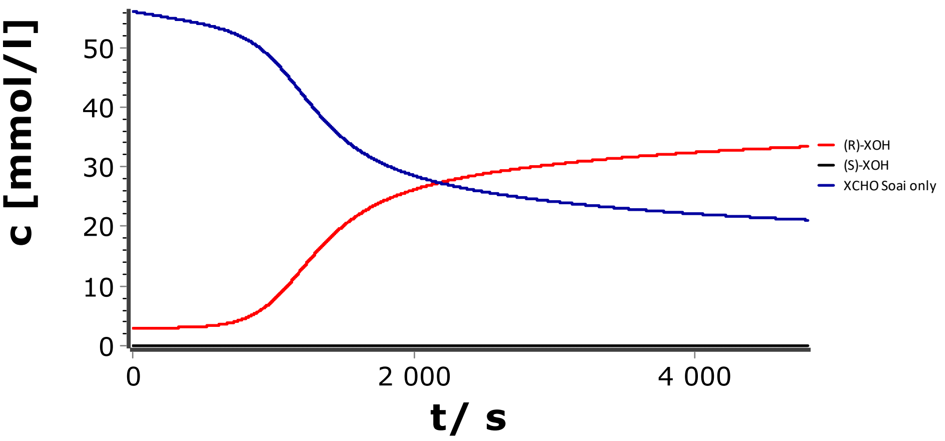

Supplementary Figure 120: Simulated concentration-time profile of the Soai reaction (56 mM 6-((adamantan-1-yl)ethynyl)nicotinaldehyde **AdPyr-CHO**, 3.0 mM (1R)-1-(6-((adamantan-1-yl)ethynyl)pyridin-3-yl)-2-methylpropan-1-ol **AdPyr-OH** (*ee* > 99.9%) and 40 mM *i*Pr_2Zn).

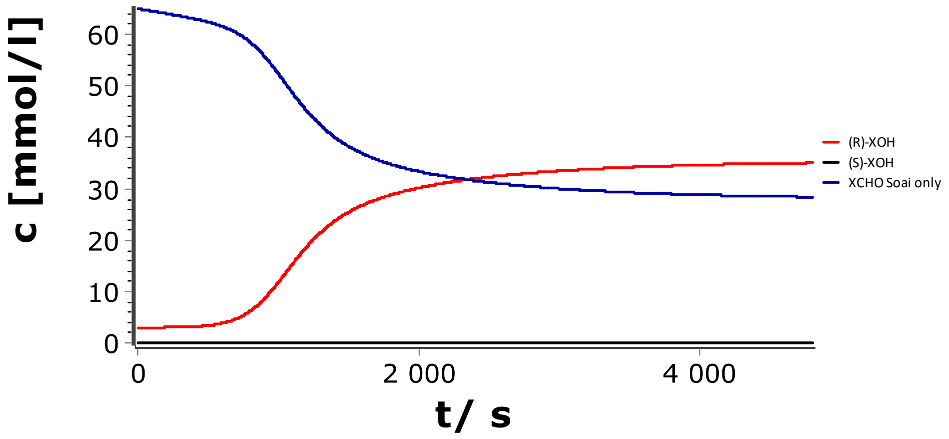

Supplementary Figure 121: Simulated concentration-time profile of the Soai reaction (65 mM 6-((adamantan-1-yl)ethynyl)nicotinaldehyde **AdPyr-CHO**, 3.0 mM (1R)-1-(6-((adamantan-1-yl)ethynyl)pyridin-3-yl)-2-methylpropan-1-ol **AdPyr-OH** (*ee* > 99.9%) and 40 mM *i*Pr_2Zn).

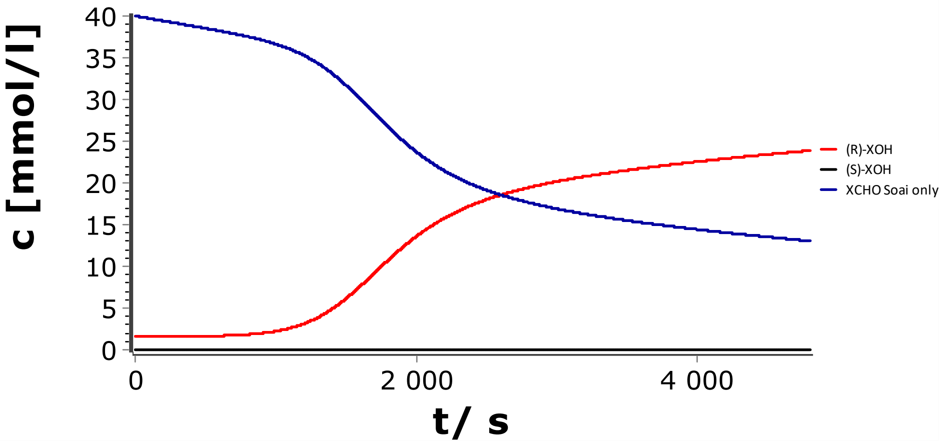

Supplementary Figure 122: Simulated concentration-time profile of the Soai reaction (40 mM 6-((adamantan-1-yl)ethynyl)nicotinaldehyde **AdPyr-CHO**, 1.6 mM (1R)-1-(6-((adamantan-1-yl)ethynyl)pyridin-3-yl)-2-methylpropan-1-ol **AdPyr-OH** (*ee* > 99.9%) and 40 mM *i*Pr_2Zn).

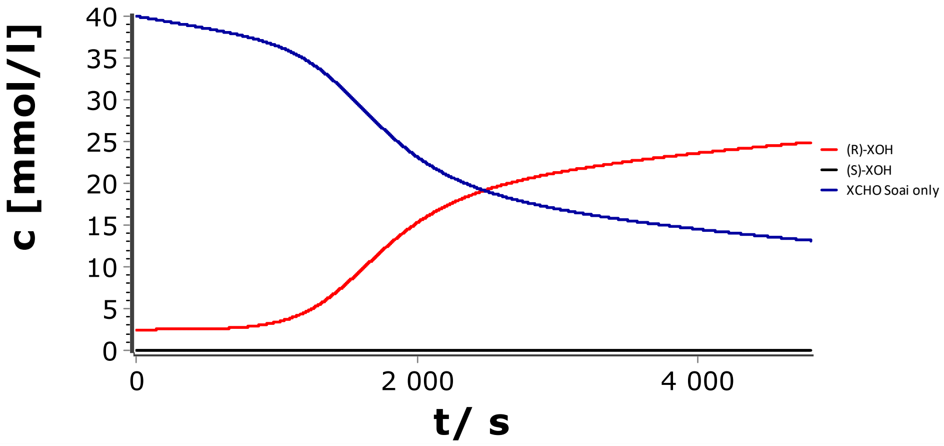

Supplementary Figure 123: Simulated concentration-time profile of the Soai reaction (40 mM 6-((adamantan-1-yl)ethynyl)nicotinaldehyde **AdPyr-CHO**, 2.5 mM (1R)-1-(6-((adamantan-1-yl)ethynyl)pyridin-3-yl)-2-methylpropan-1-ol **AdPyr-OH** **(***ee* > 99.9%) and 40 mM *i*Pr_2Zn).

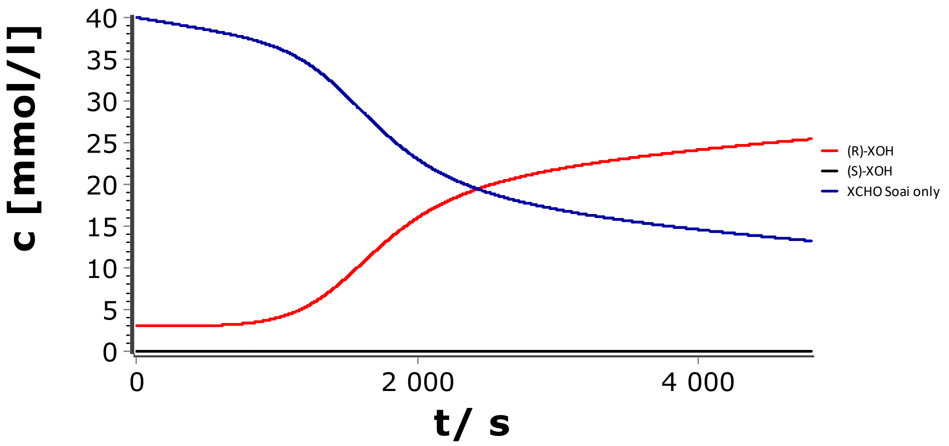

Supplementary Figure 124: Simulated concentration-time profile of the Soai reaction (40 mM 6-((adamantan-1-yl)ethynyl)nicotinaldehyde **AdPyr-CHO**, 3.0 mM (1R)-1-(6-((adamantan-1-yl)ethynyl)pyridin-3-yl)-2-methylpropan-1-ol **AdPyr-OH** (*ee* > 99.9%) and 40 mM *i*Pr_2Zn).

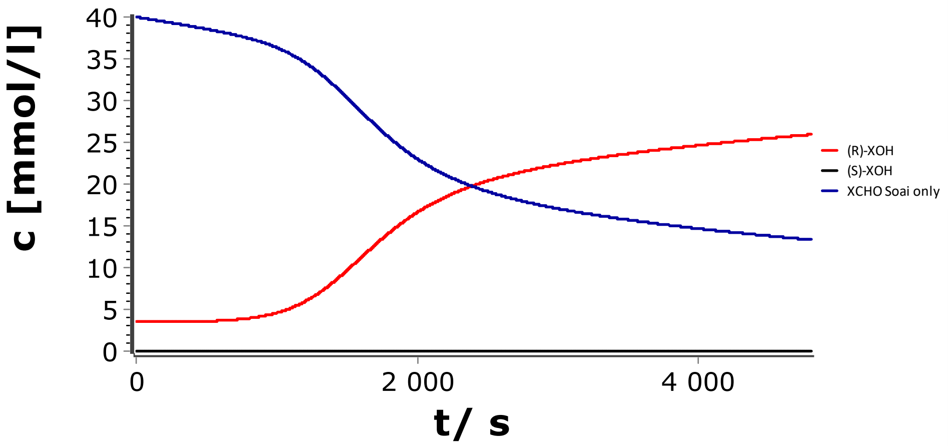

Supplementary Figure 125: Simulated concentration-time profile of the Soai reaction (40 mM 6-((adamantan-1-yl)ethynyl)nicotinaldehyde **AdPyr-CHO**, 3.5 mM (1R)-1-(6-((adamantan-1-yl)ethynyl)pyridin-3-yl)-2-methylpropan-1-ol **AdPyr-OH** (*ee* > 99.9%) and 40 mM *i*Pr_2Zn).

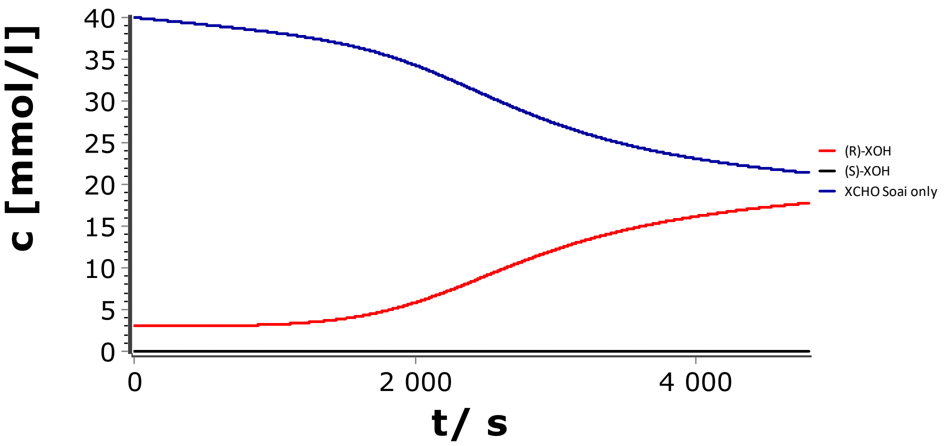

Supplementary Figure 126: Simulated concentration-time profile of the Soai reaction (40 mM 6-((adamantan-1-yl)ethynyl)nicotinaldehyde **AdPyr-CHO**, 3.0 mM (1R)-1-(6-((adamantan-1-yl)ethynyl)pyridin-3-yl)-2-methylpropan-1-ol **AdPyr-OH** (*ee* > 99.9%) and 25 mM *i*Pr_2Zn).

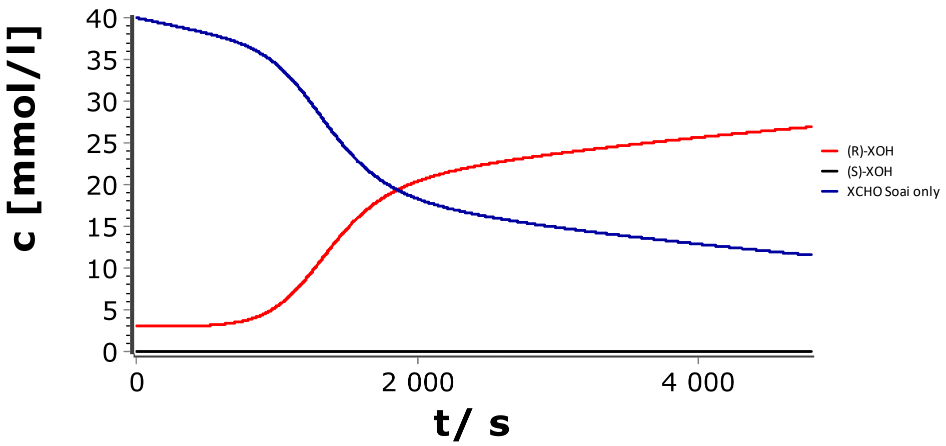

Supplementary Figure 127: Simulated concentration-time profile of the Soai reaction (40 mM 6-((adamantan-1-yl)ethynyl)nicotinaldehyde **AdPyr-CHO**, 3.0 mM (1R)-1-(6-((adamantan-1-yl)ethynyl)pyridin-3-yl)-2-methylpropan-1-ol **AdPyr-OH** (*ee* > 99.9%) and 50 mM *i*Pr_2Zn).

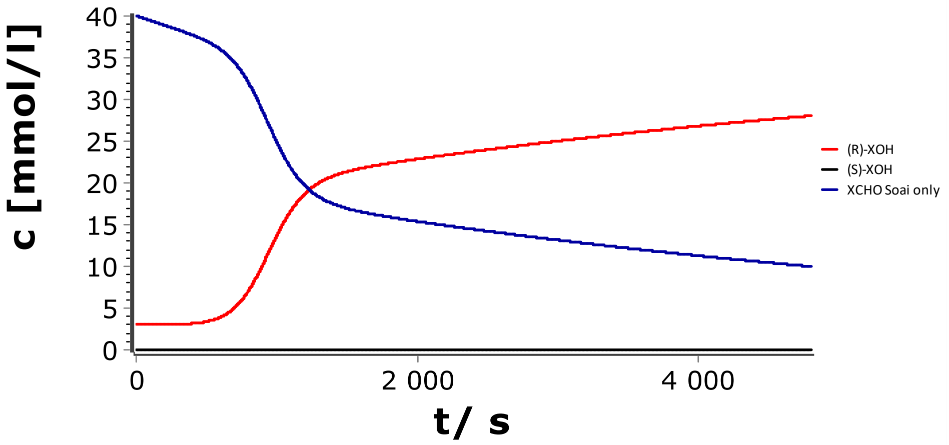

Supplementary Figure 128: Simulated concentration-time profile of the Soai reaction (40 mM 6-((adamantan-1-yl)ethynyl)nicotinaldehyde **AdPyr-CHO**, 3.0 mM (1R)-1-(6-((adamantan-1-yl)ethynyl)pyridin-3-yl)-2-methylpropan-1-ol **AdPyr-OH** (*ee* > 99.9%) and 75 mM *i*Pr_2Zn).

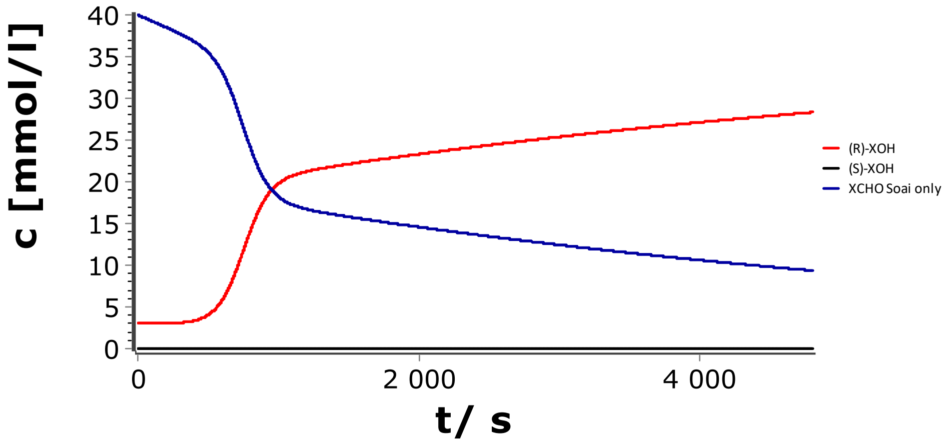

Supplementary Figure 129: Simulated concentration-time profile of the Soai reaction (40 mM 6-((adamantan-1-yl)ethynyl)nicotinaldehyde **AdPyr-CHO**, 3.0 mM (1R)-1-(6-((adamantan-1-yl)ethynyl)pyridin-3-yl)-2-methylpropan-1-ol **AdPyr-OH** (*ee* > 99.9%) and 100 mM *i*Pr_2Zn).

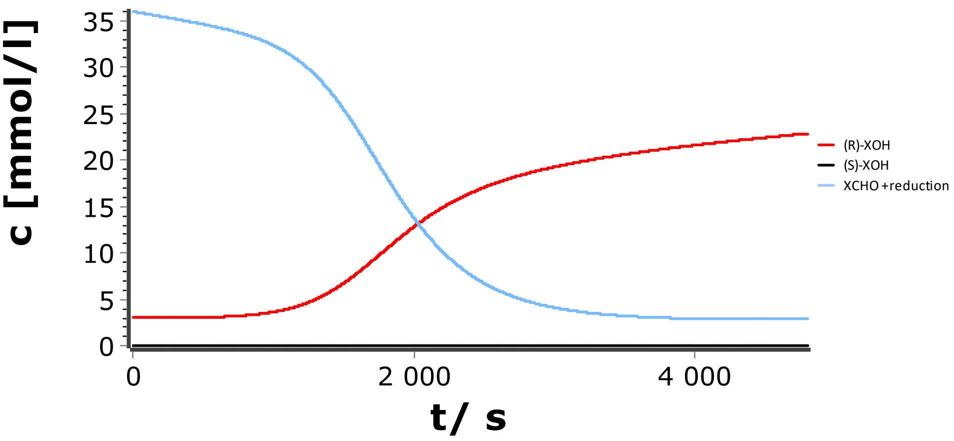

Supplementary Figure 130: Simulated concentration-time profile of the Soai reaction and the reduction side reaction (36 mM 6-((adamantan-1-yl)ethynyl)nicotinaldehyde **AdPyr-CHO**, 3.0 mM (1R)-1-(6-((adamantan-1-yl)ethynyl)pyridin-3-yl)-2-methylpropan-1-ol **AdPyr-OH** (*ee* > 99.9%) and 40 mM *i*Pr_2Zn).

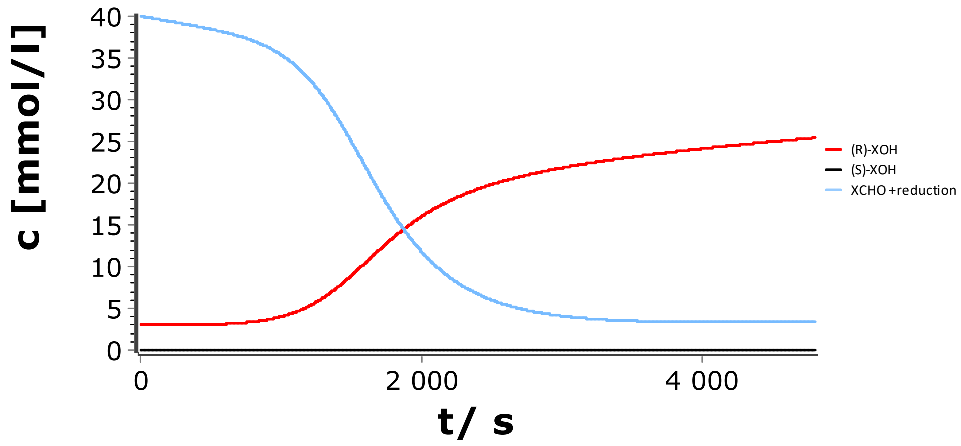

Supplementary Figure 131: Simulated concentration-time profile of the Soai reaction and the reduction side reaction (40 mM 6-((adamantan-1-yl)ethynyl)nicotinaldehyde **AdPyr-CHO,** 3.0 mM (1R)-1-(6-((adamantan-1-yl)ethynyl)pyridin-3-yl)-2-methylpropan-1-ol **AdPyr-OH** (*ee* > 99.9%) and 40 mM *i*Pr_2Zn).

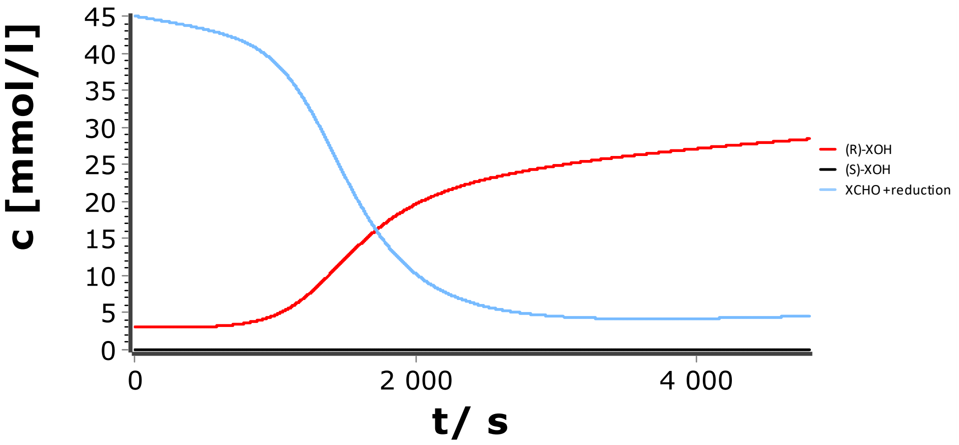

Supplementary Figure 132: Simulated concentration-time profile of the Soai reaction and the reduction side reaction (45 mM 6-((adamantan-1-yl)ethynyl)nicotinaldehyde **AdPyr-CHO**, 3.0 mM (1R)-1-(6-((adamantan-1-yl)ethynyl)pyridin-3-yl)-2-methylpropan-1-ol **AdPyr-OH** (*ee* > 99.9%) and 40 mM iPr_2Zn).

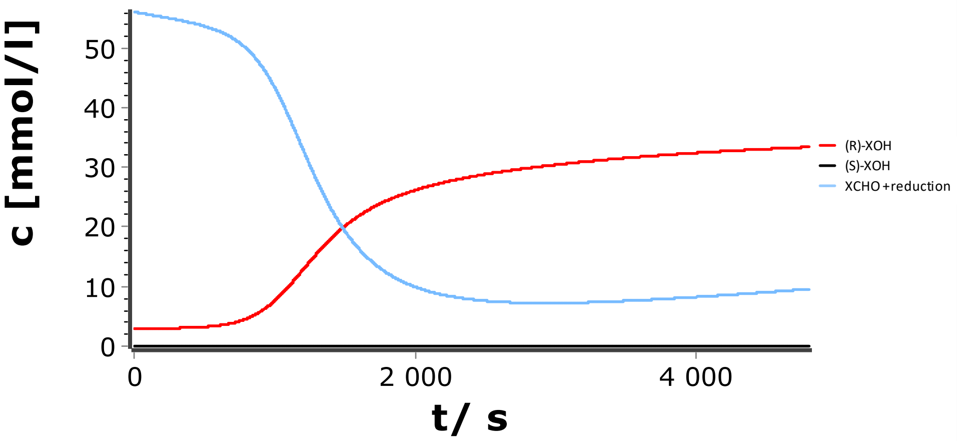

Supplementary Figure 133: Simulated concentration-time profile of the Soai reaction and the reduction side reaction (56 mM 6-((adamantan-1-yl)ethynyl)nicotinaldehyde **AdPyr-CHO**, 3.0 mM (1R)-1-(6-((adamantan-1-yl)ethynyl)pyridin-3-yl)-2-methylpropan-1-ol **AdPyr-OH** (*ee* > 99.9%) and 40 mM iPr_2Zn).

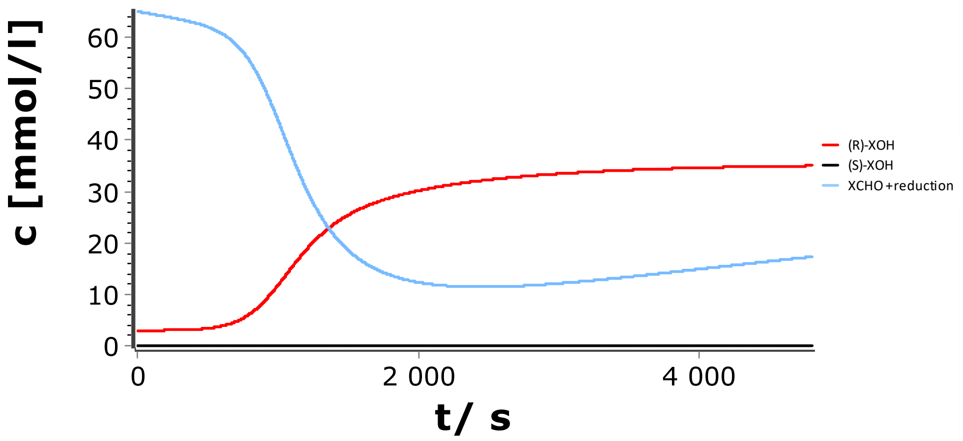

Supplementary Figure 134: Simulated concentration-time profile of the Soai reaction and the reduction side reaction (65 mM 6-((adamantan-1-yl)ethynyl)nicotinaldehyde **AdPyr-CHO**, 3.0 mM (1R)-1-(6-((adamantan-1-yl)ethynyl)pyridin-3-yl)-2-methylpropan-1-ol **AdPyr-OH** (*ee* > 99.9%) and 40 mM *i*Pr_2Zn).

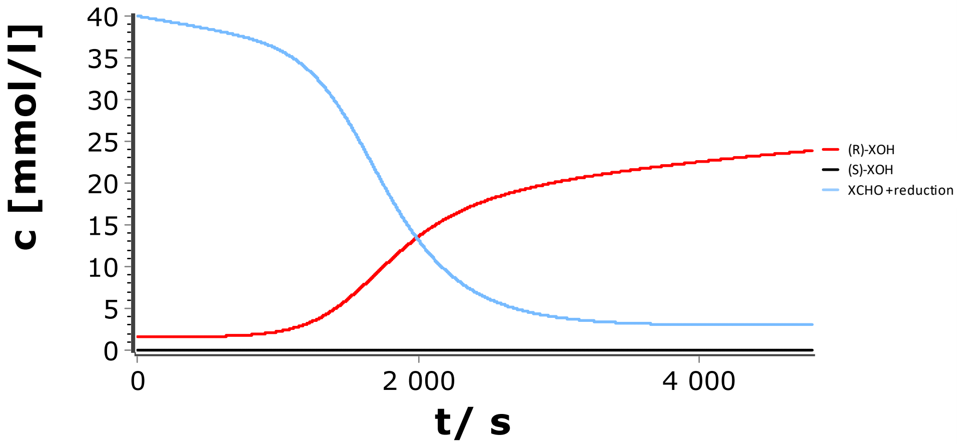

Supplementary Figure 135: Simulated concentration-time profile of the Soai reaction and the reduction side reaction (40 mM 6-((adamantan-1-yl)ethynyl)nicotinaldehyde **AdPyr-CHO**, 1.6 mM (1R)-1-(6-((adamantan-1-yl)ethynyl)pyridin-3-yl)-2-methylpropan-1-ol **AdPyr-OH** (*ee* > 99.9%) and 40 mM *i*Pr_2Zn).

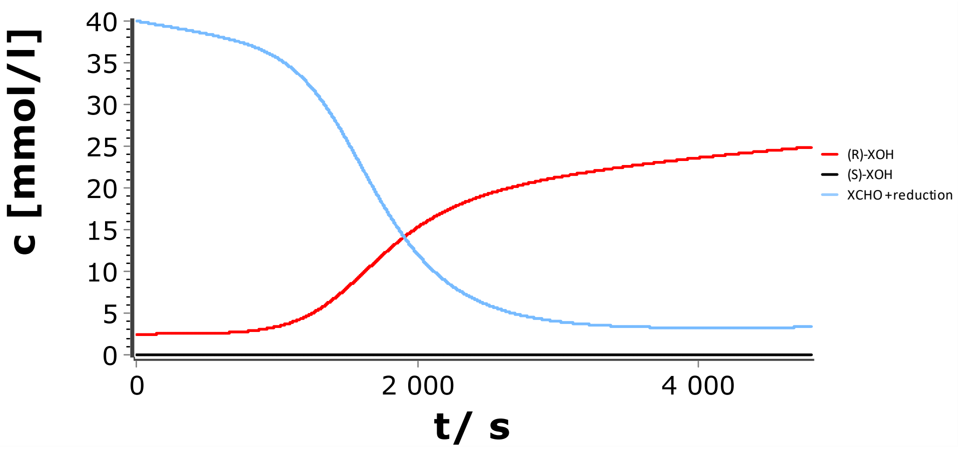

Supplementary Figure 136: Simulated concentration-time profile of the Soai reaction and the reduction side reaction (40 mM 6-((adamantan-1-yl)ethynyl)nicotinaldehyde **AdPyr-CHO**, 2.5 mM (1R)-1-(6-((adamantan-1-yl)ethynyl)pyridin-3-yl)-2-methylpropan-1-ol **AdPyr-OH** (*ee* > 99.9%) and 40 mM *i*Pr_2Zn).

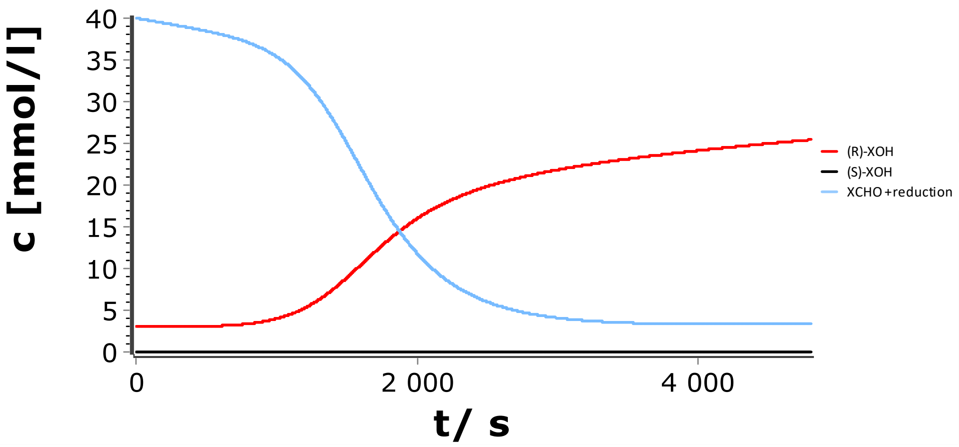

Supplementary Figure 137: Simulated concentration-time profile of the Soai reaction and the reduction side reaction (40 mM 6-((adamantan-1-yl)ethynyl)nicotinaldehyde **AdPyr-CHO**, 3.0 mM (1R)-1-(6-((adamantan-1-yl)ethynyl)pyridin-3-yl)-2-methylpropan-1-ol **AdPyr-OH** (*ee* > 99.9%) and 40 mM *i*Pr_2Zn).

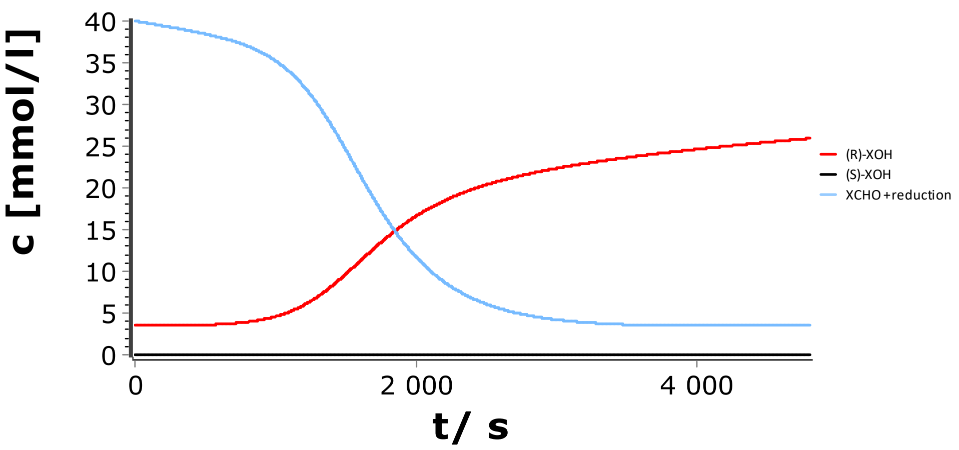

Supplementary Figure 138: Simulated concentration-time profile of the Soai reaction and the reduction side reaction (40 mM 6-((adamantan-1-yl)ethynyl)nicotinaldehyde **AdPyr-CHO**, 3.5 mM (1R)-1-(6-((adamantan-1-yl)ethynyl)pyridin-3-yl)-2-methylpropan-1-ol **AdPyr-OH** (*ee* > 99.9%) and 40 mM *i*Pr_2Zn).

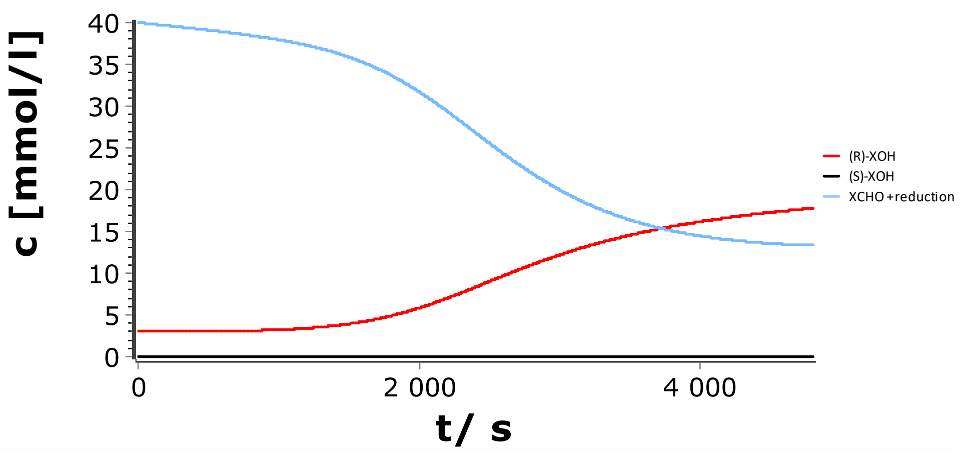

Supplementary Figure 139: Simulated concentration-time profile of the Soai reaction and the reduction side reaction (40 mM 6-((adamantan-1-yl)ethynyl)nicotinaldehyde **AdPyr-CHO**, 3.0 mM (1R)-1-(6-((adamantan-1-yl)ethynyl)pyridin-3-yl)-2-methylpropan-1-ol **AdPyr-OH** (*ee* > 99.9%) and 25 mM *i*Pr_2Zn).

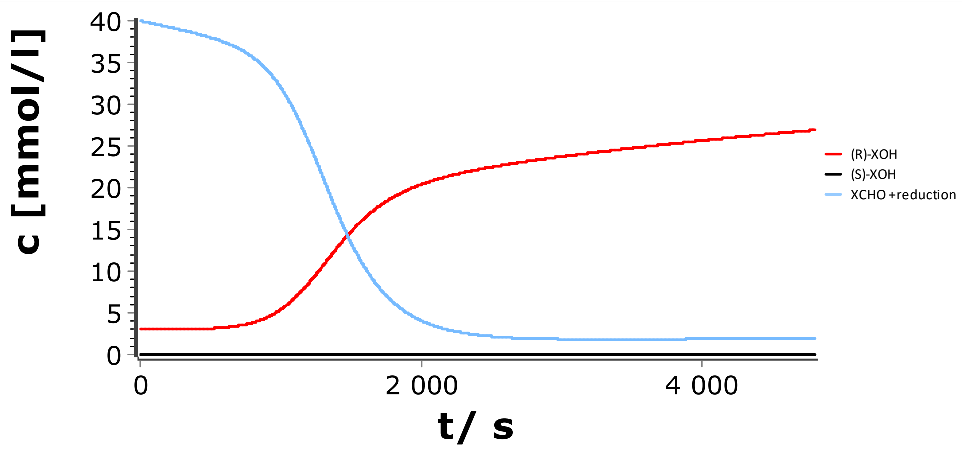

Supplementary Figure 140: Simulated concentration-time profile of the Soai reaction and the reduction side reaction (40 mM 6-((adamantan-1-yl)ethynyl)nicotinaldehyde **AdPyr-CHO**, 3.0 mM (1R)-1-(6-((adamantan-1-yl)ethynyl)pyridin-3-yl)-2-methylpropan-1-ol **AdPyr-OH** (*ee* > 99.9%) and 50 mM *i*Pr_2Zn).

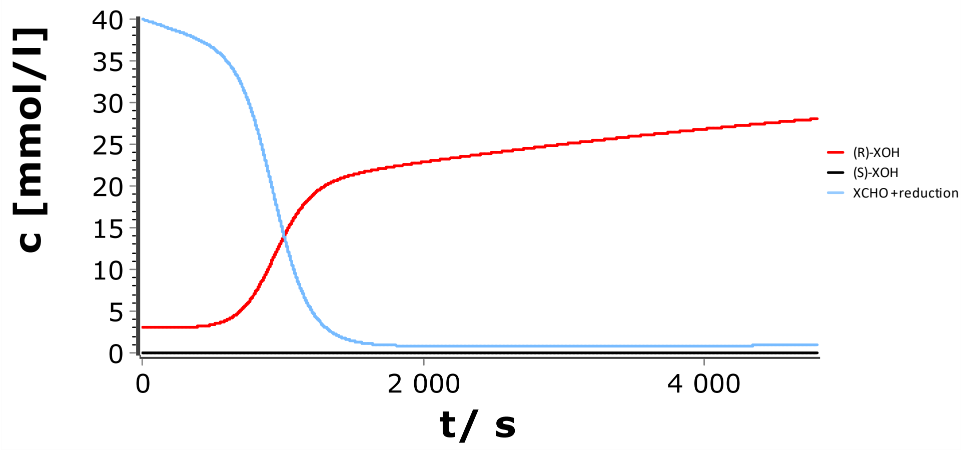

Supplementary Figure 141: Simulated concentration-time profile of the Soai reaction and the reduction side reaction (40 mM 6-((adamantan-1-yl)ethynyl)nicotinaldehyde **AdPyr-CHO**, 3.0 mM (1R)-1-(6-((adamantan-1-yl)ethynyl)pyridin-3-yl)-2-methylpropan-1-ol **AdPyr-OH** (*ee* > 99.9%) and 75 mM *i*Pr_2Zn).

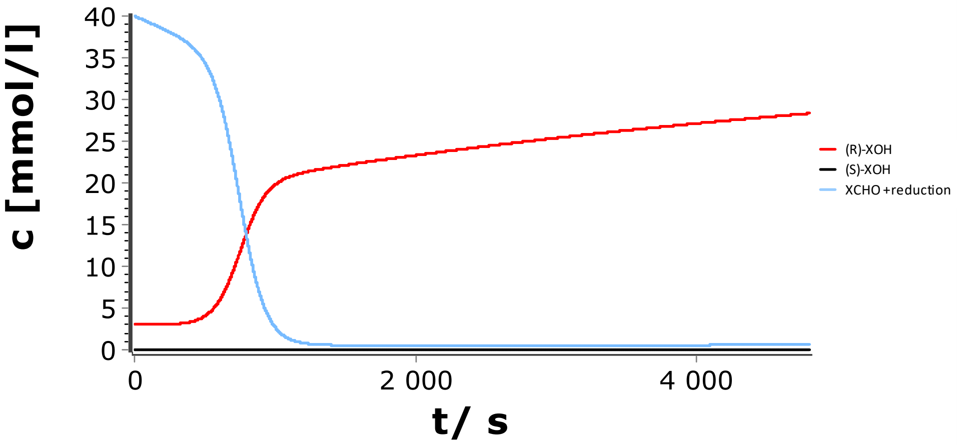

Supplementary Figure 142: Simulated concentration-time profile of the Soai reaction and the reduction side reaction (40 mM 6-((adamantan-1-yl)ethynyl)nicotinaldehyde **AdPyr-CHO**, 3.0 mM (1R)-1-(6-((adamantan-1-yl)ethynyl)pyridin-3-yl)-2-methylpropan-1-ol **AdPyr-OH** (*ee* > 99.9%) and 100 mM $i\text{Pr}_2\text{Zn}$).

6.4.3 TMSPym-CHO/TMSPym-OH System

Supplementary Table 6: Simulation Parameters for the **TMSPym-CHO/TMSPym-OH** System.

	TMSPymCHO [mmol/L]	(*R*)-TMSPymOH [mmol/L]	(*S*)-TMSPymOH [mmol/L]	ee [%]	er	$ZniPr_2$ [mmol/L]	t [s]
**1**	10	1.49925	0.00075	99.9	1999	40	2500
**2**	20	1.49925	0.00075	99.9	1999	40	2500
**3**	27	1.49925	0.00075	99.9	1999	40	2500
**4**	35	1.49925	0.00075	99.9	1999	40	2500
**5**	25	0.749625	0.000375	99.9	1999	40	2500
**6**	25	1.49925	0.00075	99.9	1999	40	2500
**7**	25	5.1974	0.0026	99.9	1999	40	2500
**8**	25	1.49925	0.00075	99.9	1999	25	2500
**9**	25	1.49925	0.00075	99.9	1999	50	2500
**10**	25	1.49925	0.00075	99.9	1999	75	2500
**11**	25	1.49925	0.00075	99.9	1999	100	2500

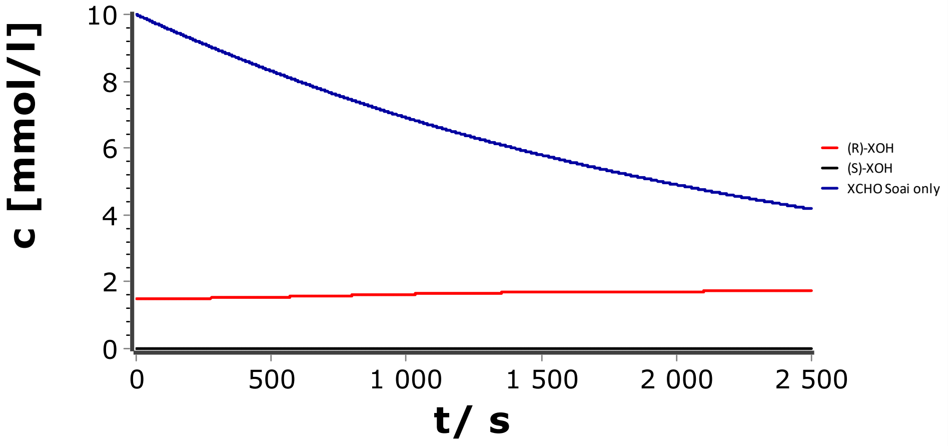

Supplementary Figure 143: Simulated concentration-time profile of the Soai reaction (10 mM 2-((trimethylsilyl)ethynyl)pyrimidine-5-carbaldehyde **TMSPym-CHO**, 1.5 mM (1*R*)-2-methyl-1-(2-((trimethylsilyl)ethynyl)pyrimidin-5-yl)propan-1-ol **TMSPym-OH** (*ee* > 99.9%) and 40 mM iPr_2Zn).

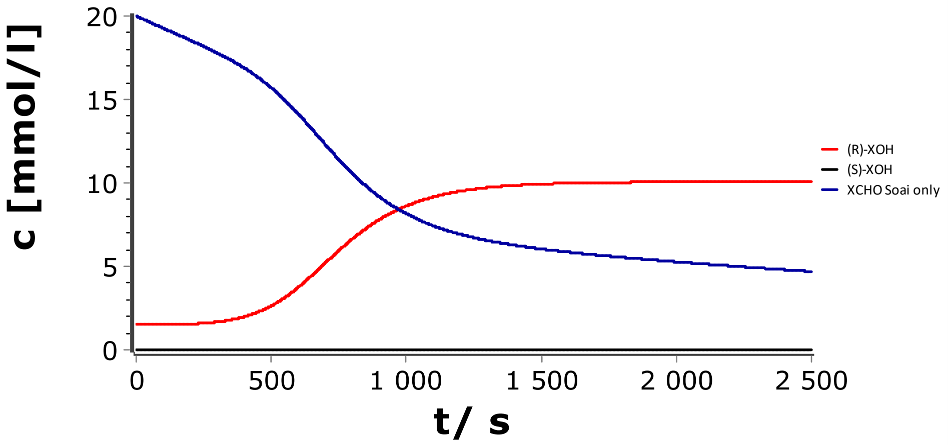

Supplementary Figure 144: Simulated concentration-time profile of the Soai reaction (20 mM 2-((trimethylsilyl)ethynyl)pyrimidine-5-carbaldehyde **TMSPym-CHO**, 1.5 mM (1*R*)-2-methyl-1-(2-((trimethylsilyl)ethynyl)pyrimidin-5-yl)propan-1-ol **TMSPym-OH** (*ee* > 99.9%) and 40 mM *i*Pr_2Zn).

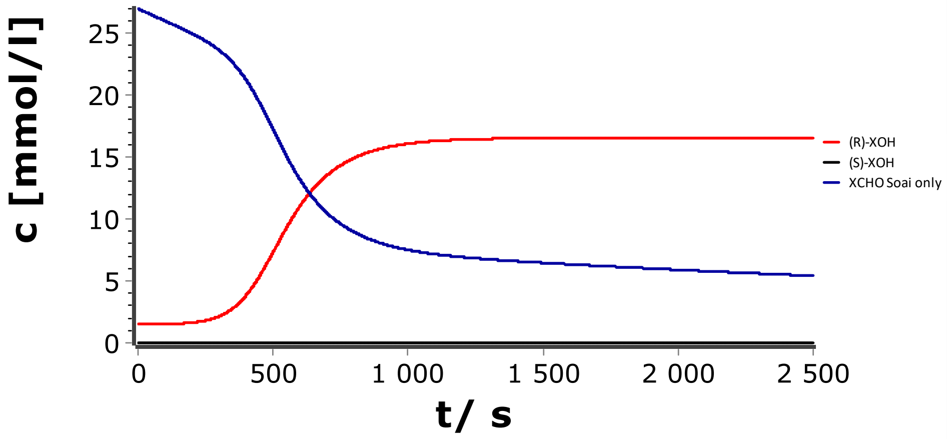

Supplementary Figure 145: Simulated concentration-time profile of the Soai reaction (27 mM 2-((trimethylsilyl)ethynyl)pyrimidine-5-carbaldehyde **TMSPym-CHO**, 1.5 mM (1*R*)-2-methyl-1-(2-((trimethylsilyl)ethynyl)pyrimidin-5-yl)propan-1-ol **TMSPym-OH** (*ee* > 99.9%) and 40 mM *i*Pr_2Zn).

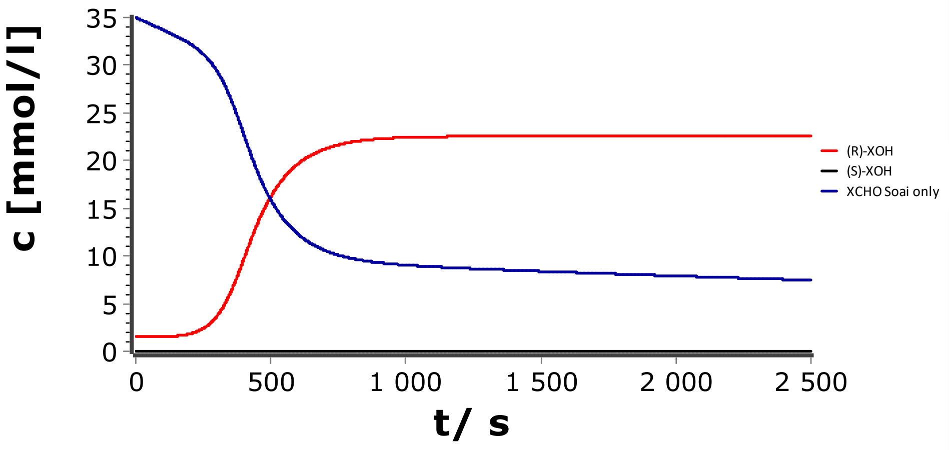

Supplementary Figure 146: Simulated concentration-time profile of the Soai reaction (35 mM 2-((trimethylsilyl)ethynyl)pyrimidine-5-carbaldehyde **TMSPym-CHO**, 1.5 mM (1*R*)-2-methyl-1-(2-((trimethylsilyl)ethynyl)pyrimidin-5-yl)propan-1-ol **TMSPym-OH** (*ee* > 99.9%) and 40 mM *i*Pr_2Zn**).**

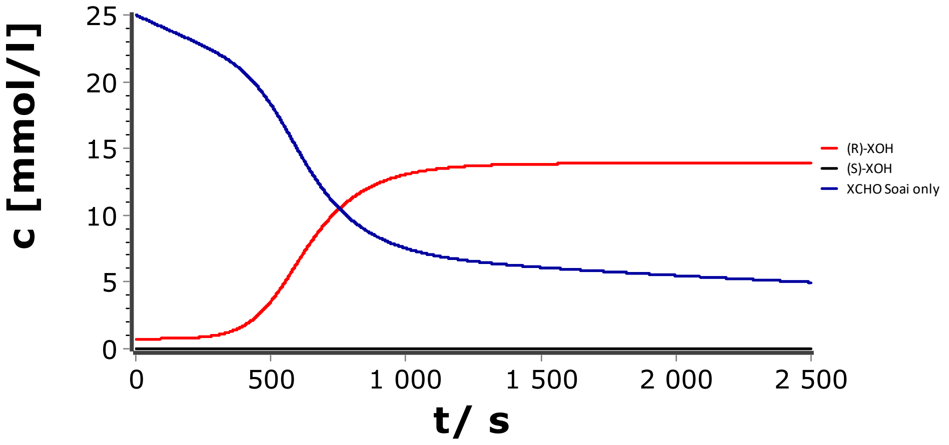

Supplementary Figure 147: Simulated concentration-time profile of the Soai reaction (25 mM 2-((trimethylsilyl)ethynyl)pyrimidine-5-carbaldehyde **TMSPym-CHO**, 0.75 mM (1R)-2-methyl-1-(2-((trimethylsilyl)ethynyl)pyrimidin-5-yl)propan-1-ol **TMSPym-OH** (ee > 99.9%) and 40 mM iPr_2Zn).

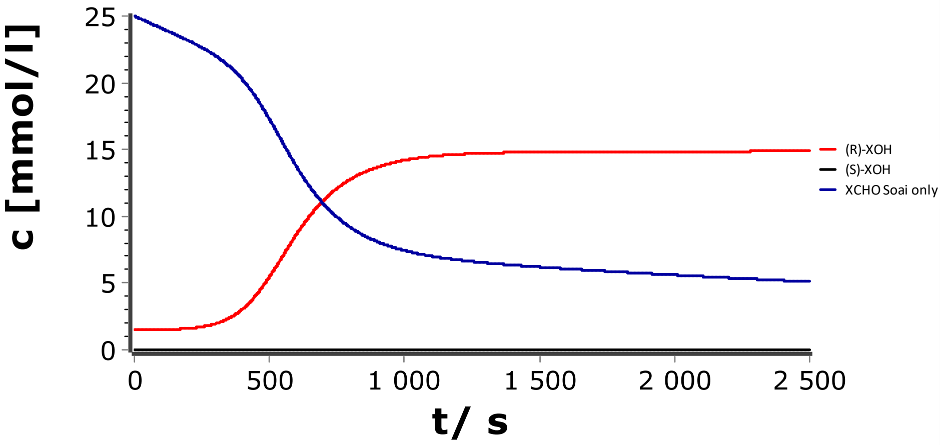

Supplementary Figure 148: Simulated concentration-time profile of the Soai reaction (25 mM 2-((trimethylsilyl)ethynyl)pyrimidine-5-carbaldehyde **TMSPym-CHO**, 1.5 mM (1R)-2-methyl-1-(2-((trimethylsilyl)ethynyl)pyrimidin-5-yl)propan-1-ol **TMSPym-OH** (ee > 99.9%) and 40 mM iPr_2Zn).

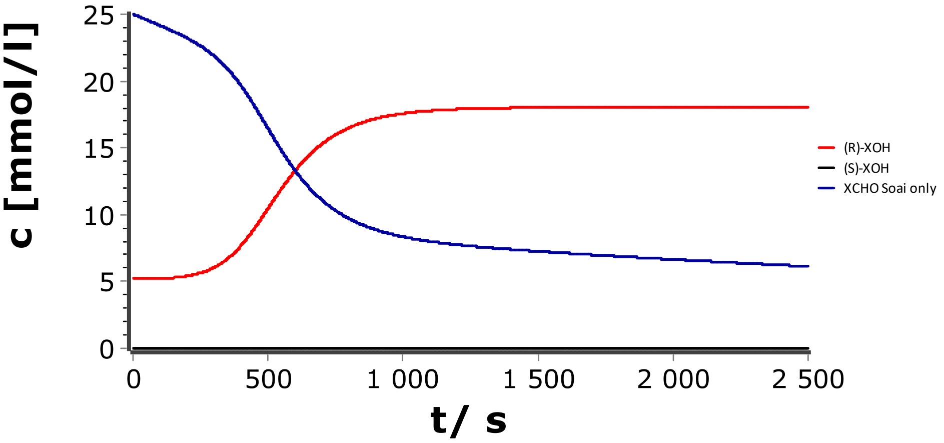

Supplementary Figure 149: Simulated concentration-time profile of the Soai reaction (25 mM 2-((trimethylsilyl)ethynyl)pyrimidine-5-carbaldehyde **TMSPym-CHO**, 5.2 mM (1R)-2-methyl-1-(2-((trimethylsilyl)ethynyl)pyrimidin-5-yl)propan-1-ol **TMSPym-OH** (ee > 99.9%) and 40 mM iPr_2Zn).

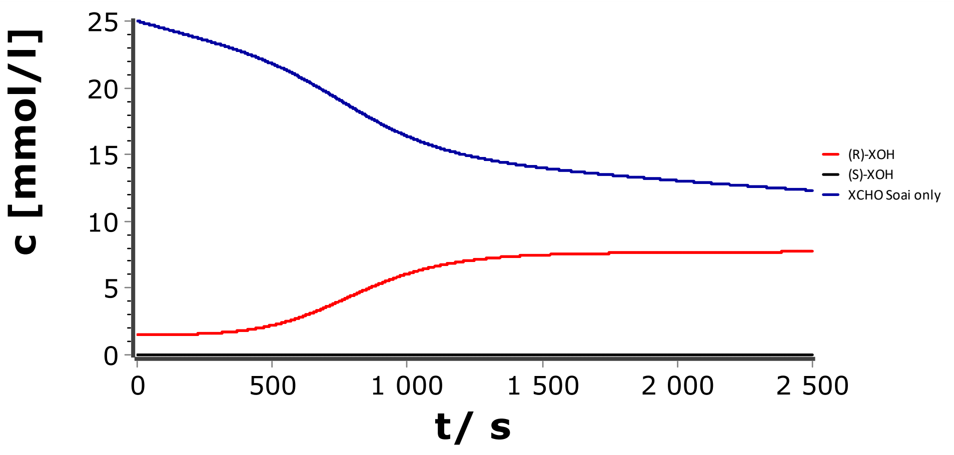

Supplementary Figure 150: Simulated concentration-time profile of the Soai reaction (25 mM 2-((trimethylsilyl)ethynyl)pyrimidine-5-carbaldehyde **TMSPym-CHO**, 1.5 mM (1R)-2-methyl-1-(2-((trimethylsilyl)ethynyl)pyrimidin-5-yl)propan-1-ol **TMSPym-OH** (ee > 99.9%) and 25 mM iPr_2Zn).

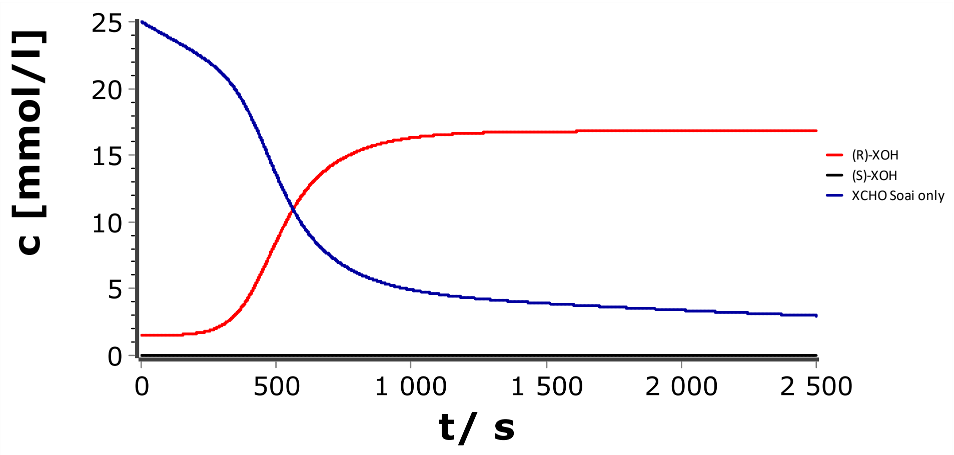

Supplementary Figure 151: Simulated concentration-time profile of the Soai reaction (25 mM 2-((trimethylsilyl)ethynyl)pyrimidine-5-carbaldehyde **TMSPym-CHO**, 1.5 mM (1R)-2-methyl-1-(2-((trimethylsilyl)ethynyl)pyrimidin-5-yl)propan-1-ol **TMSPym-OH** (ee > 99.9%) and 50 mM iPr_2Zn).

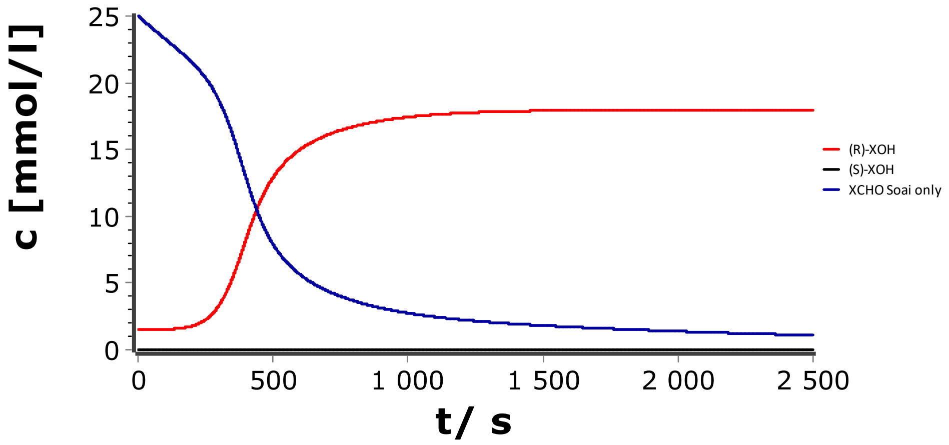

Supplementary Figure 152: Simulated concentration-time profile of the Soai reaction (25 mM 2-((trimethylsilyl)ethynyl)pyrimidine-5-carbaldehyde **TMSPym-CHO**, 1.5 mM (1R)-2-methyl-1-(2-((trimethylsilyl)ethynyl)pyrimidin-5-yl)propan-1-ol **TMSPym-OH** (ee > 99.9%) and 75 mM iPr_2Zn).

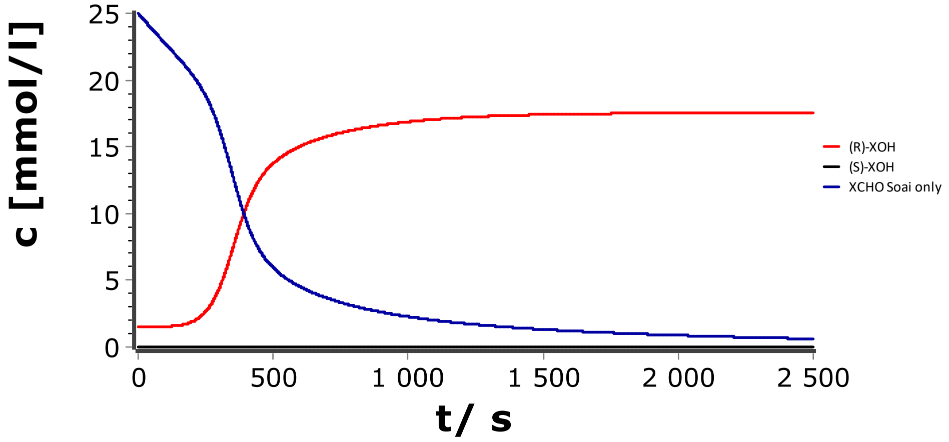

Supplementary Figure 153: Simulated concentration-time profile of the Soai reaction (25 mM 2-((trimethylsilyl)ethynyl)pyrimidine-5-carbaldehyde **TMSPym-CHO**, 1.5 mM (1R)-2-methyl-1-(2-((trimethylsilyl)ethynyl)pyrimidin-5-yl)propan-1-ol **TMSPym-OH** (ee > 99.9%) and 100 mM iPr_2Zn).

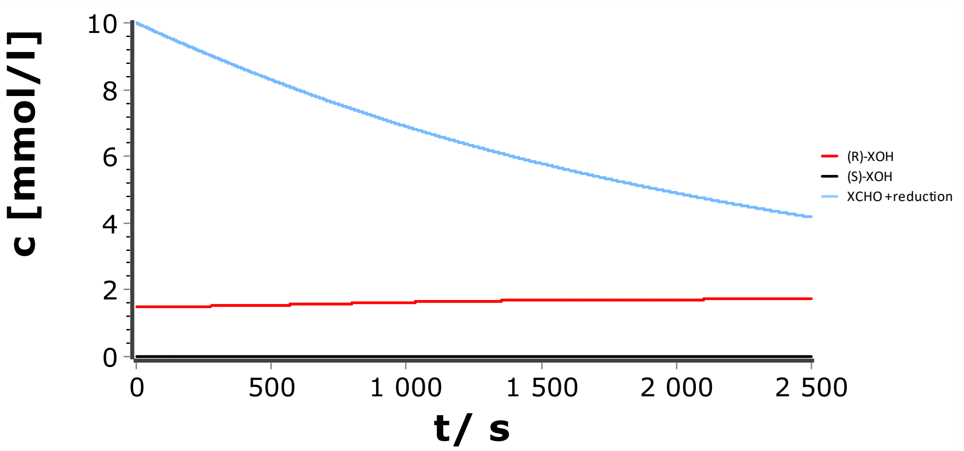

Supplementary Figure 154: Simulated concentration-time profile of the Soai reaction and the reduction side reaction (10 mM 2-((trimethylsilyl)ethynyl)pyrimidine-5-carbaldehyde **TMSPym-CHO**, 1.5 mM (1R)-2-methyl-1-(2-((trimethylsilyl)ethynyl)pyrimidin-5-yl)propan-1-ol **TMSPym-OH** (ee > 99.9%) and 40 mM iPr_2Zn).

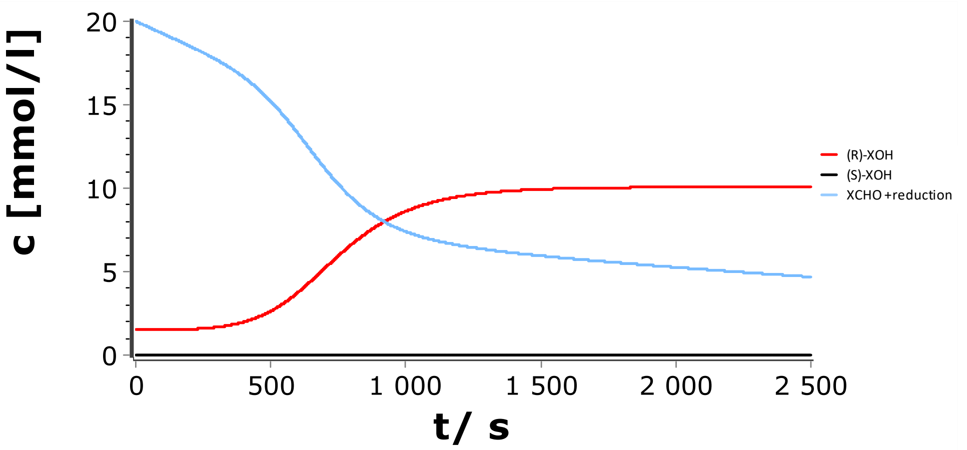

Supplementary Figure 155: Simulated concentration-time profile of the Soai reaction and the reduction side reaction (20 mM 2-((trimethylsilyl)ethynyl)pyrimidine-5-carbaldehyde **TMSPym-CHO**, 1.5 mM (1R)-2-methyl-1-(2-((trimethylsilyl)ethynyl)pyrimidin-5-yl)propan-1-ol **TMSPym-OH** (ee > 99.9%) and 40 mM iPr_2Zn).

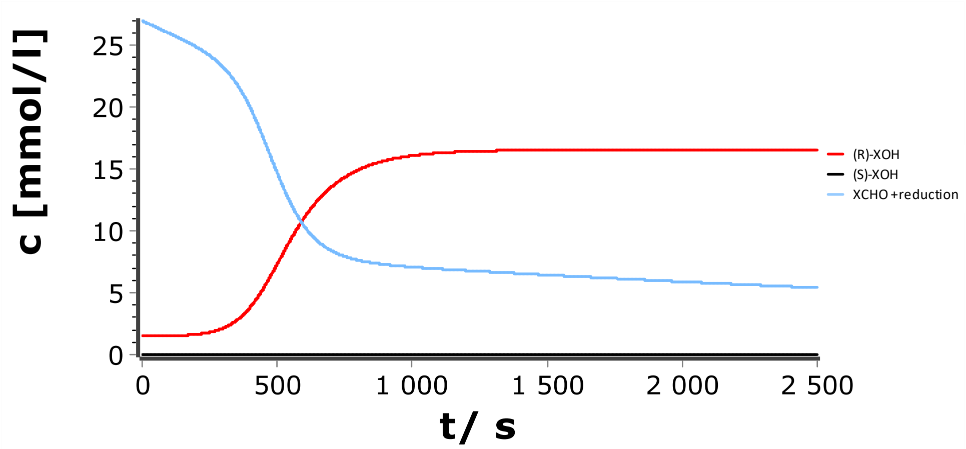

Supplementary Figure 156: Simulated concentration-time profile of the Soai reaction and the reduction side reaction (27 mM 2-((trimethylsilyl)ethynyl)pyrimidine-5-carbaldehyde **TMSPym-CHO**, 1.5 mM (1R)-2-methyl-1-(2-((trimethylsilyl)ethynyl)pyrimidin-5-yl)propan-1-ol **TMSPym-OH** (ee > 99.9%) and 40 mM iPr_2Zn).

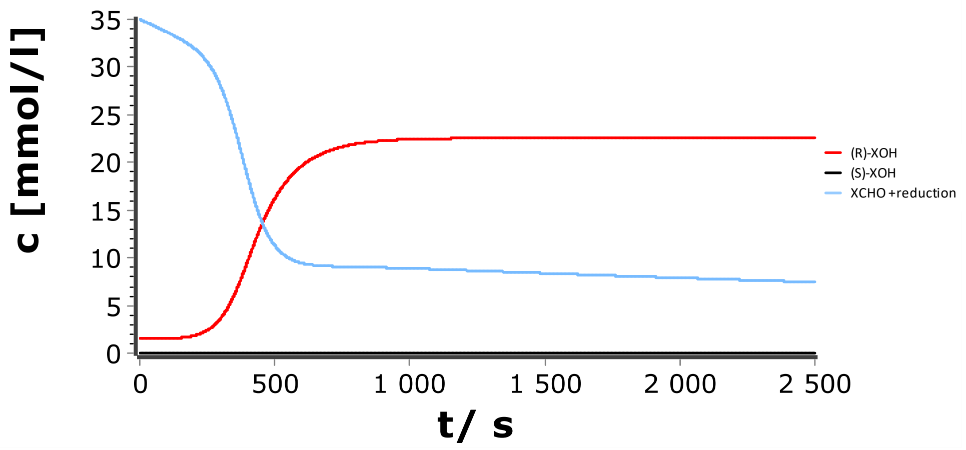

Supplementary Figure 157: Simulated concentration-time profile of the Soai reaction and the reduction side reaction (35 mM 2-((trimethylsilyl)ethynyl)pyrimidine-5-carbaldehyde **TMSPym-CHO**, 1.5 mM (1R)-2-methyl-1-(2-((trimethylsilyl)ethynyl)pyrimidin-5-yl)propan-1-ol **TMSPym-OH** (ee > 99.9%) and 40 mM iPr_2Zn).

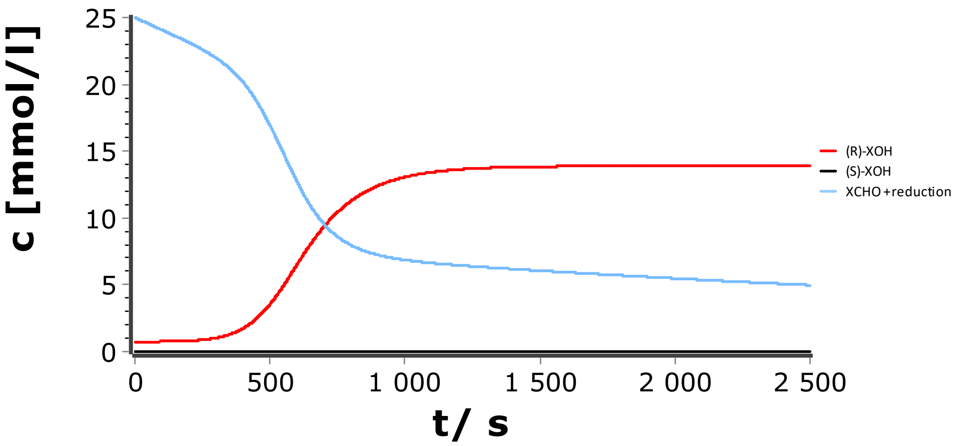

Supplementary Figure 158: Simulated concentration-time profile of the Soai reaction and the reduction side reaction (25 mM 2-((trimethylsilyl)ethynyl)pyrimidine-5-carbaldehyde **TMSPym-CHO**, 0.75 mM (1R)-2-methyl-1-(2-((trimethylsilyl)ethynyl)pyrimidin-5-yl)propan-1-ol **TMSPym-OH** (ee > 99.9%) and 40 mM iPr_2Zn).

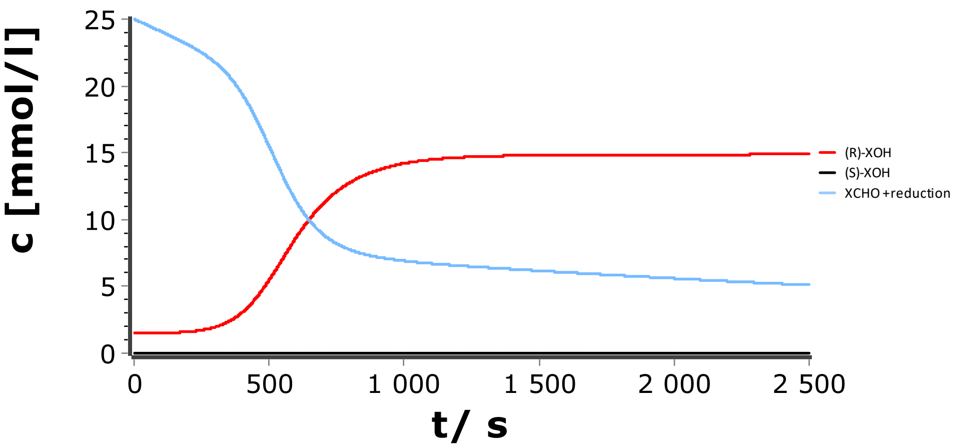

Supplementary Figure 159: Simulated concentration-time profile of the Soai reaction and the reduction side reaction (25 mM 2-((trimethylsilyl)ethynyl)pyrimidine-5-carbaldehyde **TMSPym-CHO**, 1.5 mM (1*R*)-2-methyl-1-(2-((trimethylsilyl)ethynyl)pyrimidin-5-yl)propan-1-ol **TMSPym-OH** (*ee* > 99.9%) and 40 mM *i*Pr_2Zn).

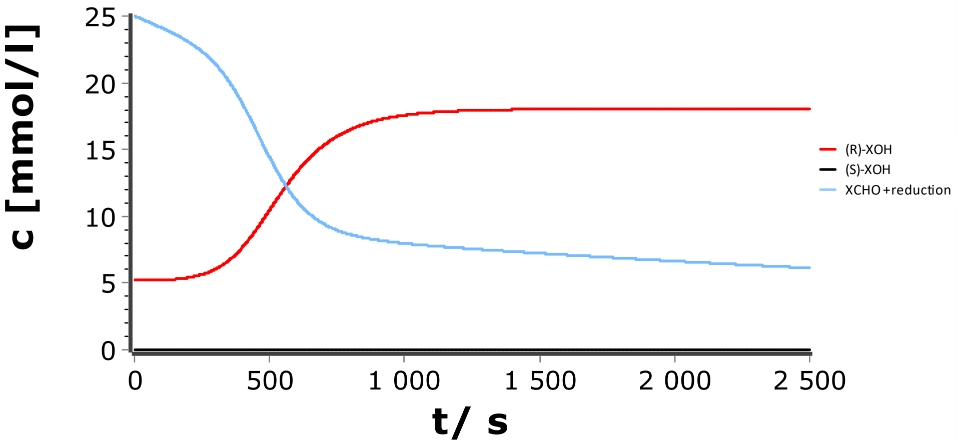

Supplementary Figure 160: Simulated concentration-time profile of the Soai reaction and the reduction side reaction (25 mM 2-((trimethylsilyl)ethynyl)pyrimidine-5-carbaldehyde **TMSPym-CHO**, 5.2 mM (1*R*)-2-methyl-1-(2-((trimethylsilyl)ethynyl)pyrimidin-5-yl)propan-1-ol **TMSPym-OH** (*ee* > 99.9%) and 40 mM *i*Pr_2Zn).

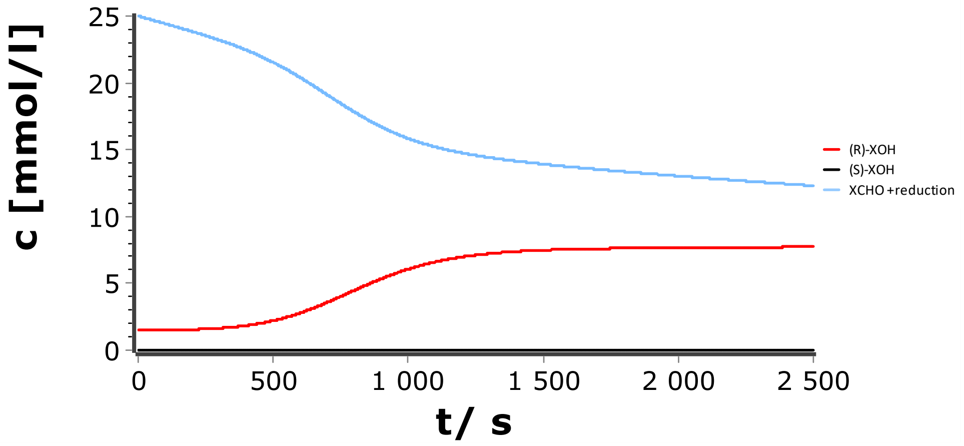

Supplementary Figure 161: Simulated concentration-time profile of the Soai reaction and the reduction side reaction (25 mM 2-((trimethylsilyl)ethynyl)pyrimidine-5-carbaldehyde **TMSPym-CHO**, 1.5 mM (1R)-2-methyl-1-(2-((trimethylsilyl)ethynyl)pyrimidin-5-yl)propan-1-ol **TMSPym-OH** (ee > 99.9%) and 25 mM iPr_2Zn).

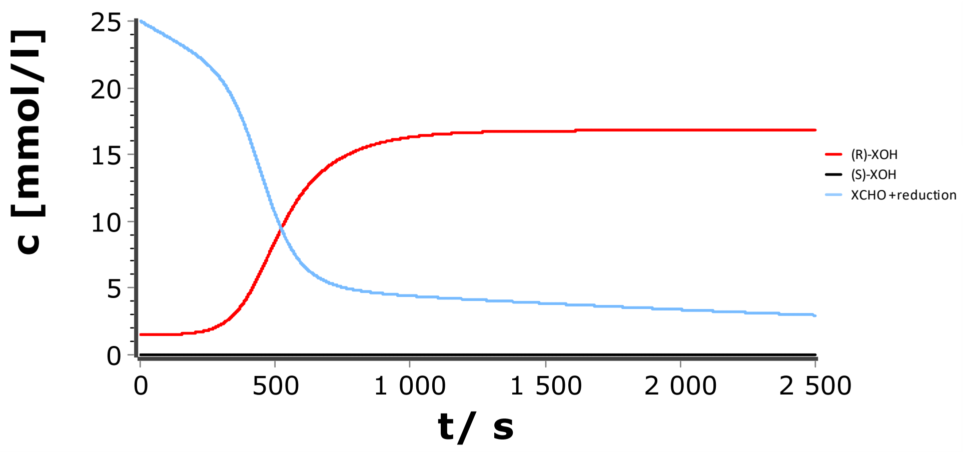

Supplementary Figure 162: Simulated concentration-time profile of the Soai reaction and the reduction side reaction (25 mM 2-((trimethylsilyl)ethynyl)pyrimidine-5-carbaldehyde **TMSPym-CHO**, 1.5 mM (1R)-2-methyl-1-(2-((trimethylsilyl)ethynyl)pyrimidin-5-yl)propan-1-ol **TMSPym-OH** (ee > 99.9%) and 50 mM iPr_2Zn).

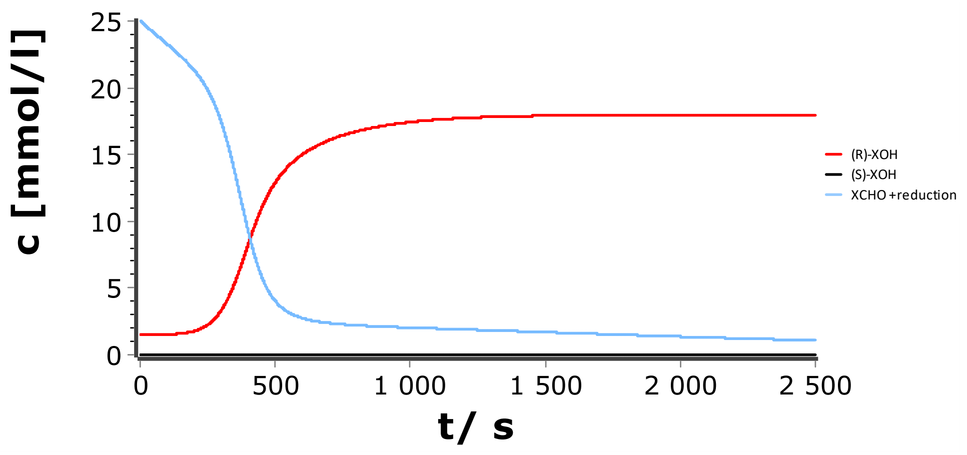

Supplementary Figure 163: Simulated concentration-time profile of the Soai reaction and the reduction side reaction (25 mM 2-((trimethylsilyl)ethynyl)pyrimidine-5-carbaldehyde **TMSPym-CHO**, 1.5 mM (1R)-2-methyl-1-(2-((trimethylsilyl)ethynyl)pyrimidin-5-yl)propan-1-ol **TMSPym-OH** (ee > 99.9%) and 75 mM iPr_2Zn).

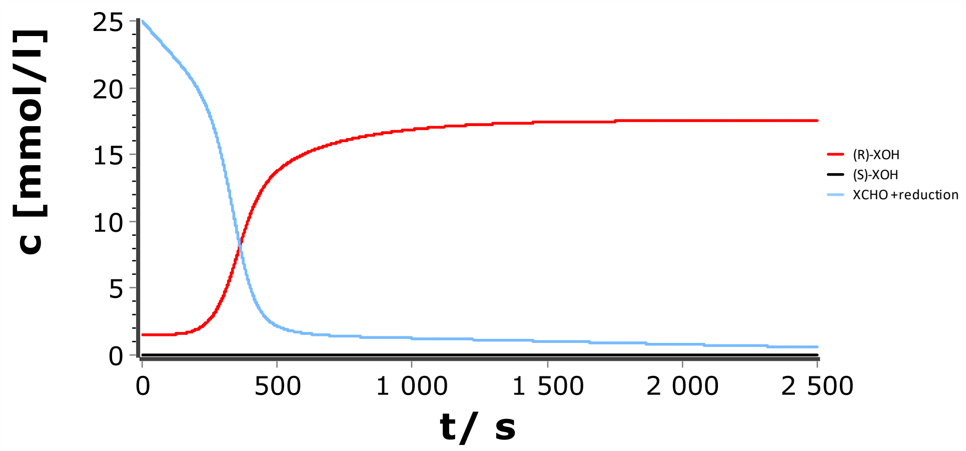

Supplementary Figure 164: Simulated concentration-time profile of the Soai reaction and the reduction side reaction (25 mM 2-((trimethylsilyl)ethynyl)pyrimidine-5-carbaldehyde **TMSPym-CHO**, 1.5 mM (1R)-2-methyl-1-(2-((trimethylsilyl)ethynyl)pyrimidin-5-yl)propan-1-ol **TMSPym-OH** (ee > 99.9%) and 100 mM iPr_2Zn).

6.4.4 AdPym-CHO/AdPym-OH System

Supplementary Table 7: Simulation Parameters for the **AdPym-CHO**/**AdPym-OH** System**.**

	AdPymCHO [mmol/L]	(*R*)-AdPymOH [mmol/L]	(*S*)-AdPymOH [mmol/L]	ee [%]	er	ZniPr$_2$ [mmol/L]	t [s]
**1**	18	1.49925	0.00075	99.9	1999	40	4000
**2**	25	1.49925	0.00075	99.9	1999	40	4000
**3**	45	1.49925	0.00075	99.9	1999	40	4000
**4**	50	1.49925	0.00075	99.9	1999	40	4000
**5**	20	0.9995	0.0005	99.9	1999	40	4000
**6**	20	1.999	0.001	99.9	1999	40	4000
**7**	20	3.1984	0.0016	99.9	1999	40	4000
**8**	20	1.49925	0.00075	99.9	1999	25	4000
**9**	20	1.49925	0.00075	99.9	1999	50	4000
**10**	20	1.49925	0.00075	99.9	1999	75	4000
**11**	20	1.49925	0.00075	99.9	1999	100	4000

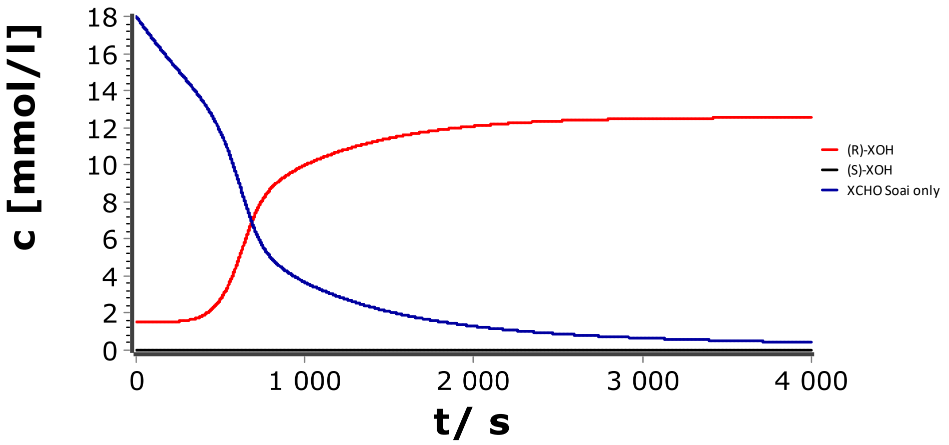

Supplementary Figure 165: Simulated concentration-time profile of the Soai reaction (18 mM 2-((adamantan-1-yl)ethynyl)pyrimidine-5-carbaldehyde **AdPym-CHO**, 1.5 mM (1R)-1-(2-((adamantan-1-yl)ethynyl)pyrimidin-5-yl)-2-methylpropan-1-ol **AdPym-OH** (ee > 99.9%) and 40 mM iPr$_2$Zn).

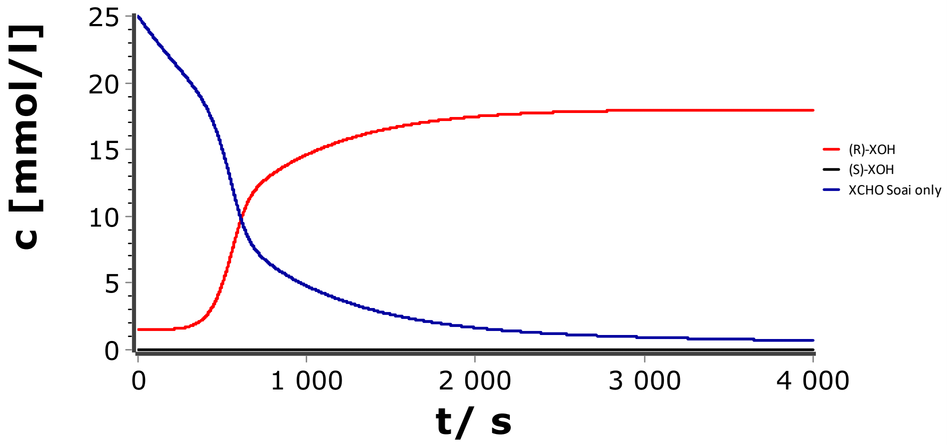

Supplementary Figure 166: Simulated concentration-time profile of the Soai reaction (25 mM 2-((adamantan-1-yl)ethynyl)pyrimidine-5-carbaldehyde **AdPym-CHO**, 1.5 mM (1R)-1-(2-((adamantan-1-yl)ethynyl)pyrimidin-5-yl)-2-methylpropan-1-ol **AdPym-OH** (ee > 99.9%) and 40 mM iPr$_2$Zn).

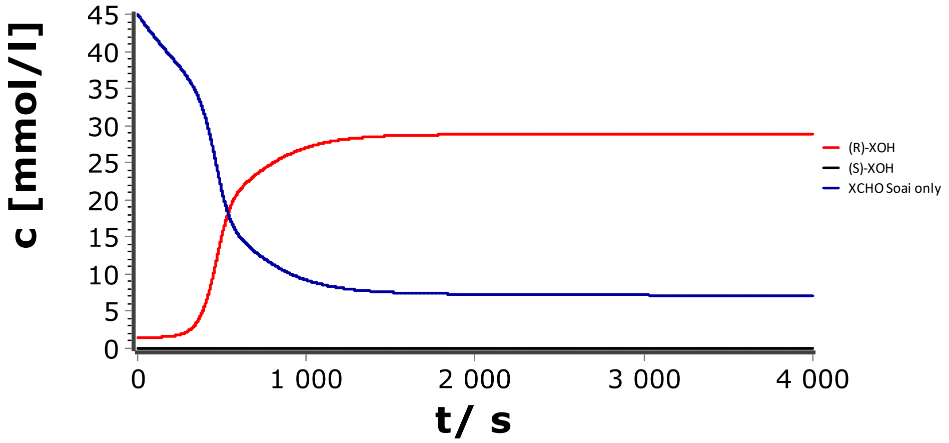

Supplementary Figure 167: Simulated concentration-time profile of the Soai reaction (45 mM 2-((adamantan-1-yl)ethynyl)pyrimidine-5-carbaldehyde **AdPym-CHO**, 1.5 mM (1*R*)-1-(2-((adamantan-1-yl)ethynyl)pyrimidin-5-yl)-2-methylpropan-1-ol **AdPym-OH** (*ee* > 99.9%) and 40 mM *i*Pr$_2$Zn).

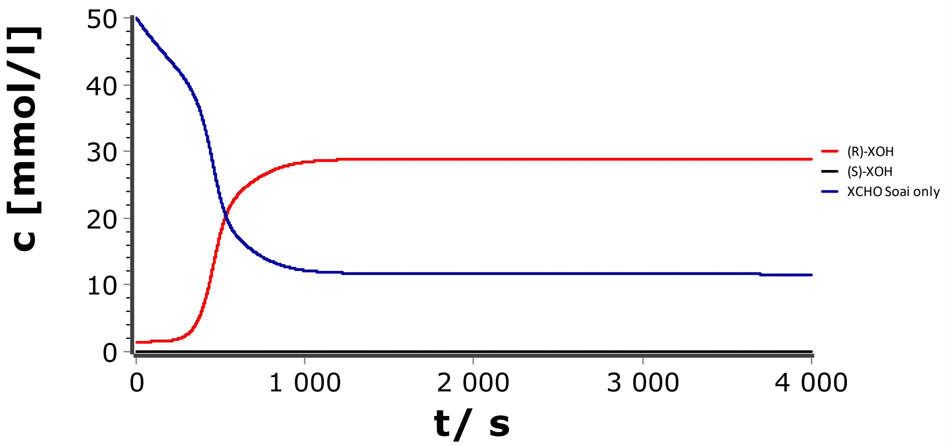

Supplementary Figure 168: Simulated concentration-time profile of the Soai reaction (50 mM 2-((adamantan-1-yl)ethynyl)pyrimidine-5-carbaldehyde **AdPym-CHO**, 1.5 mM (1R)-1-(2-((adamantan-1-yl)ethynyl)pyrimidin-5-yl)-2-methylpropan-1-ol **AdPym-OH** (ee > 99.9%) and 40 mM iPr_2Zn).

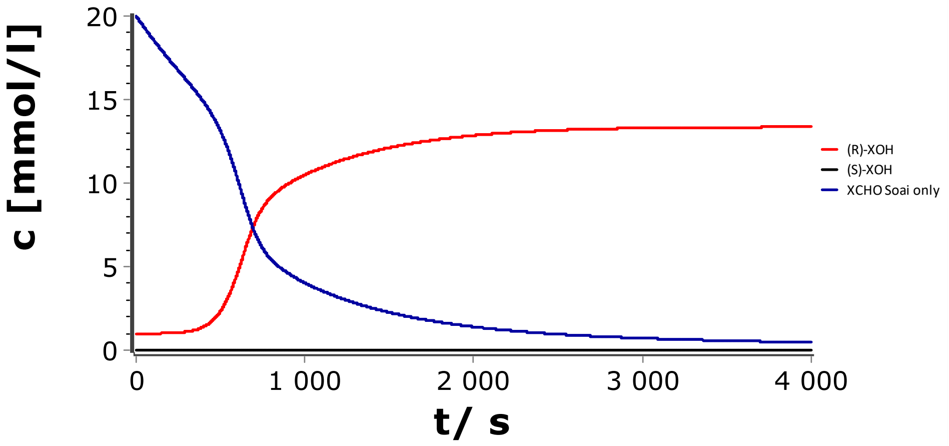

Supplementary Figure 169: Simulated concentration-time profile of the Soai reaction (20 mM 2-((adamantan-1-yl)ethynyl)pyrimidine-5-carbaldehyde **AdPym-CHO**, 1.0 mM (1R)-1-(2-((adamantan-1-yl)ethynyl)pyrimidin-5-yl)-2-methylpropan-1-ol **AdPym-OH** (ee > 99.9%) and 40 mM iPr_2Zn).

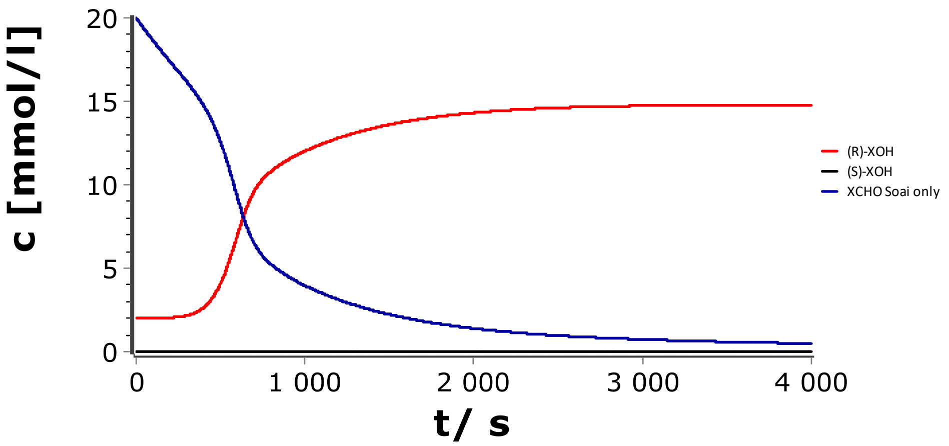

Supplementary Figure 170: Simulated concentration-time profile of the Soai reaction (20 mM 2-((adamantan-1-yl)ethynyl)pyrimidine-5-carbaldehyde **AdPym-CHO**, 2.0 mM (1R)-1-(2-((adamantan-1-yl)ethynyl)pyrimidin-5-yl)-2-methylpropan-1-ol **AdPym-OH** (ee > 99.9%) and 40 mM iPr_2Zn).

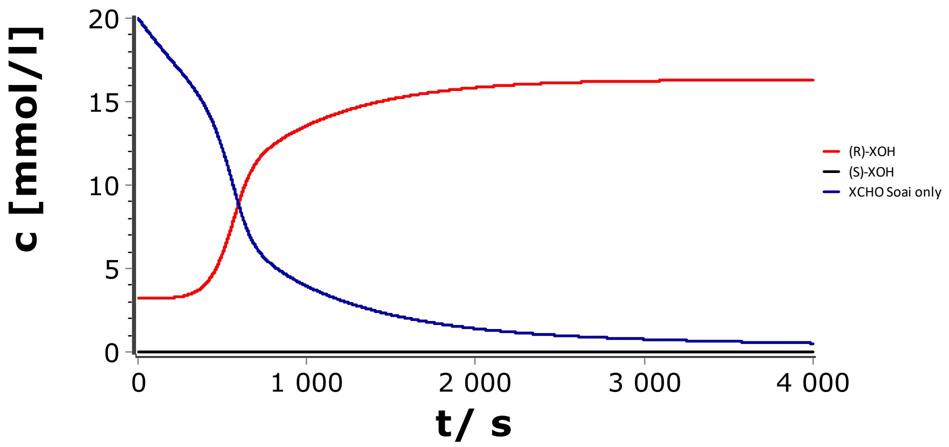

Supplementary Figure 171: Simulated concentration-time profile of the Soai reaction (20 mM 2-((adamantan-1-yl)ethynyl)pyrimidine-5-carbaldehyde **AdPym-CHO**, 3.2 mM (1R)-1-(2-((adamantan-1-yl)ethynyl)pyrimidin-5-yl)-2-methylpropan-1-ol **AdPym-OH** (ee > 99.9%) and 40 mM iPr_2Zn).

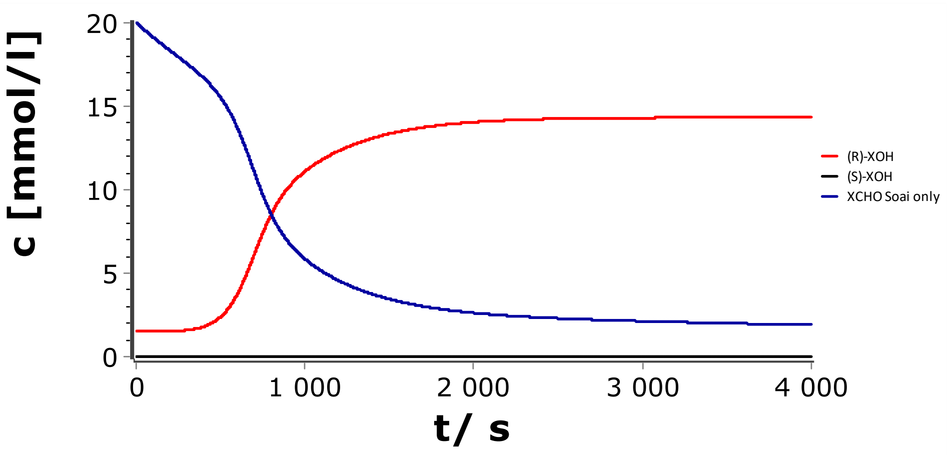

Supplementary Figure 172: Simulated concentration-time profile of the Soai reaction (20 mM 2-((adamantan-1-yl)ethynyl)pyrimidine-5-carbaldehyde **AdPym-CHO**, 1.5 mM (1*R*)-1-(2-((adamantan-1-yl)ethynyl)pyrimidin-5-yl)-2-methylpropan-1-ol **AdPym-OH** (*ee* > 99.9%) and 25 mM *i*Pr_2Zn).

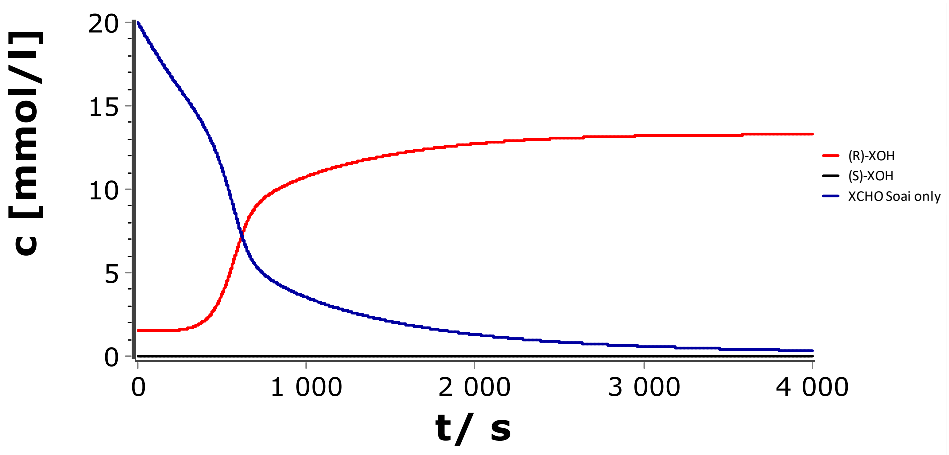

Supplementary Figure 173: Simulated concentration-time profile of the Soai reaction (20 mM 2-((adamantan-1-yl)ethynyl)pyrimidine-5-carbaldehyde **AdPym-CHO**, 1.5 mM (1R)-1-(2-((adamantan-1-yl)ethynyl)pyrimidin-5-yl)-2-methylpropan-1-ol **AdPym-OH** (ee > 99.9%) and 50 mM iPr_2Zn).

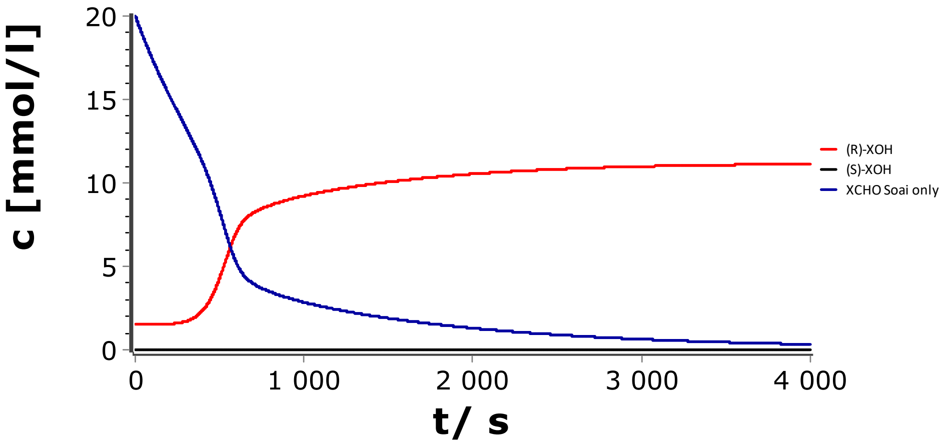

Supplementary Figure 174: Simulated concentration-time profile of the Soai reaction (20 mM 2-((adamantan-1-yl)ethynyl)pyrimidine-5-carbaldehyde **AdPym-CHO**, 1.5 mM (1R)-1-(2-((adamantan-1-yl)ethynyl)pyrimidin-5-yl)-2-methylpropan-1-ol **AdPym-OH** (ee > 99.9%) and 75 mM iPr_2Zn).

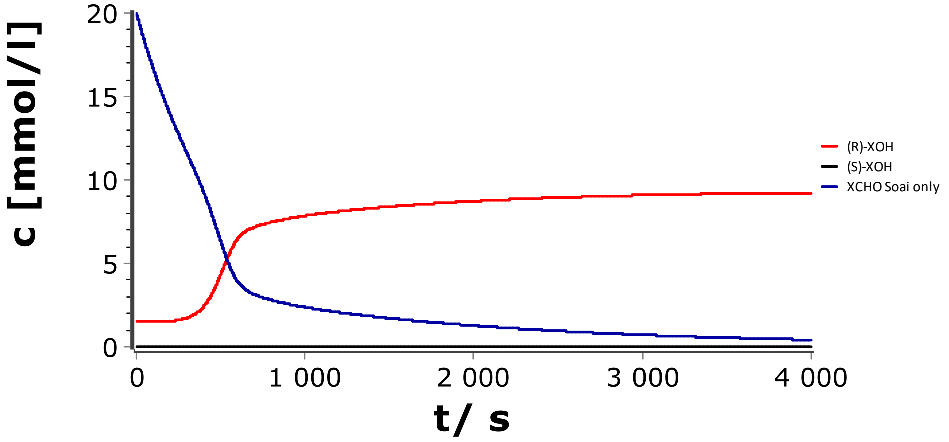

Supplementary Figure 175: Simulated concentration-time profile of the Soai reaction (20 mM 2-((adamantan-1-yl)ethynyl)pyrimidine-5-carbaldehyde **AdPym-CHO**, 1.5 mM (1R)-1-(2-((adamantan-1-yl)ethynyl)pyrimidin-5-yl)-2-methylpropan-1-ol **AdPym-OH** (ee > 99.9%) and 100 mM iPr_2Zn).

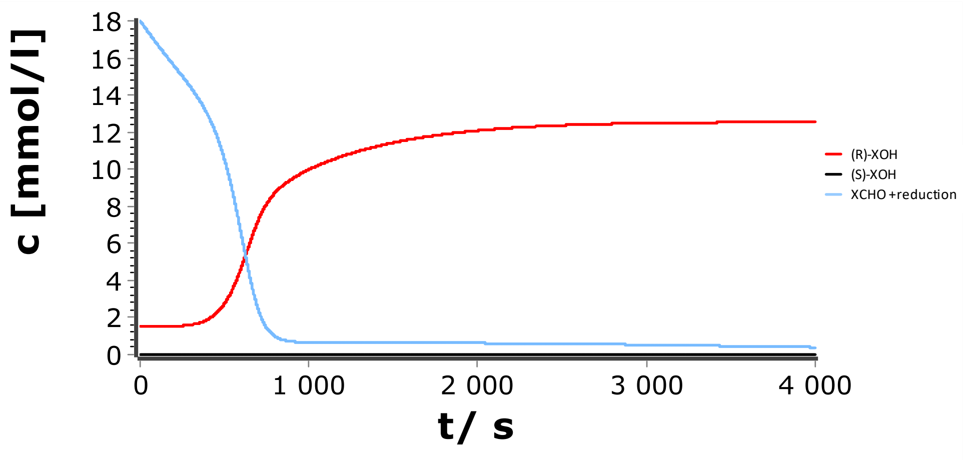

Supplementary Figure 176: Simulated concentration-time profile of the Soai reaction and the reduction side reaction (18 mM 2-((adamantan-1-yl)ethynyl)pyrimidine-5-carbaldehyde **AdPym-CHO**, 1.5 mM (1R)-1-(2-((adamantan-1-yl)ethynyl)pyrimidin-5-yl)-2-methylpropan-1-ol **AdPym-OH** (ee > 99.9%) and 40 mM iPr_2Zn).

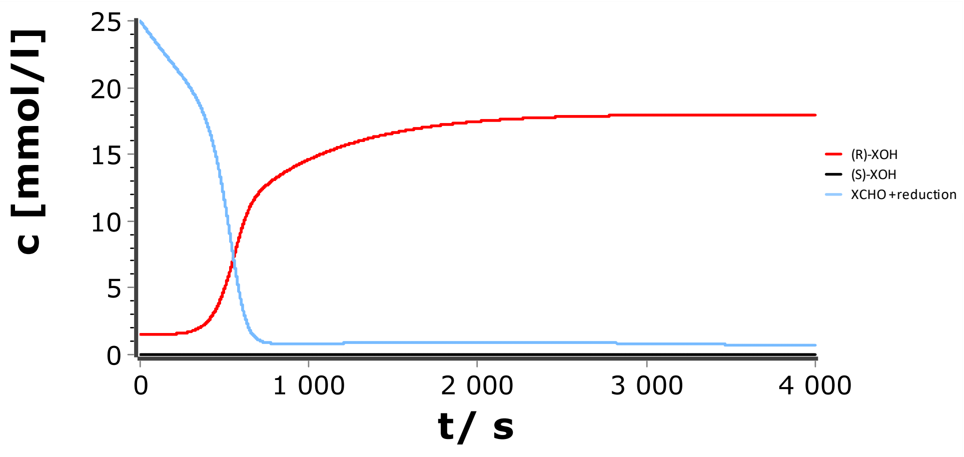

Supplementary Figure 177: Simulated concentration-time profile of the Soai reaction and the reduction side reaction (25 mM 2-((adamantan-1-yl)ethynyl)pyrimidine-5-carbaldehyde **AdPym-CHO**, 1.5 mM (1R)-1-(2-((adamantan-1-yl)ethynyl)pyrimidin-5-yl)-2-methylpropan-1-ol **AdPym-OH** (ee > 99.9%) and 40 mM iPr_2Zn).

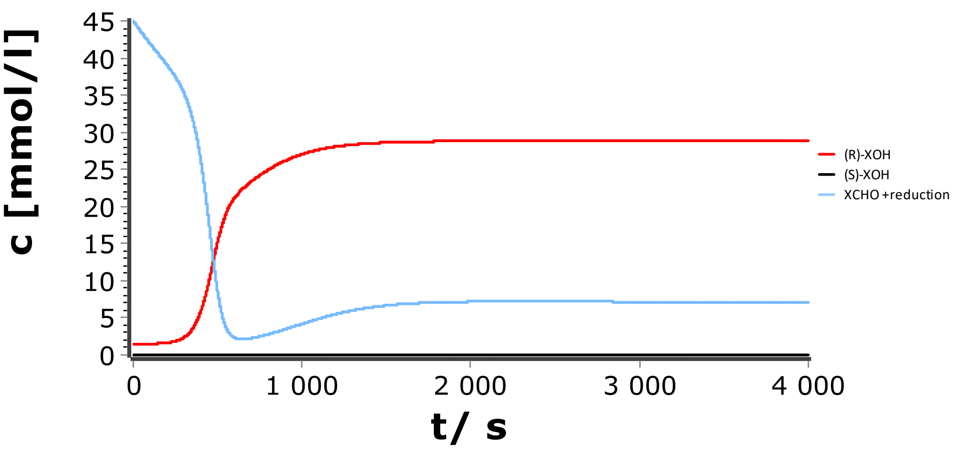

Supplementary Figure 178: Simulated concentration-time profile of the Soai reaction and the reduction side reaction (45 mM 2-((adamantan-1-yl)ethynyl)pyrimidine-5-carbaldehyde **AdPym-CHO**, 1.5 mM (1R)-1-(2-((adamantan-1-yl)ethynyl)pyrimidin-5-yl)-2-methylpropan-1-ol **AdPym-OH** (ee > 99.9%) and 40 mM iPr_2Zn).

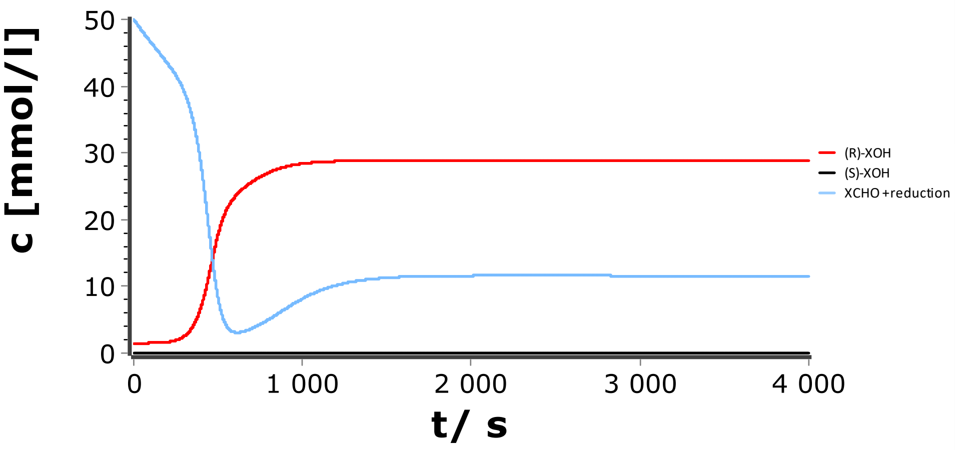

Supplementary Figure 179: Simulated concentration-time profile of the Soai reaction and the reduction side reaction (50 mM 2-((adamantan-1-yl)ethynyl)pyrimidine-5-carbaldehyde **AdPym-CHO**, 1.5 mM (1R)-1-(2-((adamantan-1-yl)ethynyl)pyrimidin-5-yl)-2-methylpropan-1-ol **AdPym-OH** (ee > 99.9%) and 40 mM iPr_2Zn).

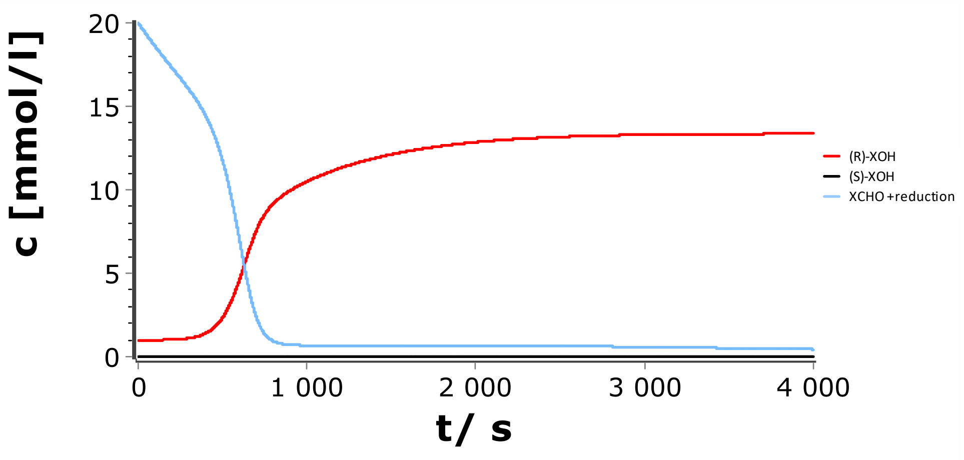

Supplementary Figure 180: Simulated concentration-time profile of the Soai reaction and the reduction side reaction (20 mM 2-((adamantan-1-yl)ethynyl)pyrimidine-5-carbaldehyde **AdPym-CHO**, 1.0 mM (1R)-1-(2-((adamantan-1-yl)ethynyl)pyrimidin-5-yl)-2-methylpropan-1-ol **AdPym-OH** (ee > 99.9%) and 40 mM iPr_2Zn).

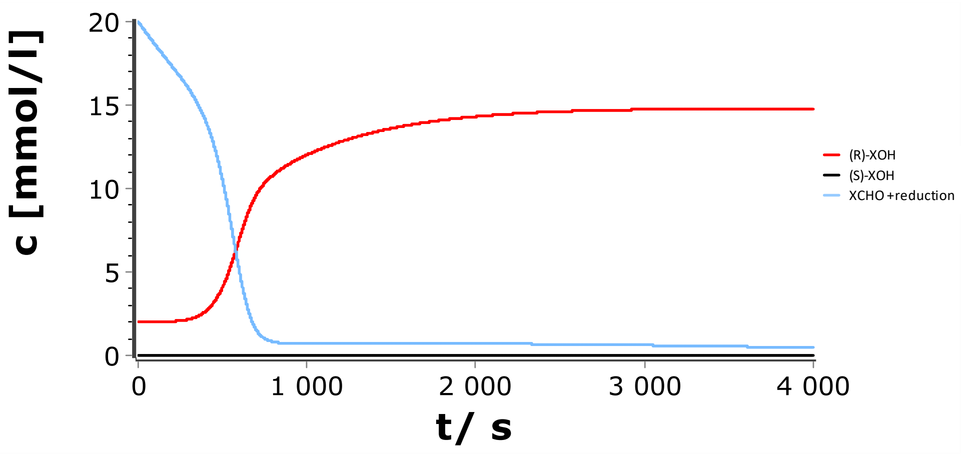

Supplementary Figure 181: Simulated concentration-time profile of the Soai reaction and the reduction side reaction (20 mM 2-((adamantan-1-yl)ethynyl)pyrimidine-5-carbaldehyde **AdPym-CHO**, 2.0 mM (1R)-1-(2-((adamantan-1-yl)ethynyl)pyrimidin-5-yl)-2-methylpropan-1-ol **AdPym-OH** (ee > 99.9%) and 40 mM iPr_2Zn).

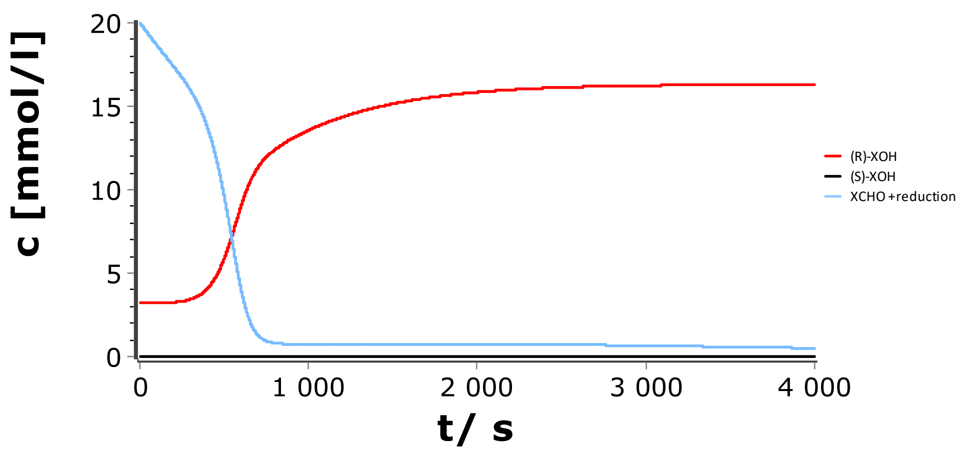

Supplementary Figure 182: Simulated concentration-time profile of the Soai reaction and the reduction side reaction (20 mM 2-((adamantan-1-yl)ethynyl)pyrimidine-5-carbaldehyde **AdPym-CHO**, 3.2 mM (1R)-1-(2-((adamantan-1-yl)ethynyl)pyrimidin-5-yl)-2-methylpropan-1-ol **AdPym-OH** (ee > 99.9%) and 40 mM iPr_2Zn).

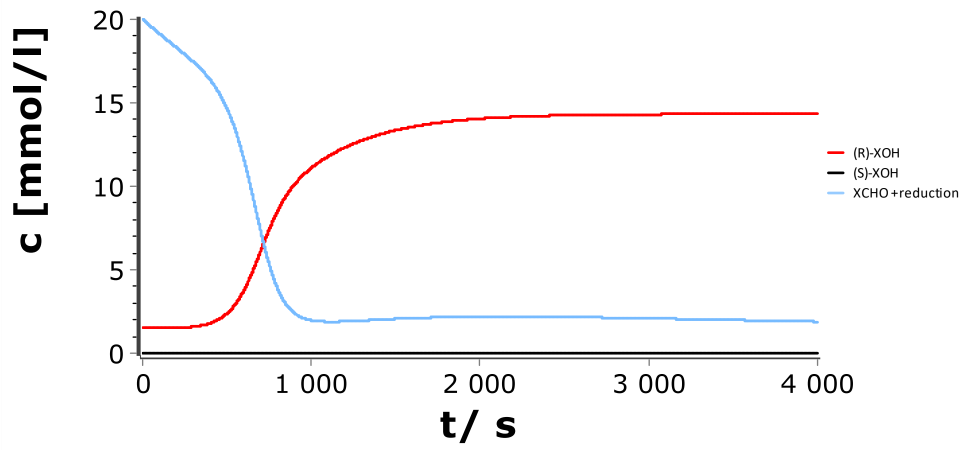

Supplementary Figure 183: Simulated concentration-time profile of the Soai reaction and the reduction side reaction (20 mM 2-((adamantan-1-yl)ethynyl)pyrimidine-5-carbaldehyde **AdPym-CHO**, 1.5 mM (1R)-1-(2-((adamantan-1-yl)ethynyl)pyrimidin-5-yl)-2-methylpropan-1-ol **AdPym-OH** (ee > 99.9%) and 25 mM iPr_2Zn).

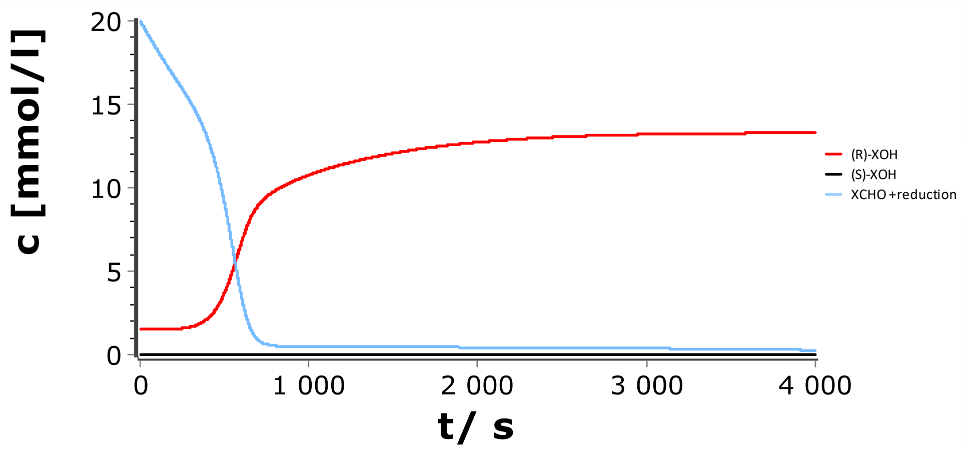

Supplementary Figure 184: Simulated concentration-time profile of the Soai reaction and the reduction side reaction (20 mM 2-((adamantan-1-yl)ethynyl)pyrimidine-5-carbaldehyde **AdPym-CHO**, 1.5 mM (1R)-1-(2-((adamantan-1-yl)ethynyl)pyrimidin-5-yl)-2-methylpropan-1-ol **AdPym-OH** (ee > 99.9%) and 50 mM iPr_2Zn).

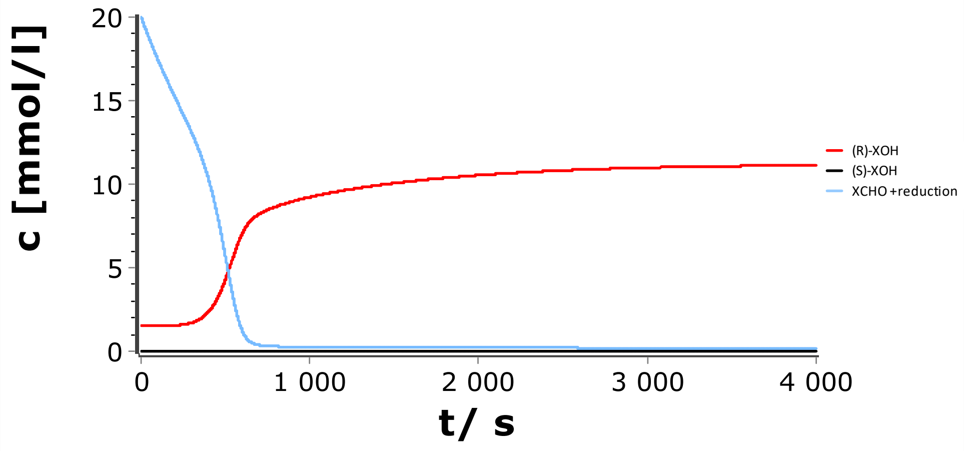

Supplementary Figure 185: Simulated concentration-time profile of the Soai reaction and the reduction side reaction (20 mM 2-((adamantan-1-yl)ethynyl)pyrimidine-5-carbaldehyde **AdPym-CHO**, 1.5 mM (1R)-1-(2-((adamantan-1-yl)ethynyl)pyrimidin-5-yl)-2-methylpropan-1-ol **AdPym-OH** (ee > 99.9%) and 75 mM iPr_2Zn).

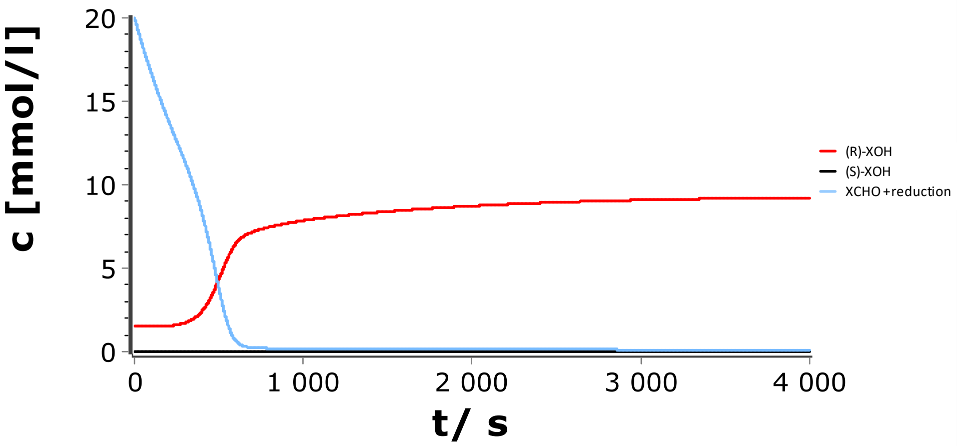

Supplementary Figure 186: Simulated concentration-time profile of the Soai reaction and the reduction side reaction (20 mM 2-((adamantan-1-yl)ethynyl)pyrimidine-5-carbaldehyde **AdPym-CHO**, 1.5 mM (1R)-1-(2-((adamantan-1-yl)ethynyl)pyrimidin-5-yl)-2-methylpropan-1-ol **AdPym-OH** (ee > 99.9%) and 100 mM iPr_2Zn).

7 NMR Spectra

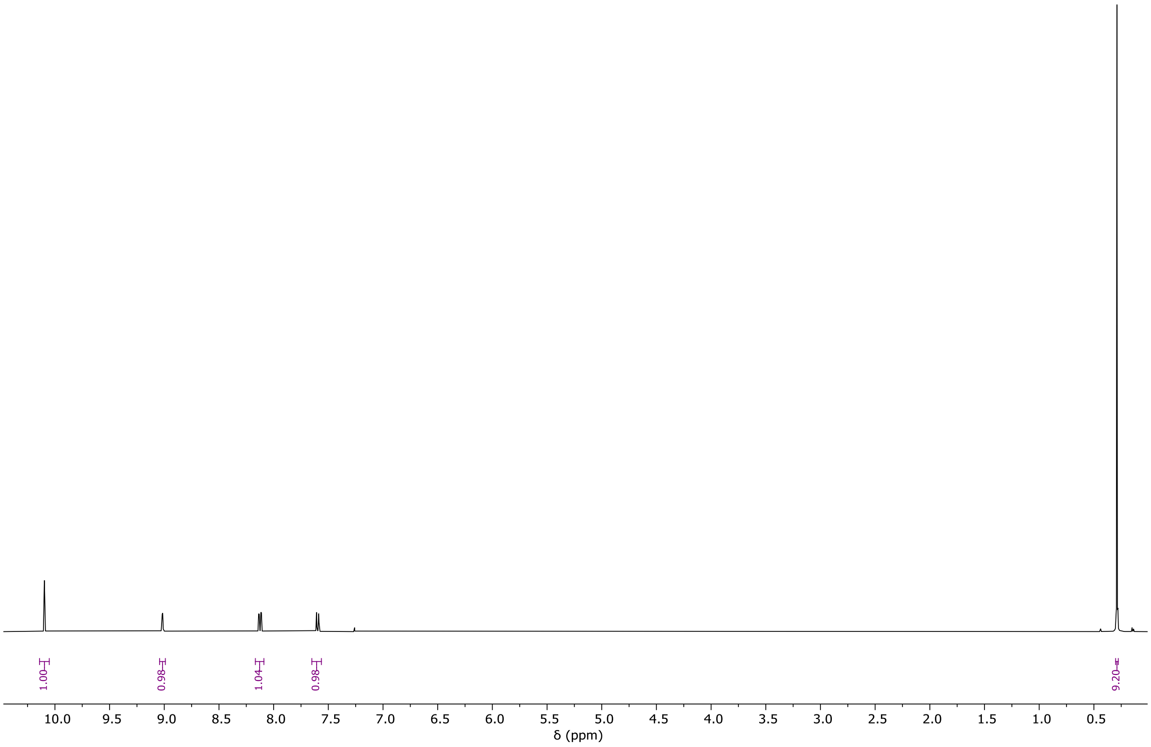

Supplementary Figure 187: ^{1}H-NMR ($CDCl_3$, 400 MHz) of 6-((trimethylsilyl)ethynyl)nicotinaldehyde (**TMSPyr-CHO**).

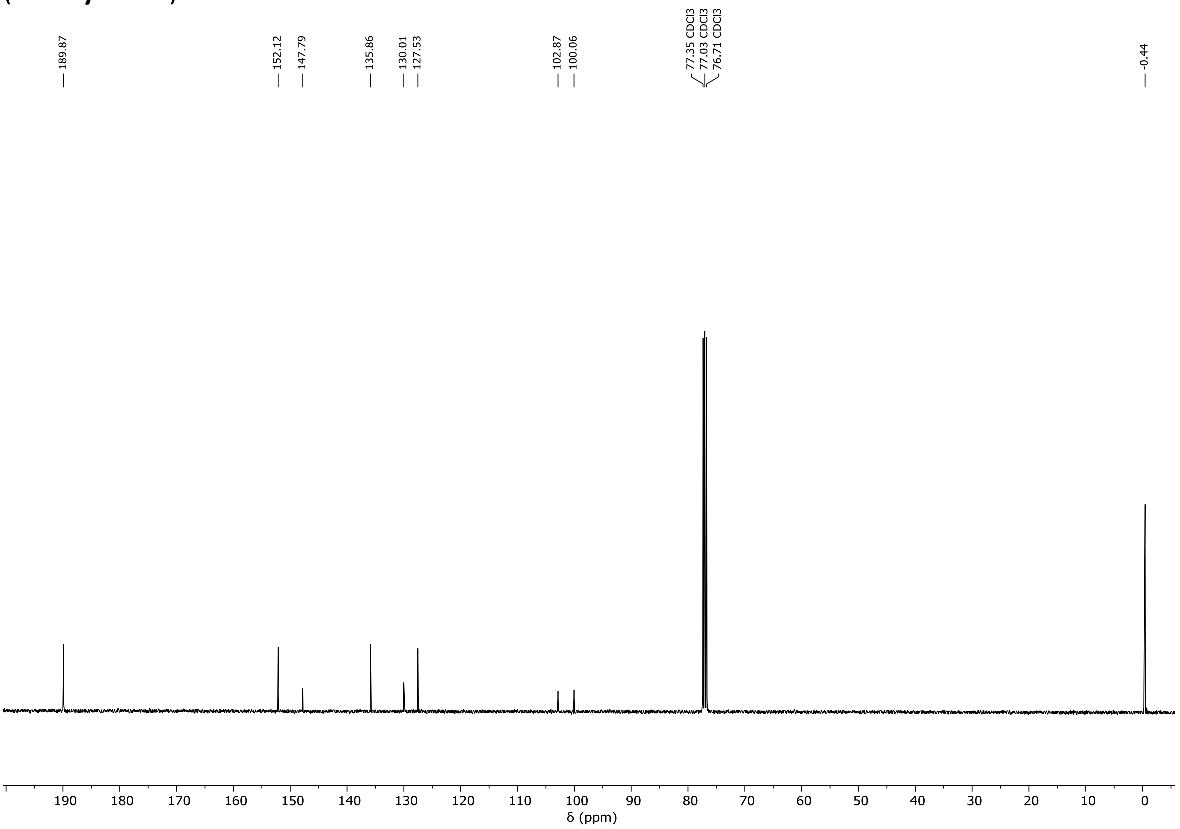

Supplementary Figure 188: ^{13}C-NMR (CDCl3, 400 MHz) of 6-((trimethylsilyl)ethynyl)nicotinaldehyde (**TMSPyr-CHO**).

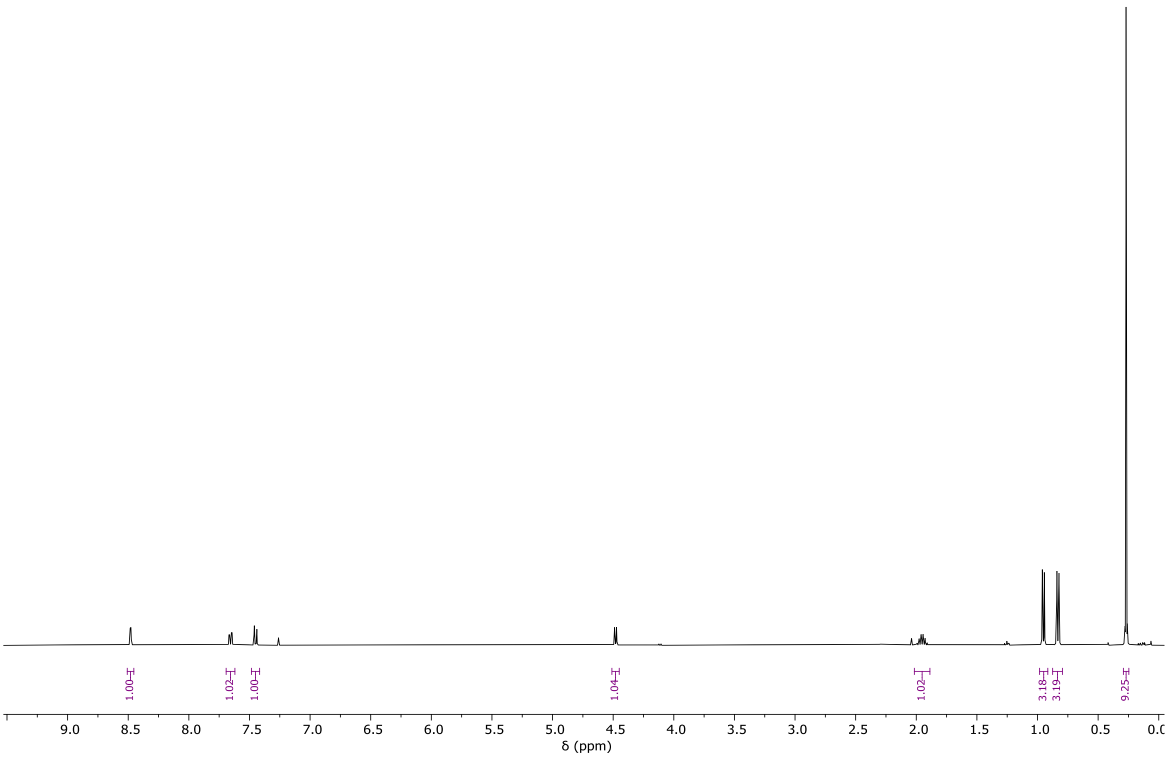

Supplementary Figure 189: ^{1}H-NMR ($CDCl_3$, 400 MHz) of 2-Methyl-(6-((trimethylsilyl)ethynyl)pyridine-3-yl)propanol (**TMSPyr-OH**).

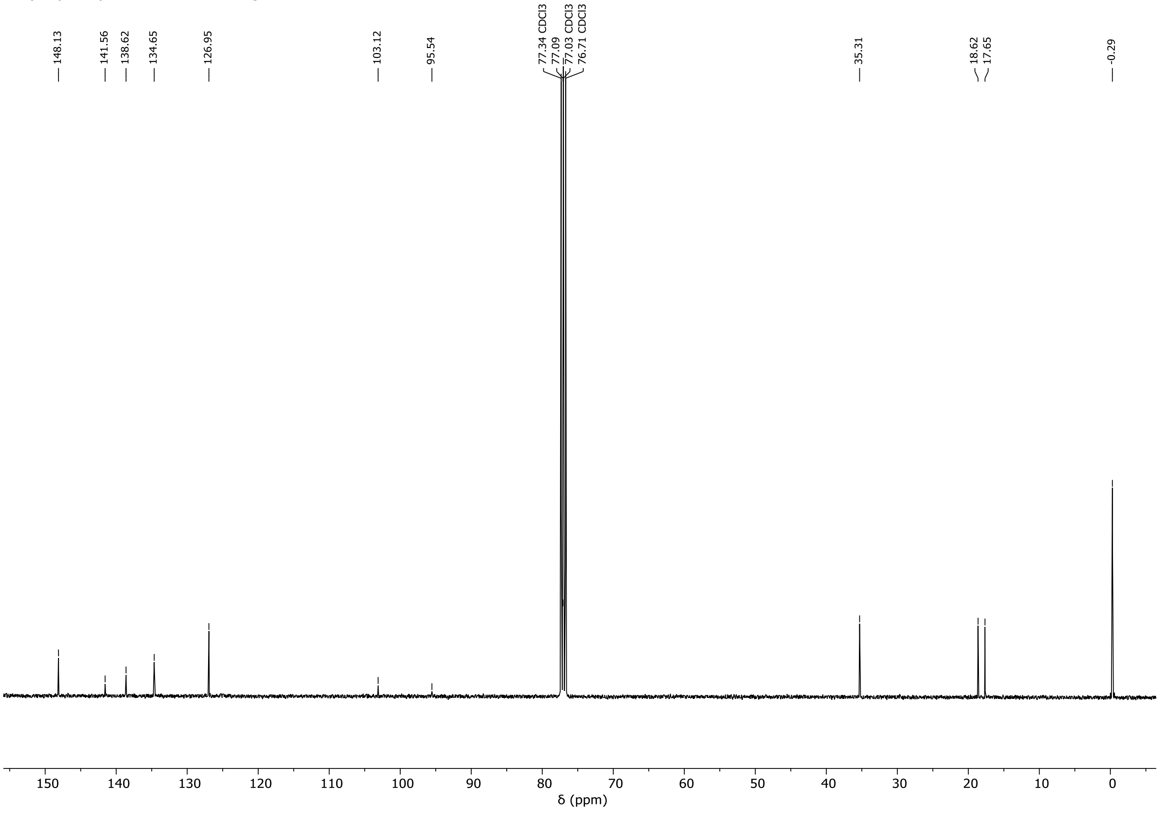

Supplementary Figure 190: ^{13}C-NMR (CDCl3, 400 MHz) of 2-Methyl-(6-((trimethylsilyl)ethynyl)pyridine-3-yl)propanol (**TMSPyr-OH**).

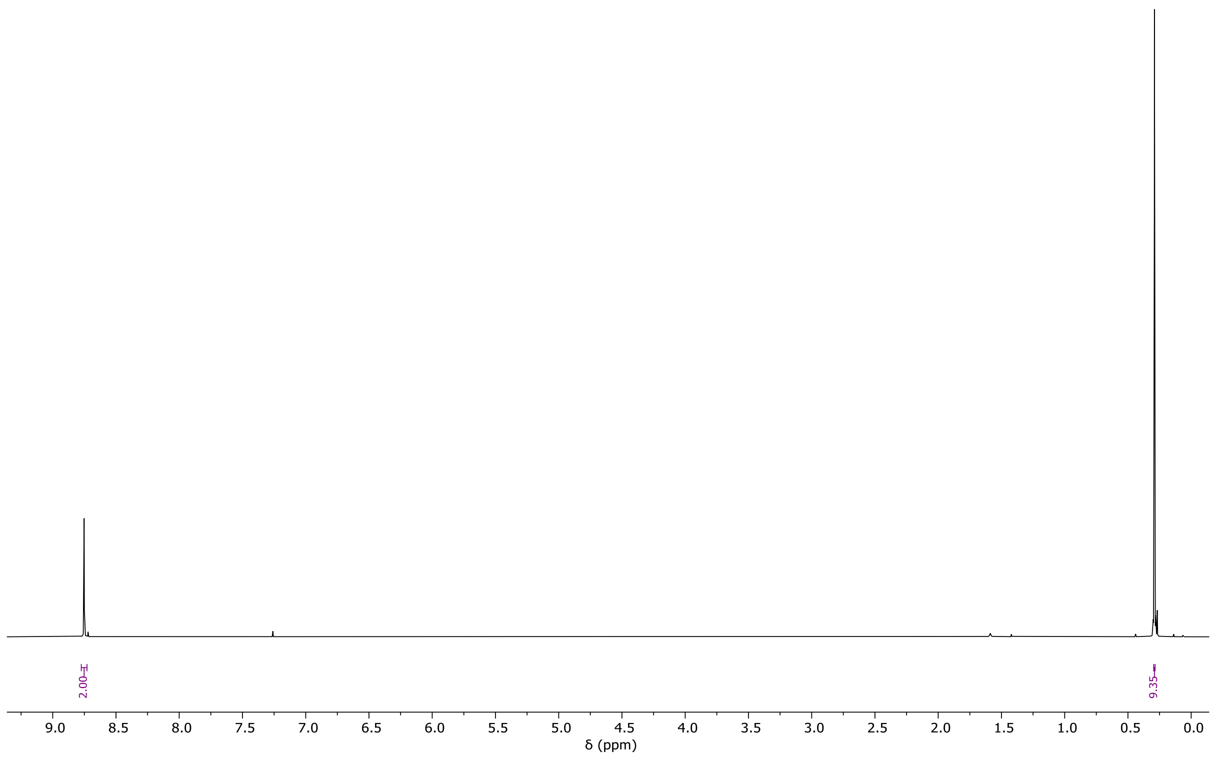

Supplementary Figure 191: ^{1}H-NMR ($CDCl_3$, 400 MHz) of 5-Bromo-2-((trimethylsilyl)ethynyl) pyrimidine (**TMSPym-Br**).

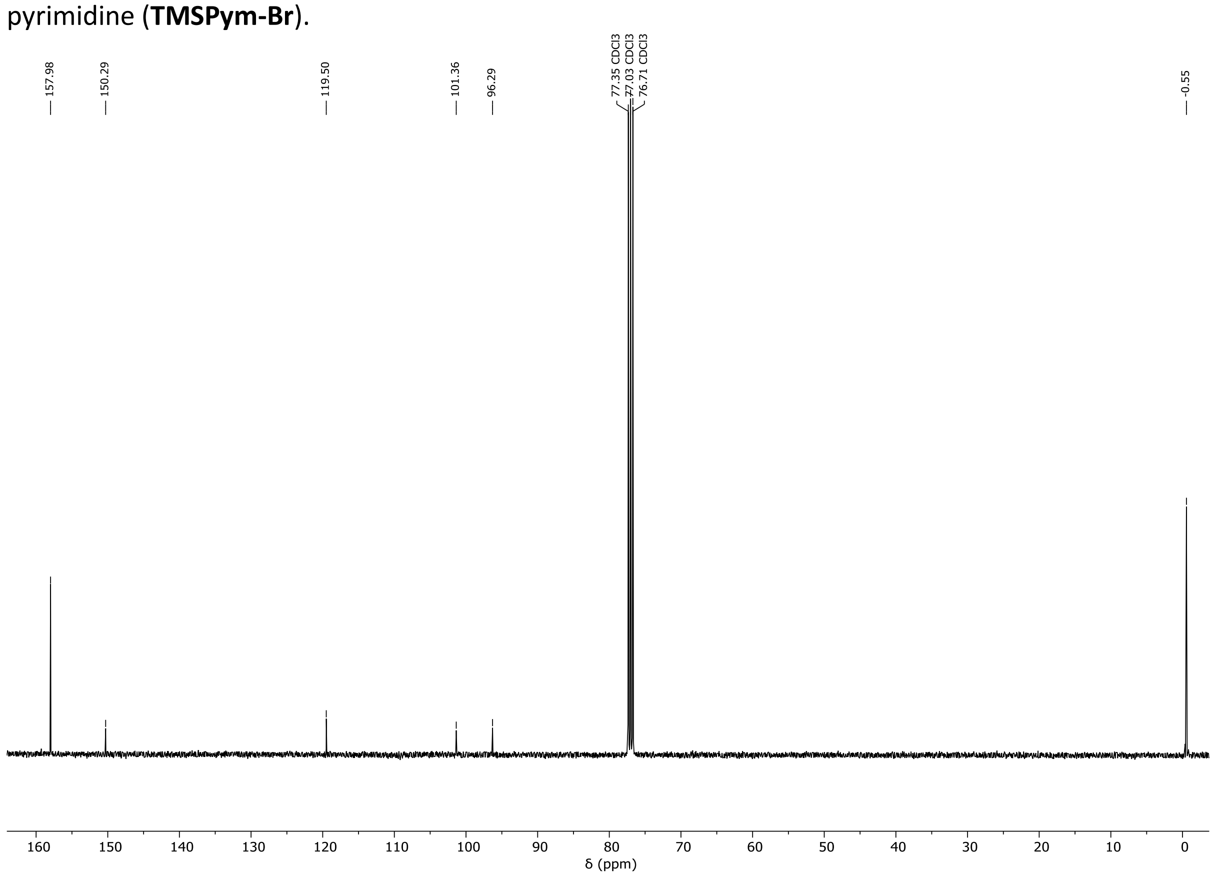

Supplementary Figure 192: ^{13}C-NMR ($CDCl_3$, 400 MHz) of 5-Bromo-2-((trimethylsilyl)ethynyl) pyrimidine (**TMSPym-Br).**

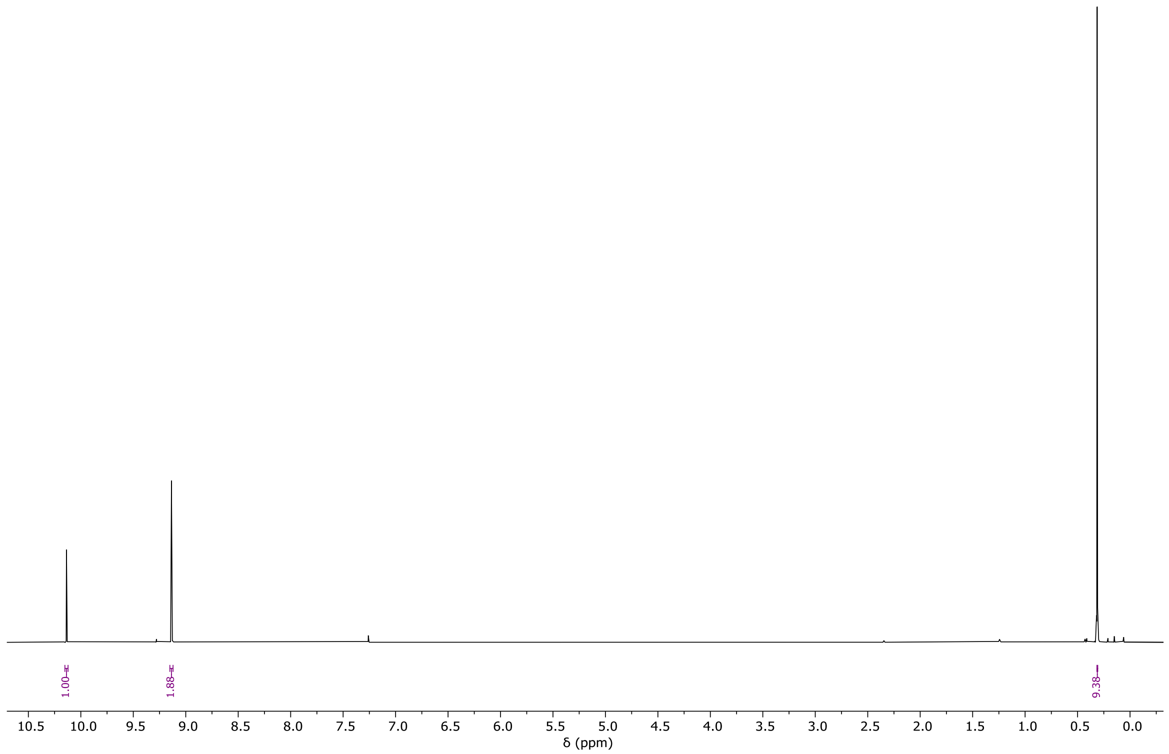

Supplementary Figure 193: ^{1}H-NMR ($CDCl_3$, 400 MHz) of 2-(Ethynyl-adamantyl)-pyrimidine-5-carbaldehyde (**AdPym-CHO**).

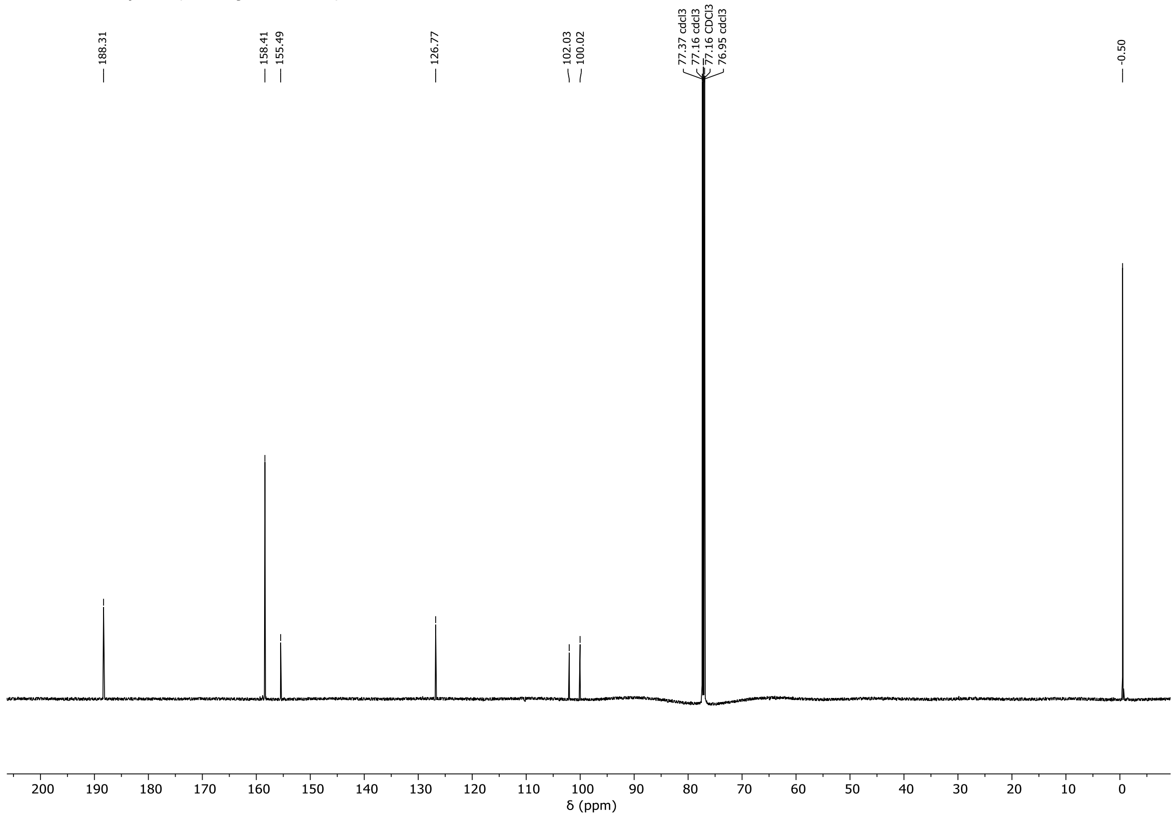

Supplementary Figure 194: ^{13}C-NMR ($CDCl_3$, 400 MHz) of 2-(Ethynyl-adamantyl)-pyrimidine-5-carbaldehyde (**AdPym-CHO**).

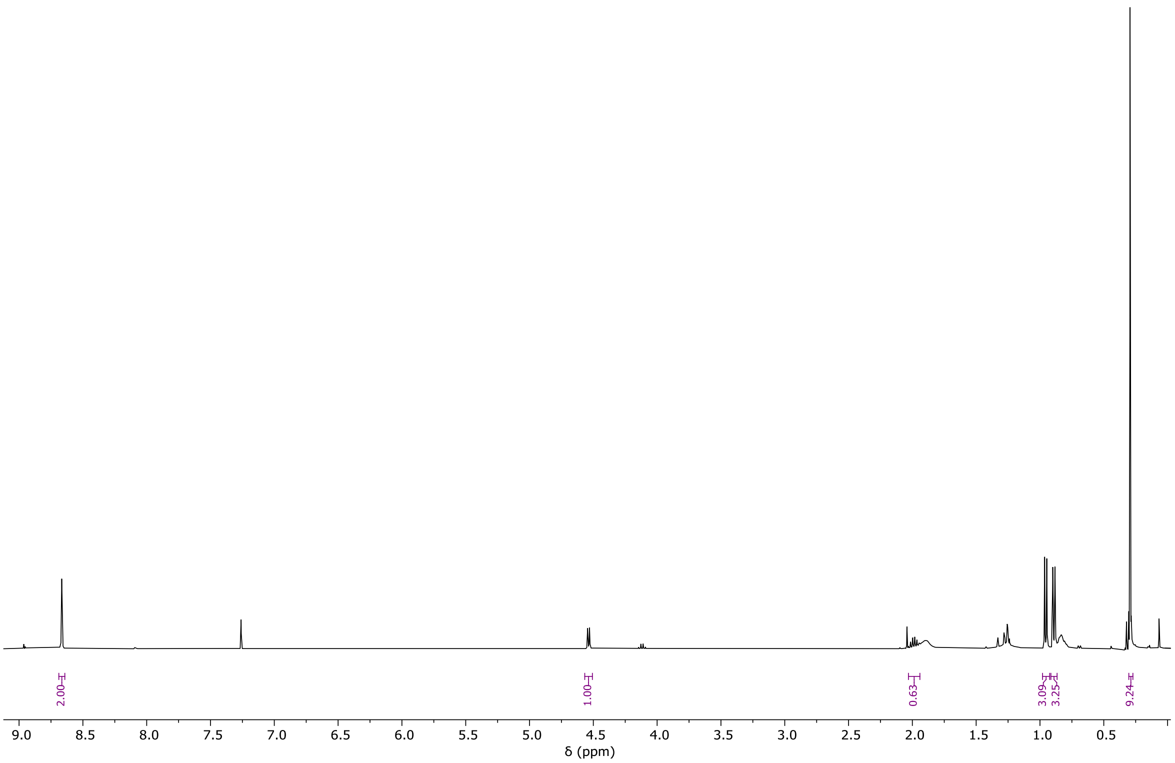

Supplementary Figure 195: ^{1}H-NMR ($CDCl_3$, 400 MHz) of 2-Methyl-((2-trimethylsilylalkynyl)-5-pyrimidinyl)propanol (**TMSPym-OH**).

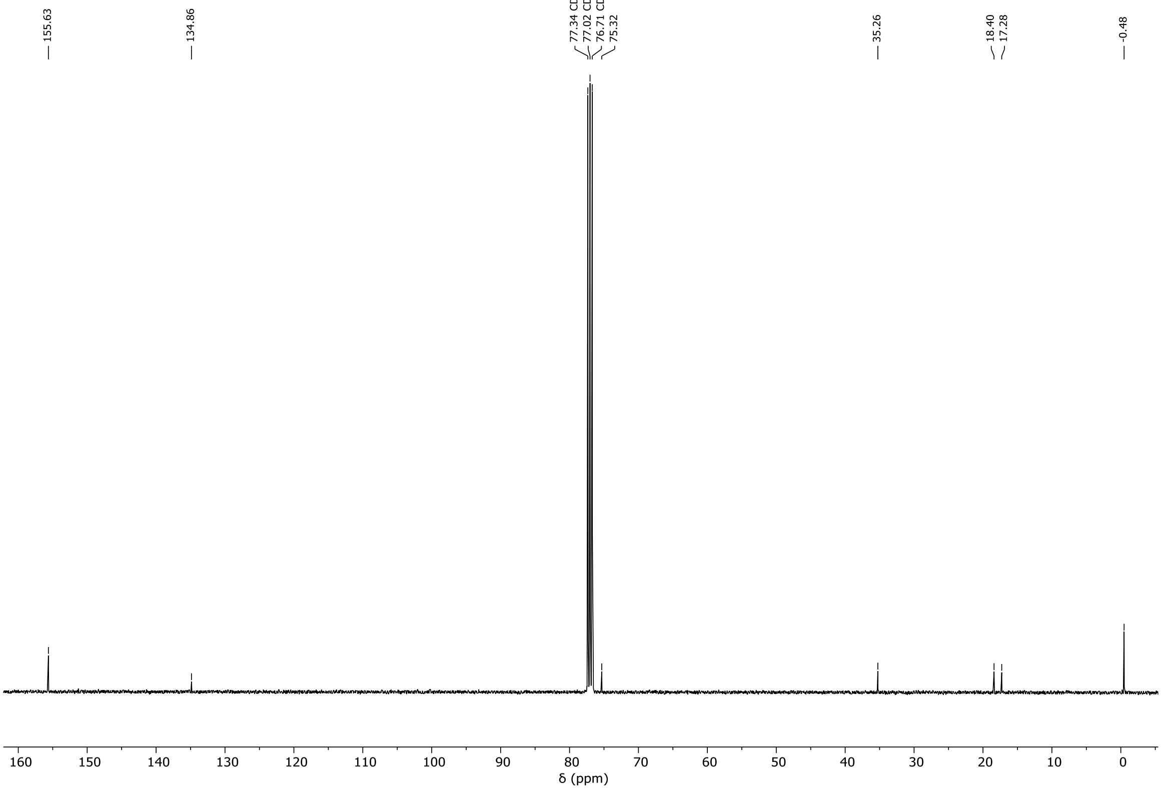

Supplementary Figure 196: ^{13}C-NMR ($CDCl_3$, 400 MHz) of 2-Methyl-((2-trimethylsilylalkynyl)-5-pyrimidinyl)propanol (**TMSPym-OH**).

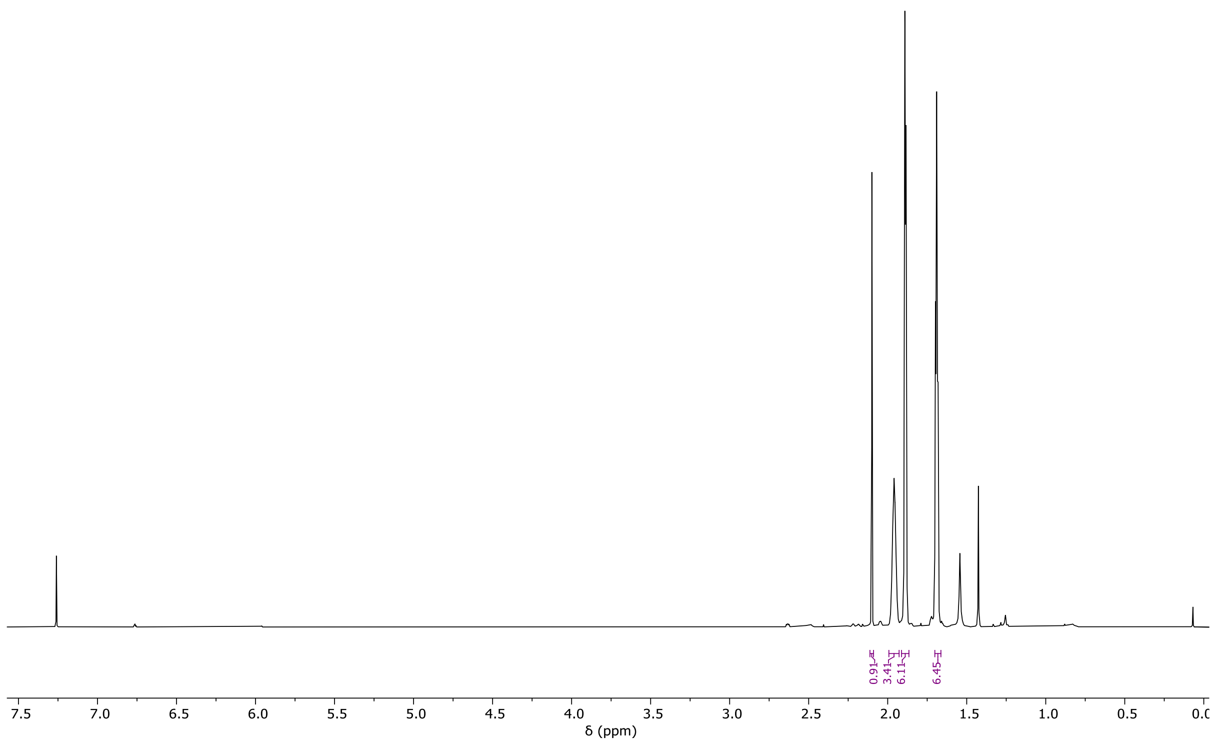

Supplementary Figure 197: ^{1}H-NMR ($CDCl_3$, 400 MHz) of ethinyl adamantane.

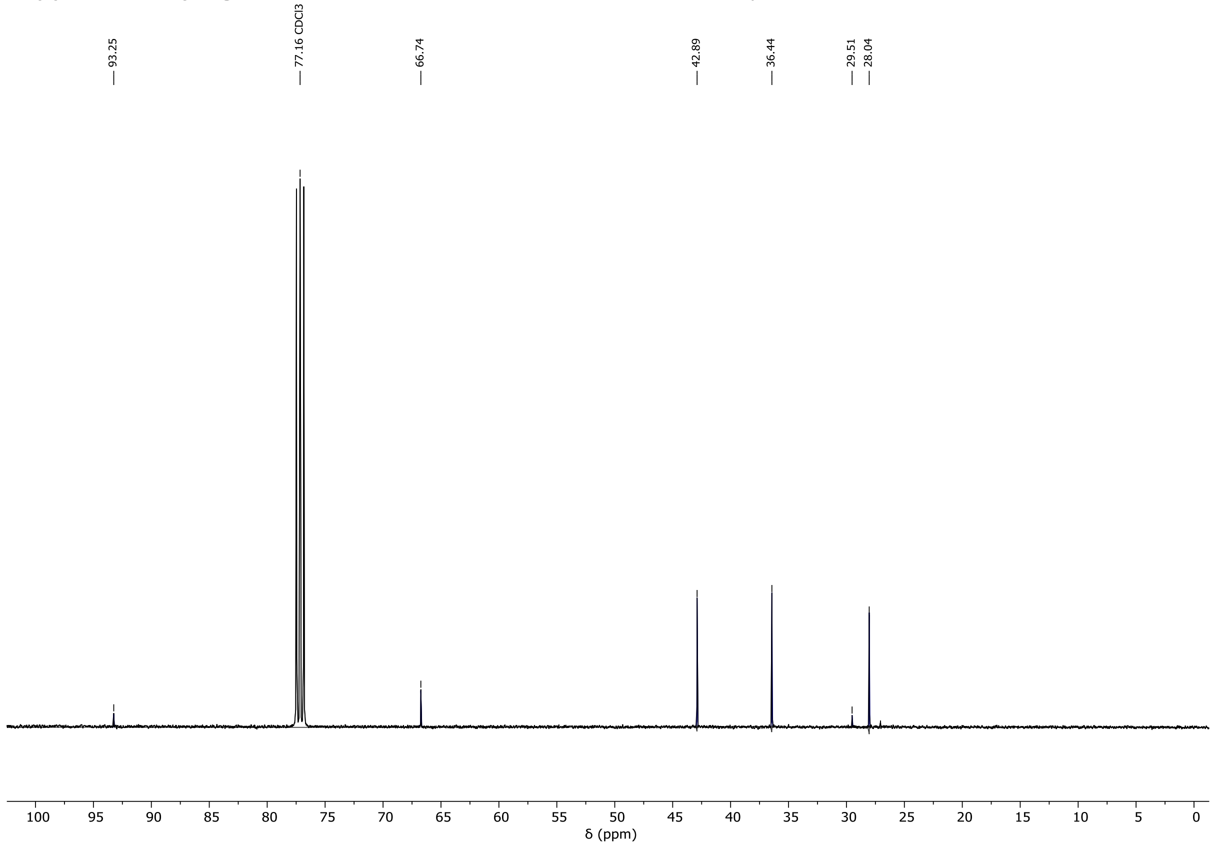

Supplementary Figure 198: ^{13}C-NMR ($CDCl_3$, 400 MHz) of ethinyl adamantane.

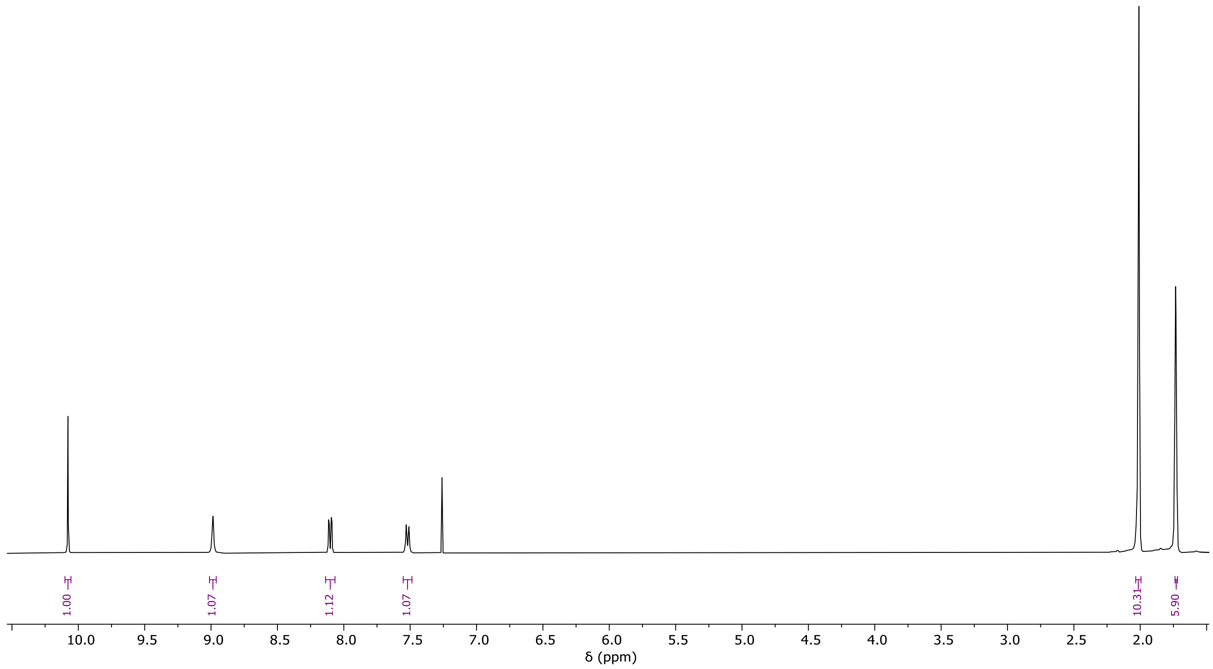

Supplementary Figure 199: ^{1}H-NMR ($CDCl_3$, 400 MHz) of 6-((adamantan-1-yl)ethynyl)nicotinaldehyde (**AdPyr-CHO**).

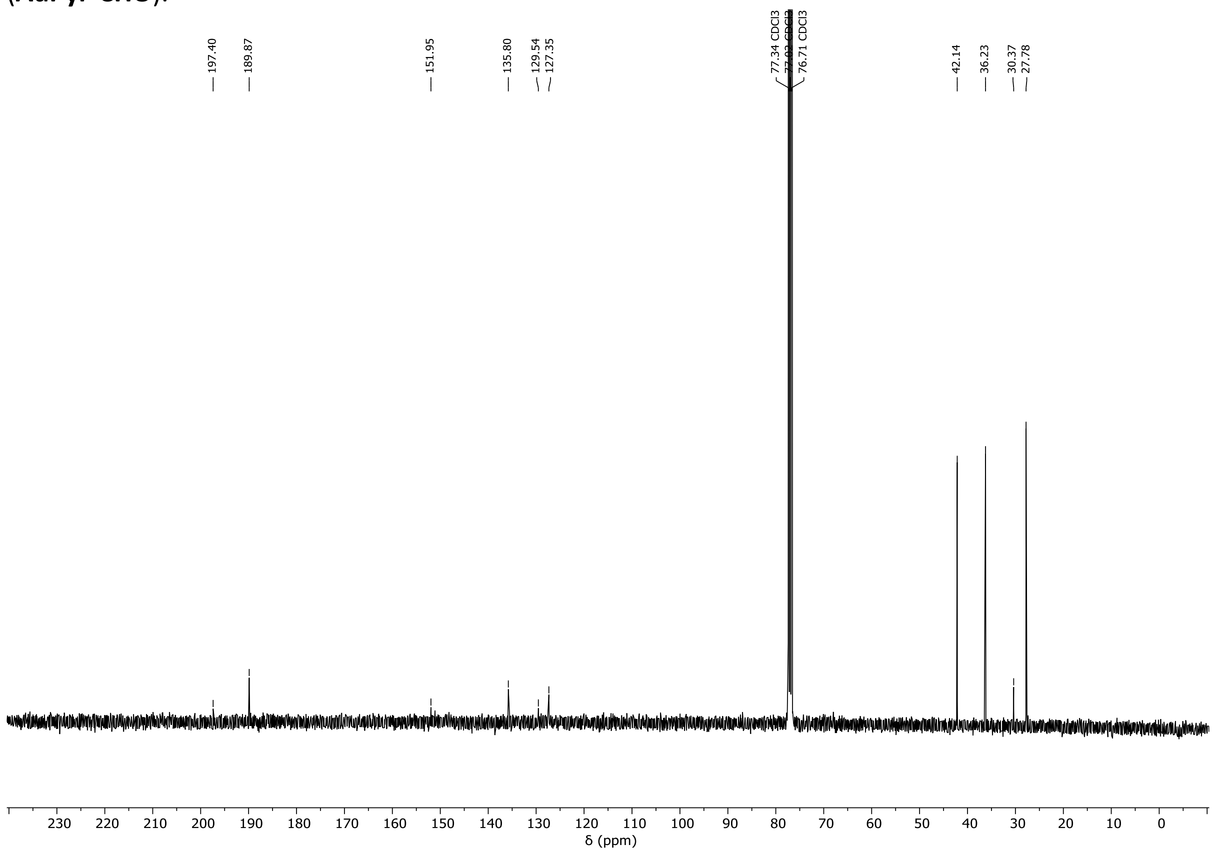

Supplementary Figure 200: ^{13}C-NMR ($CDCl_3$, 400 MHz) of 6-((adamantan-1-yl)ethynyl)nicotinaldehyde (**AdPyr-CHO**).

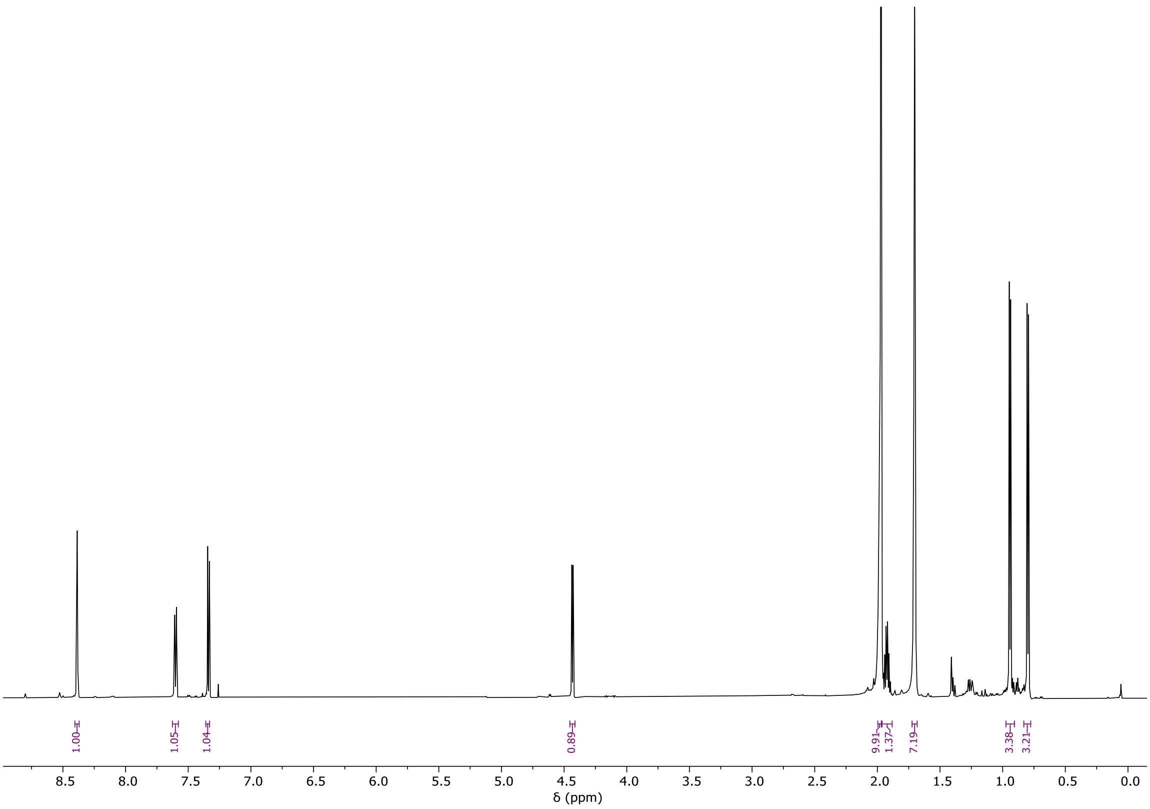

Supplementary Figure 201: ^{1}H-NMR ($CDCl_3$, 400 MHz) of 2-Methyl-(6-((trimethylsilyl)ethynyl)pyridine-3-yl)propanol **(TMSPyr-OH).**

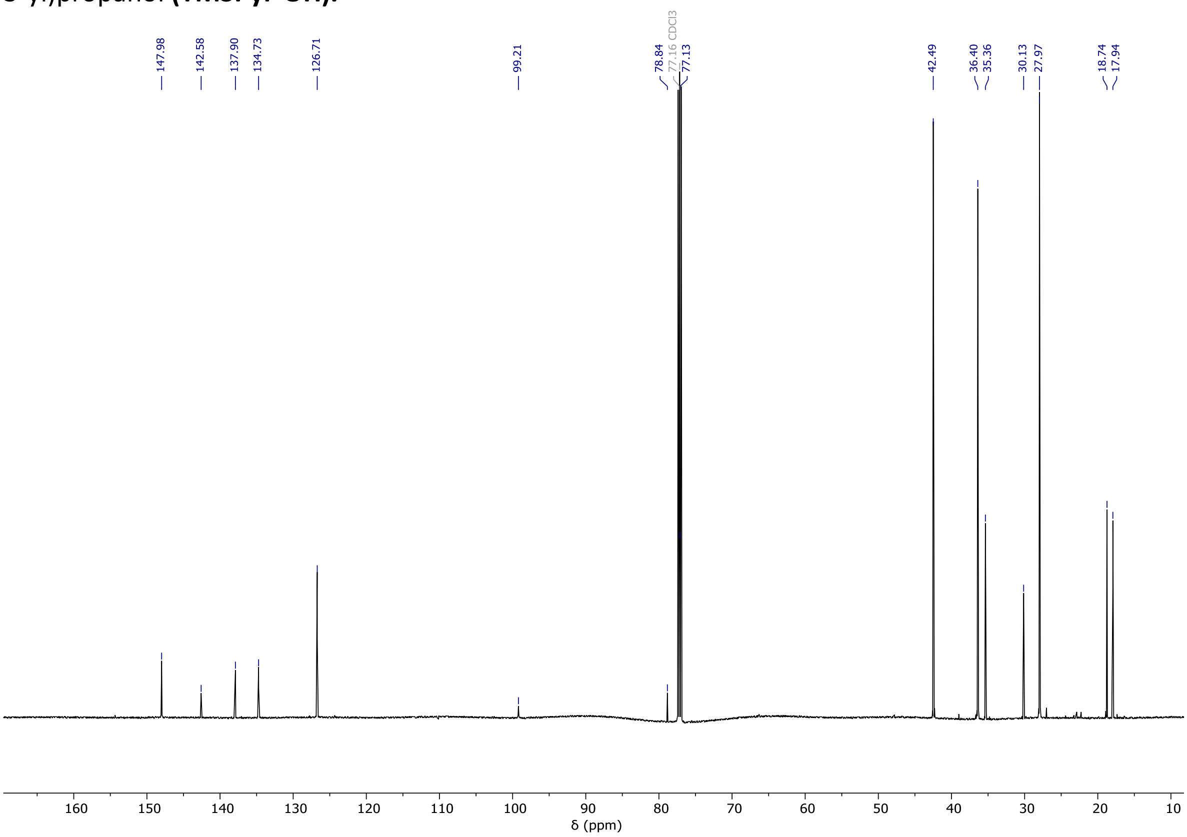

Supplementary Figure 202: ^{13}C-NMR ($CDCl_3$, 400 MHz) of 2-Methyl-(6-((trimethylsilyl)ethynyl)pyridine-3-yl)propanol (**TMSPyr-OH**).

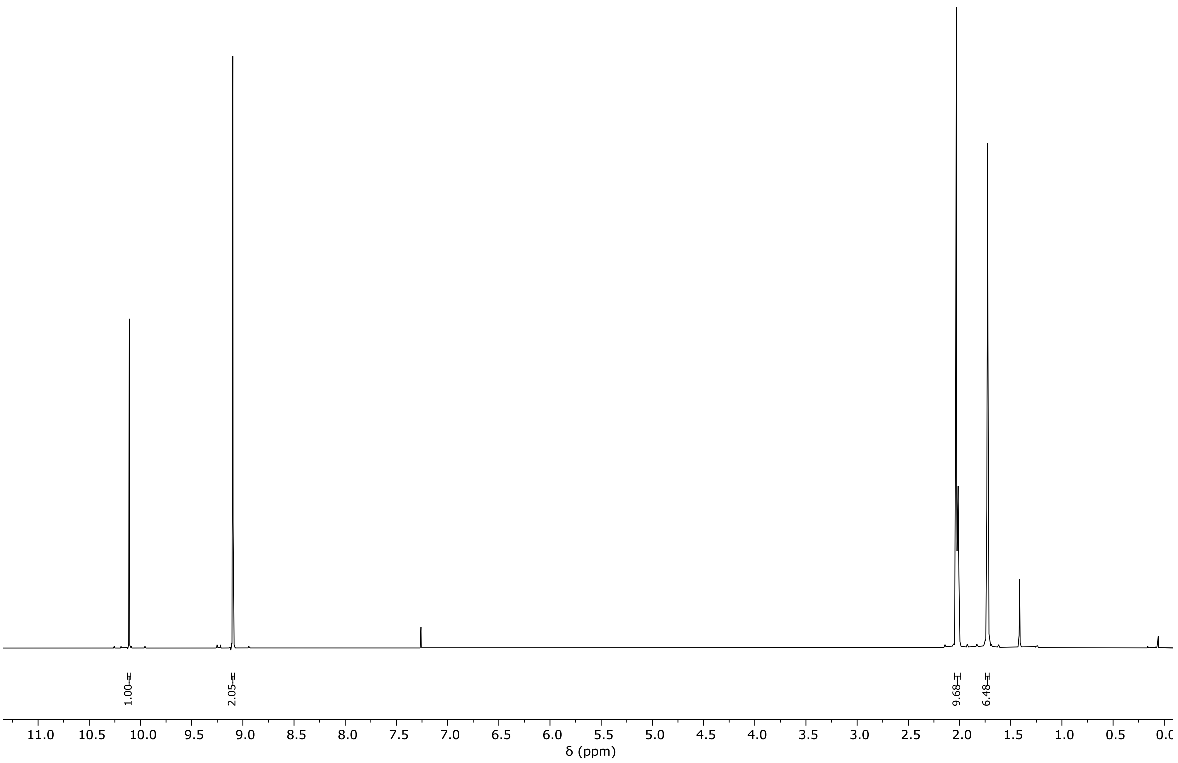

Supplementary Figure 203: ^{1}H-NMR ($CDCl_3$, 400 MHz) of 2-(Ethynyl-adamantyl)-pyrimidine-5-carbaldehyde (**AdPym-CHO**).

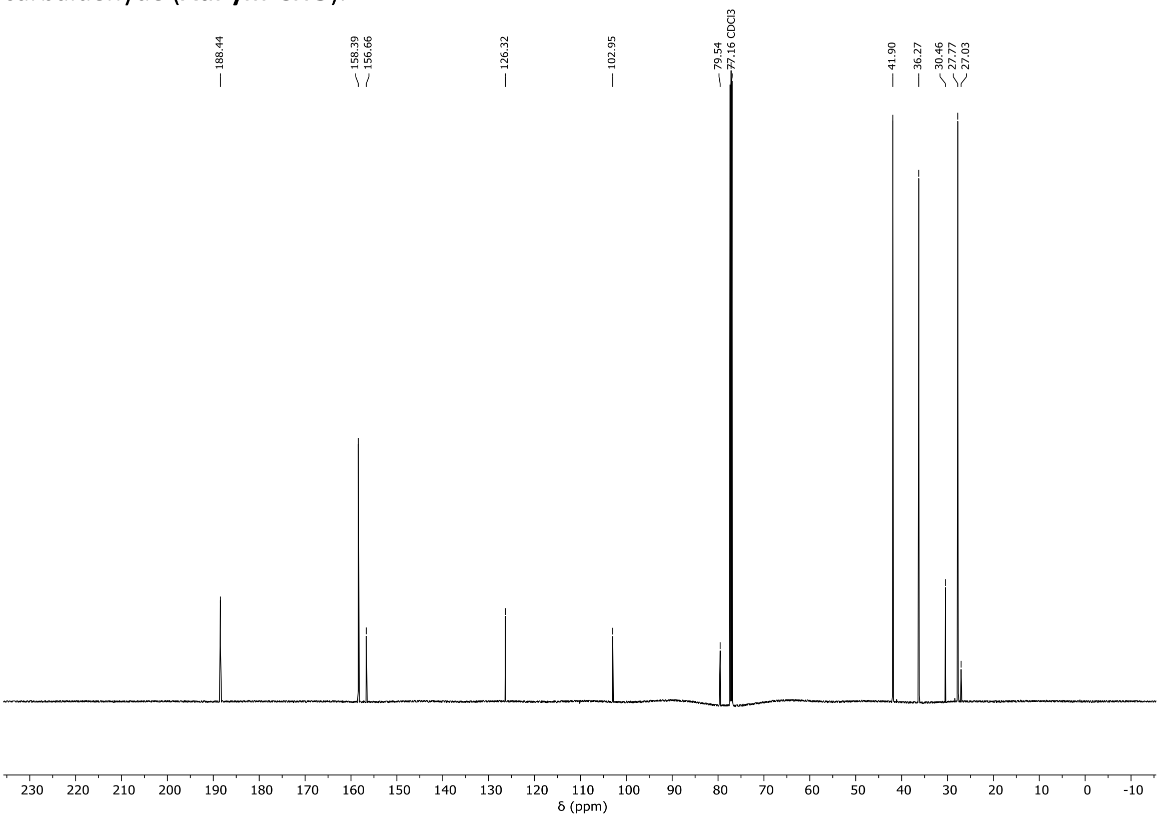

Supplementary Figure 204: ^{13}C-NMR ($CDCl_3$, 400 MHz) of 2-(Ethynyl-adamantyl)-pyrimidine-5-carbaldehyde (**AdPym-CHO**).

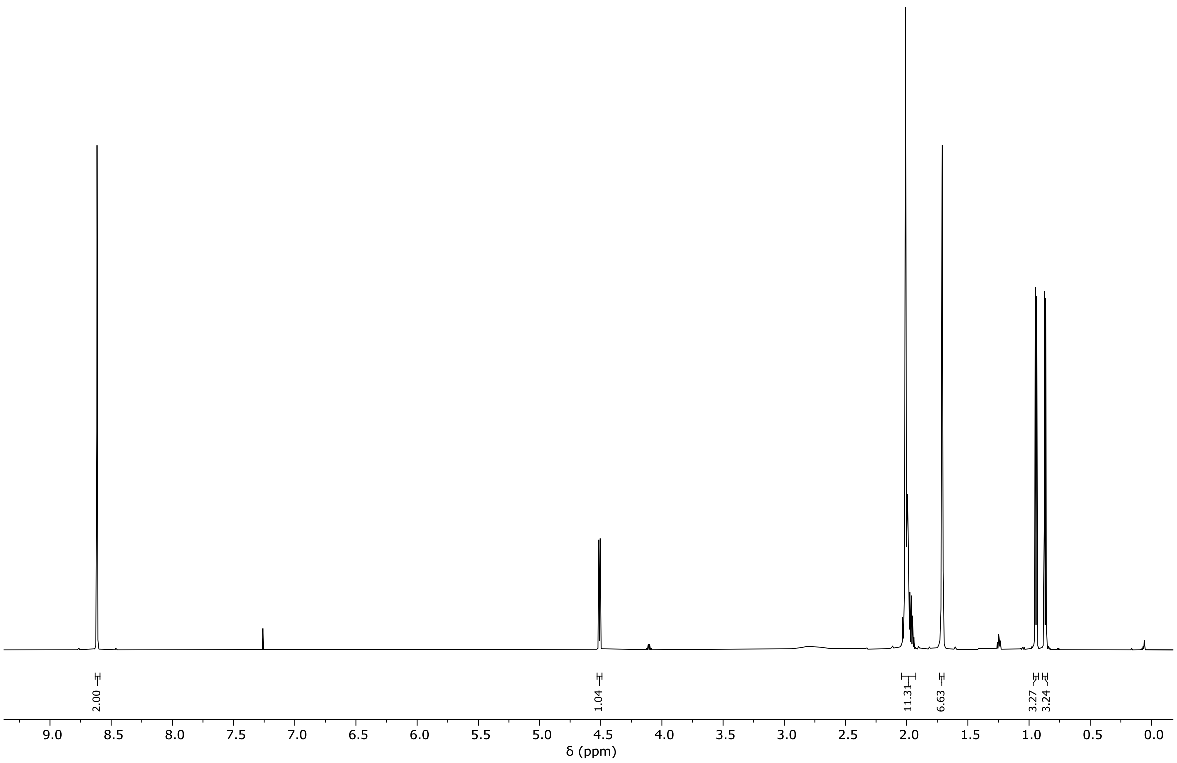

Supplementary Figure 205: ^{1}H-NMR ($CDCl_3$, 400 MHz) of 2-Methyl-((2-adamantylalkynyl)-5-pyrimidyl)propanol (**AdPym-OH**).

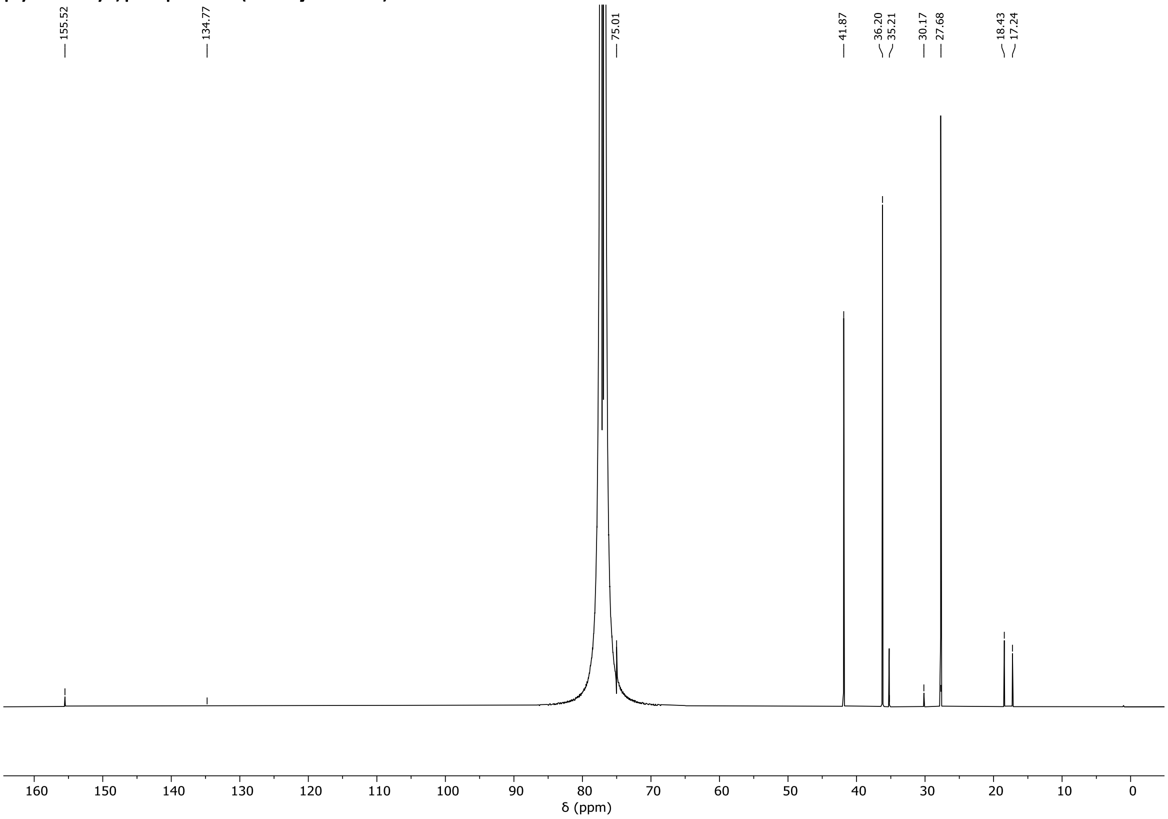

Supplementary Figure 206: ^{13}C-NMR ($CDCl_3$, 400 MHz) of 2-Methyl-((2-adamantylalkynyl)-5-pyrimidyl)propanol (**AdPym-OH**).

8 References

1. Fulmer, G.R., et al., *NMR Chemical Shifts of Trace Impurities: Common Laboratory Solvents, Organics, and Gases in Deuterated Solvents Relevant to the Organometallic Chemist.* Organometallics, 2010. **29**(9): p. 2176-2179.
2. Romagnoli, C., B. Sieng, and M. Amedjkouh, *Asymmetric Amplification Coupling Enantioselective Autocatalysis and Asymmetric Induction for Alkylation of Azaaryl Aldehydes.* European Journal of Organic Chemistry, 2015. **2015**(19): p. 4087-4092.
3. Athavale, S.V., et al., *Demystifying the asymmetry-amplifying, autocatalytic behaviour of the Soai reaction through structural, mechanistic and computational studies.* Nature Chemistry, 2020. **12**(4): p. 412-423.
4. Athavale, S.V., et al., *Structural Contributions to Autocatalysis and Asymmetric Amplification in the Soai Reaction.* Journal of the American Chemical Society, 2020. **142**(43): p. 18387-18406.
5. Kenny, R.T. and F. Liu, *Robust and Scalable Synthesis of Soai Aldehydes via Improved Barbier-type Halogen−lithium Exchange.* Asian Journal of Organic Chemistry, 2022. **11**(7): p. e202100787.
6. Busch, M., et al., *Systematic Studies using 2-(1-Adamantylethynyl)pyrimidine-5-carbaldehyde as a Starting Material in Soai's Asymmetric Autocatalysis.* Chemistry – A European Journal, 2009. **15**(33): p. 8251-8258.
7. Trapp, O., et al., *In Situ Mass Spectrometric and Kinetic Investigations of Soai's Asymmetric Autocatalysis.* Chemistry – A European Journal, 2020. **26**(68): p. 15871-15880.
8. Trapp, O., *Unified Equation for Access to Rate Constants of First-Order Reactions in Dynamic and On-Column Reaction Chromatography.* Analytical Chemistry, 2006. **78**(1): p. 189-198.
9. Trapp, O., *A novel software tool for high throughput measurements of interconversion barriers: DCXplorer.* Journal of Chromatography B, 2008. **875**(1): p. 42-47.
10. Ebert, K.& Ederer, H. *Computeranwendungen in der Chemie* (VCH, Weinheim, 1985).
